# Electrochemically Induced Reactivity of Ru–Arene Complexes Supported By Triarylphosphine Ligands

**DOI:** 10.1021/acs.inorgchem.6c01267

**Published:** 2026-06-17

**Authors:** Julia E. Fumo, Anthony N. Micci, Christopher Yang, Robert D. Pike, Davide Lionetti

**Affiliations:** a Department of Chemistry, 4400 Franklin & Marshall College, P.O. Box 3003, Lancaster, Pennsylvania 17604, United States; b Department of Chemistry, 8604 College of William & Mary, P.O. Box 8795, Williamsburg, Virginia 23187, United States

## Abstract

Half-sandwich ruthenium­(II) complexes supported by phosphine ligands typically undergo one-electron oxidation processes at potentials that depend on the identity of the phosphine ligand. While in noncoordinating solvents these electron transfer (ET) processes are often *quasi*reversible, electrochemical oxidation in the presence of exogenous ligands (such as in coordinating solvents) results in chemical reactivity of the nascent Ru­(III) species. Herein, we report the synthesis and spectroscopic, structural, and electrochemical characterization of several series of (*p*-cymene)Ru complexes, including dichloride, solvento, bis­(phosphine), and benzonitrile compounds. Based on extensive comparisons across these series of complexes and of Hammett analyses of the effects of phosphine-based substituents on Ru reduction potential, this ET-induced reactivity was confirmed to involve dissociation of the cymene moiety and incorporation of three nitrile ligands, with retention of the phosphine and chloride ligands present in the relative precursors. Investigations into the properties of the three isomers of [RuCl­(PPh_3_)_2_(NCCH_3_)_3_]^+^, the byproduct of ET-induced reactivity of the [(*p*-cymene)­RuCl­(PPh_3_)_2_]^+^ complex, indicated the *cis*-(PPh_3_)_2_-*fac*-(CH_3_CN)_3_ isomer as the likely form of the complex generated following electrochemical oxidation, suggesting that ET-induced ligand exchange results in replacement of the facially coordinated arene ligand with three monodentate ligands also in a *facial* arrangement.

## Graphical abstract

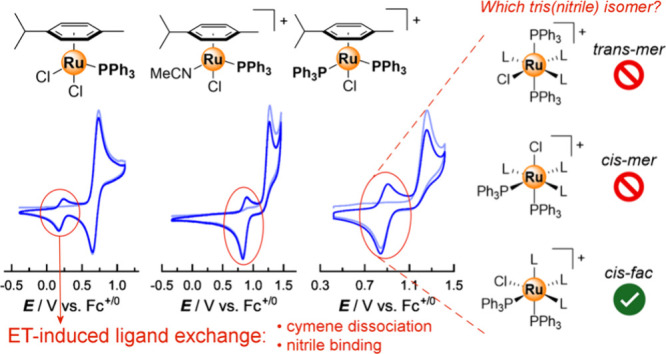

## Introduction

Piano-stool ruthenium complexes supported by neutral arene ligands are ubiquitous in organometallic chemistry due to their numerous applications in catalysis and biochemistry.^1–6^ The commercial availability of several [(arene)­RuCl_2_]_2_ compounds (especially [(*p*-cymene)­RuCl_2_]_2_) and the ability to readily install a varied set of additional ligands at Ru via cleavage of these dimeric precursors have enabled extensive structure–function studies of these complexes for diverse applications.^2,7^ Among these, neutral or cationic *p*-cymene–Ru complexes containing phosphine or phosphite ligands have been explored as catalysts for numerous organic transformations.^8–19^ In particular, compounds in this family have been shown to catalyze atom-transfer radical addition^20,21^ (ATRA) and polymerization^21–25^ (ATRP) reactions, for which insight into the electron transfer (ET) profile of a candidate catalyst could be particularly beneficial as the catalytic mechanism for these transformations involves an oxidation state change at the catalyst.^20^ Over the past three decades, a growing number of studies on the biochemical applications of these complexes have also been reported.^5,6,26^ The discovery of the antitumoral properties of the RAPTA family of compounds, supported by the 1,2,5-triaza-7-phosphaadamantane ligand, has led to the development of extensive libraries of (arene)­Ru complexes and to studies of their anticancer,^27–33^ antiparasitic,^34^ and antibacterial properties,^35^ greatly expanding the structural features of the ligands employed to support these motifs.

Although several compounds in this family have been characterized via electrochemical methods,^11,20,36–45^ a surprisingly modest number of investigations have employed systematic ET studies as a tool to elucidate how modifications on the phosphine framework, a common screening target in both catalytic^20^ and biochemical^44^ studies, influence ET. Additionally, while in noncoordinating solvents such as dichloromethane complexes in this family display *quasi*reversible electrochemical oxidation to Ru­(III),^11,20,36–40^ electrochemical studies in coordinating solvents (typically CH_3_CN) have revealed rapid ligand exchange reactivity in the nascent Ru­(III) species.^40,42–44^ As both catalytic and biochemical applications of complexes in this family often involve coordinating reagents or solvents, which may therefore engender ligand substitution processes analogous to those observed following ET, investigations into the potential downstream reactivity engendered by redox changes at Ru would be of particular interest.

In one of the few reports in this context, Wright and co-workers described the ET profile of a cymene–Ru complex supported by the PPh_2_(CH_2_)_3_Ph ligand (Scheme 1),^42^ which features a pendant arene that could bind to Ru via replacement of the cymene ligand. Oxidation of this complex in the presence of CH_3_CN results in formation of a new product whose composition was postulated as RuCl_2_(PR_3_)­(NCCH_3_)_3_, though spectroscopic characterization indicated a more symmetric compound (see SI). More recently, deAraujo and co-workers reported cationic complexes of the form [(*p*-cymene)­RuCl­(Ar_2_P–N^R^–PAr_2_)]^+^, supported by bidentate, bis­(phosphine) ligands (Ar_2_P–N^R^–PAr_2_; Ar = Ph, *p*-tolyl; R = CH_2_py, CH_2_Ph).^43^ Upon oxidation to Ru­(III) in CH_3_CN, these complexes are converted to products formulated as [RuCl­(Ar_2_P–N^R^–PAr_2_)­(NCCH_3_)_3_]^+^ species, which could be chemically prepared from the original Ru­(II) complexes via thermolysis. The electrochemically generated tris­(nitrile) products were identified as the [*fac*-(NCCH_3_)_3_-RuCl­(Ar_2_P–N^R^–PAr_2_)]^+^ isomers of these complexes (Scheme 1). Wonrath, Boeré, and co-workers also reported that [(*p*-cymene)­Ru] complexes bearing both phosphine and N-donor ligands (e.g., nitrile, pyridine) display similar ET-induced reactivity (Scheme 1).^44^

**Scheme 1:**
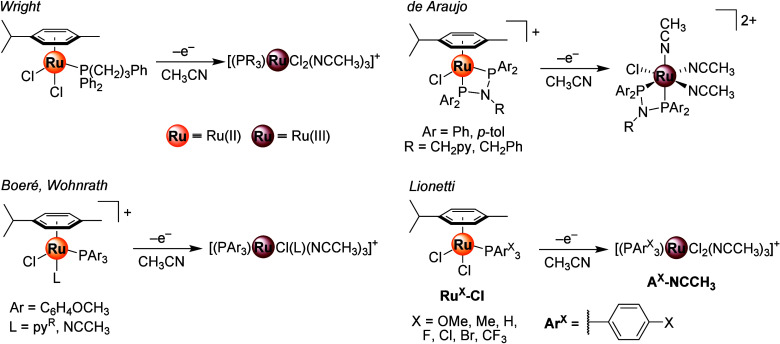
Chemical Reactivity of (cymene)Ru Complexes Induced by Electrochemical Oxidation

Recently, our group reported electrochemical investigations of a series of complexes supported by *para*-substituted triarylphosphine ligands (PAr^X^_3_; Ar^X^ = 4-X-C_6_H_4_, X = OMe, Me, H, F, Br, Cl, CF_3_), which are among the most investigated compounds in this family.^40^ (*p*-cymene)­RuCl_2_(PAr^X^_3_) complexes (**Ru** ^**X**^ **-Cl**, Scheme 1) undergo oxidation-induced reactivity (an EC process^46^) to generate new electroactive products with more negative reduction potentials. Leveraging Hammett analyses^47–50^ and studies of complexes of other arene ligands (benzene, mesitylene, hexamethylbenzene), these products were assigned as tris­(nitrile) complexes RuCl_2_(PAr^X^_3_)­(NCCH_3_)_3_ (**A** ^**X**^ **-NCCH**_**3**,_ Scheme 1), suggesting the general nature of reactivity with exogenous ligands in [(arene)­Ru] complexes supported by phosphine ligands.^42–44^

Our original conclusions hinged on two key considerations; dissociation of the arene ligand was supported by the observation that the byproducts generated by complexes containing benzene or *p*-cymene ligands had identical reduction potentials, suggesting that the arene was not retained in these products. However, since complexes containing more sterically demanding arene ligands did not undergo the same ET-induced reactivity, the possibility that the byproducts of the benzene and cymene compounds possess coincidentally similar reduction potentials while retaining the arene ligand could not be ruled out. Second, Hammett analyses were predicated on the assumption that substituent effects on reduction potential should be additive for complexes in this family, resulting in similar Hammett reaction constant (ρ) values between dichloride precursors and tris­(nitrile) byproducts.^47–53^ However, though supported by literature precedent, this assumption was not fully tested in this system.

To provide additional evidence supporting the composition and geometry of the electrochemical oxidation byproducts of (*p*-cymene)­RuCl_2_(PAr^X^_3_) complexes, we report here synthetic, structural, and electrochemical studies on (*p*-cymene)Ru complexes including solvento ([(*p*-cymene)­RuCl­(PAr^X^_3_)­(NCCH_3_)]^+^; **Ru** ^**X**^ **-NCCH**_**3**_, Chart 1, left panel), bis­(phosphine) (([(*p*-cymene)­RuCl­(PAr^X^_3_)_2_]^+^; **Ru** ^**X**^ **-PAr**_**3**_), and benzonitrile ([(*p*-cymene)­RuCl­(PPh_3_)­(NCAr^X^)]^+^; **Ru** ^**H**^ **-NCAr** ^**X**^) compounds. Hammett analyses of these series of complexes enabled validation of our hypothesis that the number of substituted phosphine ligands contained in complexes in this family could be identified from the effects of peripheral substituents on reduction potential at Ru. These investigations also confirmed the retention of Cl^–^ ligands and incorporation of nitrile motifs in the products of the EC processes observed in voltammetric studies. Taken together, these studies provided substantive evidence that oxidation of dichloride, solvento, and bis­(phosphine) complexes in this family results in dissociation of cymene and binding of three exogenous ligands to generate (PAr^X^_3_)­RuCl_2_(L)_3_ (**A** ^**X**^ **-L**, Chart 1), [(PAr^X^_3_)­RuCl­(L)_4_]^+^ (**B** ^**X**^ **-L**), or [(PAr^X^_3_)_2_RuCl­(L)_3_]^+^ (**C** ^**X**^ **-L**) complexes (L = NCCH_3_, NCAr^X^), respectively.

**Chart 1:**
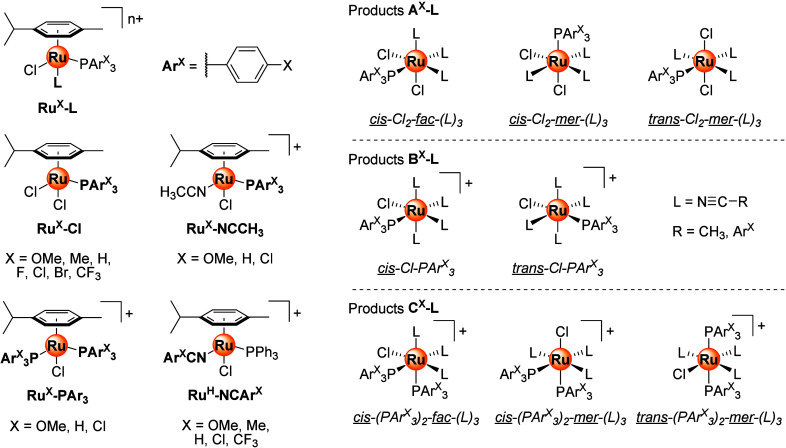
Cymene-Ru Complexes Investigated in the Present Study (Left Panel) and Possible Isomers of Electrochemical Oxidation Byproducts (Right Panel)

Three different isomers of complexes of the form RuCl_2_(PPh_3_)­(NCR)_3_ are possible based on the relative arrangement of the two chloride ligands (in *cis* or *trans* configuration) and of the three nitrile ligands (*mer* or *fac* configuration; Chart 1, right panel). In literature reports of ET-induced reactivity of (arene)­Ru complexes, the isomer of the resulting byproduct was identified only for complexes of bidentate bis­(phosphine) ligands. To probe the structure of the tris­(nitrile) byproducts in complexes of monodentate phosphines, different isomers of the **C** ^**H**^ **-L** complex (derived from ET-induced reactivity of bis­(phosphine) complex **Ru** ^**H**^ **-PAr**_**3**_) were investigated. Structural and electrochemical characterization indicated that dissociation of cymene upon oxidation results in binding of three facially arranged nitrile ligands. The ET profile of these various series of complexes and their oxidation-induced reactivity, the influence of ligand-based substituents on ET, and our work to independently access the products of these ligand substitution reactions through chemical means and evaluate their ET profiles are discussed.

## Experimental Section

### General Considerations

All manipulations were carried out on the benchtop without exclusion of air or water except where otherwise noted. All solvents were of commercial grade; all chemicals were obtained from major commercial suppliers and used as received. [(*p*-cymene)­RuCl_2_]_2_ was obtained from Ambeed; phosphine ligands were obtained from Ambeed, TCI, or Sigma-Aldrich. Silver salts and RuCl_2_(PPh_3_)_3_ were obtained from Strem. **Ru** ^**X**^ **-Cl** complexes were prepared as previously described.^40^ Deuterated NMR solvents were purchased from TCI, Oakwood, Sigma-Aldrich, or Cambridge Isotope Laboratories. ^1^H, ^13^C, ^19^F, and ^31^P NMR spectra were collected on a 500 MHz Agilent spectrometer or on a 400 MHz Bruker NeoAvance spectrometer and referenced to the residual protio solvent signal in the case of ^1^H and ^13^C.^54^ ^31^P NMR spectra were referenced to 85% aqueous H_3_PO_4_ and ^19^F NMR spectra were referenced to CFCl_3_ following the recommended scale based on ratios of absolute frequencies (Ξ).^55,56^ Chemical shifts (δ) are reported in units of ppm and coupling constants (*J*) are reported in Hz. Numbering schemes for assignment of spectroscopic signatures for new compounds are shown in Figures 1 and 2. Elemental analyses were performed by Midwest Microlab (Indianapolis, IN). No uncommon hazards are noted.

**Figure 1:**
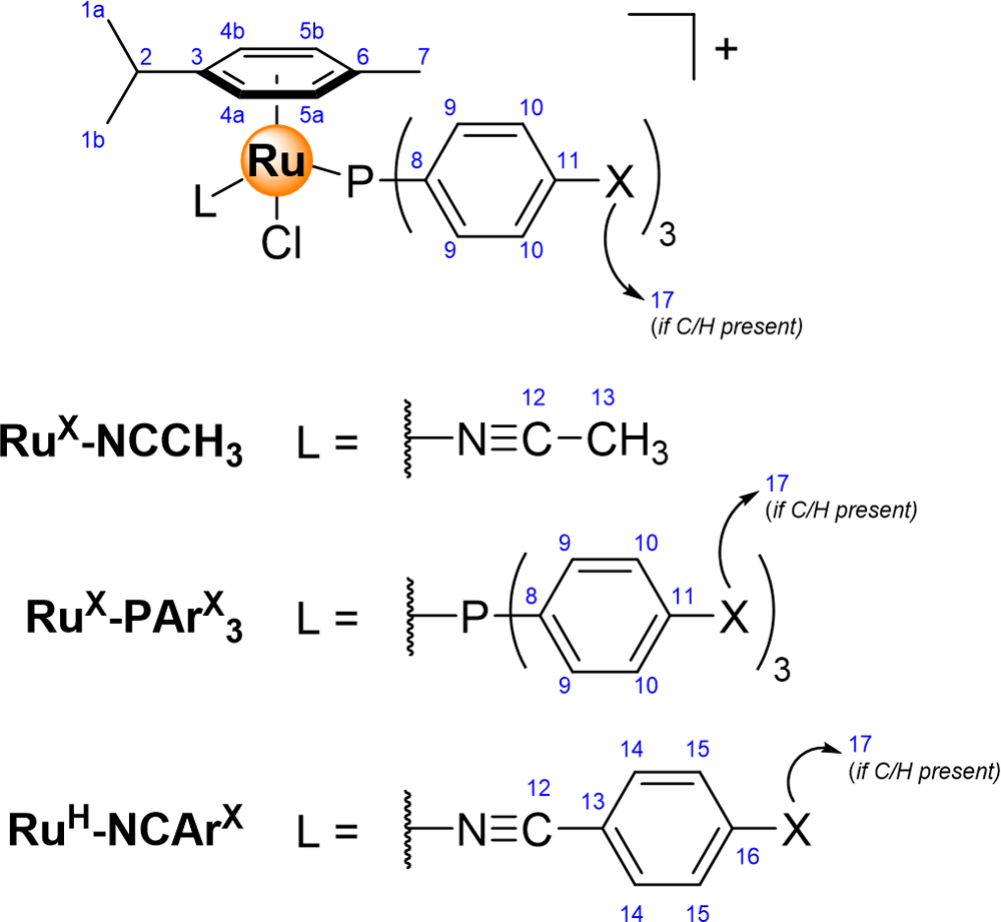
Numbering scheme for spectroscopic assignments for complexes **Ru** ^X^ **-L**.

**Figure 2:**
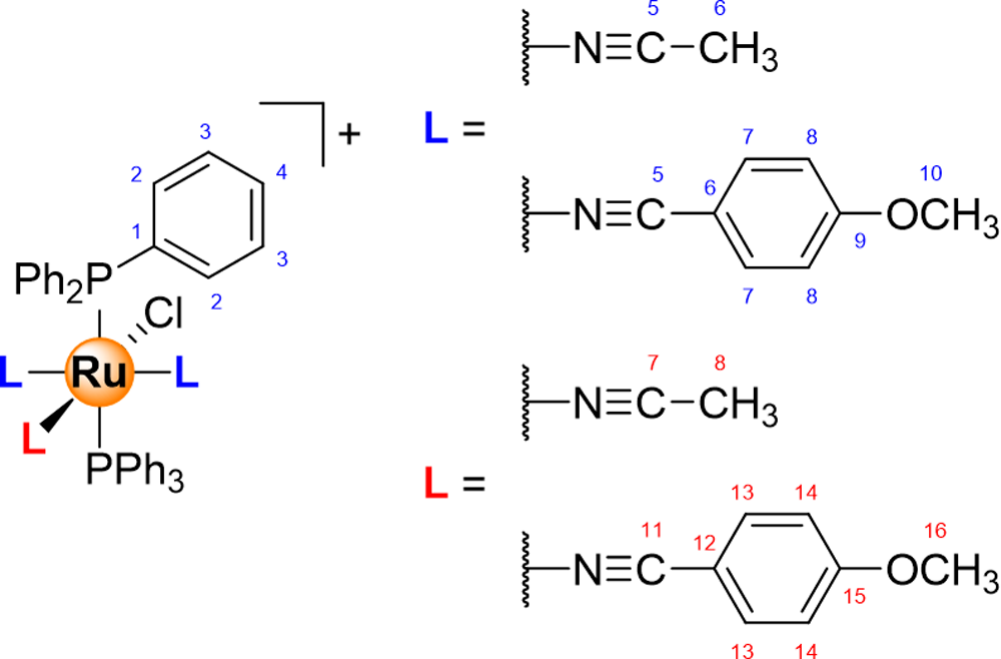
Numbering scheme for spectroscopic assignments for complexes **C** ^H^ **-L**.

### X-ray Crystallography

Crystals were mounted on MiTeGen Micro Mounts using Paratone oil. All measurements were made at 100 K using Mo Kα radiation on a Bruker-AXS D8 Venture four-circle diffractometer, equipped with a microfocus tube and a Photon 3 CPAD detector. Initial space group determination was based on a fast scan with 180 frames. The data were reduced using SAINT+,^57,58^ and empirical absorption correction applied using SADABS.^59^ Structures were solved using intrinsic phasing (SHELXT).^60^ Least-squares refinement for all structures was carried out on *F* ^2^. The non-hydrogen atoms were refined anisotropically. Hydrogen atoms were placed in riding positions and refined isotropically. Structure refinement and the calculation of derived results were performed using the Olex software equipped with the SHELX package of computer programs.^61,62^

### Electrochemistry

Electrochemical experiments were carried out under N_2_ or Ar. 0.1 *M* tetra­(*n*-butylammonium) hexafluorophosphate (electrochemical grade, Sigma-Aldrich) in acetonitrile or dichloromethane served as the supporting electrolyte. Measurements were made with a Pine Research WaveDriver 40 potentiostat/galvanostat, a CH Instruments Model 604C electrochemical analyzer, or a Pine Research WaveNow potentiostat, using a standard three-electrode configuration. In CH_3_CN, the working electrode was the basal plane of highly oriented pyrolytic graphite (HOPG; GraphiteStore.com, Buffalo Grove, IL; surface area 0.090 cm^2^). In CH_2_Cl_2_, the working electrode was a circular glassy carbon electrode with a PTFE shroud (Pine Research; surface area: 0.0707 cm^2^). The counter electrode was a platinum wire (Kurt J. Lesker, Jefferson Hills, PA; 99.99%, 0.5 mm diameter) and a silver wire immersed in CH_3_CN electrolyte solution and separated from the working solution by a Vycor frit (CH Instruments, Sigma-Aldrich) served as a pseudoreference electrode. Ferrocene was added to the electrolyte solution at the conclusion of each experiment (∼1 m*M*); the midpoint potential of the ferrocenium/ferrocene couple (denoted as Fc^+/0^) served as an external standard for comparison of the recorded potentials. Concentrations of analyte for cyclic voltammetry were typically 2–3 m*M.*

### Synthesis of Complexes **Ru^X^-NCCH_3_**

#### General Procedure

To a solution of **Ru** ^**X**^ **-Cl** (1 equiv) in CH_3_CN was added AgPF_6_ (1 equiv) as a CH_3_CN solution. The solution immediately turned from orange to yellow, and a colorless precipitate formed. After 30 min, the solution was filtered through Celite to remove AgCl. The volume was reduced to 1 mL *in vacuo* and Et_2_O was added to precipitate the product. The product was isolated via filtration through a fritted glass funnel, washed with Et_2_O, and air-dried.

### Ru^OMe^-NCCH_3_

Synthesized via the above procedure using 0.490 g **Ru** ^**OMe**^ **-Cl** (0.744 mmol) and 0.188 g AgPF_6_ (0.0744 mmol). Yellow powder; yield 0.450 g (74.8%). ^1^H NMR (500 MHz, CDCl_3_): δ 7.48 (dd, ^2^ *J*_H,P_ = 10.5 Hz, ^3^ *J*_H,H_ = 8.7 Hz, 6H, C9–*H*), 6.99 (dd, ^3^ *J*_H,H_ = 8.7 Hz, ^3^ *J*_H,P_ = 1.5 Hz 6H, C10–*H*), 6.07 (d, ^3^ *J*_H,H_ = 6.3 Hz, 1H, C4b–*H*), 5.89 (d, ^3^ *J*_H,H_ = 6.1 Hz, 1H, C4a–*H*), 5.32 (d, ^3^ *J*_H,H_ = 6.1 Hz, 1H, C5a–*H*), 4.61 (d, ^3^ *J*_H,H_ = 6.3 Hz, 1H, C5b–*H*), 3.84 (s, 9H, C17–*H*), 3.03 (hept, ^3^ *J*_H,H_ = 6.9 Hz, 1H, C2–*H*), 1.99 (s, 3H, C13–*H*), 1.75 (s, 3H, C7–*H*), 1.36 (d, ^3^ *J*_H,H_ = 6.9 Hz, 3H, C1a–*H*), 1.34 (d, ^3^ *J*_H,H_ = 6.9 Hz, 3H, C1b–*H*) ppm. ^31^P­{^1^H} NMR (202 MHz, CDCl_3_) δ 32.23 (s, *P*Ar_3_), – 144.25 (hept, ^1^ *J*_P,F_ = 712.7 Hz, *P*F_6_^–^) ppm. See the SI for ^13^C­{^1^H} and ^19^F data. Elemental analysis: calculated for C_33_H_38_ClF_6_NO_3_P_2_Ru (%): C 48.99, H 4.73, N 1.73; found: C 48.66, H 4.71, N 1.70.

### Ru^H^-NCCH_3_

Synthesized via the above procedure using 0.2093 g **Ru** ^**H**^ **-Cl** (0.3682 mmol) and 0.0931 g AgPF_6_ (0.368 mmol). Yellow powder; yield 0.2298 g (86.80%). Spectroscopic data was consistent with previous literature reports.^63^

### Ru^Cl^-NCCH_3_

Synthesized via the above procedure using 0.2201 g **Ru** ^**Cl**^ **-Cl** (0.3276 mmol) and 0.0828 g AgPF_6_ (0.328 mmol). Yellow powder; yield 0.166 g (61.6%). ^1^H NMR (500 MHz, CDCl_3_): δ 7.50 (m, 12H, C9–*H* and C10–*H*), 6.19 (dd, ^3^ *J*_H,H_ = 6.3 Hz, ^4^ *J*_H,H_ = 1.5 Hz, 1H, C4b–*H*), 5.97 (d, ^3^ *J*_H,H_ = 6.1 Hz, 1H, C4a–*H*), 5.41 (dd, ^3^ *J*_H,H_ = 6.1 Hz, ^4^ *J*_H,H_ = 1.4 Hz, 1H, C5a–*H*), 4.68 (d, ^3^ *J*_H,H_ = 6.3 Hz, 1H, C5b–*H*), 3.01 (hept, ^3^ *J*_H,H_ = 7.0 Hz, 1H, C2–*H*), 2.02 (s, 3H, C13–*H*), 1.74 (s, 3H, C7–*H*), 1.38 (d, ^3^ *J*_H,H_ = 7.0 Hz, 3H, C1–*H*), 1.35 (d, ^3^ *J*_H,H_ = 7.1 Hz, 3H, C1b–*H*) ppm. ^31^P­{^1^H} NMR (202 MHz, CDCl_3_) δ 35.17 (s, *P*Ar_3_), – 144.29 (hept, ^1^ *J*_P,F_ = 712.7 Hz, *P*F_6_^–^) ppm. See the SI for ^13^C­{^1^H} and ^19^F data. Elemental analysis: calculated for C_30_H_29_Cl_4_F_6_NP_2_Ru (%): C 43.82, H 3.55, N 1.70; found: C 43.74, H 3.55, N 1.35.

### Synthesis of **Ru^OMe^-PAr_3_**

**Ru** ^**OMe**^ **-NCCH**_**3**_ (0.1500 g, 0.1854 mmol, 1 equiv) and tris­(4-methoxyphenyl)­phosphine (0.1966 g, 0.5579 mmol, 3 equiv) were dissolved in CHCl_3_ and the solution heated to reflux for 1 h. After cooling, the volume of the solution was reduced *in vacuo* and diethyl ether added to precipitate the product, which was collected by filtration through a fritted glass funnel and air-dried. Yellow powder; yield 0.1646 g (79.24%). ^1^H NMR (500 MHz, CDCl_3_): δ 7.32 (m, 12H, C9–*H*), 6.74 (d, ^3^ *J*_H,H_ = 8.5 Hz, 12H, C10–*H*), 5.51 (dt, ^3^ *J*_H,H_ = 6.5 Hz, ^2^ *J*_H,P_ = 2.1 Hz, 2H, C5–*H*), 5.08 (d, ^3^ *J*_H,H_ = 6.2 Hz, 2H, C4–*H*), 3.82 (s, 18H, C17–*H*), 2.68 (hept, *J* = 6.8 Hz, 1H, C3–*H*), 1.22 (d, *J* = 7.0 Hz, 6H, C1–*H*), 1.19 (s, 3H, C7–*H*) ppm. ^31^P­{^1^H} NMR (202 MHz, CDCl_3_) δ 18.07 (s, *P*Ar_3_), – 144.44 (hept, ^1^ *J*_P,F_ = 712.8 Hz) ppm. See the SI for ^13^C­{^1^H} and ^19^F data. Elemental analysis: calculated for C_52_H_56_ClF_6_O_6_P_3_Ru (%): C 55.74, H 5.04, N 0.00; found: C 55.72, H 5.00, N 0.00.

### Synthesis of **Ru^H^-PAr_3_**

**Ru** ^**H**^ **-NCCH**_**3**_ (0.2117 g, 0.2944 mmol, 1 equiv) and triphenylphosphine (0.3861 g, 1.472 mmol, 5 equiv) were dissolved in CHCl_3_ and the solution heated to reflux for 3 h. After cooling, the volume of the solution was reduced *in vacuo* and diethyl ether added to precipitate the product, which was collected by filtration through a fritted glass funnel. The product was redissolved in CHCl_3_ and a further 5 equiv. PPh_3_ added, and the solution was heated to reflux for 3 h. After cooling, the volume of the solution was reduced *in vacuo* and diethyl ether added to precipitate the product, which was collected by filtration through a fritted glass funnel and air-dried. Yellow powder; yield 0.242 g (87.4%). Spectroscopic data was consistent with previous literature reports.^64^

### Synthesis of **Ru^Cl^-PAr_3_**

Attempted synthesis of **Ru** ^**Cl**^ **-PAr**_**3**_ via the same procedures described above for **Ru** ^**OMe**^ **-PAr**_**3**_ and **Ru** ^**H**^ **-PAr**_**3**_ revealed that replacement of CH_3_CN by tris­(4-chlorophenyl)­phosphine was slow even in the presence of excess phosphine under reflux conditions, while prolonged heating led to generation of undesired byproducts. Therefore, **Ru** ^**Cl**^ **-PAr**_**3**_ was prepared according to the following procedure: to a solution of **Ru** ^**Cl**^ **-Cl** (0.0687 g, 0.102 mmol, 1 equiv) in acetone was added an acetone solution of AgPF_6_ (0.0259 g, 0.102 mmol, 1 equiv), upon which a colorless precipitate formed. After 10 min, the solution was filtered through Celite and the solvent removed *in vacuo*. The oily residue was dissolved in CH_2_Cl_2_ and tris­(4-chlorophenyl)­phosphine (0.1122 g, 0.3069 mmol, 3 equiv) was added to the stirred solution. The color of the solution immediately became lighter. After 40 min, the slightly turbid solution was filtered, and the volume of the filtrate was reduced to ∼2 mL. Addition of hexanes resulted in formation of a slightly oily precipitate, which became solid upon sonication for 15 min. The solid was collected via filtration through a fritted glass funnel, washed with hexanes (x2) and pentane (x2), and air-dried. Yellow powder; yield 0.0915 g (78.2%). ^1^H NMR (500 MHz, CDCl_3_): δ 7.34–7.27 (m, 24H, C9–*H* and C10–*H*), 5.67 (dt, ^3^ *J*_H,H_ = 6.2 Hz, ^2^ *J*_H,P_ = 2.1 Hz, 2H, C5–*H*), 5.16 (d, ^3^ *J*_H,H_ = 6.3 Hz, 2H, C4–*H*), 2.63 (hept, *J* = 7.1 Hz, 1H, C2–*H*), 1.22 (d, *J* = 7.0 Hz, 6H, C1–*H*), 1.17 (s, 3H, C7–*H*) ppm. ^31^P­{^1^H} NMR (202 MHz, CDCl_3_) δ 20.62 (s, *P*Ar_3_), – 144.56 (hept, ^1^ *J*_P,F_ = 713.4 Hz) ppm. See the SI for ^13^C­{^1^H} and ^19^F data. Elemental analysis: calculated for C_46_H_38_Cl_7_F_6_P_3_Ru: C 48.17, H 3.34, N 0.00; found: C 48.16, H 3.30, N 0.00.

### Synthesis of Complexes **Ru^H^-NCAr^X^**

#### General Procedure

To a CH_2_Cl_2_ solution of **Ru** ^**H**^ **-Cl** (1 equiv) and the desired substituted benzonitrile NCAr^X^ (5 equiv) was added AgPF_6_ (1 equiv) as a CH_2_Cl_2_ solution. Within minutes, the dark orange solution lightened to yellow in color and a colorless precipitate formed. After 15 min, the solution was filtered through Celite and the filtrate reduced in volume to ∼1 mL *in vacuo*. Hexane or Et_2_O was added to precipitate the desired product, which was collected via filtration through a fritted glass funnel, washed with hexane and pentane, then air-dried.

### Ru^H^-NCAr^OMe^

Synthesized via the above procedure using 0.0581 g **Ru** ^**H**^ **-Cl** (0.102 mmol), 0.0680 g 4-methoxybenzonitrile (0.511 mmol), and 0.0258 g AgPF_6_ (0.102 mmol). Yellow powder; yield 0.0781 g (94.4%). ^1^H NMR (500 MHz, CDCl_3_): δ 7.60 (m, 6H, C9–*H*), 7.45 (m, 9H, C10–*H* and C11–*H*), 7.31 (d, ^3^ *J*_H,H_ = 8.6 Hz, 2H, C15–*H*), 6.84 (d, ^3^ *J*_H,H_ = 8.7 Hz, 2H, C14–*H*), 6.29 (d, ^3^ *J*_H,H_ = 6.3 Hz, 1H, C4b–*H*), 5.99 (d, ^3^ *J*_H,H_ = 6.3 Hz, 1H, C4a–*H*), 5.42 (d, ^3^ *J*_H,H_ = 6.1 Hz, 1H, C5a–*H*), 4.82 (d, ^3^ *J*_H,H_ = 6.2 Hz, 1H, C5b–*H*), 3.83 (s, 3H, C17–*H*), 3.04 (hept, ^3^ *J*_H,H_ = 6.9 Hz, 1H, C2–*H*), 1.75 (s, 3H, C7–*H*), 1.37 (d, ^3^ *J*_H,H_ = 6.9 Hz, 3H, C1a–*H*), 1.36 (d, ^3^ *J*_H,H_ = 6.9 Hz, 3H, C1b–*H*) ppm. ^31^P­{^1^H} NMR (202 MHz, CDCl_3_) δ 35.47 (s, *P*Ar_3_), – 144.09 (hept, ^1^ *J*_P,F_ = 712.9 Hz) ppm. See the SI for ^13^C­{^1^H} and ^19^F data. Elemental analysis: calculated for C_36_H_36_ClF_6_NOP_2_Ru (%): C 53.31, H 4.47, N 1.73; found: C 53.10, H 4.49, N 1.77.

### Ru^H^-NCAr^Me^

Synthesized via the above procedure using 0.0542 g **Ru** ^**H**^ **-Cl** (0.0953 mmol), 0.0560 g 4-methylbenzonitrile (0.478 mmol), and 0.0241 g AgPF_6_ (0.0953 mmol). Yellow powder; yield 0.0708 g (93.4%). ^1^H NMR (500 MHz, CD_2_Cl_2_): δ 7.64–7.57 (m, 6H, C9–*H*), 7.54–7.43 (m, 9H, C10–*H* and C11–*H*), 7.25 (d, ^3^ *J*_H,H_ = 8.1 Hz, 2H, C15–*H*), 7.12 (d, ^3^ *J*_H,H_ = 8.2 Hz, 2H, C14–*H*), 6.03 (d, ^3^ *J*_H,H_ = 6.3, ^4^ *J*_H,H_ = 1.5 Hz, 1H, C4b–*H*), 5.60 (d, ^3^ *J*_H,H_ = 6.1, ^4^ *J*_H,H_ = 1.4 Hz, 1H, C4a–*H*), 5.53 (d, ^3^ *J*_H,H_ = 6.3, ^4^ *J*_H,H_ = 1.4 Hz, 1H, C5b–*H*), 5.00 (d, ^3^ *J*_H,H_ = 6.2 Hz, 1H, C5a–*H*), 2.84 (hept, ^3^ *J*_H,H_ = 7.0 Hz, 1H, C2–*H*), 2.42 (s, 3H, C17–*H*), 1.91 (s, 3H, C7–*H*), 1.36 (d, ^3^ *J*_H,H_ = 7.0 Hz, 3H, C1a–*H*), 1.30 (d, ^3^ *J*_H,H_ = 6.9 Hz, 3H, C1b–*H*) ppm. ^31^P­{^1^H} NMR (162 MHz, CDCl_3_) δ 33.49 (s, *P*Ar_3_), – 144.39 (hept, ^1^ *J*_P,F_ = 712.8 Hz) ppm. See the SI for ^13^C­{^1^H} and ^19^F data. Elemental analysis: calculated for C_36_H_36_ClF_6_NP_2_Ru (%): C 54.38, H 4.56, N 1.76; found: C 53.97, H 4.57, N 1.77.

### Ru^H^-NCAr^H^

Synthesized via the above procedure using 0.0606 g **Ru** ^**H**^ **-Cl** (0.107 mmol), 0.0550 g benzonitrile (0.533 mmol), and 0.0270 g AgPF_6_ (0.107 mmol). Yellow powder; yield 0.0779 g (93.5%). ^1^H NMR (400 MHz, CD_2_Cl_2_): δ 7.66 (tt, 1H, ^3^ *J*_H,H_ = 7.6, ^4^ *J*_H,H_ = 1.2 Hz, C16–*H*), 7.63–7.58 (m, 6H, C9–*H*), 7.54–7.42 (m, 11H, C10–*H*, C11–*H*, C15–*H*), 7.24 (m, 2H, C14–*H*), 6.04 (dd, ^3^ *J*_H,H_ = 6.2, ^2^ *J*_H,P_ = 1.5 Hz, 1H, C5b–*H*), 5.65 (dd, ^3^ *J*_H,H_ = 6.1, ^2^ *J*_H,P_ = 1.4 Hz, 1H, C5a–*H*), 5.54 (dd, ^3^ *J*_H,H_ = 6.1, ^2^ *J*_H,P_ = 1.4 Hz, 1H, C4b–*H*), 5.00 (dd, ^3^ *J*_H,H_ = 6.2, ^2^ *J*_H,P_ = 1.7 Hz, 1H, C4a–*H*), 2.86 (hept, ^3^ *J*_H,H_ = 6.9 Hz, 1H, C2–*H*), 1.89 (d, ^4^ *J*_H,P_ = 0.7 Hz, 3H, C7–*H*), 1.37 (d, ^3^ *J*_H,H_ = 6.9 Hz, 3H, C1a–*H*), 1.31 (d, ^3^ *J*_H,H_ = 6.9 Hz, 3H, C1b–*H*) ppm. ^31^P­{^1^H} NMR (162 MHz, CDCl_3_) δ 33.68 (s, *P*Ar_3_), – 144.38 (hept, ^1^ *J*_Pz,F_ = 711.2 Hz) ppm. See the SI for ^13^C­{^1^H} and ^19^F data. Elemental analysis: calculated for C_35_H_34_ClF_6_NP_2_Ru•0.25CH_2_Cl_2_ (%): C 52.77, H 4.33, N 1.75; found: C 52.91, H 4.59, N 2.04.

### Ru^H^-NCAr^Cl^

Synthesized via the above procedure using 0.0563 g **Ru** ^**H**^ **-Cl** (0.0990 mmol), 0.0681 g 4-chlorobenzonitrile (0.495 mmol), and 0.0250 g AgPF_6_ (0.0990 mmol). Yellow powder; yield 0.0731 g (90.5%). ^1^H NMR (500 MHz, CDCl_3_): δ 7.59 (m, 6H, C9–*H*), 7.45 (m, 9H, C10–*H* and C11–*H*), 7.35 (d, ^3^ *J*_H,H_ = 8.7 Hz, 2H, C15–*H*), 7.31 (d, ^3^ *J*_H,H_ = 8.7 Hz, 2H, C14–*H*), 6.41 (dd, ^3^ *J*_H,H_ = 6.3 Hz, ^4^ *J*_H,H_ = 1.5 Hz, 1H, C4b–*H*), 6.03 (d, ^3^ *J*_H,H_ = 6.1 Hz, 1H, C4a–*H*), 5.46 (dd, ^3^ *J*_H,H_ = 6.2 Hz, ^4^ *J*_H,H_ = 1.4 Hz, 1H, C5a–*H*), 4.76 (d, ^3^ *J*_H,H_ = 6.3 Hz, 1H, C5b–*H*), 3.07 (hept, ^3^ *J*_H,H_ = 6.9 Hz, 1H, C2–*H*), 1.73 (s, 3H, C7–*H*), 1.39 (d, ^3^ *J*_H,H_ = 6.9 Hz, 3H, C1a–*H*), 1.37 (d, ^3^ *J*_H,H_ = 6.9 Hz, 3H, C1b–*H*) ppm. ^31^P­{^1^H} NMR (202 MHz, CDCl_3_) δ 35.88 (s, *P*Ar_3_), – 144.08 (hept, ^1^ *J*_P,F_ = 713.0 Hz) ppm. See the SI for ^13^C­{^1^H} and ^19^F data. Elemental analysis: calculated for C_35_H_33_Cl_2_F_6_NP_2_Ru (%): C 51.55, H 4.08, N 1.72; found: C 51.29, H 4.10, N 1.71.

### Ru^H^-NCAr^CF3^

Synthesized via the above procedure using 0.0612 g **Ru** ^**H**^ **-Cl** (0.108 mmol), 0.0921 g 4-(trifluoromethyl)­benzonitrile (0.538 mmol), and 0.0272 g AgPF_6_ (0.108 mmol). Yellow powder; yield 0.0809 g (88.5%). ^1^H NMR (500 MHz, CDCl_3_): δ 7.63 (d, ^3^ *J*_H,H_ = 8.6 Hz, 2H, C15–*H*), 7.60 (m, 6H, C9–*H*), 7.51 (d, ^3^ *J*_H,H_ = 8.2 Hz, 2H, C14–*H*), 7.45 (m, 9H, C10–*H* and C11–*H*), 6.44 (dd, ^3^ *J*_H,H_ = 6.3 Hz, ^4^ *J*_H,H_ = 1.5 Hz, 1H, C4b–*H*), 6.07 (d, ^3^ *J*_H,H_ = 6.1 Hz, 1H, C4a–*H*), 5.49 (dd, ^3^ *J*_H,H_ = 6.2 Hz, ^4^ *J*_H,H_ = 1.4 Hz, 1H, C5a–*H*), 4.77 (d, ^3^ *J*_H,H_ = 6.3 Hz, 1H, C5b–*H*), 3.09 (hept, ^3^ *J*_H,H_ = 6.9 Hz, 1H, C2–*H*), 1.73 (d, ^3^ *J*_H,P_ = 1.5 Hz, 3H, C7–*H*), 1.39 (d, ^3^ *J*_H,H_ = 6.8 Hz, 3H, C1a–*H*), 1.38 (d, ^3^ *J*_H,H_ = 6.8 Hz, 3H, C1b–*H*) ppm. ^31^P­{^1^H} NMR (202 MHz, CDCl_3_) δ 35.97 (s, *P*Ar_3_), – 144.07 (hept, ^1^ *J*_P,F_ = 713.0 Hz) ppm. See the SI for ^13^C­{^1^H} and ^19^F data. Elemental analysis: calculated for C_36_H_33_ClF_9_NP_2_Ru (%): C 50.92, H 3.92, N 1.65; found: C 51.06, H 3.87, N 1.71.

### Synthesis of *trans-mer*-C^H^-NCCH_3_

Under N_2_, (PPh_3_)_3_RuCl_2_ (0.2337 g, 0.2437 mmol, 1 equiv) was suspended in dry CH_3_CN, and the suspension was stirred. After 10 min, the solid had dissolved to give a yellow solution. KPF_6_ (0.0449 g, 0.244 mmol, 1 equiv) was added under N_2_ and the solution was heated to reflux. Over time, the color changed to light green, and a colorless precipitate formed. After 5 h, the solution was cooled and filtered through Celite. The volume of the filtrate was reduced to 1 mL, at which point a light-colored solid began to form, and the solution placed in a freezer to induce further precipitation. After 24 h, the solution was quickly filtered through a fritted glass funnel, and the resulting solid was washed with cold CH_3_CN and then with hexanes and air-dried. Light green-yellow powder; yield 0.0812 g (35.9%). An additional crop of product can be obtained by removing the solvent from the filtrate of the final filtration *in vacuo*, redissolving the resulting solid in minimal CH_3_CN, cooling to – 10 °C for several hours, then filtering the solution while cold. Total yield 0.111 g, (49.1%). Additional product remains in the soluble fraction after sequential precipitations, but its high solubility in CH_3_CN and the presence of other impurities typically prevent the isolation of the title compound in higher yields. ^1^H NMR (500 MHz, CD_3_CN): δ 7.82 (m, 12H, C2–*H*), 7.46 (m, 18H, C3–*H* and C4–*H*), 1.86 (t, ^5^ *J*_H,P_ = 1.1 Hz, 6H, C6–*H*), 1.31 (t, ^5^ *J*_H,P_ = 0.8 Hz, 3H, C8–*H*) ppm. ^31^P­{^1^H} NMR (202 MHz, CD_3_CN) δ 27.88 (s, *P*Ph_3_), – 145.49 (hept, ^1^ *J* = 706.7 Hz, *P*F_6_^–^) ppm. See the SI for ^13^C­{^1^H} and ^19^F data. Elemental analysis: calculated for C_42_H_39_ClF_6_N_3_P_3_Ru (%): C 54.29, H 4.23, N 4.52; found: C 54.32, H 4.18, N 4.59.

### Synthesis of *trans-mer*-C^H^-NCAr^OMe^

Under N_2_, (PPh_3_)_3_RuCl_2_ (0.2541 g, 0.2650 mmol, 1 equiv), 4-methoxybenzonitrile (0.7057 g, 5.300 mmol, 20 equiv), and AgBF_4_ (0.0516 g, 0.265 mmol, 1 equiv) were weighed out in a 100 mL Schlenk flask, and toluene (20 mL) was added. The solution was heated to 100 °C for 18 h, during which time the color changed from yellow-brown to light yellow and a fine colorless precipitate formed. The solution was filtered through Celite and the volume reduced to ∼2 mL *in vacuo*. Diethyl ether was added to precipitate a light-colored solid, which was collected via filtration on a fritted glass funnel. The solid was washed with ether (30 mL) to remove unreacted benzonitrile and give the product as a light tan powder (0.1089 g, 34.09% yield). ^1^H NMR (400 MHz, CDCl_3_): δ 7.86 (m, 12H, C2–*H*), 7.31 (m, 18H, C3–*H* and C4–*H*), 6.86 (s, 6 H, C7–*H* and C8–*H*), 6.83 (d, ^3^ *J*_H,H_ = 8.9 Hz, C13–*H*), 6.61 (d, ^3^ *J*_H,H_ = 8.9 Hz, C14–*H*), 3.85 (s, 6H, C10–*H*), 3.85 (s, 3H, C16–*H*) ppm. ^31^P­{^1^H} NMR (162 MHz, CDCl_3_) δ 27.51 (s, *P*Ph_3_) ppm. See the SI for ^13^C­{^1^H} and ^19^F data. Elemental analysis: calculated for C_60_H_51_BClF_4_N_3_O_3_P_2_Ru (%): C 62.81, H 4.48, N 3.66; found: C 62.83, H 4.46, N 3.51.

## Results and Discussion

### Synthesis and Characterization of Bis­(phosphine) and Solvento Complexes

In order to test our hypothesis that the number of phosphine ligands in the byproducts of electrochemical oxidation can be identified from the effects of the X substituents on their reduction potential, we sought to investigate the electrochemical properties of bis­(phosphine) analogues (**Ru** ^**X**^ **-PAr**_**3**_) of the previously studied **Ru** ^**X**^ **-Cl** complexes. While triphenylphosphine complex **Ru** ^**H**^ **-PAr**_**3**_ had previously been characterized,^8,64^ few other compounds of this type supported by substituted triarylphosphine ligands had been reported. Therefore, the synthesis of **Ru** ^**X**^ **-PAr**_**3**_ complexes bearing electron-donating and -withdrawing substituents, respectively, was targeted. **Ru** ^**OMe**^ **-Cl** and **Ru** ^**H**^ **-Cl** were initially converted to the corresponding acetonitrile solvento complexes **Ru** ^**OMe**^ **-NCCH**_**3**_ and **Ru** ^**H**^ **-NCCH**_**3**_ via treatment with AgPF_6_ in CH_3_CN (Scheme 2a). Subsequent heating of these complexes in CHCl_3_ in the presence of excess free phosphine resulted in substitution of CH_3_CN by a second phosphine ligand, providing **Ru** ^**OMe**^ **-PAr**_**3**_ and **Ru** ^**H**^ **-PAr**_**3**_ as yellow powders in acceptable yields. While solvento complex **Ru** ^**Cl**^ **-NCCH**_**3**_ could be accessed via a similar procedure, exchange of CH_3_CN for a second equivalent of PAr^Cl^_3_ proved challenging, likely due the electron-withdrawing Cl substituents lowering the donating ability of this ligand. Thus, although pure samples of **Ru** ^**Cl**^ **-PAr**_**3**_ could occasionally be prepared via this procedure, the poor yields and low reproducibility led us to investigate alternative synthetic protocols. We found that *in situ* generation of the putative acetone solvento complex, followed by immediate treatment with excess PAr^Cl^_3_ ligand, provided **Ru** ^**Cl**^ **-PAr**_**3**_ in good yield and without the need for extensive purification (Scheme 2a).

**Scheme 2:**
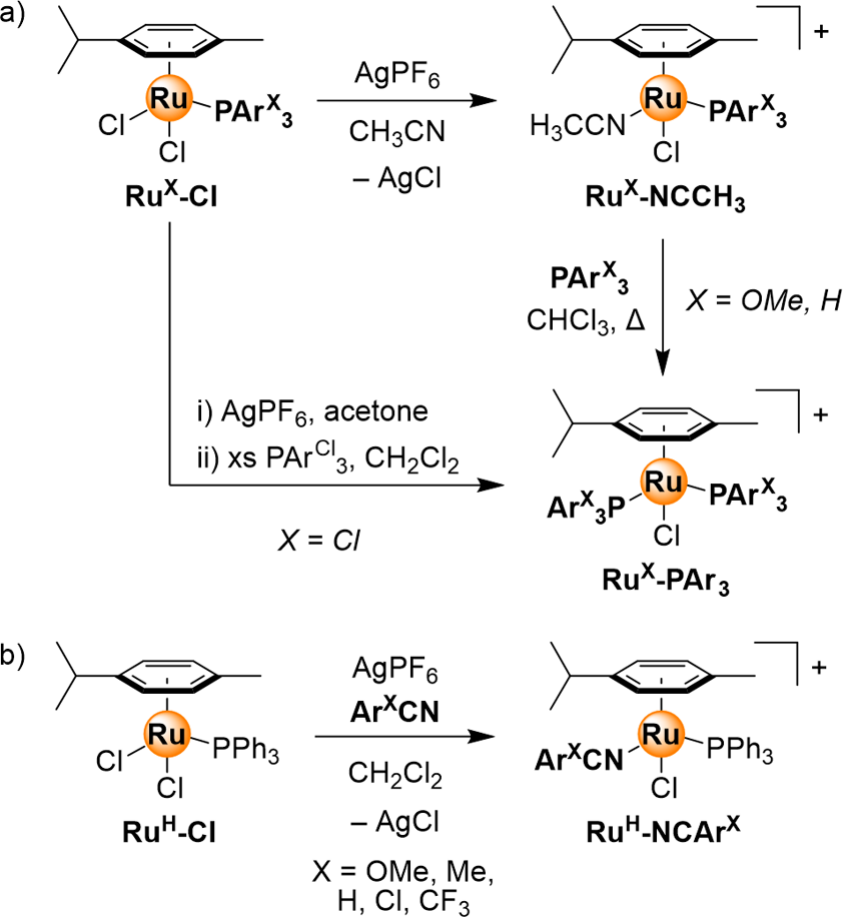
Synthesis of Ru^X^-L Complexes

The two series of products were identified by ^1^H and ^31^P­{^1^H} nuclear magnetic resonance (NMR). Dichloride complexes **Ru** ^**X**^ **-Cl** possess *C*_*s*_ symmetry in solution, and the aromatic protons on their cymene ligand therefore give rise to two signals in the ^1^H NMR spectra for these compounds (Figure 3, upper trace). Exchange of one Cl^–^ ligand for acetonitrile results in lowering of the symmetry to *C*_1_, making all four cymene protons inequivalent in complexes **Ru** ^**X**^ **-NCCH**_**3**_ (Figure 3, middle trace). Replacement of the CH_3_CN ligand with a second, identical phosphine ligand restores the *C*_s_ symmetry, leading to the collapse of the four signals in the cymene region to two resonances in **Ru** ^**X**^ **-PAr**_**3**_ complexes (Figure 3, lower trace). The changes in coordination at Ru also have a significant effect on the environment of the bound phosphorus atom as determined via ^31^P­{^1^H} NMR. In solvento complexes **Ru** ^**X**^ **-NCCH**_**3**_, the resonance for the P atom in the PAr^X^_3_ ligands is shifted ∼10 ppm downfield with respect to dichloride complexes **Ru** ^**X**^ **-Cl**; introduction of a second phosphine reverses this shift, with the ^31^P resonances in bis­(phosphine) complexes **Ru** ^**X**^ **-PAr**_**3**_ appearing slightly upfield of those for both solvento compounds **Ru** ^**X**^ **-NCCH**_**3**_ and their dichloride precursors **Ru** ^**X**^ **-Cl**. A correlation was observed between the ^31^P chemical shifts for **Ru** ^**X**^ **-PAr**_**3**_ complexes and the Hammett parameter σ_p_,^65^ while neither dichloride nor solvento complexes displayed a similar relationship (Table S1, Figure S97). The stability of the **Ru^H^-NCCH_3_** and **Ru^H^-PAr_3_** complexes was evaluated in both coordinating (CDCl_3_) and noncoordinating (CD_3_CN) solvents under various conditions (see SI).

**Figure 3:**
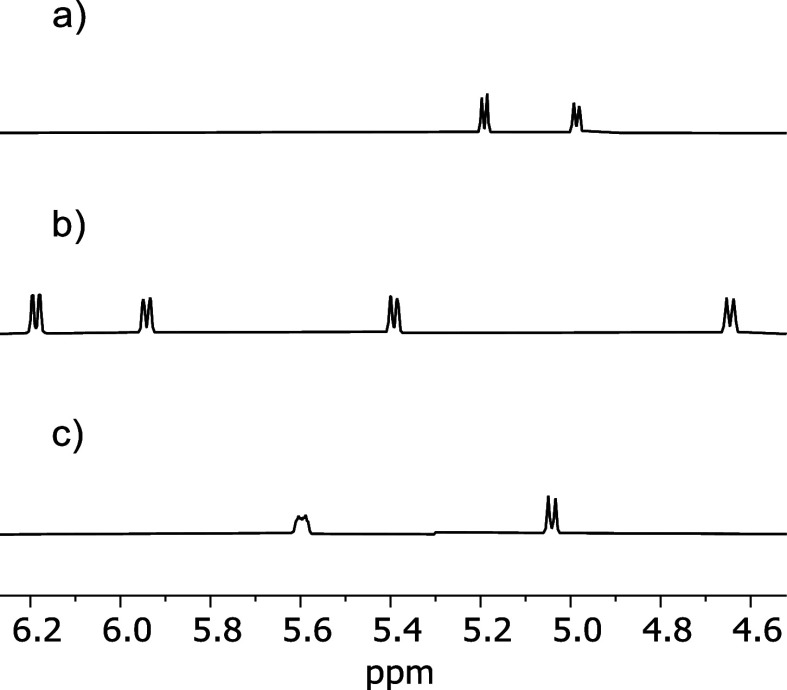
Partial ^1^H NMR spectra (400 MHz, CDCl_3_) for (a) **Ru** ^H^ **-Cl**, (b) **Ru** ^H^ **-NCCH**_3_, and (c) **Ru** ^H^ **-PAr**_3_, displaying the resonances corresponding to the *p*-cymene hydrogens and highlighting the differences in symmetry across the three series of compounds.

Single-crystal X-ray diffraction (XRD) data for **Ru** ^**OMe**^ **-PAr**_**3**_ revealed the expected piano-stool structure, with a *p*-cymene ligand bound in an η^6^ fashion to a pseudo-octahedral Ru center (Figure 4). Two PAr^OMe^_3_ ligands and a chloride complete the Ru coordination sphere. The Ru–P and Ru–Cl bond distances correlate with those of other compounds in this family whose structures have been reported, such as solvento complex **Ru** ^**OMe**^ **-NCCH**_**3**_ and bis­(phosphine) compound **Ru** ^**H**^ **-PAr**_**3**_ (Table 1).^44,66^ Introduction of the second bulky phosphine ligand in **Ru** ^**OMe**^ **-PAr**_**3**_ causes a substantial expansion of the angle between the nonchloride ligands (98.81(4)° for the P–Ru–P angle, vs 85.89(13)° for the corresponding P–Ru–N angle in **Ru** ^**OMe**^ **-NCCH**_**3**_), similar to what is observed in the structure of **Ru** ^**H**^ **-PAr**_**3**_. Likewise, a slight elongation in the Ru–cymene distances in the bis­(phosphine) complexes (measured as the distance between Ru and the calculated centroid of the cymene ligand) may be ascribed to the steric demands of the two phosphine ligands. Aryl groups on the two different phosphine ligands (C32–C37, bound to P2, and C25–C30, bound to P1; the latter fragment is disordered over two positions, labeled A and B, respectively) are nearly parallel, displaying a possible π-π interaction (interplane angle: 13.648° (A orientation), 3.390° (B orientation); centroid–centroid distance = 3.755 Å (A), 3.427 Å (B); plane shift distance = 1.518 Å (A), 1.158 Å (B); see SI for further details).^67^

**Figure 4:**
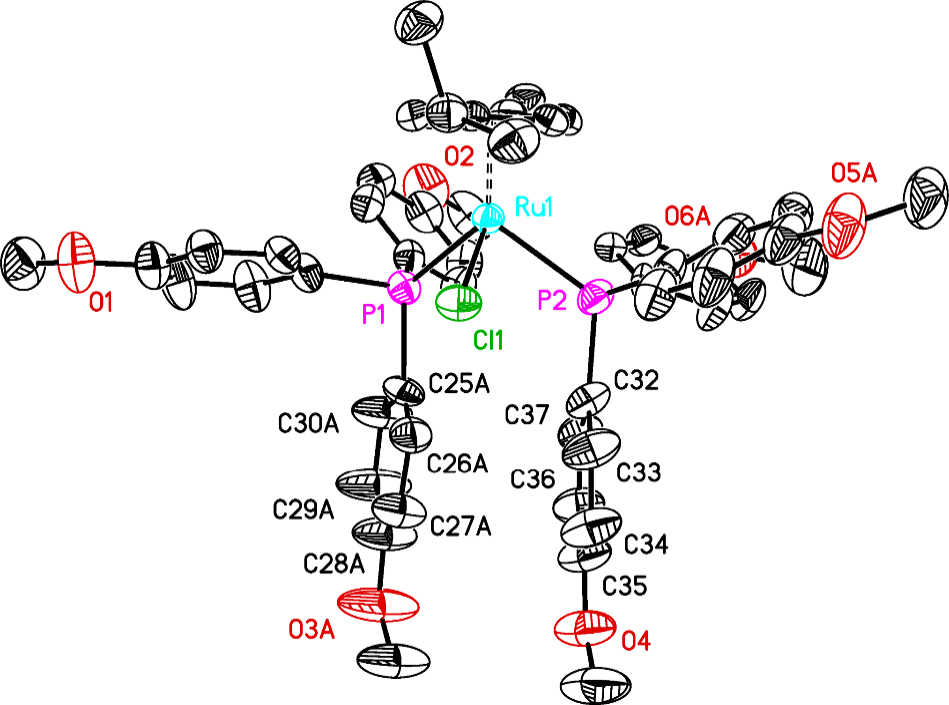
Solid-state structure (XRD) of complex **Ru** ^OMe^ **-PAr**_3_. Diffraction data collected at 100 K. Hydrogen atoms and outer-sphere PF_6_^–^ counteranion not shown for clarity; only the major orientations (50–75%) of the disordered aryl rings shown for clarity. Displacement ellipsoids shown at the 50% probability level.

**Table 1:** Selected Bond and Angle Metrics for Complex Ru^OMe^-PAr_3_ and Comparison Compounds

Ru^X^-L	Ru^OMe^-NCCH_3_	Ru^OMe^-PAr_3_	Ru^H^-PAr_3_
CCDC #	1894829	2532847	212964

	bond lengths (Å)		
Ru–(cymene)^a^	1.7259(19)	1.7784(15)	1.739
Avg. Ru–C^b^	2.230 ± 0.023	2.271 ± 0.037	2.278 ± 0.040
Ru–Cl	2.3880(12)	2.3851(11)	2.3911(7)
Ru–P	2.3684(13)	2.3901(11)	2.3649(6)
Ru–L^c^	2.058(4)	2.3705(11)	2.4042(6)

	bond angles (°)		
Cl–Ru–P	84.44(4)	89.05(4)	87.61(2)
Cl–Ru–L^b^	86.74(13)	83.61(4)	90.13(2)
P–Ru–L^b^	85.89(13)	98.81(4)	97.97(2)
reference	^44^	this work	^66^

a Distance between Ru and the calculated centroid of the cymene ligand.

b Average distance between Ru and cymene C atoms.

c **Ru** ^**OMe**^ **-NCCH**_**3**_, L = NCCH_3_; **Ru** ^**OMe**^ **-PAr**_**3**_, L = PAr^OMe^_3_; **Ru** ^**H**^ **-PAr**_**3**_, L = PPh_3_.

### Electrochemical oxidation and Hammett analyses

In CH_2_Cl_2_, solvento complexes **Ru** ^**X**^ **-NCCH**_**3**_ display *quasi*reversible 1e^–^ events at potentials between +1.2 and +1.4 V vs the ferrocenium/ferrocene couple (denoted hereafter as Fc^+/0^), consistent with oxidation of Ru­(II) to Ru­(III) (Figure S105). In these systems, Ru oxidation occurs at potentials >0.5 V more positive than in the case of our previously investigated **Ru** ^**X**^ **-Cl** complexes,^40^ likely due to the cationic nature of the solvento compounds (vs the neutral dichlorides). In CH_3_CN, **Ru** ^**X**^ **-NCCH**_**3**_ complexes likewise undergo a 1e^–^ oxidation at potentials ∼0.5 V more positive than their **Ru** ^**Cl**^ **-NCCH**_**3**_ counterparts (Figure 5, left panel; Table 2, *E*_pa_(**[Ru** ^**X**^ **-NCCH**_**3**_ **]** ^**2+/+**^), though these events are completely irreversible. Concurrent appearance of new *quasi*reversible events at a potential ∼0.35 V more negative than the initial Ru­(III/II) couple indicates that oxidation to the Ru­(III) state results in rapid formation of a new series of electroactive byproducts via an analogous EC mechanism^46^ to that outlined for complexes **Ru** ^**X**^ **-Cl.**

**Figure 5:**
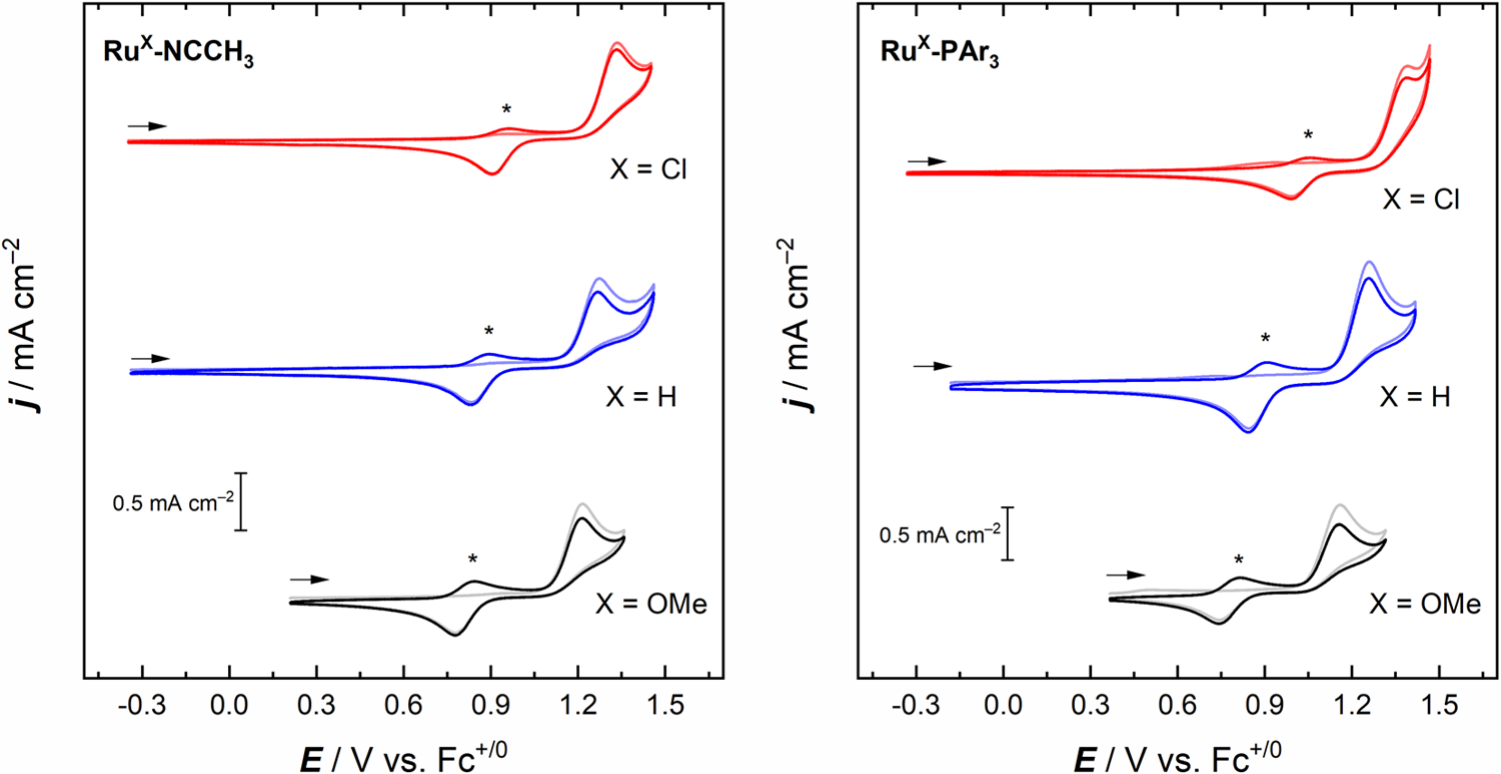
CV data (0.1 *M* [^n^Bu_4_N]­[PF_6_] in CH_3_CN, 100 mV/s; [Ru] = 3 m*M*) for complexes **Ru** ^X^ **-NCCH**_3_ (left panel) and **Ru** ^X^ **-PAr**_3_ (right panel). The first two voltammetric scans are shown, with the first scan being lighter in color. Events corresponding to **B** ^X^ **-NCCH**_3_ (left panel) and **C** ^X^ **-NCCH**_3_ species (right panel) are marked with (*).

**Table 2:** Reduction Potentials^a^ for Complexes Ru^X^-L and for Their Byproducts in CH_3_CN

	X = OMe	X = H	X = Cl
σ_p_(X)^b^	–0.27	0	0.23
*E*°′([**Ru** ^**X**^ **-Cl**]^+/0^)	+0.65	+0.70	+0.76
*E*°′([**A** ^**X**^ **-NCCH**_**3**_ **]** ^+/0^)	+0.17	+0.21	+0.27
*E*_pa_([**Ru** ^**X**^ **-NCCH**_**3**_]^2+/+^)	+1.22	+1.27	+1.33
*E*°′([**B** ^**X**^ **-NCCH**_**3**_]^2+/+^)	+0.81	+0.86	+0.93
*E*_pa_([**Ru** ^**X**^ **-PAr**_**3**_]^2+/+^)	+1.16	+1.26	+1.39
*E*°′([**C** ^**X**^ **-NCCH**_**3**_ **]** ^2+/+^)	+1.79	+0.88	+1.02

a In V vs Fc^+/0^.

b From ref 65.

Based on our earlier work, these observations suggest displacement of *p*-cymene by three CH_3_CN ligands to yield, in this case, tetrakis­(nitrile) complexes **[B** ^**X**^ **-NCCH**_**3**_ **]** ^**+**^ (Scheme 3). The shift in potential for the oxidation byproducts is consistent with the replacement of the more π-acidic *p*-cymene ligand for the weaker π-accepting acetonitrile moieties. Following the initial oxidation step (E), the chemical step (C) in this EC mechanism appears to be significantly faster than in the case of dichloride complexes **Ru** ^**X**^ **-Cl**, as no cathodic return peak is observed for the initial oxidation of complexes **Ru** ^**X**^ **-NCCH**_**3**_ even at fast scan rates (2,000 mV/s; Figure S107). As the reaction of the nascent 17e^–^ Ru­(III) complexes with exogenous CH_3_CN is expected to proceed via an associative mechanism,^68–71^ the greater positive charge at Ru in the cationic **[Ru** ^**X**^ **-NCCH**_**3**_ **]** ^**+**^ complexes likely results in faster initial binding of CH_3_CN, leading to complete conversion of the Ru­(III) complex generated at the electrode surface on the CV time scale.

**Scheme 3:**
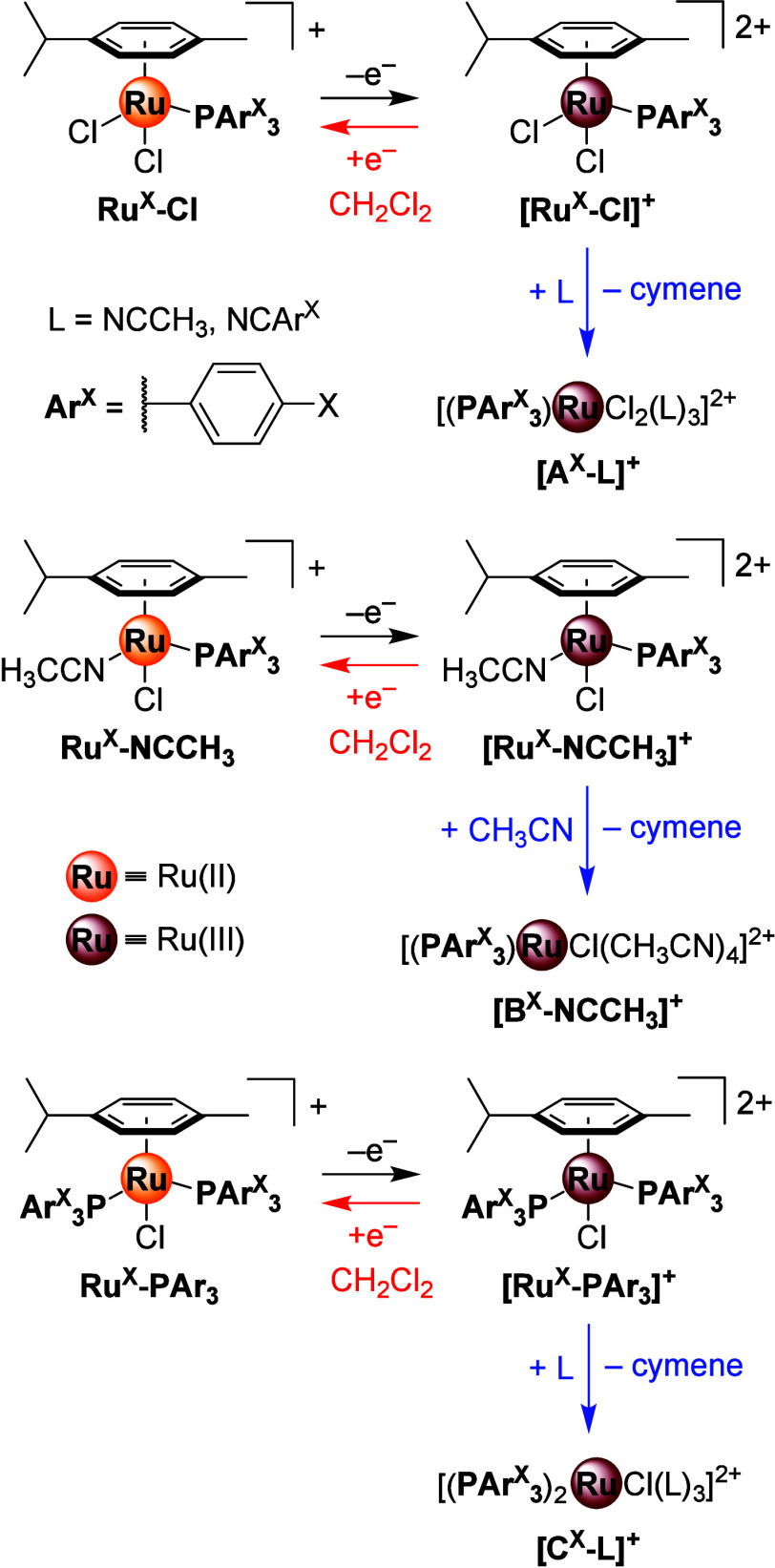
Proposed ET and Chemical Reactivity of Ru^X^-L Complexes

Similarly to solvento compounds, bis­(phosphine) complexes **Ru** ^**X**^ **-PAr**_**3**_ display 1e^–^ oxidations that are shifted positively by ∼ 0.5–0.7 V with respect to those of dichloride complexes **Ru** ^**X**^ **-Cl**, consistent with their cationic charge (Figure 5, right panel). Indeed, the potentials for the Ru­(III/II) oxidation in complexes **Ru** ^**H**^ **-NCCH**_**3**_ and **Ru** ^**H**^ **-PAr**_**3**_ are almost identical (+1.27 vs +1.26 V vs Fc^+/0^, respectively), suggesting that effects of replacing CH_3_CN (a poor σ donor and poor π acceptor ligand) with PPh_3_ (a stronger σ donor but also a stronger π acceptor) coincidentally offset in this case. In CH_2_Cl_2_, **Ru** ^**H**^ **-PAr**_**3**_ and **Ru** ^**Cl**^ **-PAr**_**3**_ display *quasi*reversible 1e^–^ events at +1.33 and +1.46 V vs Fc^+/0^, respectively (Figure S106, Table S3). Surprisingly, oxidation of **Ru** ^**OMe**^ **-PAr**_**3**_ is irreversible under analogous conditions (*E*_pa_ = +1.32 V vs Fc^+/0^); subsequent voltammetric scans reveal fouling of the electrode surface, indicating possible deposition of decomposition products. In CH_3_CN, oxidation of **Ru** ^**X**^ **-PAr**_**3**_ complexes is irreversible (Figure 5, right panel; Table 2), and a new *quasi*reversible couple is observed in each case at a potential ∼0.35 V more negative than the initial oxidation. These observations are consistent with analogous reactivity to that described for complexes **Ru** ^**X**^ **-Cl** and **Ru** ^**X**^ **-NCCH**_**3**_–oxidation to Ru­(III) leads to displacement of *p*-cymene by CH_3_CN, resulting in formation of byproducts with the general formula [RuCl­(PAr^X^_3_)_2_(NCCH_3_)_3_]^+^ (**[C** ^**X**^ **-NCCH**_**3**_ **]** ^**+**^. Scheme 3). As in the case of solvento complexes **Ru** ^**X**^ **-NCCH**_**3**_, virtually no cathodic return current is observed following oxidation of compounds **Ru** ^**X**^ **-PAr**_**3**_ even at faster scan rates (2000 mV/s, Figure S108), consistent with faster reactivity following ET, likely due to the cationic charge of these complexes.

Hammett analyses were carried out to provide insight into the fate of the solvento and bis­(phosphine) complexes following oxidation to Ru­(III). Plots of the anodic peak potentials for both **Ru** ^**X**^ **-NCCH**_**3**_ and **Ru** ^**X**^ **-PAr**_**3**_ complexes vs the *para* Hammett parameter (σ_p_) for the X substituents^65^ reveal linear correlations for each series of compounds (Figure 6, left panel). The slope of the correlation observed for **Ru** ^**X**^ **-NCCH**_**3**_ complexes, which contain a single PAr^X^_3_ ligand, is close to that previously determined for dichloride complexes **Ru** ^**X**^ **-Cl**, which are also monophosphine species (0.23 and 0.24 V, respectively). On the other hand, bis­(phosphine) complexes **Ru** ^**X**^ **-NCCH**_**3**_ display a stronger dependence on σ_p_, with a slope close to twice that for the two sets of monophosphine complexes (0.46 V). This is consistent with the additivity of substituent effects: the presence of a larger number of substituents on the bis­(phosphine) complexes (6 vs 3 for monophosphine compounds) results in a greater influence on the potential for ET at Ru.

**Figure 6:**
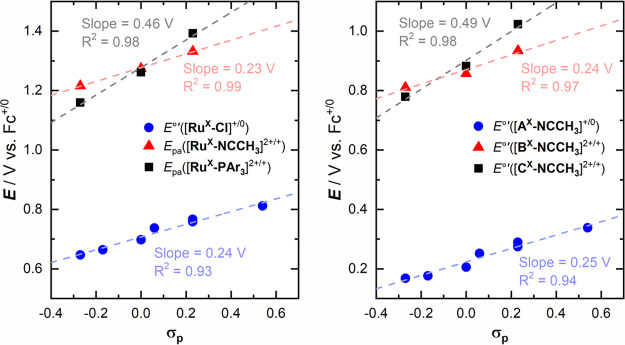
Plots of *E* vs σ_p_ for complexes **Ru** ^X^ **-Cl**, **Ru** ^X^ **-NCCH**_3_, and **Ru** ^X^ **-PAr**_3_ (left panel) and for their byproducts **A** ^X^ **-NCCH**_3_, **B** ^X^ **-NCCH**_3_, and **C** ^X^ **-NCCH**_3_ (right panel) based on CV data in CH_3_CN. The slope and R^2^ value for the lines of best fit for each series are included. The vertical axes for both plots are shown at the same scale.

To confirm our earlier hypothesis on the similarity of the electronic communication between peripheral substituents and Ru in both mono- and bis­(phosphine) complexes, the Hammett reaction constant (ρ) was calculated for each series of complexes. Values of ρ can be calculated by plotting the *shift* in potential (Δ*E*) for each complex bearing a substituent (*E*_X_) with respect to the unsubstituted complex (*E*_H_; in our work, this corresponds to the reduction potential of **Ru** ^**H**^ **-Cl**, **Ru** ^**H**^ **-NCCH**_**3**_, or **Ru** ^**H**^ **-PAr**_**3**_) as a function of the sum of Hammett parameters for all substituents present (Σσ_p_), according to the following equation:^47,48^

$$\Delta E=E_{X}-E_{H}=\left(0.0592V\right)\rho \left({\Sigma \sigma }_{p}\right)$$ 1

Notably, this approach is predicated on the additivity of substituent effects, which has been well-established in numerous organic and inorganic systems.^47–52,72^ In this context, the value of ρ has been used to quantify the extent of electronic communication between the substituents and the site of ET.^49,50^ Given that all complexes considered here (**Ru** ^**X**^ **-Cl**, **Ru** ^**X**^ **-NCCH**_**3**_, and **Ru** ^**X**^ **-PAr**_**3**_) share the same ligand substitution pattern, it is expected that ρ would remain essentially unchanged across the three series of complexes, as it is unlikely that substantial differences in electronic communication would arise in systems containing only one or two substituted ligand platforms. Δ*E* vs Σσ_p_ plots for **Ru** ^**X**^ **-NCCH**_**3**_, and **Ru** ^**X**^ **-PAr**_**3**_ gave ρ values of 1.3 (Figure 7), similar to that obtained earlier from analysis of dichloride complexes **Ru** ^**X**^ **-Cl** (ρ = 1.2; Table 3). This observation supports the additivity of substituent effects in these systems.

**Figure 7:**
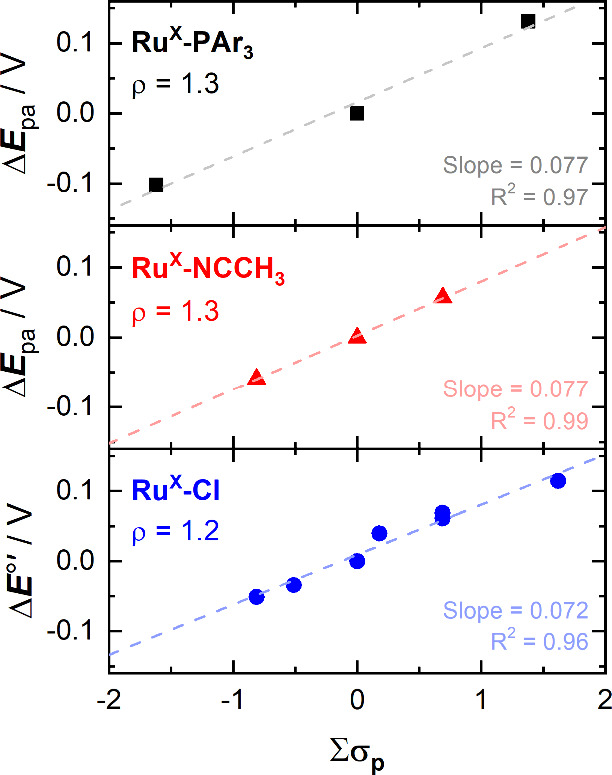
Hammett plots for complexes **Ru** ^X^ **-Cl** (lower panel, blue circles), **Ru** ^X^ **-NCCH**_3_ (middle panel, red triangles), and **Ru** ^X^ **-PAr**_3_ (upper panel, black squares). The slope and R^2^ value for the lines of best fit and the calculated value of ρ (from eq 1) for each series are included. The vertical axes for all plots are shown at the same scale.

**Table 3:** Hammett Reaction Constants^a^ (ρ) for Complexes in the Present Study

complex	Σσ model	ρ
**Ru** ^**X**^ **-Cl**	3σ^b^	1.2
**Ru** ^**X**^ **-NCCH**_**3**_	3σ^b^	1.3
**Ru** ^**X**^ **-PAr**_**3**_	6σ^b^	1.3
**A** ^**X**^ **-NCCH**_**3**_	3σ	1.3
	6σ	0.64
**B** ^**X**^ **-NCCH**_**3**_	3σ	1.4
	6σ	0.69
**C** ^**X**^ **-NCCH**_**3**_	3σ	2.7
	6σ	1.4
**Ru** ^**H**^ **-NCAr** ^**X**^	1σ^b^	1.4
**A** ^**X**^ **-NCAr** ^**X**^ ^c^	1σ	3.3
	2σ	1.6
	3σ	1.1
**C** ^**H**^ **-NCAr** ^**X**^ ^d^	1σ	3.5
	2σ	1.6
	3σ	1.1

a Calculated from eq 1.

b Ligand stoichiometry known from spectroscopic/structural characterization.

c Generated electrochemically from addition of Ar^X^CN to **Ru** ^**H**^ **-Cl**.

d Generated electrochemically from addition of Ar^X^CN to **Ru** ^**H**^ **-PAr** **_3_**.

The reduction potentials for complexes **B** ^**X**^ **-NCCH**_**3**_ and **C** ^**X**^ **-NCCH**_**3**_ correlate linearly with σ_p_ in a similar fashion to those for the **A** ^**X**^ **-NCCH**_**3**_ products. The correlation observed for the **B** ^**X**^ **-NCCH**_**3**_ complexes (slope = 0.24 V; Figure 6, right panel) formed from solvento complexes **Ru** ^**X**^ **-NCCH**_**3**_ (slope = 0.23 V) is similar to those for monophosphine compounds **Ru** ^**X**^ **-Cl** (slope = 0.24 V) and the respective byproducts **A** ^**X**^ **-NCCH**_**3**_ (slope = 0.25 V). On the other hand, products **C** ^**X**^ **-NCCH**_**3**_ display a steeper correlation with σ_p_ (slope = 0.49 V), more similar to their precursors (**Ru** ^**X**^ **-PAr**_**3**_, slope = 0.46 V) than to any monophosphine complexes. Hammett analysis of **A** ^**X**^ **-NCCH**_**3**_, **B** ^**X**^ **-NCCH**_**3**_, and **C** ^**X**^ **-NCCH**_**3**_ based on different values of Σσ_p_ (3σ_p_ for monophosphines, 6σ_p_ for bis­(phosphines)) enabled calculation of possible values of ρ for different ligand stoichiometries (Figure 8; Table 3). For these electrochemical byproducts, only modeling of **A** ^**X**^ **-NCCH**_**3**_ and **B** ^**X**^ **-NCCH**_**3**_ as monophosphine species (Figure 8, left panel) and of **C** ^**X**^ **-NCCH**_**3**_ compounds as bis­(phosphine) complexes (Figure 8, right panel) provide values of ρ in agreement with those determined for complexes **Ru** ^**X**^ **-Cl**, **Ru** ^**X**^ **-NCCH**_**3**_, and **Ru** ^**X**^ **-PAr**_**3**_ (ρ = 1.2–1.4). Our observations on the structurally well-defined complexes are consistent with the hypothesis that substituent effects are fully additive within this family and that Hammett analyses can provide insight into the number of Ru-bound substituent-bearing ligands. Modeling of the ligand stoichiometry in complexes **A** ^**X**^-**C** ^**X**^ in Hammett analyses furthermore supports the conclusion that the chemical reactivity that follows electrochemical oxidation does *not* involve changes in the number of phosphine ligands. Therefore, the changes undergone by these complexes upon oxidation must only involve other ligand groups.

**Figure 8:**
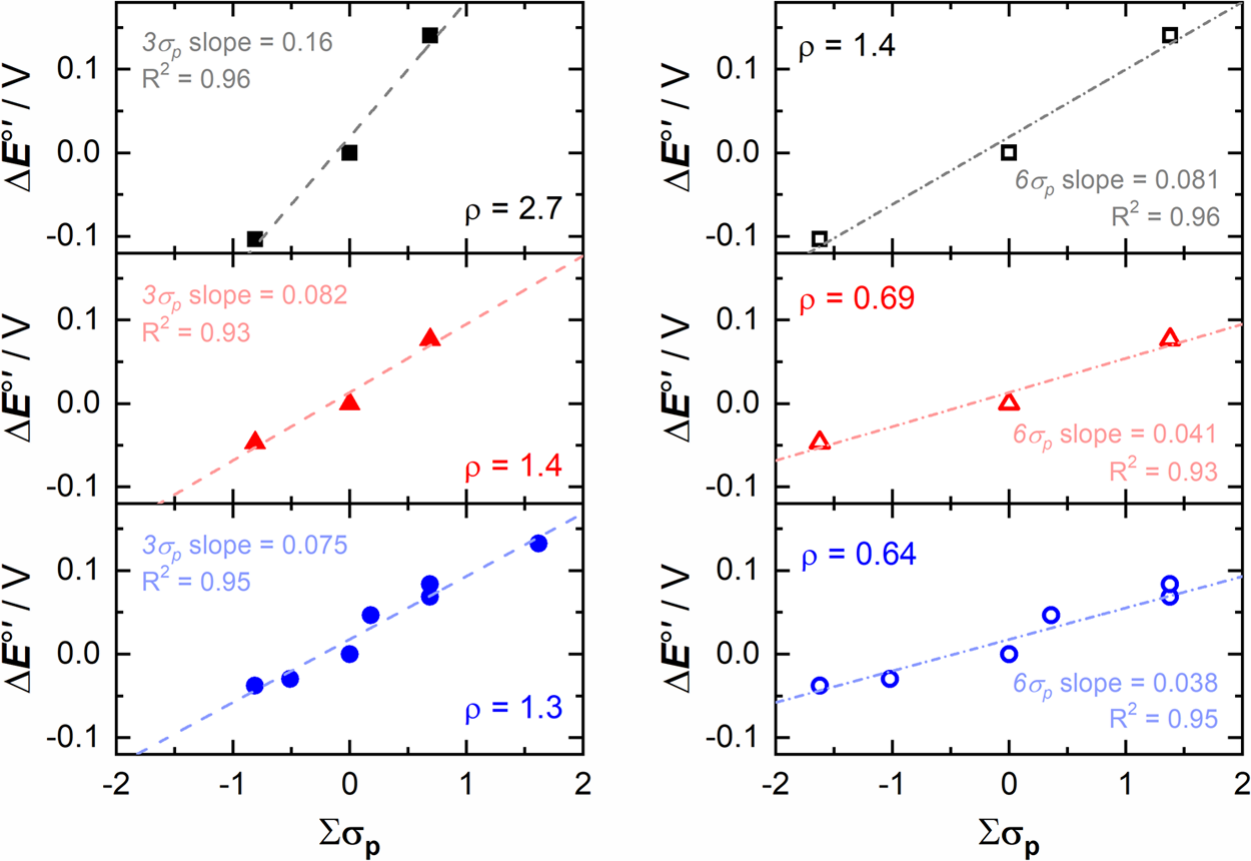
Hammett plots for complexes **A** ^X^ **-NCCH**_3_ (blue circles), **B** ^X^ **-NCCH**_3_ (red triangles), and **C** ^X^ **-NCCH**_3_ (black squares) modeled as monophosphine (left panel, filled markers) and bisphosphine (right panel, hollow markers). The slopes and R^2^ values for the lines of best fit and the calculated values of ρ (from eq 1) for each series are included.

The proposed assignment of **A** ^**X**^ **-NCCH**_**3**_ complexes as dichloride species (i.e., RuCl_2_(PAr^X^_3_)­(NCCH_3_)_3_) assumed that loss of Cl^–^ would result in generation of species with more *positive* reduction potentials due to the cationic nature of Ru­(II) complexes featuring a single chloride ligand. As the reduction potentials for complexes **A** ^**X**^ **-NCCH**_**3**_ are ∼ 0.35 V more *negative* than those of their precursors, Cl^–^ dissociation appeared unlikely. Additionally, it was not expected that the interaction between Ru and the anionic chloride ligands would be weakened upon oxidation to the more positively charged Ru­(III) state.^43^ *E°′* values for the Ru­(III/II) couple in **Ru** ^**X**^ **-NCCH**_**3**_ and **B** ^**X**^ **-NCCH**_**3**_ complexes are shifted *positively* by about 0.5 V vs those of the corresponding dichloride and **A** ^**X**^ **-NCCH**_**3**_ compounds, respectively, supporting retention of the Cl^–^ ligands in the reaction following electrochemical oxidation. The more negative potential of **A** ^**X**^ **/B** ^**X**^ **/C** ^**X**^ complexes vs their precursors could instead be consistent with the *addition* of a Cl^–^ ligand, possibly abstracted from a second equivalent of compound in a disproportionation reaction. While monomeric trichlororuthenium complexes are known,^73–79^ the majority of such compounds contain multiple phosphine ligands,^76–79^ which are ruled out for **A** ^**X**^ **-NCCH**_**3**_ and **B** ^**X**^ **-NCCH**_**3**_ complexes based on Hammett analyses. Conversely, a negative shift in potential could indicate the formation of chloride-bridged, dimeric species containing more than two Cl^–^/Ru (e.g., [Ru_2_Cl_5_]), many of which have been reported in the literature.^80–85^ To probe the possibility of dimerization processes in the formation of complexes **A** ^**X**^ **-NCCH**_**3**_, CV data was collected for **Ru** ^**H**^ **-Cl** at various Ru concentrations (0.1–10 mM). The ratio between the cathodic peak currents for the reduction of **[Ru** ^**H**^ **-Cl]** ^**+**^ and **[A** ^**H**^ **-NCCH**_**3**_ **]** ^**+**^, a proxy for the extent of conversion of **[Ru** ^**H**^ **-Cl]** ^**+**^ to **[A** ^**H**^ **-NCCH**_**3**_ **]** ^**+**^, remained virtually constant across the concentrations investigated (∼0.17; Figure S113). Together with the known propensity for Cl-bridged dimeric structures to cleave to monomeric species in the presence of excesses of exogenous ligands (as in coordinating solvent),^10,43,86^ these results support a monomeric nature for the byproducts of electrochemical oxidation.

### Incorporation of Benzonitriles in ET Byproducts

Having obtained evidence for the retention of phosphine and chloride ligands during reactivity at Ru­(III), we sought to confirm that nitriles are incorporated as ligands following cymene dissociation. Addition of benzonitrile (Ar^H^CN, ∼ 3 *M*, ∼ 1,000 equiv) to a CH_2_Cl_2_ solution of **Ru** ^**H**^ **-Cl** resulted in loss of reversibility for the Ru­(III/II) couple and appearance of a new *quasi*reversible couple at *E°′* = +0.31 V vs Fc^+/0^ (Figure 9, left panel). The potential for this new couple is almost 100 mV positive of that for the byproduct generated in acetonitrile (+0.22 V). Electrochemical oxidation of **Ru** ^**H**^ **-Cl** in the presence of various *para*-substituted benzonitriles (Ar^X^CN = 4-X-benzonitrile; X = OMe, Me, H, Cl, CF_3_) generated byproducts whose potentials shifted as a function of the benzonitrile X substituent (Figure 9, right panel; Table 4). These observations indicate that the benzonitriles are incorporated in the resulting products (**A** ^**H**^ **-NCAr** ^**X**^, Scheme 3). 4-trifluoromethylbenzonitrile represented an exception to this trend, as no signal corresponding to the **A** ^**H**^ **-NCAr** ^**CF3**^ complex was observed even at a benzonitrile concentration of ∼ 6 *M* (∼2,000 equiv.; Figure S114). We hypothesize that the presence of the electron-withdrawing CF_3_ severely limits the donor ability of this compound and prevents its reaction with Ru­(III) on the electrochemical time scale.

**Figure 9:**
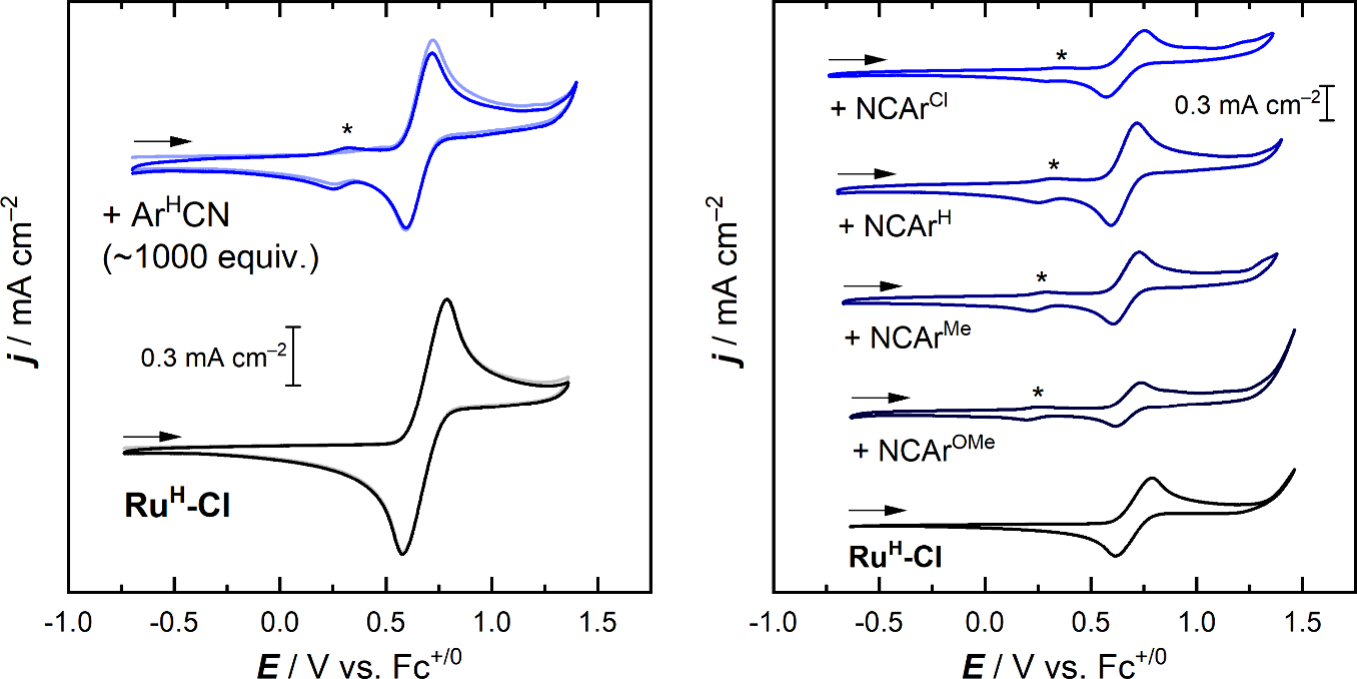
Left panel: CV data (0.1 *M* [^n^Bu_4_N]­[PF_6_] in CH_2_Cl_2_, 100 mV/s, [Ru] = 3 m*M*) for **Ru** ^H^ **-Cl** in CH_2_Cl_2_ in the absence (lower trace, black) and presence (upper trace, blue) of ∼3 M (∼1000 equiv) of benzonitrile (Ar^H^CN). Right panel: CV data (0.1 *M* [^n^Bu_4_N]­[PF_6_] in CH_2_Cl_2_, 100 mV/s, [Ru] = 3 mM) for complex **Ru** ^H^ **-Cl** in the presence of ∼ 3 M (∼1,000 equiv) of various *para*-substituted benzonitriles (NCAr^X^, X = OMe, Me, H, Cl). Only the second voltammetric scans are shown for clarity; events corresponding to **A** ^H^ **-NCAr** ^X^ species are marked with (*).

**Table 4:** Reduction Potentials^a^ for Complexes A^H^-NCAr^X^ and Ru^H^-NCAr^X^

X	σ_p_ ^b^	*E°′*([**A** ^**H**^ **-NCAr** ^**X**^]^+/0^)	*E°′*([**Ru** ^**H**^ **-NCAr** ^**X**^]^2+/+^)
OMe	–0.27	0.23	1.31
Me	–0.17	0.26	1.33
H	0	0.28	1.34
Cl	0.23	0.33	1.36
CF_3_	0.54	^c^	1.38

a In V vs Fc^+/0^.

b From ref 65.

c No byproduct observed at ∼6 *M* Ar^CF3^CN.

The reduction potentials for the **A** ^**H**^ **-NCAr** ^**X**^ products displayed a linear correlation with the σ_p_ parameter (slope = 0.19 V; Figure S115). However, literature reports have indicated that the effects on ET processes of ligand-based substituents located on differing frameworks are not directly comparable when these scaffolds are not isostructural, have different donor profiles, or display different substitution patterns.^49^ Thus, we expected that the substantial differences in connectivity between the X substituents and the Ru center in the putative **A** ^**H**^ **-NCAr** ^**X**^ complexes vs compounds **Ru** ^**X**^ **-L** both in terms of number of intervening atoms between X and Ru (5 in complexes **Ru** ^**H**^ **-L** vs 6 in **A** ^**H**^ **-NCAr** ^**X**^) and of the nature of this linkage (three substituted aryls interacting with Ru through the same P donor vs a single substituted aryl interacting with Ru through a nitrile moiety) would complicate direct comparisons between different substituted ligands. Preparation of a series of complexes incorporating substituted benzonitrile ligands in a known stoichiometry was therefore targeted.

### Synthesis and Voltammetric Studies of Monobenzonitrile Complexes **Ru^H^-NCAr^X^**

A series of monobenzonitrile complexes (**Ru** ^**H**^ **-NCAr** ^**X**^) was prepared by treatment of **Ru** ^**H**^ **-Cl** with AgPF_6_ in the presence of an excess of the desired benzonitrile (X = – OMe, – Me, – H, – Cl, – CF_3_; Scheme 2b). The incorporation of a single benzonitrile ligand in each complex was confirmed via NMR; in the aromatic region of the ^1^H spectrum, resonances consistent with the newly introduced NCAr^X^ ligand were observed that displayed the expected integration for a single benzonitrile moiety (Figure 10). ^31^P NMR data revealed that the resonance corresponding to the PPh_3_ ligand appeared at a similar chemical shift to that for the corresponding CH_3_CN complexes (**Ru** ^**X**^ **-NCCH**_**3**_, ∼35 ppm in CDCl_3_) across this series (Table S1) and correlated linearly with σ_p_, similar to what was observed for **Ru** ^**X**^ **-PAr**_**3**_ complexes (Figure S97). Exchange of the nitrile ligand is facile, at least in neat acetonitrile, as ^1^H NMR data for **Ru** ^**H**^ **-NCAr** ^**H**^ obtained in CD_3_CN indicates complete conversion to **Ru** ^**H**^ **-NCCH**_**3**_ and free PhCN within minutes from sample preparation (Figure S96).

**Figure 10:**
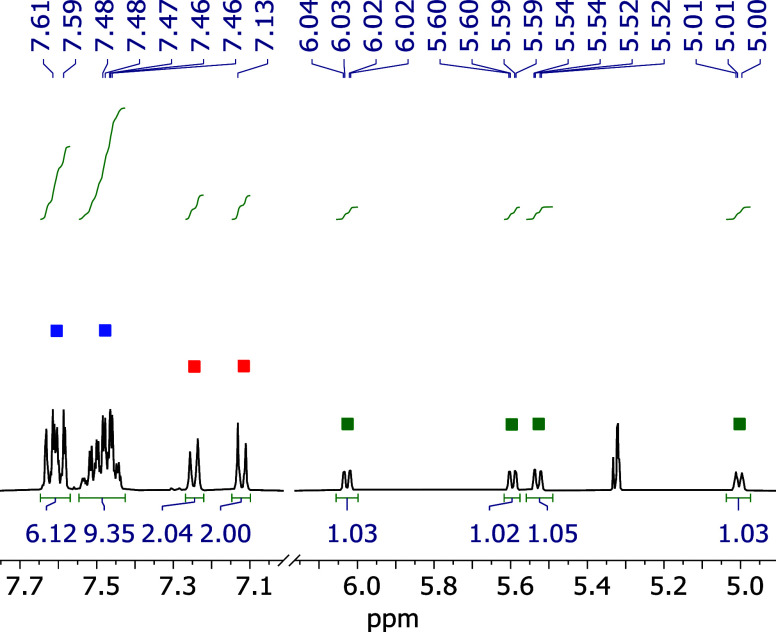
Partial ^1^H NMR spectrum (400 MHz, CD_2_Cl_2_) for complex **Ru** ^H^ **-NCAr** ^Me^. Peaks corresponding to three of the ligands in this complex are labeled: PPh_3_ ligand (blue squares), Ar^Me^CN ligand (red squares), and *p*-cymene (green squares). The relative integrations are consistent with the expected 1:1:1 PPh_3_/Ar^Me^CN/*p*-cymene ligand stoichiometry.

Solid-state structures were obtained from XRD data for all **Ru** ^**H**^ **-NCAr** ^**X**^ complexes and are shown in Figure 11. All complexes adopt the expected piano-stool structure, with *p*-cymene bound in an η^6^ fashion, a single Cl^–^ ligand, a PPh_3_ moiety, and the substituted benzonitrile ligand. The bond and angle metrics, summarized in Table 5, correlate with those of solvento complex **Ru** ^**OMe**^ **-NCCH**_**3**_.^44^ As expected, the nitrile moieties are nearly linear (Ru–N–C angles = 174–178°); no significant trends are observed (e.g., vs σ_p_ for the X substituents) in Ru–P, Ru–Cl, Ru–N, or Ru–C bond lengths, nor in the distance between Ru and the *p*-cymene ligand, indicating that the nitrile-based substituents have minimal effects on the structural profile of these compounds.

**Table 5:** Selected Bond and Angle Metrics for Benzonitrile Complexes **Ru** ^H^ **-NCAr** ^X^

	Ru^H^-NCAr^OMe^	Ru^H^-NCAr^Me^	Ru^H^-NCAr^H^	Ru^H^-NCAr^Cl^	**Ru** ^**H**^ **-NCAr** ^**CF3**^ ^a^
CCDC #	2532845	2532848	2532844	2532846	2532850

	bond lengths (Å)				
Ru–(cymene)^b^	1.731(3)	1.7283(10)	1.7238(10)	1.7311(18)	1.7205
Avg. Ru–C^c^	2.230 ± 0.034	2.233 ± 0.037	2.228 ± 0.030	2.232 ± 0.040	2.225 ± 0.032
Ru–Cl	2.3849(19)	2.3972(6)	2.4066(7)	2.3973(12)	2.3922
Ru–P	2.3774(19)	2.3585(7)	2.3562(7)	2.3571(12)	2.361
Ru–N	2.063(6)	2.040(2)	2.0439(18)	2.039(3)	2.027
C–N	1.108(9)	1.144(3)	1.139(3)	1.133(5)	1.142

	bond angles (°)				
Cl–Ru–P	85.91(7)	85.97(2)	88.53(2)	86.55(4)	86.46
Cl–Ru–N	86.70(17)	85.06(6)	85.18(5)	85.97(10)	84.70
P–Ru–N	85.28(17)	86.89(6)	87.68(6)	86.83(11)	87.21
Ru–N–C	177.8(6)	175.9(2)	175.09(18)	175.7(4)	173.6

a Average of values for three independent molecules in the asymmetric unit cell.

b Distance between Ru and the calculated centroid of the cymene ligand.

c Average distance between Ru and cymene C atoms.

**Figure 11:**
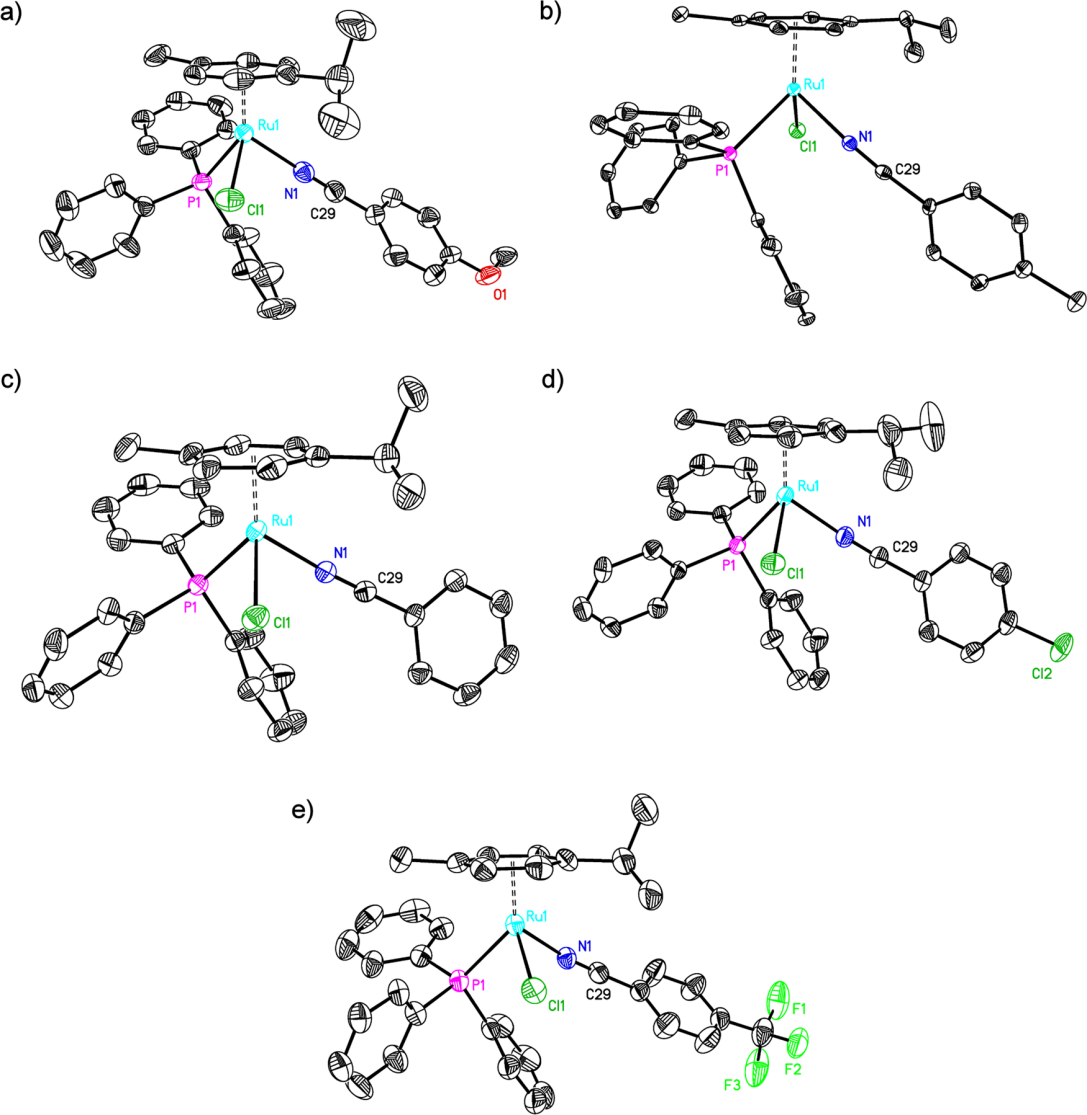
Solid-state structures (XRD) of **Ru** ^H^ **-NCAr** ^X^ complexes. Diffraction data collected at 100 K. Hydrogen atoms and outer-sphere counteranions not shown for clarity. Displacement ellipsoids shown at 50% probability level. (a) **Ru** ^H^ **-NCAr** ^OMe^; (b) **Ru** ^H^ **-NCAr** ^Me^; (c) **Ru** ^H^ **-NCAr** ^H^; (d) **Ru** ^H^ **-NCAr** ^Cl^; (e) **Ru** ^H^ **-NCAr** ^CF3^ (only one of three independent molecules in the asymmetric unit cell shown for clarity).

Complexes **Ru** ^**H**^ **-NCAr** ^**X**^ undergo *quasi*reversible 1e^–^ oxidations at potentials near +1.3 V vs Fc^+/0^ in CH_2_Cl_2_ (Figure 12). Hammett analysis for this series of complexes according to eq 1 gave a Hammett reaction constant value of 1.4 (Figure 13, left panel), suggesting that despite the structural differences between benzonitrile and triarylphosphine ligands, the electronic communication between the ligand-based substituents and Ru is not substantially different in **Ru** ^**H**^ **-NCAr** ^**X**^ vs **Ru** ^**X**^ **-Cl** complexes. Modeling of electrochemically generated **A** ^**H**^ **-NCAr** ^**X**^ complexes as mono-, bis-, and tris­(benzonitrile) compounds yields ρ values of 3.2, 1.6, and 1.1, respectively (Figure 13, right panel). Surprisingly, none of these values are in perfect agreement with that obtained from monobenzonitrile complexes **Ru** ^**H**^ **-NCAr** ^**X**^. Comparison of the slopes of the *E* vs σ_p_ plots for complexes **Ru** ^**H**^ **-NCAr** ^**X**^ and **A** ^**H**^ **-NCAr** ^**X**^ (Figure S115) showcases the same incongruence: in these plots, complexes supported by substituted ligands of the same type (e.g., benzonitriles or triarylphosphines) are expected to display correlations that depend on the number of ligands present–for example, monophosphine complexes **Ru** ^**X**^ **-Cl** and **Ru** ^**X**^ **-NCCH**_**3**_ display correlations with slopes of 0.24 and 0.23 V, respectively, while that for the series of bis­(phosphines) **Ru** ^**X**^ **-PAr**_**3**_ gives a slope of 0.46 V, close to twice the former values due to the presence of double the number of substituents (Figure 6). However, complexes **A** ^**H**^ **-NCAr** ^**X**^, which are postulated as tris­(benzonitrile) compounds, give a slope of 0.19 V, while monobenzonitriles **Ru** ^**H**^ **-NCAr** ^**X**^ give a value of 0.084 V–a ratio of 2.3:1, not quite the expected 3:1 ratio that would clearly suggest incorporation of three benzonitrile ligands.

**Figure 12:**
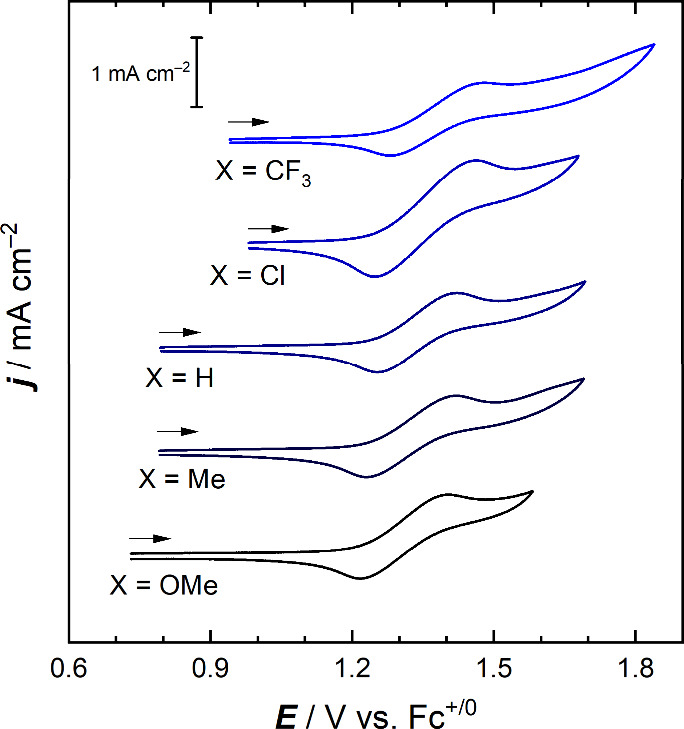
CV data (0.1 *M* [^n^Bu_4_N]­[PF_6_] in CH_2_Cl_2_, 100 mV/s, [Ru] = 2 m*M*) for complexes **Ru** ^H^ **-NCAr** ^X^.

**Figure 13:**
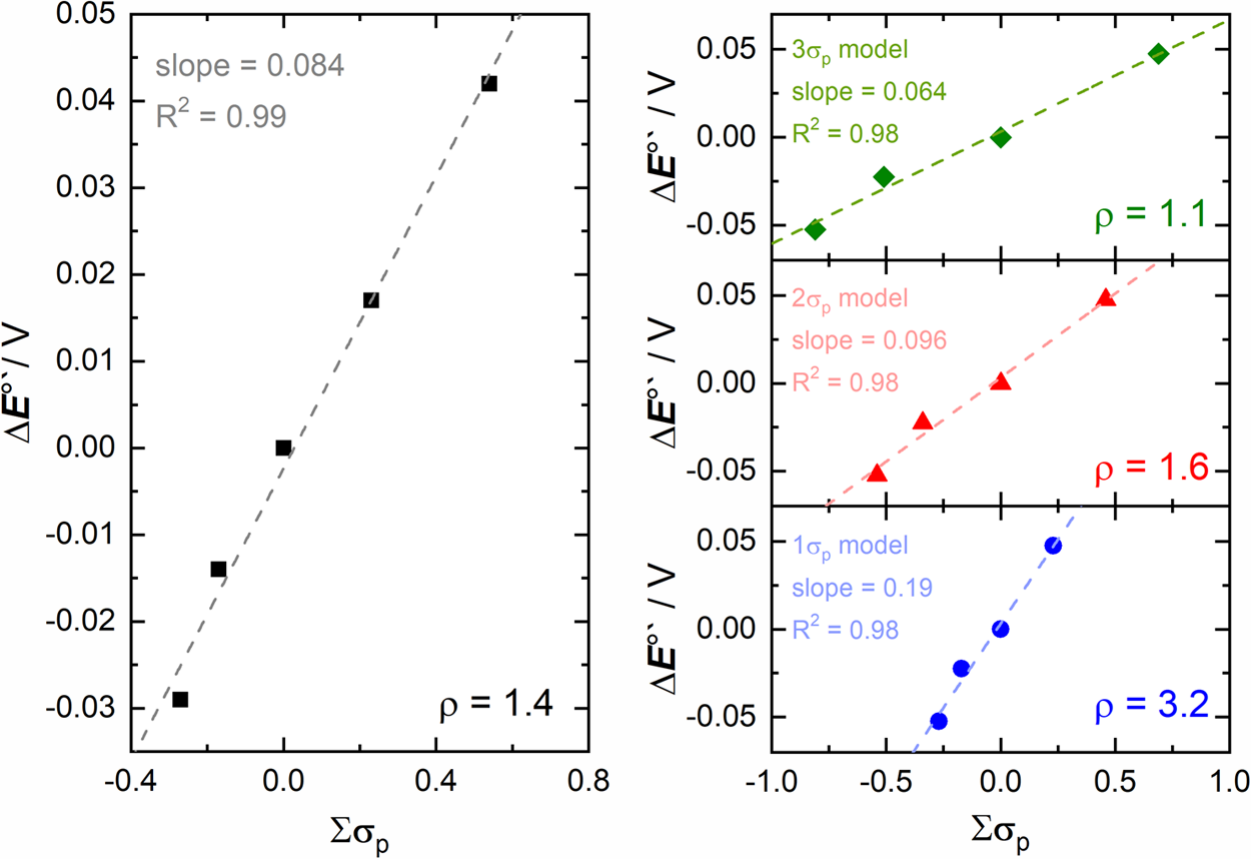
Left panel: Hammett plot for monobenzonitrile **Ru** ^H^ **-NCAr** ^X^ complexes. Right panel: Hammett plots for *in situ*-generated **A** ^H^ **-NCAr** ^X^ complexes modeled as mono- (blue circles, lower panel), bis- (red triangles, middle panel), and trisbenzonitrile (green diamonds, upper panel) complexes. The slopes and R^2^ values for the lines of best fit and the calculated values of ρ from each series are included.

These observations may indicate that electrochemical oxidation results in an equilibrium between complexes bearing different number of benzonitrile ligands (e.g., a mixture of bis- and tris­(benzonitrile) complexes), possibly via exchange with other exogenous ligands or through dimerization. Alternatively, the electronic communication between ligand-based substituents and the Ru center may depend on the orientation of the substituted ligands, resulting in a loss of additivity of substituent effects for certain geometries. The geometry-dependent nature of the additivity of ligand contributions to reduction potential in various electrochemical models has been described by Bursten and Green;^87^ differential effects of ligands on metal reduction potential due to *fac* vs *mer* geometries have been observed for molybdenum carbonyl complexes containing phosphine or isocyanide complexes.^88^ Nonetheless, the incorporation of multiple nitrile ligands is supported by the electrochemical data. Therefore, these results corroborate the long-standing hypothesis that dissociation of cymene is accompanied by binding of exogenous nitrile ligands upon oxidation of complexes **Ru** ^**X**^ **-Cl**, **Ru** ^**X**^ **-NCCH**_**3**_, and **Ru** ^**X**^ **-PAr**_**3**_.

### Synthesis and Electrochemical Studies of C^X^-L Complexes

As previously described, attempts to independently synthesize **A** ^**X**^ **-NCCH**_**3**_ complexes via chemical means were unsuccessful.^40^ Likewise, attempts to isolate **A** ^**H**^ **-NCCH**_**3**_ via bulk electrolysis according to procedures by Wright^42^ yielded a mixture of species likely involving exchange of Cl^–^ for CH_3_CN (see SI). This is consistent with the sparse literature on these complexes: only a single isomer of **A** ^**H**^ **-NCCH**_**3**_ (prepared via a procedure that was found to have limited reproducibility) has been structurally characterized,^89^ and isomers of **B** ^**H**^ **-NCCH**_**3**_ have only been observed as countercations for other Ru species.^90^ However, the *trans*-*mer* isomer of bis­(phosphine) byproduct **C** ^**H**^ **-NCCH**_**3**_ (Chart 1) has been reported from the treatment of RuCl_2_(PPh_3_)_3_ with NaBPh_4_ or NaClO_4_ in CH_3_CN.^91^ Preparation of the *cis*-*mer* isomer of **C** ^**H**^ **-NCCH**_**3**_ has also been described, though no structural characterization of this complex has been reported.^86^ Based on this precedent, we opted to further explore the electrochemical properties of tris­(nitrile) complexes containing two phosphine ligands to provide insight into the geometry of complexes **C** ^**X**^ **-NCCH**_**3**_, for which three isomeric forms are possible (Chart 1), generated following electrochemical oxidation of **Ru** ^**X**^ **-PAr**_**3**_ compounds.

[*trans*-(PPh_3_)_2_-*mer*-(NCCH_3_)_3_-RuCl]­[BPh_4_] was prepared according to the literature procedure^91^ and its ET profile investigated via CV methods (Figure S116). While preliminary results were promising, with an ET event (*E°′* = +0.87 V) observed near the potential for the byproduct generated by electrochemical oxidation of **Ru** ^**H**^ **-PAr**_**3**_ in CH_3_CN, the redox noninnocence of the BPh_4_^–^ anion (*E*_pa_ = +0.49 V vs Fc^+/0^,^92^ Figure S121) made conclusive comparison of the CV data challenging. Therefore, the PF_6_^–^ salt of this complex was prepared via treatment of RuCl_2_(PPh_3_)_3_ with KPF_6_ in refluxing CH_3_CN (Scheme 4a). NMR characterization of this compound in CD_3_CN revealed a singlet in the ^31^P NMR spectrum, consistent with a single P environment, ruling out generation of the *cis*-*mer* isomer. ^1^H NMR data indicated two intricate signals in the aromatic region, integrating to a total of 30 H, and two resonances in the aliphatic region (δ 1.86 and 1.31 ppm) integrating to 6 and 3 H, respectively. These upfield signals appeared as triplets (*J* = 1.1 and 0.8 Hz, respectively), which coalesced to singlets in a ^31^P-decoupled spectrum (Figure S100), supporting their assignment as the two inequivalent CH_3_CN ligands in **C** ^**H**^ **-NCCH**_**3**_ (Chart 1). Exchange of the bound CH_3_CN ligands with solvent is sufficiently slow for NMR characterization in CD_3_CN, though irradiation with visible light results in complete exchange with CD_3_CN over the course of several hours, as evidenced by the disappearance of the upfield triplets and the concurrent increase in the amount of free CH_3_CN (Figure 14). Despite numerous attempts, we have been unable to prepare high-quality single crystals of this material; nonetheless, a preliminary XRD structure–of sufficient quality only for connectivity assignments but not for full refinement–supported the *trans* arrangement of the two PPh_3_ ligands (Figure S129). Based on these results and on comparison with literature data, this complex can be assigned as [*trans*-(PPh_3_)_2_-*mer*-(NCCH_3_)_3_-RuCl]­[PF_6_] (*trans*-*mer*-**C** ^**H**^ **-NCCH**_**3**_; Scheme 4a).

**Scheme 4:**
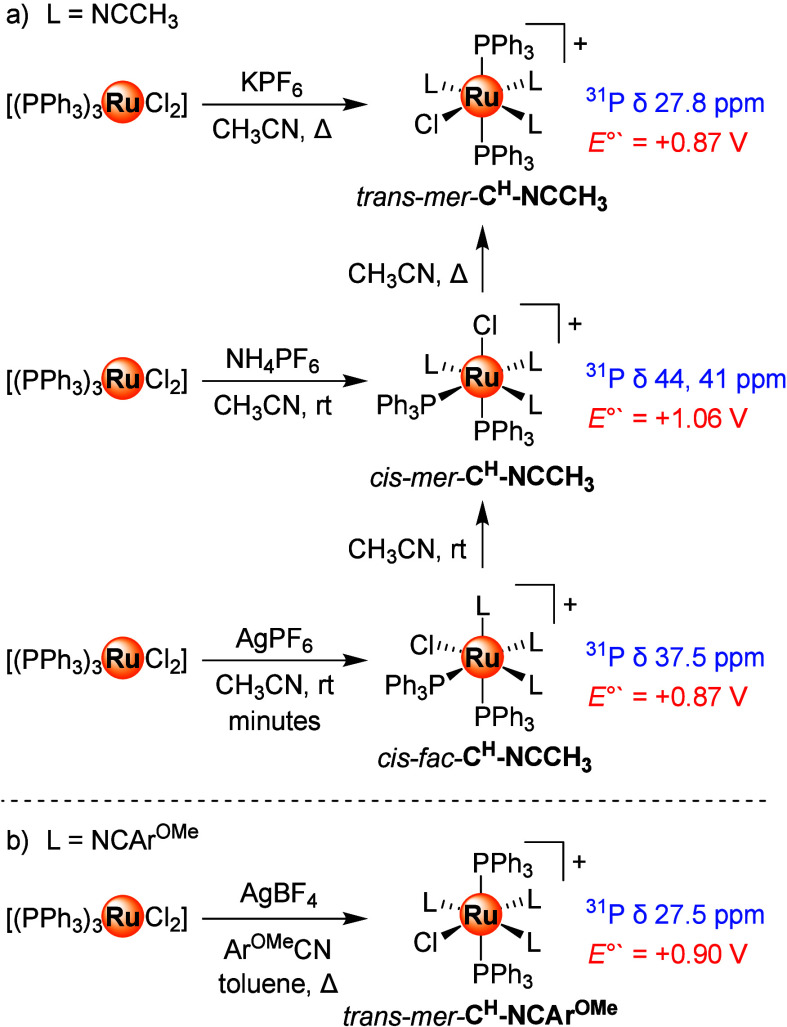
Synthesis of **C** ^**H**^ **-L** Complexes and Relevant ^31^P NMR and Potential Data

**Figure 14:**
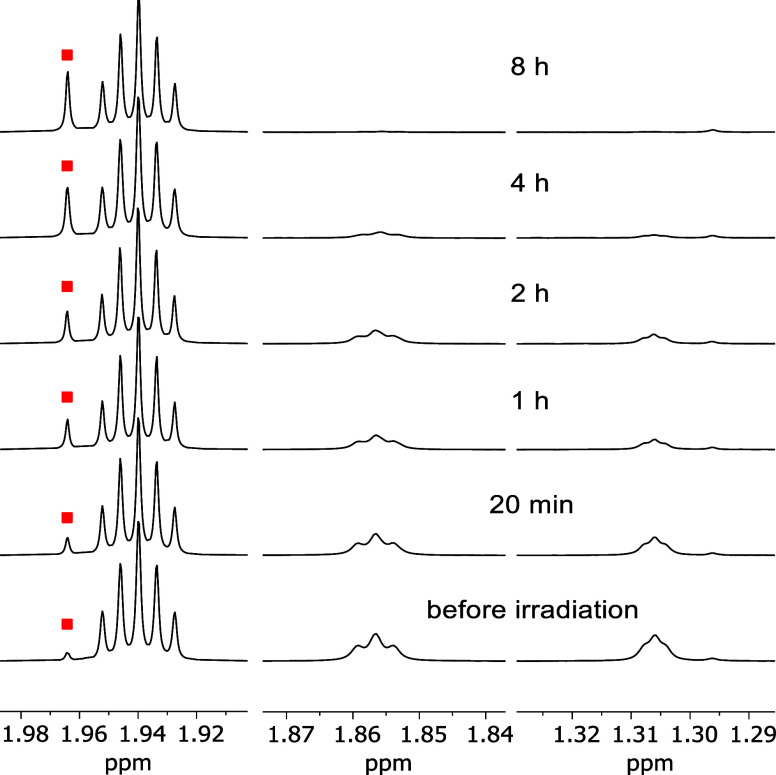
Partial ^1^H NMR spectra (400 MHz, CD_3_CN) for complex *trans*-*mer*-**C** ^H^ **-NCCH**_3_ during irradiation with visible light from a compact fluorescence light bulb showing disappearance of the triplet signals (1.86 and 1.31 ppm) corresponding to the Ru-bound CH_3_CN ligands with solvent acetonitrile. The peaks corresponding to free CH_3_CN (1.96 ppm) are marked with red squares. The pentet at 1.94 ppm is the solvent residual peak.^54^

CV data for *trans*-*mer*-**C** ^**H**^ **-NCCH**_**3**_ in CH_3_CN confirmed the presence of a *quasi*reversible event at +0.87 V vs Fc^+/0^, almost identical to that for electrochemically generated **C** ^**H**^ **-NCCH**_**3**_ (Figure 15). Yet, this finding is in contrast with the postulated fate of literature complexes supported by chelating bis­(phosphine) ligands, which generated the corresponding *fac*-(NCCH_3_)_3_ products following electrochemical oxidation (though the chelating nature of these ligands prevents *trans* arrangement of the two P donors in these cases).^43^ To provide additional clues into the nature of the geometry of our **C** ^**X**^ **-NCCH**_**3**_ byproducts, chemical preparation of other isomers of these complexes was undertaken. Treatment of RuCl_2_(PPh_3_)_3_ with NH_4_PF_6_ in place of KPF_6_ at room temperature in CH_3_CN according to the literature procedure by Fogg and James^86^ provided access to the *cis*-*mer* isomer of **C** ^**H**^ **-NCCH**_**3**_ based on NMR characterization. In our hands, the reproducibility of this procedure was found to be limited, with varying amounts (5–20%) of the corresponding *trans*-*mer* isomer and of other P-containing complexes observed as byproducts. Nonetheless, in agreement with the earlier report, the major species observed in these mixtures displayed two broad signals by ^31^P NMR (44.1 and 40.7 ppm, respectively), consistent with *cis*-*mer*-**C** ^**H**^ **-NCCH**_**3**_ (Scheme 4a). Further spectroscopic studies provided new insight into the ^1^H NMR spectrum for this complex and suggested its dimerization in noncoordinating solvent (see SI). CV data collected for this compound in CH_3_CN indicates that this complex undergoes a 1e^–^ oxidation at a potential of +1.06 V vs Fc^+/0^ (Figure 15, red trace). This reduction potential is ∼200 mV more positive than that for the oxidation of the *trans*-*mer* isomer, and is inconsistent with the product generated electrochemically from **Ru** ^**H**^ **-PAr**_**3**_. These results rule out formation of the *cis*-*mer* isomer upon reaction of the oxidized form of **Ru** ^**H**^ **-PAr**_**3**_ with CH_3_CN.

**Figure 15:**
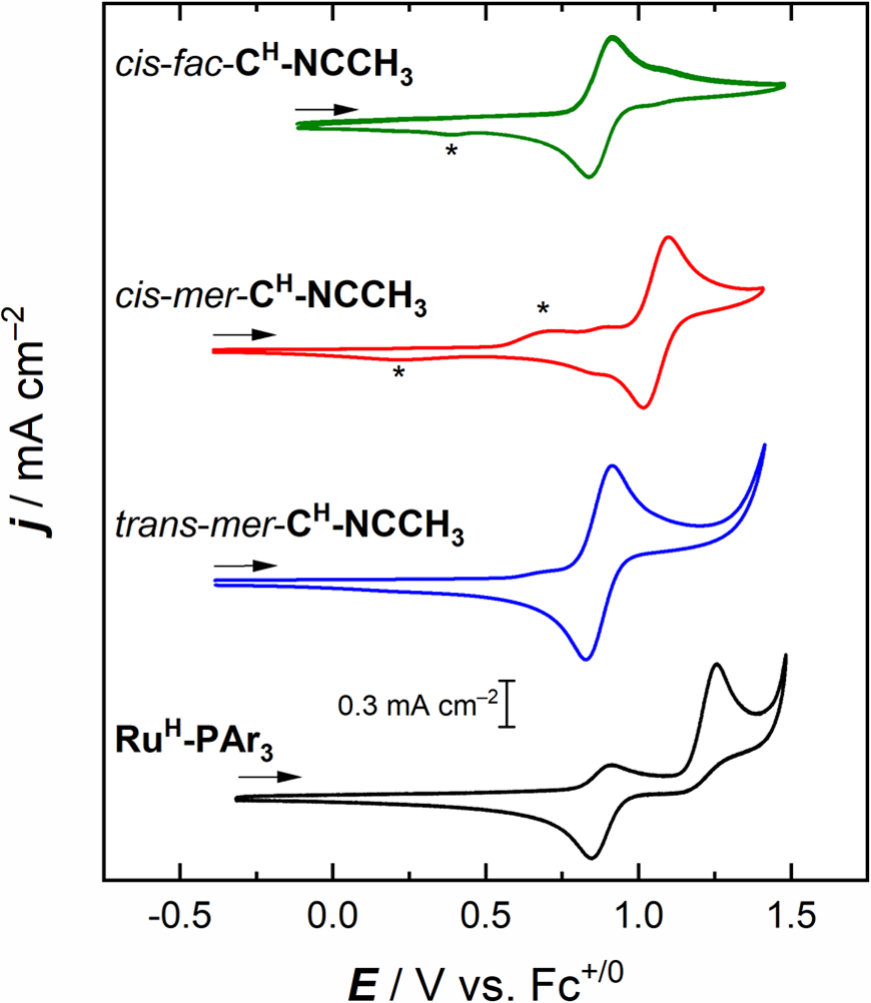
CV data (0.1 *M* [^n^Bu_4_N]­[PF_6_] in CH_3_CN, 100 mV/s; [Ru] = 3 m*M*) for complexes **Ru** ^H^ **-PAr**_3_ (lower trace, black; second voltammetric cycle only), *trans*-*mer*-**C** ^H^ **-NCCH**_3_ (second trace, blue), *cis*-*mer*-**C** ^H^ **-NCCH**_3_ (third trace, red), and *cis*-*fac*-**C** ^H^ **-NCCH**_3_ (upper trace, green). Peaks marked with (*) correspond to Ru impurities.

Treatment of RuCl_2_(PPh_3_)_3_ with AgPF_6_ in CH_3_CN followed by immediate work up resulted in isolation of a new product; analysis of this material by ^31^P NMR revealed a major product with a single resonance at 37.5 ppm (Figure S103), consistent with a single phosphine environment and a different identity from the *trans*-*mer* isomer of **C** ^**H**^ **-NCCH**_**3**_ (δ 27.8 ppm). CV data collected for this product in CH_3_CN revealed one prominent oxidation event at +0.87 V vs Fc^+/0^ (Figure 15, green trace); this is virtually identical to that for the *trans*-*mer* isomer, though the NMR data was indicated the presence of a *different* species as the major component of this mixture. Based on this observation, we hypothesize that the reaction of RuCl_2_(PPh_3_)_3_ with AgPF_6_ generates, at least initially, the *cis*-*fac* isomer of **C** ^**H**^ **-NCCH**_**3**_ as the major product (see SI for a proposed mechanism).

The fate of the putative *cis*-*fac* isomer of **C** ^**H**^ **-NCCH**_**3**_ was investigated through parallel experiments in which solutions of this complex were monitored via NMR and CV methods, respectively (Figure 16; Table S2). Over the course of several hours, analyses of this material indicated conversion of the initial product (^31^P NMR: singlet at 37.5 ppm; CV: *quasi*reversible event at +0.87 V) into the *cis-mer* isomer of **C** ^**H**^ **-NCCH**_**3**_ (^31^P NMR: broad signals at 44 and 41 ppm; CV: *quasi*reversible event at +1.06 V). Heating of the respective solutions converted this compound to the *trans*-*mer* isomer (^31^P NMR: singlet at 27.8 ppm; CV: *quasi*reversible event at +0.87 V). These observations support the initial generation of a complex consistent with the *cis*-*fac* isomer of **C** ^**H**^ **-NCCH**_**3**_ and which displays a reduction potential of +0.87 V. At room temperature, this species converts to the previously characterized *cis*-*mer* isomer of **C** ^**H**^ **-NCCH**_**3**_, which can further be converted to the corresponding *trans*-*mer* isomer upon heating. While the Ru­(III/II) couples of both the initial and final product in these experiments coincidentally occur at the same potential (+0.87 V), the different NMR profiles of these two species (singlets at 37.5 vs 27.8 ppm, respectively) indicate different structures for these compounds. Since the complex displaying a singlet at 27.8 ppm in its ^31^P NMR spectrum was identified as *trans*-*mer*-**C** ^**H**^ **-NCCH**_**3**_, we conclude that chloride abstraction from RuCl_2_(PPh_3_)_3_ initially generates the *cis*-*fac* isomer of the same complex. Based on these analyses, we postulate that the *fac*-(NCCH_3_)_3_ isomer of **C** ^**H**^ **-NCCH**_**3**_ is generated following oxidation of **Ru** ^**H**^ **-PAr**_**3**_.

**Figure 16:**
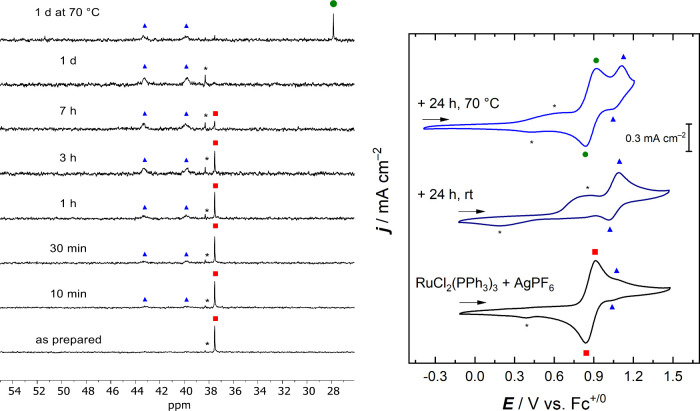
Left panel: ^31^P­{^1^H} NMR (162 MHz, CD_3_CN) of the material obtained from treatment of RuCl_2_(PPh_3_)_3_ with AgPF_6_ over time. Right panel: CV data (0.1 M [^n^Bu_4_N]­[PF_6_] in CH_3_CN, 100 mV/s; [Ru] = 3 mM) for the same material over time. The signals corresponding to the putative *cis*-*fac*-**C** ^H^ **-NCCH**_3_ are labeled with red squares; the signals corresponding to *cis*-*mer*-**C** ^H^ **-NCCH**_3_ are labeled with blue triangles; the signals for *trans*-*mer*-**C** ^H^ **-NCCH**_3_ are marked with green circles; signals marked with (*) correspond to other Ru impurities.

### Studies of C^H^-NCAr^X^ Complexes

To provide additional evidence for this assignment, we investigated the properties of **C** ^**H**^ complexes bearing substituted benzonitrile ligands. Addition of 4-substituted benzonitriles (^X^ArCN; X = Cl, H, OMe) to solutions of **Ru** ^**H**^ **-PAr**_**3**_ in CH_2_Cl_2_ resulted in CV profiles consistent with generation of benzonitrile-containing **C** ^**H**^ **-NCAr** ^**X**^ species following electrochemical oxidation (Scheme 3). These byproducts displayed *quasi*reversible events at potentials between +0.85 and +1.0 V that varied as a function of the identity of the X substituents (Figure 17, Table S4). A very similar dependence (slope = 0.19 V, Figure S115) of these potentials on σ_p_ was observed to that for the corresponding **A** ^**H**^ **-NCAr** ^**X**^ complexes (slope = 0.19 V), possibly indicative of similar structural features.

**Figure 17:**
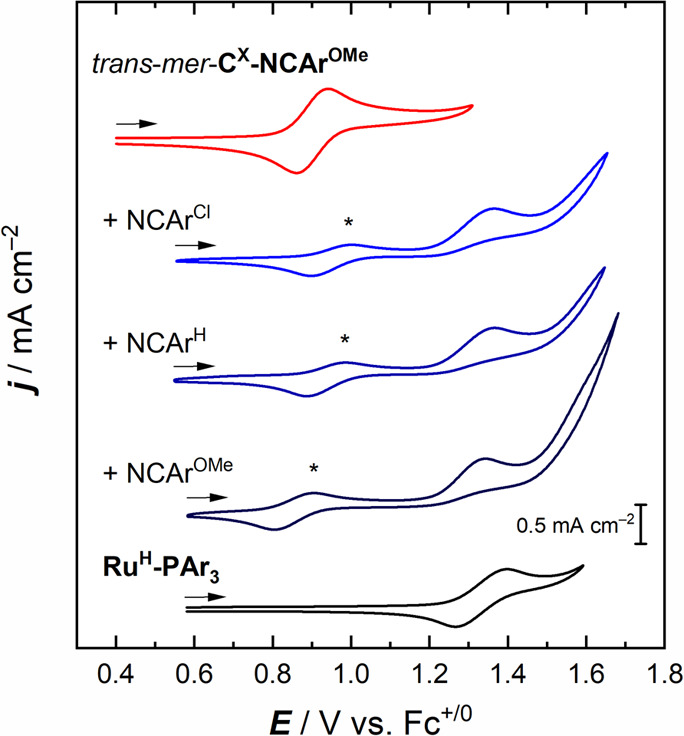
Lower traces: CV data (0.1 *M* [^n^Bu_4_N]­[PF_6_] in CH_2_Cl_2_, 100 mV/s, [Ru] = 2 m*M*) for complex **Ru** ^H^ **-PAr**_3_ in the presence of *para*-substituted benzonitriles (NCAr^X^, X = OMe, H, Cl). Only the second voltammetric scans are shown for clarity; events corresponding to **C** ^H^ **-NCAr** ^X^ species are marked with (*). Concentration of NCAr^X^ = 0.4 *M* (X = OMe, H; 200 equiv) or 0.8 *M* (X = Cl; 400 equiv). Top trace: CV data (0.1 *M* [^n^Bu_4_N]­[PF_6_] in CH_3_CN, 100 mV/s, [Ru] = 3 m*M*) for complex **C** ^H^ **-NCAr** ^OMe^.

Reaction of RuCl_2_(PPh_3_)_3_ with AgBF_4_ and an excess of 4-methoxybenzonitrile (20 equiv) in toluene (100 °C, 18 h) provided a material displaying features in its NMR spectra consistent with those for the *trans*-*mer* isomer of **C** ^**H**^ **-NCAr** ^**OMe**^–a singlet in its ^31^P spectrum (δ 27.5 ppm) and resonances consistent with two benzonitrile environments in its ^1^H data (Scheme 4b). Single-crystal XRD analysis of this compound revealed two PPh_3_ ligands in a *trans* arrangement and three NCAr^OMe^ ligands in a *meridional* configuration, confirming the expected *trans*-*mer* isomeric form (Figure 18). The structural metrics for this complex are similar to those reported in the literature for *trans*-*mer*-**C** ^**H**^ **-NCCH**_**3**_ with a BPh_4_^–^ counteranion (Table S8), suggesting minimal structural effects of the electron-donating OMe substituents. The conclusive identification of *trans-mer*-**C** ^**H**^ **-NCAr** ^**OMe**^ is consistent with the similarity of its spectroscopic features to those of its acetonitrile analogue (^31^P NMR: δ 27.8 ppm), further corroborating the assignment of the latter as the corresponding *trans*-*mer* isomer of complex **C** ^**H**^ **-NCCH**_**3**_. CV data collected for a solution of this complex revealed a single event centered at +0.90 V vs Fc^+/0^ (Figure 21, upper trace). This potential differs from that determined for the electrochemically generated product in CV data collected for **Ru** ^**H**^ **-PAr**_**3**_ in the presence of 4-methoxybenzonitrile (*E°′* = +0.86 V vs Fc^+/0^), indicating that a different isomer of **C** ^**H**^ **-NCAr** ^**OMe**^ is generated under electrochemical conditions. This observation is consistent with the generation of *cis*-*fac*-**C** ^**H**^ **-NCAr** ^**OMe**^ under electrochemical conditions and supports the coincidental nature of the similar Ru­(III/II) potentials determined for the *cis*-*fac* and *trans*-*mer* isomers of **C** ^**H**^ **-NCCH**_**3**_.

**Figure 18:**
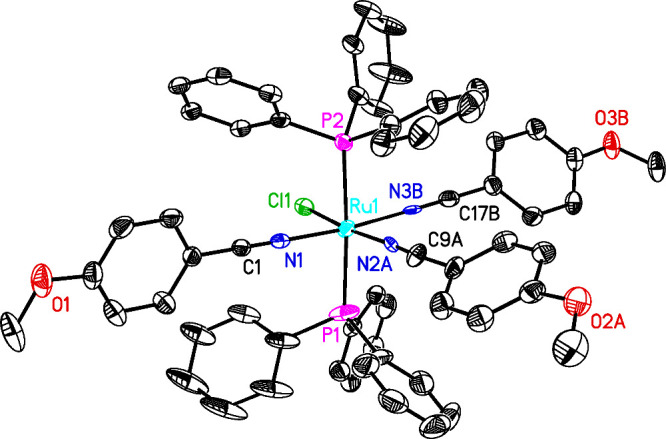
Solid-state structure (XRD) of *trans-mer*-**C** ^H^ **-NCAr** ^OMe^. Diffraction data collected at 100 K. Hydrogen atoms, outer-sphere BF_4_^–^ counteranion, and cocrystallized solvent molecules not shown for clarity; only the major orientations (50–60%) of disordered fragments (two benzonitrile ligands and one Ph ring on PPh_3_ ligand) shown for clarity. Displacement ellipsoids are shown at the 50% probability level.

Taken together, our observations support the assignment of the product of ET-induced reactivity in **Ru** ^**X**^ **-PAr**_**3**_ complexes as the *cis-fac* isomer of [RuCl­(PAr^X^_3_)_2_(NCCH_3_)_3_]^+^. Given that even bis­(phosphine) complexes do not generate the tris­(nitrile) complex that is expected to minimize steric clash between the two bulky phosphine ligands (i.e., with the two PAr^X^_3_ ligands in a *trans* arrangement), and given the apparent similarities in ET-induced reactivity profile across the various series of complexes presented here, we further hypothesize that oxidized monophosphine **Ru** ^**X**^ **-Cl** and solvento **Ru** ^**X**^ **-NCCH**_**3**_ complexes also react with CH_3_CN via replacement of cymene with acetonitrile ligands in a *facial* arrangement, resulting in generation of *cis*-*fac*-**A** ^**X**^ **-NCCH**_**3**_ and *cis*-**B** ^**X**^ **-NCCH**_**3**_ (Chart 1), respectively, following electrochemical oxidation.

## Conclusions

Electrochemical analyses of dichloride, solvento, bis­(phosphine), and benzonitrile complexes indicate that (arene)Ru complexes containing phosphine ligands react with exogenous nitriles upon oxidation to Ru­(III), resulting in arene displacement and binding of multiple equivalents of nitrile ligands. These products were shown to contain the same number of phosphine ligands as their precursors on the basis of Hammett analyses of the effects of *para*-substituted triarylphosphines on the potential for ET at Ru in both mono- (**Ru** ^**X**^ **-Cl**, **Ru** ^**X**^ **-NCCH**_**3**_) and bis­(phosphine) compounds (**Ru** ^**X**^ **-PAr**_**3**_). Investigations of solvento complexes (**Ru** ^**X**^ **-NCCH**_**3**_) supported retention of two Cl^–^ ligands in reactivity of dichloride complexes **Ru** ^**X**^ **-Cl**. Studies of the reactivity of **Ru** ^**X**^ **-Cl** with benzonitriles following oxidation to Ru­(III) confirmed nitrile incorporation into the downstream products, providing clear evidence for the process responsible for the previously observed displacement of the arene ligand. Structurally well-defined monobenzonitrile complexes (**Ru** ^**H**^ **-NCAr** ^**X**^) were also investigated to provide a direct point of comparison to experiments involving addition of exogenous benzonitriles to complex **Ru** ^**H**^ **-Cl** in CV experiments. While Hammett analysis of the reduction potentials for nitrile complexes generated electrochemically vs monobenzonitrile compounds **Ru** ^**H**^ **-NCAr** ^**X**^ was consistent with incorporation of multiple nitriles, it also suggested incomplete additivity of substituent effects in tris­(nitrile) complexes, preventing conclusive confirmation of the presence of three CH_3_CN ligands in these products. Nonetheless, on the basis of the available evidence, it was concluded that **A** ^**X**^ **-NCCH**_**3**_ complexes are best formulated as RuCl_2_(PAr^X^_3_)­(NCCH_3_)_3_ species; analogous reactivity leads to generation of [RuCl­(PAr^X^_3_)­(NCCH_3_)_4_]^+^ compounds (**B** ^**X**^ **-NCCH**_**3**_) from solvento complexes **Ru** ^**H**^ **-NCCH**_**3**_ and of [RuCl­(PAr^X^_3_)_2_(NCCH_3_)_3_]^+^ complexes (**C** ^**X**^ **-NCCH**_**3**_) from bis­(phosphines) **Ru** ^**X**^ **-PAr**_**3**_. Investigation of three isomers of bis­(phosphine) byproduct complex **C** ^**H**^ **-NCCH**_**3**_ (*cis*-*fac*, *cis*-*mer*, *trans*-*mer*), prepared via treatment of RuCl_2_(PPh_3_)_3_ with different Cl^–^-abstracting agents and identified through spectroscopic and structural methods, revealed that the most likely isomer of this compound that is generated via electrochemical oxidation of **Ru** ^**H**^ **-PAr**_**3**_ is [*cis*-(PPh_3_)_2_-*fac*-(NCCH_3_)_3_-RuCl]^+^, whose conversion to its *cis*-*mer* and, finally, *trans*-*mer* isomers under chemical conditions was also investigated. These results confirm that reactivity of the nascent Ru­(III) complex generates products in which one facially coordinated ligand is replaced with three monodentate ligands in the same facial arrangement.

## Supplementary Material



## Notes

Additional characterization data and spectroscopic assignments; NMR (^1^H, ^13^C, ^31^P, and ^19^F) spectra for characterization; stability studies of **Ru** ^**H**^ **-NCCH**_**3**_ and **Ru** ^**H**^ **-PAr**_**3**_ in CDCl_3_ and CD_3_CN; stability studies of **Ru** ^**H**^ **-Cl** in electrolyte solution; stability studies of **Ru** ^**H**^ **-NCAr** ^**H**^ in CD_3_CN; characterization of cis-*mer*-**C** ^**H**^ **-NCCH**_**3**_ in CD_3_CN vs acetone-*d*_6_; comparison of characterization for **C** ^**X**^ **-NCCH**_**3**_ and [*trans*-(PPh_3_)_2_-*mer*-(NCCH_3_)_3_-RuCl]­[BPh_4_]; discussion of mechanism for formation of *cis*-*fac*-**C** ^**H**^ **-NCCH**_**3**_; additional cyclic voltammetry data; discussion of reductive electrochemistry; discussion of bulk electrolysis of **Ru** ^**H**^ **-Cl**; additional Hammett plots; additional elemental analysis data for *cis*-*mer*-**C** ^**H**^ **-NCCH**_**3**_; additional X-ray diffraction data; additional discussion of XRD analyses; structural metrics for *trans*-*mer*-**C** ^**H**^ **-NCAr** ^**OMe**^; refinement details for all XRD structures (PDF)
