# Supplementary material for: Electrochemically Induced Reactivity of Ru–Arene Complexes Supported By Triarylphosphine Ligands
Source: Inorg Chem. 2026 Jun 17;65(26):14663–82. doi: 10.1021/acs.inorgchem.6c01267 (PMC13343465; doi:10.1021/acs.inorgchem.6c01267)
Supplement: Supplementary file 1 [file ic6c01267_si_001.pdf]

Supporting information for

Electrochemically-induced reactivity of Ru–arene complexes supported by triarylphosphine ligands

*Julia E. Fumo,[a] Anthony N. Micci,[a] Christopher Yang,[a] Robert D. Pike,[b] Davide Lionetti[a,]**

[a] Department of Chemistry, Franklin & Marshall College, P.O. Box 3003, Lancaster, PA 17604, USA
[b] Department of Chemistry, College of William & Mary, P.O. Box 8795, Williamsburg, VA 23187
* Email: dlionett@fandm.edu

Contents **Page**

NMR data S5
Characterization of new compounds S5
Additional characterization data S5
Figure S1. ^{1}H NMR spectrum of **Ru^{OMe}-NCCH$_3$** S7
Figure S2. ^{13}C{^{1}H} NMR spectrum of **Ru^{OMe}-NCCH$_3$** S7
Figure S3. ^{31}P{^{1}H} NMR spectrum of **Ru^{OMe}-NCCH$_3$** S8
Figure S4. ^{19}F NMR spectrum of **Ru^{OMe}-NCCH$_3$** S8
Figure S5. COSY spectrum of **Ru^{OMe}-NCCH$_3$** S9
Figure S6. HSQC spectrum of **Ru^{OMe}-NCCH$_3$** S9
Figure S7. HMBC spectrum of **Ru^{OMe}-NCCH$_3$** S10
Figure S8. NOESY spectrum of **Ru^{OMe}-NCCH$_3$** S10
Figure S9. ^{1}H NMR spectrum of **Ru^{Cl}-NCCH$_3$** S11
Figure S10. ^{13}C{^{1}H} NMR spectrum of **Ru^{Cl}-NCCH$_3$** S11
Figure S11. ^{31}P{^{1}H} NMR spectrum of **Ru^{Cl}-NCCH$_3$** S12
Figure S12. ^{19}F NMR spectrum of **Ru^{Cl}-NCCH$_3$** S12
Figure S13. COSY spectrum of **Ru^{Cl}-NCCH$_3$** S13
Figure S14. HSQC spectrum of **Ru^{Cl}-NCCH$_3$** S13
Figure S15. HMBC spectrum of **Ru^{Cl}-NCCH$_3$** S14
Figure S16. NOESY spectrum of **Ru^{Cl}-NCCH$_3$** S14
Figure S17. ^{1}H NMR spectrum of **Ru^{OMe}-NCCH$_3$** S15
Figure S18. ^{13}C{^{1}H} NMR spectrum of **Ru^{OMe}-NCCH$_3$** S15
Figure S19. ^{31}P{^{1}H} NMR spectrum of **Ru^{OMe}-NCCH$_3$** S16
Figure S20. ^{19}F NMR spectrum of **Ru^{OMe}-NCCH$_3$** S16
Figure S21. COSY spectrum of **Ru^{OMe}-NCCH$_3$** S17
Figure S22. HSQC spectrum of **Ru^{OMe}-NCCH$_3$** S17
Figure S23. HMBC spectrum of **Ru^{OMe}-NCCH$_3$** S18
Figure S24. NOESY spectrum of **Ru^{OMe}-NCCH$_3$** S18
Figure S25. ^{1}H NMR spectrum of **Ru^{Cl}-PAr$_3$** S19
Figure S26. ^{13}C{^{1}H} NMR spectrum of **Ru^{Cl}-PAr$_3$** S19
Figure S27. ^{31}P{^{1}H} NMR spectrum of **Ru^{Cl}-PAr$_3$** S20
Figure S28. ^{19}F NMR spectrum of **Ru^{Cl}-PAr$_3$** S20
Figure S29. COSY spectrum of **Ru^{Cl}-PAr$_3$** S21
Figure S30. HSQC spectrum of **Ru^{Cl}-PAr$_3$** S21
Figure S31. HMBC spectrum of **Ru^{Cl}-PAr$_3$** S22
Figure S32. NOESY spectrum of **Ru^{Cl}-PAr$_3$** S22
Figure S33. ^{1}H NMR spectrum of **Ru^{H}-NCAr^{OMe}** S23
Figure S34. ^{13}C{^{1}H} NMR spectrum of **Ru^{H}-NCAr^{OMe}** S23
Figure S35. ^{31}P{^{1}H} NMR spectrum of **Ru^{H}-NCAr^{OMe}** S24
Figure S36. ^{19}F NMR spectrum of **Ru^{H}-NCAr^{OMe}** S24

Figure S37. COSY spectrum of **Ru^{H}-NCAr^{OMe}**. S25
Figure S38. HSQC spectrum of **Ru^{H}-NCAr^{OMe}** S25
Figure S39. HMBC spectrum of **Ru^{H}-NCAr^{OMe}** S26
Figure S40. NOESY spectrum of **Ru^{H}-NCAr^{OMe}** S26
Figure S41. ^{1}H NMR spectrum of **Ru^{H}-NCAr^{Me}** S27
Figure S42. ^{13}C{^{1}H} NMR spectrum of **Ru^{H}-NCAr^{Me}** S27
Figure S43. ^{31}P{^{1}H} NMR spectrum of **Ru^{H}-NCAr^{Me}** S28
Figure S44. ^{19}F NMR spectrum of **Ru^{H}-NCAr^{Me}** S28
Figure S45. COSY spectrum of **Ru^{H}-NCAr^{Me}**. S29
Figure S46. HSQC spectrum of **Ru^{H}-NCAr^{Me}** S29
Figure S47. HMBC spectrum of **Ru^{H}-NCAr^{Me}** S30
Figure S48. NOESY spectrum of **Ru^{H}-NCAr^{Me}** S30
Figure S49. ^{1}H NMR spectrum of **Ru^{H}-NCAr^{Me}** S31
Figure S50. ^{13}C{^{1}H} NMR spectrum of **Ru^{H}-NCAr^{H}** S31
Figure S51. ^{31}P{^{1}H} NMR spectrum of **Ru^{H}-NCAr^{H}** S32
Figure S52. ^{19}F NMR spectrum of **Ru^{H}-NCAr^{H}** S32
Figure S53. COSY spectrum of **Ru^{H}-NCAr^{H}** S33
Figure S54. HSQC spectrum of **Ru^{H}-NCAr^{H}** S33
Figure S55. HMBC spectrum of **Ru^{H}-NCAr^{H}** S34
Figure S56. NOESY spectrum of **Ru^{H}-NCAr^{H}** S34
Figure S57. ^{1}H NMR spectrum of **Ru^{H}-NCAr^{Cl}** S35
Figure S58. ^{13}C{^{1}H} NMR spectrum of **Ru^{H}-NCAr^{Cl}** S35
Figure S59. ^{31}P{^{1}H} NMR spectrum of **Ru^{H}-NCAr^{Cl}** S36
Figure S60. ^{19}F NMR spectrum of **Ru^{H}-NCAr^{Cl}** S36
Figure S61. COSY spectrum of **Ru^{H}-NCAr^{Cl}** S37
Figure S62. HSQC spectrum of **Ru^{H}-NCAr^{Cl}** S37
Figure S63. HMBC spectrum of **Ru^{H}-NCAr^{Cl}** S38
Figure S64. NOESY spectrum of **Ru^{H}-NCAr^{Cl}** S38
Figure S65. ^{1}H NMR spectrum of **Ru^{H}-NCAr^{CF3}** S39
Figure S66. ^{13}C{^{1}H} NMR spectrum of **Ru^{H}-NCAr^{CF3}** S39
Figure S67. ^{31}P{^{1}H} NMR spectrum of **Ru^{H}-NCAr^{CF3}** S40
Figure S68. ^{19}F NMR spectrum of **Ru^{H}-NCAr^{CF3}** S40
Figure S69. COSY spectrum of **Ru^{H}-NCAr^{CF3}** S41
Figure S70. HSQC spectrum of **Ru^{H}-NCAr^{CF3}** S41
Figure S71. HMBC spectrum of **Ru^{H}-NCAr^{CF3}** S42
Figure S72. NOESY spectrum of **Ru^{H}-NCAr^{CF3}** S42
Figure S73. ^{1}H NMR spectrum of *trans-mer*-**C^{H}-NCCH$_3$** S43
Figure S74. ^{13}C{^{1}H} NMR spectrum of *trans-mer*-**C^{H}-NCCH$_3$** S43
Figure S75. ^{31}P{^{1}H} NMR spectrum of *trans-mer*-**C^{H}-NCCH$_3$** S44
Figure S76. ^{19}F NMR spectrum of *trans-mer*-**C^{H}-NCCH$_3$** S44
Figure S77. HSQC spectrum of *trans-mer*-**C^{H}-NCCH$_3$** S45
Figure S78. HMBC spectrum of *trans-mer*-**C^{H}-NCCH$_3$** S45
Figure S79. ^{1}H NMR spectrum of *cis-mer*-**C^{H}-NCCH$_3$** S46
Figure S80. ^{13}C{^{1}H} NMR spectrum of *trans-mer*-**C^{H}-NCAr^{OMe}** S46
Figure S81. ^{31}P{^{1}H} NMR spectrum of *trans-mer*-**C^{H}-NCAr^{OMe}** S47
Figure S82. ^{19}F NMR spectrum of *trans-mer*-**C^{H}-NCAr^{OMe}** S47
Figure S83. HSQC spectrum of *trans-mer*-**C^{H}-NCAr^{OMe}** S48
Figure S84. HMBC spectrum of *trans-mer*-**C^{H}-NCAr^{OMe}** S48
Figure S85. NOESY spectrum of *trans-mer*-**C^{H}-NCAr^{OMe}** S49
*Stability studies of complexes **Ru^{H}-NCCH$_3$** and **Ru^{H}-PAr$_3$*** S50
Figure S86. NMR spectra for complex **Ru^{H}-NCCH$_3$** in CDCl$_3$ over time. S52

Figure S87. NMR spectra for complex **Ru^{H}-NCCH$_3$** in CD_3CN over time. S53
Figure S88. NMR spectra for complex **Ru^{H}-NCCH$_3$** in CD_3CN in the dark over time. S54
Figure S89. NMR spectra for complex **Ru^{H}-PAr$_3$** in $CDCl_3$ over time. S55
Figure S90. NMR spectra for complex **Ru^{H}-PAr$_3$** in $CDCl_3$ (stored over K_2CO_3). S56
Figure S91. NMR spectra for complex **Ru^{H}-PAr$_3$** in $CDCl_3$ in the dark over time. S57
Figure S92. NMR spectra for complex **Ru^{H}-PAr$_3$** in $CDCl_3$ with trace HCl. S58
Figure S93. NMR spectra for complex **Ru^{H}-PAr$_3$** in CD_3CN over time. S59
Figure S94. NMR spectra for complex **Ru^{H}-PAr$_3$** in CD_3CN in the dark over time. S60
Figure S95. NMR monitoring of **Ru^{H}-Cl** in CH_3CN electrolyte over time. S61
Figure S96. NMR data for **Ru^{H}-NCAr^{H}** in CD_3CN. S61
Table S1. ^{31}P NMR chemical shifts for complexes **Ru^{X}-L** S62
Figure S97. Plot of $\delta(^{31}P)$ vs. σ_p for **Ru^{H}-NCAr^{X}** complexes S62
Figure S98. Comparison of NMR data $[RuCl(PPh_3)_2(NCCH_3)_3]X$ (X = PF_6^-, BPh_4^-) S63
Figure S99. Expanded view of NMR data $[RuCl(PPh_3)_2(NCCH_3)_3]X$. S63
Figure S100. ^{1}H and $^{1}H\{^{31}P\}$ NMR spectra for *trans-mer*-**C^{H}-NCCH$_3$** S64
*Characterization of cis-mer-**C^{H}-NCCH$_3$** in coordinating vs. non-coordinating solvent.* S65
Figure S101. NMR of *cis-mer*-**C^{H}-NCCH$_3$** in CD_3CN and acetone-*d*$_6$. S66
Figure S102. NMR of *cis-mer*-**C^{H}-NCCH$_3$** in dry CD_3CN. S66
Scheme S1. Dimerization of *cis-mer*-**C-H** S68
Figure S103. $^{31}P\{^{1}H\}$ NMR for *cis-fac*-**C^{H}-NCCH$_3$** S67
Table S2. Spectroscopic and electrochemical data for isomers of complex **C^{H}-NCCH$_3$** S68
*Proposed mechanism for formation of cis-fac-**C^{H}-NCCH$_3$** from $RuCl_2(PPh_3)_3$* S68
Scheme S2. Proposed mechanism for generation of *cis-fac*-C^{H}-NCCH$_3$ from $RuCl_2(PPh_3)_3$ S68

Electrochemistry S69
Figure S104. CV data for **Ru^{H}-Cl** in CH_3CN over time. S69
Table S3. Reduction potentials for complexes **Ru^{X}-NCCH$_3$** and **Ru^{X}-PAr$_3$** in CH_2Cl_2. .... S69
Figure S105. CV data for complexes **Ru^{X}-NCCH$_3$** in CH_2Cl_2. .... S70
Figure S106. CV data for complexes **Ru^{X}-PAr$_3$** in CH_2Cl_2. .... S70
Figure S107. CV data for complex **Ru^{H}-NCCH$_3$** at varying scan rates S71
Figure S108. CV data for complex **Ru^{H}-PAr$_3$** at varying scan rates. S71
*Reductive electrochemistry of **Ru^{X}-NCCH$_3$** and **Ru^{X}-PAr$_3$** complexes* S72
Figure S109. CV data for complexes **Ru^{X}-NCCH$_3$** in CH_2Cl_2 (cathodic scanning). S72
Figure S110. CV data for complexes **Ru^{X}-NCCH$_3$** in CH_3CN (cathodic scanning). S73
Figure S111. CV data for complexes **Ru^{X}-PAr$_3$** in CH_2Cl_2 (cathodic scanning). S73
Figure S112. CV data for complexes **Ru^{X}-PAr$_3$** in CH_3CN (cathodic scanning). S74
Figure S113. CV data for complex **Ru^{H}-Cl** at varying concentrations. S74
Figure S114. CV data for addition of Ar^{CF3}CN to **Ru^{H}-Cl** S75
Figure S115. Plot of $E°'$ vs. σ_p for complexes **Ru^{H}-NCAr^{X}**, **A^{H}-NCAr^{X}**, and **C^{H}-NCAr^{X}**. S75
Figure S116. CV data for [*trans*-$(PPh_3)_2$-*mer*-$(NCCH_3)_3$-RuCl][$BBPh_4$] and $NaBPh_4$. S76
Figure S117. CV data for *cis-fac*-**C^{H}-NCCH$_3$** S76
Table S4. Reduction potentials for electrochemically generated **C^{H}-NCAr^{X}** complexes S77
Figure S118. Hammett plots for all monophosphine complexes S77
*Bulk electrolysis of **Ru^{H}-Cl*** S78
Figure S119. CV data following bulk electrolysis. S79
Figure S120. NMR data following bulk electrolysis. S79

Elemental analysis of *cis-mer*-C^{H}-NCCH$_3$ S80
Table S5. Elemental analysis results for *cis-mer*-**C^{H}-NCCH$_3$** samples S80

X-ray crystallography S81

Table S6. Crystal and refinement data........ S81
Table S7. Crystal and refinement data (cont'd)........ S82
Evaluation of possible π-π interactions in solid-state structures of bis(phosphine) complexes........ S83
Table S8. Selected bond and angle metrics for tris(nitrile) complexes **C^{H}-L**. S83
Refinement details for Ru^{OMe}-PAr_3........ S84
Figure S121. Solid-state structure of **Ru^{OMe}-PAr_3**........ S84
Refinement details for Ru^{H}-$NCAr^{OMe}$ S85
Figure S122. Solid-state structure of **Ru^{H}-$NCAr^{OMe}$** S85
Refinement details for Ru^{H}-$NCAr^{Me}$ S86
Figure S123. Solid-state structure of **Ru^{H}-$NCAr^{Me}$** S86
Refinement details for Ru^{H}-$NCAr^{H}$........ S87
Figure S124. Solid-state structure of **Ru^{H}-$NCAr^{H}$**........ S87
Refinement details for Ru^{H}-$NCAr^{Cl}$ S88
Figure S125. Solid-state structure of **Ru^{H}-$NCAr^{Cl}$** S88
Refinement details for Ru^{H}-$NCAr^{CF3}$ S89
Figure S126. Solid-state structure of **Ru^{H}-$NCAr^{CF3}$** S89
Refinement details for *trans-mer*-C^{H}-$NCAr^{OMe}$........ S90
Figure S127. Solid-state structure of *trans-mer*-**C^{H}-$NCAr^{OMe}$** (full)........ S90
Figure S128. Solid-state structure of *trans-mer*-**C^{H}-$NCAr^{OMe}$** (Ru fragment)........ S91
Preliminary structure for *trans-mer*-C^{H}-$NCCH_3$........ S92
Figure S129. Preliminary solid-state structure of *trans-mer*-**C^{H}-$NCCH_3$** S92

References........ S93

NMR data

Characterization of new compounds

Additional characterization data; for numbering, see Figures 1 and 2 in the main text.

Ru^{OMe}-NCCH$_3$. ^{13}C{^{1}H} NMR (125 MHz, CDCl$_3$): δ 161.96 (C11), 136.11 (d, $^{2}J_{C,P}$ = 11.1 Hz, C9), 127.22 (C12), 121.55 (d, $^{1}J_{C,P}$ = 55.1 Hz, C8), 116.45 (d, $^{2}J_{C,P}$ = 7.7 Hz, C3), 114.34 (d, $^{3}J_{C,P}$ = 11.6 Hz, C10), 103.50 (C6), 95.58 (d, $^{2}J_{C,P}$ = 6.1 Hz, C4a), 89.57 (C5b), 88.84 (d, $^{2}J_{C,P}$ = 4.9 Hz, C4b), 84.69 (C5a), 55.57 (C17), 31.39 (C2), 23.62 (C1b), 21.28 (C1a), 18.48 (C7), 3.41 (C13) ppm. ^{19}F NMR (469 MHz, CDCl$_3$): δ –72.70 (d, $^{1}J_{F,P}$ = 713.2 Hz, PF_6^-) ppm.

Ru^{Cl}-NCCH$_3$. ^{13}C{^{1}H} NMR (125 MHz, CDCl$_3$): δ 138.84 (d, $^{4}J_{C,P}$ = 3.0 Hz, C11), 135.79 (d, $^{2}J_{C,P}$ = 11.0 Hz, C9 or C10), 129.50 (d, $^{2}J_{C,P}$ = 11.1 Hz, C10 or C9), 127.86 (d, $^{1}J_{C,P}$ = 50.4 Hz, C8), 127.62 (C12) 117.15 (d, $^{2}J_{C,P}$ = 7.9 Hz, C3), 103.68 (C6), 96.12 (C4a), 89.96 (C4b), 89.72 (C5b), 84.59 (C5a), 31.53 (C2), 23.65 (C1b), 21.17 (C1a), 18.63 (C7), 3.51 (C13) ppm. ^{19}F NMR (469 MHz, CDCl$_3$): δ –72.42 (d, $^{1}J_{F,P}$ = 713.2 Hz, PF_6^-) ppm.

Ru^{OMe}-PAr$_3$. ^{13}C{^{1}H} NMR (125 MHz, CDCl$_3$): δ 161.14 (C11), 135.69 (vt, J = 5.2 Hz, C9), 130.48 (C3), 125.28 (vt, J = 26.5 Hz, C8), 113.64 (vt, J = 5.5 Hz, C10), 100.44 (C6), 97.10 (C5), 89.20 (t, $^{2}J_{C,P}$ = 4.9 Hz, C4), 55.34 (C17), 31.31 (C2), 21.51 (C1), 15.87 (C7) ppm. ^{19}F NMR (469 MHz, CDCl$_3$): δ –73.12 (d, $^{1}J_{F,P}$ = 713.1 Hz, PF_6^-) ppm.

Ru^{Cl}-PAr$_3$. ^{13}C{^{1}H} NMR (125 MHz, CDCl$_3$): δ 138.48 (C11), 135.30 (vt, J = 5.1 Hz, C9 or C10), 132.61 (C3), 131.39 (vt, J = 24.6 Hz, C8), 129.08 (vt, J = 5.3 Hz, C10 or C9), 101.28 (C6), 97.82 (C4 or C5), 89.85 (t, $^{2}J_{C,P}$ = 4.6 Hz, C5 or C4), 31.87 (C2), 21.48 (C1), 15.65 (C7) ppm. ^{19}F NMR (469 MHz, CDCl$_3$): δ –72.49 (d, $^{1}J_{F,P}$ = 713.4 Hz, PF_6^-) ppm.

Ru^{H}-NCAr^{OMe}. ^{13}C{^{1}H} NMR (125 MHz, CDCl$_3$): δ 164.40 (C16), 135.70 (C15), 134.59 (d, $^{2}J_{C,P}$ = 9.7 Hz, C9), 131.50 (d, $^{4}J_{C,P}$ = 2.8 Hz, C11), 130.30 (d, $^{1}J_{C,P}$ = 49.2 Hz, C8), 128.94 (d, $^{3}J_{C,P}$ = 10.5 Hz, C10), 128.24 (C12 or C13), 116.39 (d, $^{2}J_{C,P}$ = 7.5 Hz, C3), 114.77 (C14), 104.20 (C6), 100.74 (C13 or C12), 96.25 (d, $^{2}J_{C,P}$ = 5.9 Hz, C4a), 90.10 (C5b), 89.85 (C4b), 85.53 (C5a), 55.83 (C17), 31.65 (C2), 23.39 (C1b), 21.50 (C1a), 18.55 (C7) ppm. ^{19}F NMR (469 MHz, CDCl$_3$): δ –72.37 (d, $^{1}J_{F,P}$ = 712.6 Hz, PF_6^-) ppm.

Ru^{H}-NCAr^{Me}. ^{13}C{^{1}H} NMR (125 MHz, CDCl$_3$): δ 147.28 (C16), 134.71 (d, $J_{C,P}$ = 9.8 Hz, C9 or C10), 133.42 (C14), 132.07 (d, $^{4}J_{C,P}$ = 2.6 Hz, C11), 131.13 (d, $^{1}J_{C,P}$ = 49.3 Hz, C8), 130.48 (C15), 129.41 (d, J = 10.5 Hz, C10 or C9), 128.64 (C12 or C13), 115.12 (d, $^{2}J_{C,P}$ = 5.0 Hz, C6), 106.63 (C13 or C12), 103.89 (C3), 94.42 (d, $^{2}J_{C,P}$ = 6.1 Hz, C4b), 90.87 (d, $^{2}J_{C,P}$ = 2.3 Hz, C5b), 90.24 (d, $^{2}J_{C,P}$ = 1.9 Hz, C4a), 89.88 (C5a), 32.13 (C2), 23.37 (C1b), 22.41 (C17), 21.81 (C1a), 18.72 (C7) ppm. ^{19}F NMR (376 MHz, CDCl$_3$): δ –73.72 (d, $^{1}J_{F,P}$ = 710.6 Hz, PF_6^-) ppm.

Ru^{H}-NCAr^{H}. ^{13}C{^{1}H} NMR (101 MHz, CDCl$_3$): δ 135.47 (C16), 134.74 (d, $J_{C,P}$ = 9.8 Hz, C9 or C10), 133.58 (d, $^{5}J_{C,P}$= 0.7 Hz, C14), 132.10 (d, $^{4}J_{C,P}$ = 2.7 Hz, C11), 131.04 (d, $^{1}J_{C,P}$ = 49.4 Hz, C8), 129.73 (C15), 129.44 (d, $J_{C,P}$ = 10.5 Hz, C10 or C9), 128.20 (C13 or C12), 115.31 (d, $^{2}J_{C,P}$ = 5.0 Hz, C3), 109.86 (d, $^{2}J_{C,P}$ = 0.8 Hz, C12 or C13), 104.14 (s, C6), 94.69 (d, $^{2}J_{C,P}$ = 6.0 Hz, C5b), 90.58 (d, $^{2}J_{C,P}$ = 2.2 Hz, C4b), 90.34 (d, $^{2}J_{C,P}$ = 1.9 Hz, C5a) 89.96 (d, $^{2}J_{C,P}$ = 1.0 Hz, C4a), 32.16 (C2), 23.43 (C1b), 21.80 (C1a), 18.75 (C7) ppm. ^{19}F NMR (376 MHz, CDCl$_3$): δ –72.84 (d, $^{1}J_{F,P}$ = 710.2 Hz, PF_6^-) ppm.

Ru^{H}-NCAr^{Cl}. ^{13}C{^{1}H} NMR (125 MHz, CDCl$_3$): δ 141.63 (C16 or C13), 134.85 (C14), 134.66 (d, $^{2}J_{C,P}$ = 9.9 Hz, C9), 131.58 (d, $^{4}J_{C,P}$ = 2.8 Hz, C11), 130.04 (d, $^{1}J_{C,P}$ = 49.8 Hz, C8), 129.56 (C15), 128.98 (d, $^{3}J_{C,P}$ = 10.5 Hz, C10), 126.73 (C12), 116.82 (d, $^{2}J_{C,P}$ = 7.9 Hz, C3), 107.81 (C13 or C16), 104.90 (C6), 96.99 (d, $^{2}J_{C,P}$ = 6.1 Hz, C4a), 90.22 (C4b), 90.09 (C5b), 84.83 (C5a), 31.66 (C2), 23.51 (d, $^{3}J_{C,P}$ = 2.1 Hz, C1b), 21.44 (C1a), 18.56 (C7) ppm. ^{19}F NMR (469 MHz, CDCl$_3$): δ –72.15 (d, $^{1}J_{F,P}$ = 712.9 Hz, PF_6^-) ppm.

Ru^{H}-NCAr^{CF3}. ^{13}C{^{1}H} NMR (125 MHz, CDCl$_3$): δ 135.81 (q, $^{2}J_{C,F}$ = 33.5 Hz, C16), 134.63 (d, $^{2}J_{C,P}$ = 9.9 Hz, C9), 134.21 (C14), 131.61 (d, $^{4}J_{C,P}$ = 2.7 Hz, C11), 129.95 (d, $^{1}J_{C,P}$ = 49.6 Hz, C8), 129.00 (d, $^{3}J_{C,P}$ = 10.6 Hz, C10), 126.03 (C12), 125.90 (q, $^{3}J_{C,F}$ = 3.7 Hz, C15), 122.95 (q, $^{1}J_{C,F}$ = 273.3 Hz, C17) , 116.81 (d, $^{2}J_{C,P}$ = 7.8 Hz, C6), 113.13 (C13), 105.28 (C3), 97.10 (d, $^{2}J_{C,P}$ = 5.8 Hz, C4a), 90.25 (C4b), 90.14 (C5b), 85.20 (C5a), 31.65 (C2), 23.48 (d, $^{3}J_{C,P}$ = 2.1 Hz, C1b), 21.41 (C1a), 18.52 (C7) ppm. ^{19}F NMR (469 MHz, CDCl$_3$): δ –63.72 (s, C17–F), –72.06 (d, $^{1}J_{F,P}$ = 713.1 Hz, PF_6^-) ppm.

trans-mer-**C^{H}-NCCH$_3$.** $^{13}C\{^{1}H\}$ NMR (125 MHz, CD_3CN): δ 135.11 (vt, $^{2}J_{C,P}$ = 5.5 Hz, C2), 132.74 (vt, $^{1}J_{C,P}$ = 20.5 Hz, C1), 131.07 (C4 or C3), 129.17 (C3 or C4), 127.85 (C5), 125.85 (C7), 4.18 (C6), 3.65 (C8) ppm. ^{19}F NMR (469 MHz, CD_3CN): δ –73.79 (d, $^{1}J_{F,P}$ = 706.5 Hz, PF_6^-) ppm.

trans-mer-**C^{H}-NCAr^{OMe}.** $^{13}C\{^{1}H\}$ NMR (101 MHz, $CDCl_3$): δ 163.86 (C9), 163.58 (C15), 134.61 (C14), 134.53 (C7 or C8), 134.25 ($^{2}J_{C,P}$ = 5.3 Hz, C2), 131.51 (vt, $^{1}J_{C,P}$ = 21.0 Hz, C1), 130.25 (C4), 128.70 (vt, $^{2}J_{C,P}$ = 4.7 Hz, C3), 127.82 (C5 or C11), 126.77 (C11 or C5), 115.07 (C8 or C7), 114.73 (C13), 102.30 (C6), 101.97 (C12), 56.05 (C10 or C16), 55.94 (C16 or C10) ppm. ^{19}F NMR (376 MHz, $CDCl_3$): δ –153.76 (s, $^{10}BF_4^-$), –153.82 (s, $^{11}BF_4^-$) ppm.

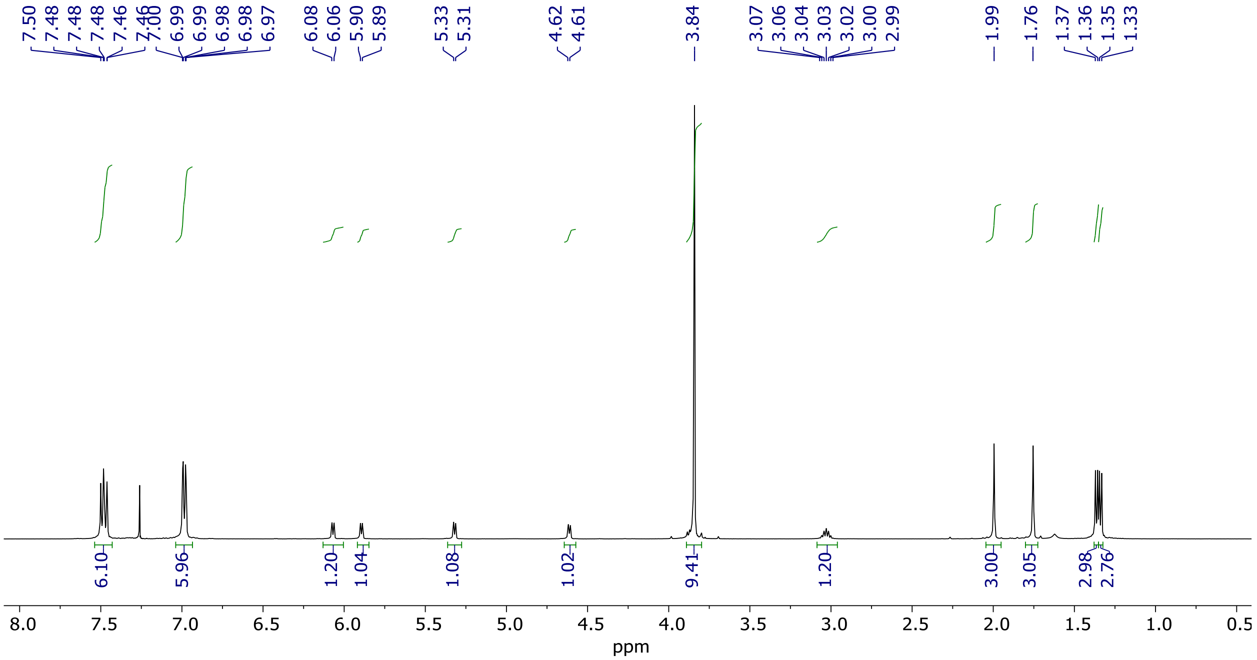

Figure S1. ^{1}H NMR (500 MHz, $CDCl_3$) of **Ru^{OMe}-NCCH$_3$.**

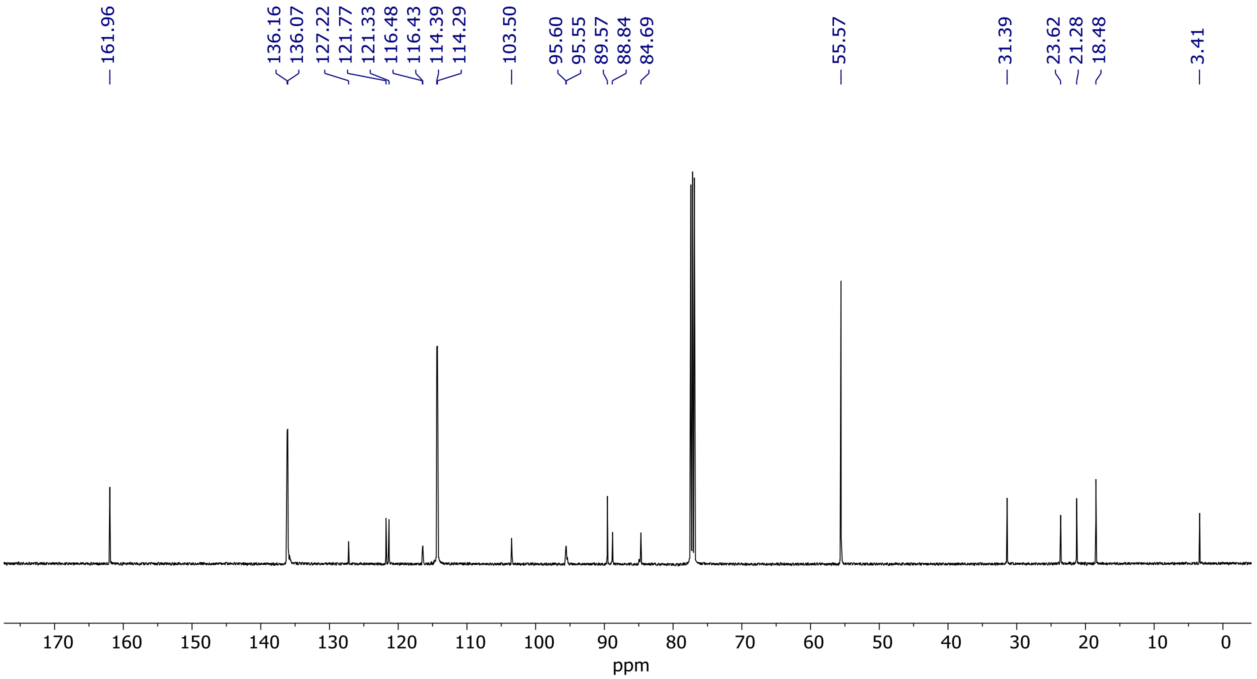

Figure S2. $^{13}C\{^{1}H\}$ NMR spectrum (125 MHz, $CDCl_3$) of **Ru^{OMe}-NCCH$_3$**.

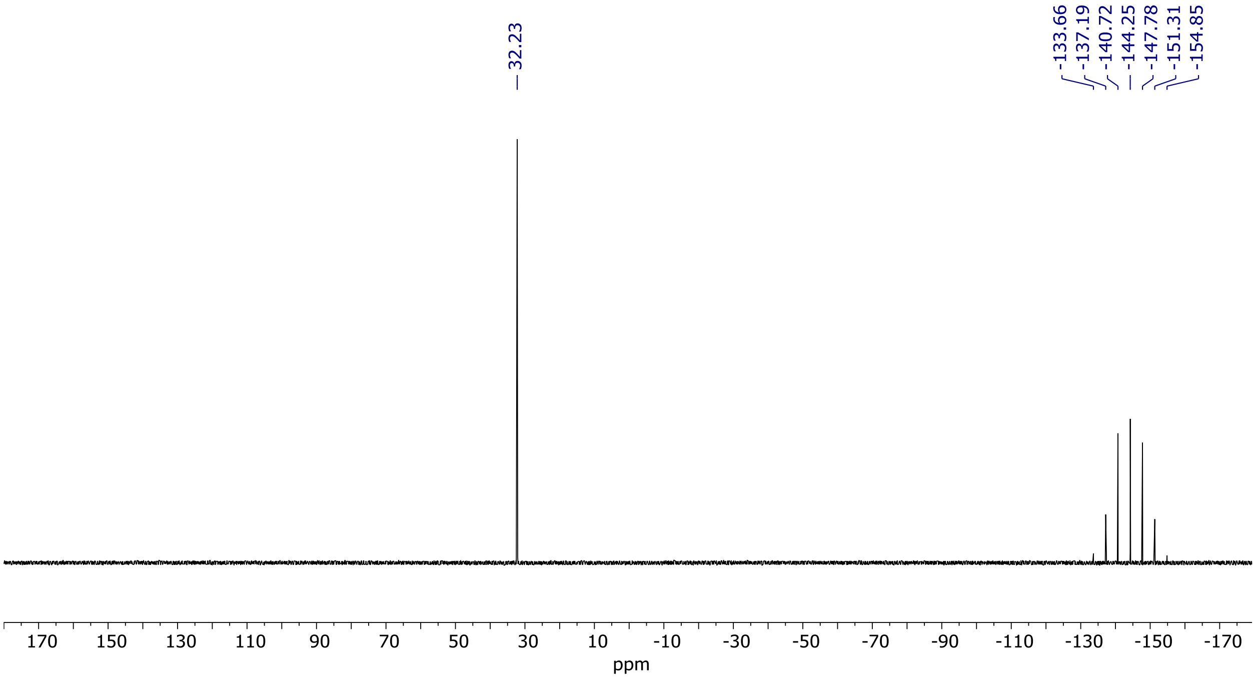

Figure S3. $^{31}P\{^{1}H\}$ NMR spectrum (202 MHz, $CDCl_3$) of **Ru^{OMe}-NCCH$_3$**.

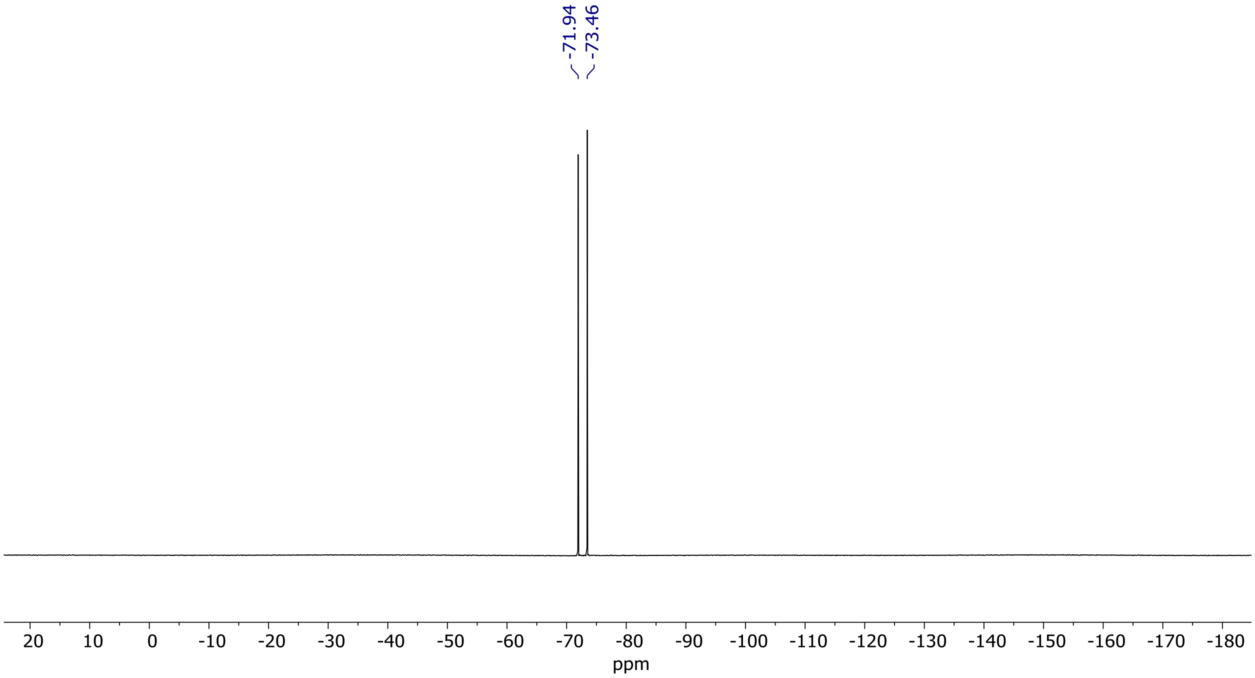

Figure S4. ^{19}F NMR spectrum (469 MHz, $CDCl_3$) of **Ru^{OMe}-NCCH$_3$**.

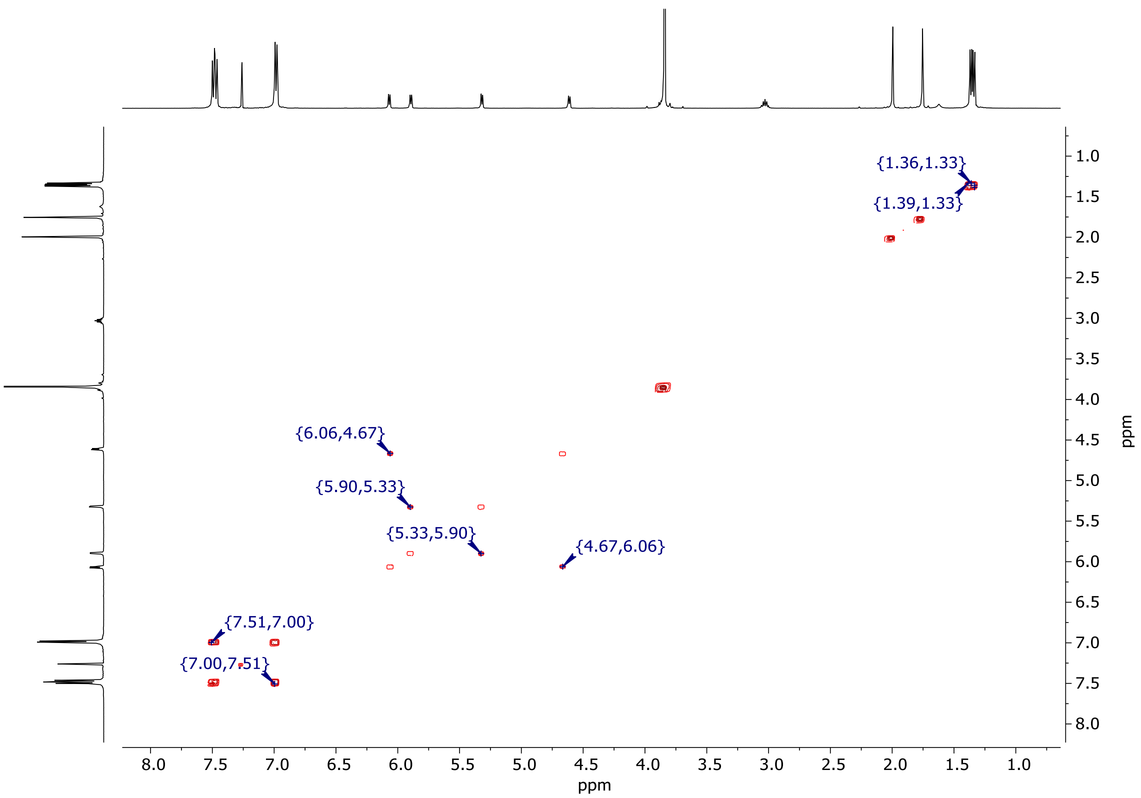

Figure S5. COSY spectrum (500 MHz, $CDCl_3$) of **Ru^{OMe}-NCCH$_3$**.

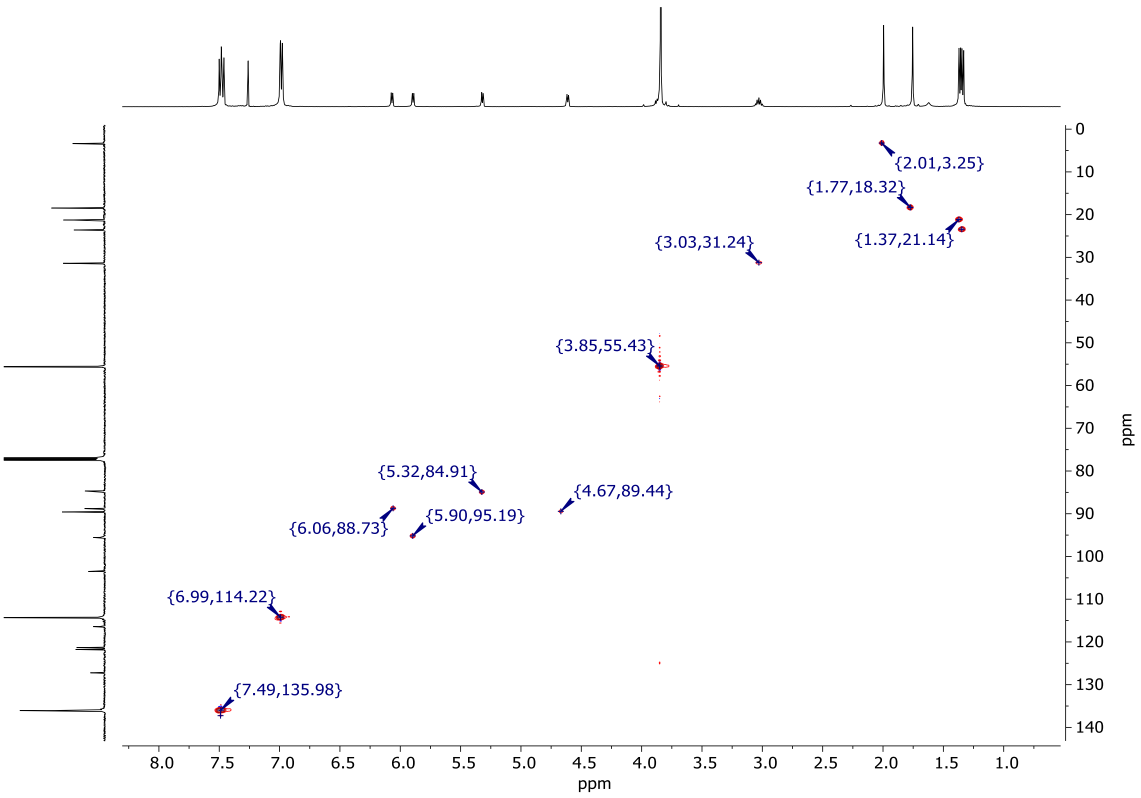

Figure S6. HSQC spectrum (500 MHz, $CDCl_3$) of **2-OMe**.

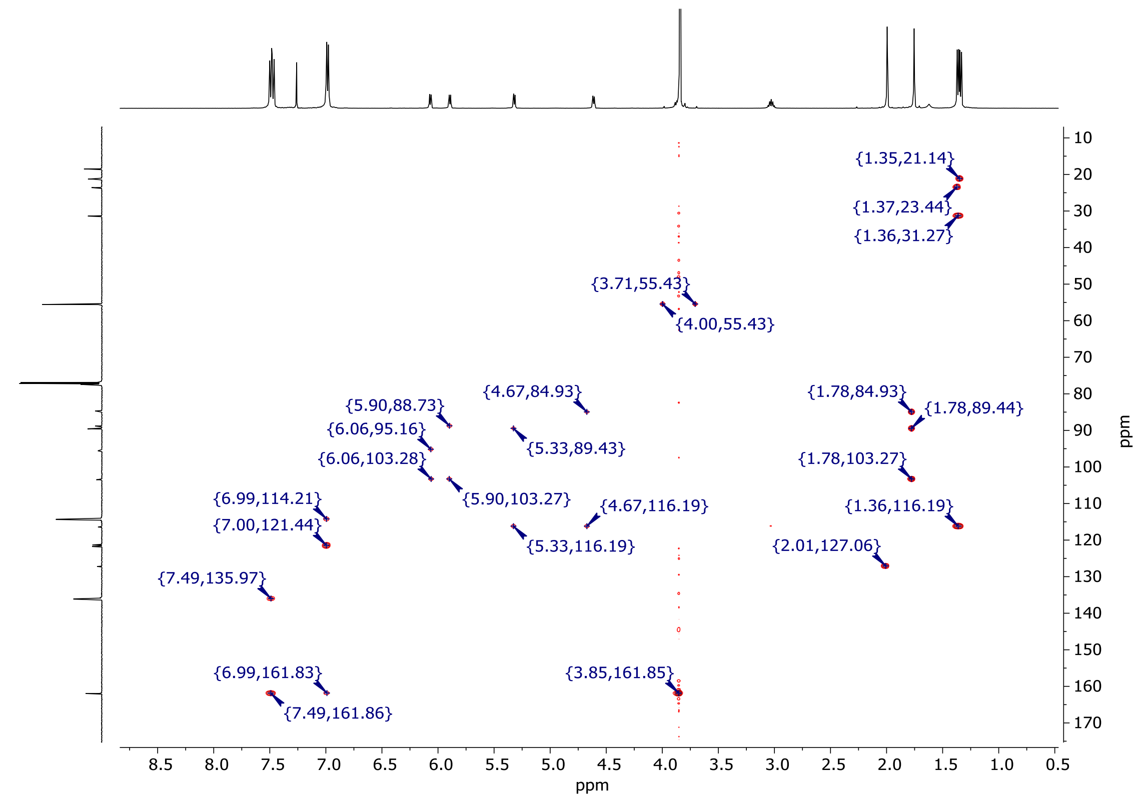

Figure S7. HMBC spectrum (500MHz, $CDCl_3$) of **Ru^{OMe}-NCCH$_3$**.

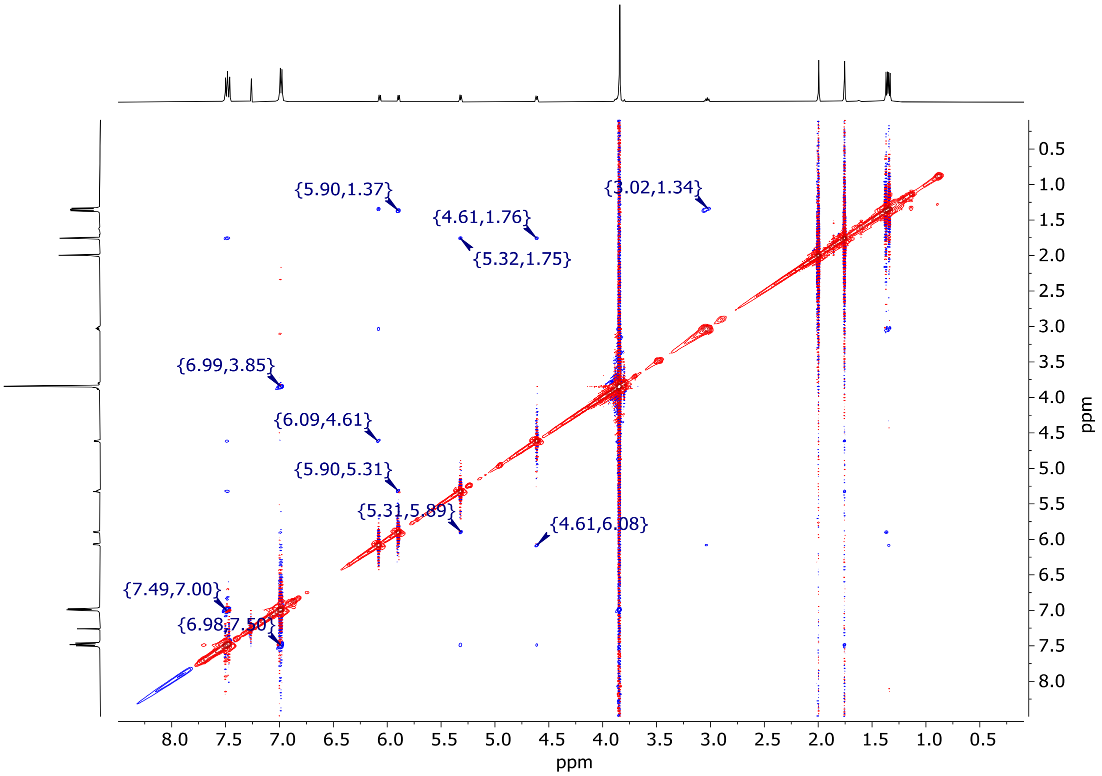

Figure S8. NOESY spectrum (500MHz, $CDCl_3$) of **Ru^{OMe}-NCCH$_3$**.

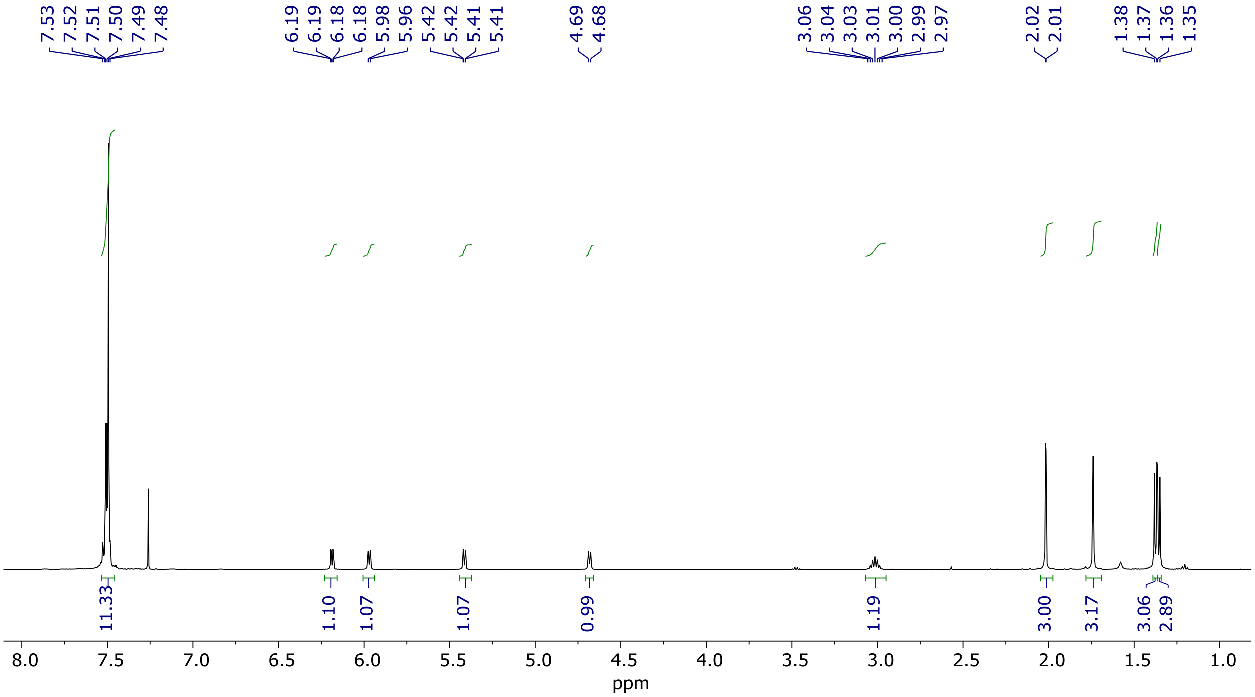

Figure S9. ^{1}H NMR (500 MHz, $CDCl_3$) of **Ru^{Cl}-NCCH$_3$.**

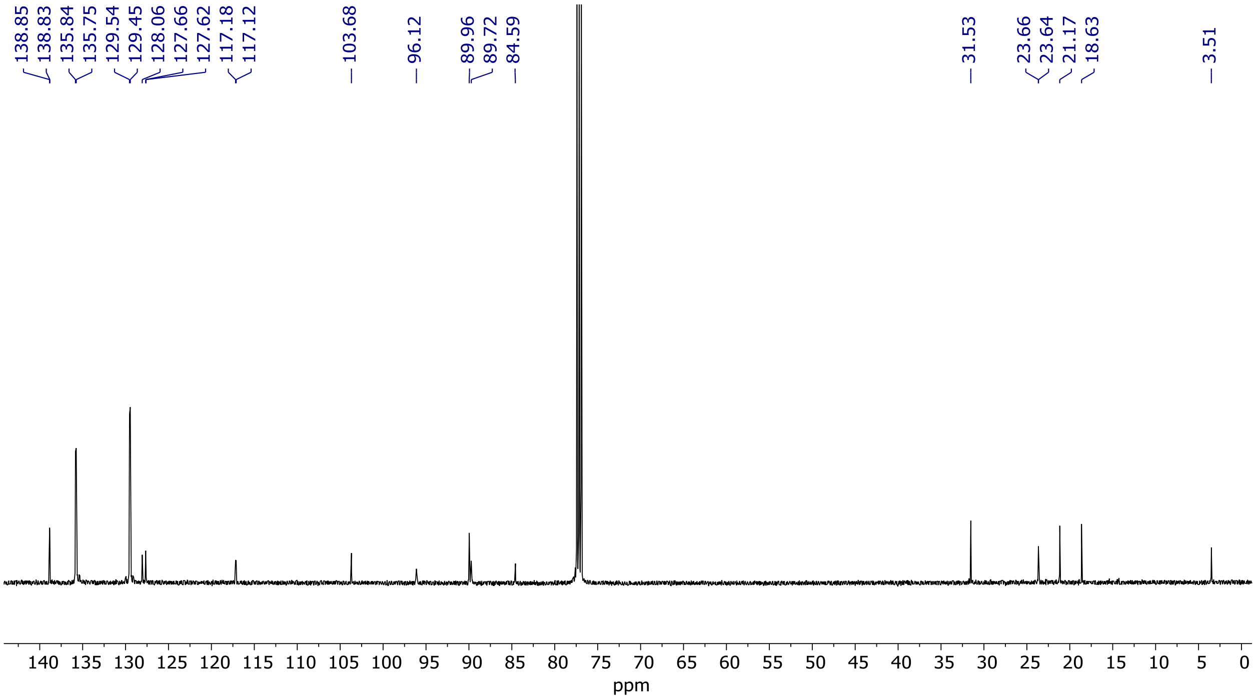

Figure S10. $^{13}C\{^{1}H\}$ NMR spectrum (125 MHz, $CDCl_3$) of **Ru^{Cl}-NCCH$_3$**.

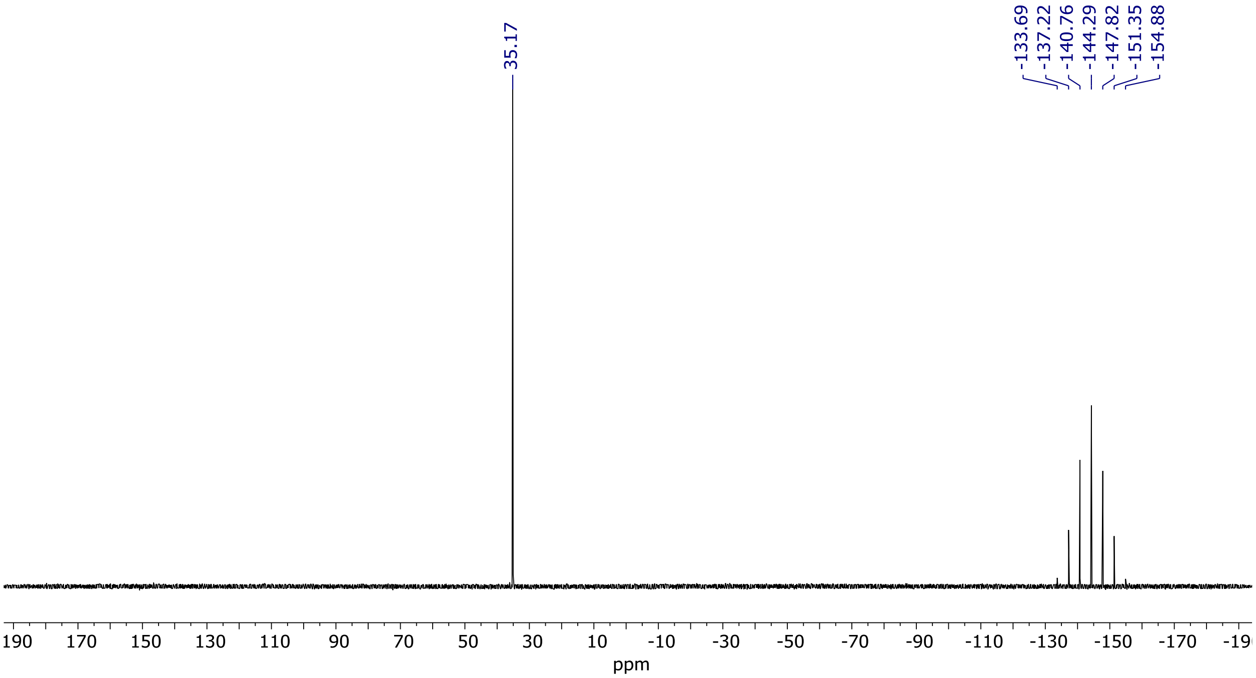

Figure S11. $^{31}P\{^{1}H\}$ NMR (202 MHz, $CDCl_3$) of **Ru^{Cl}-NCCH$_3$**.

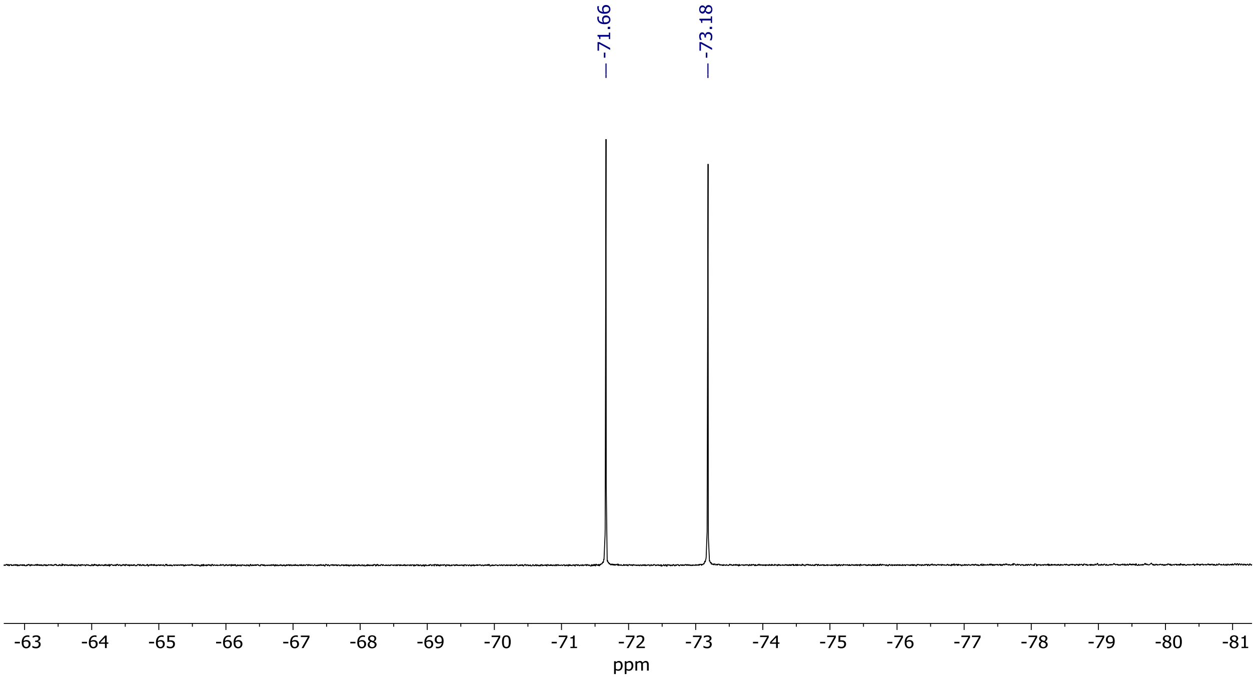

Figure S12. ^{19}F NMR (469 MHz, $CDCl_3$) of **Ru^{Cl}-NCCH$_3$**.

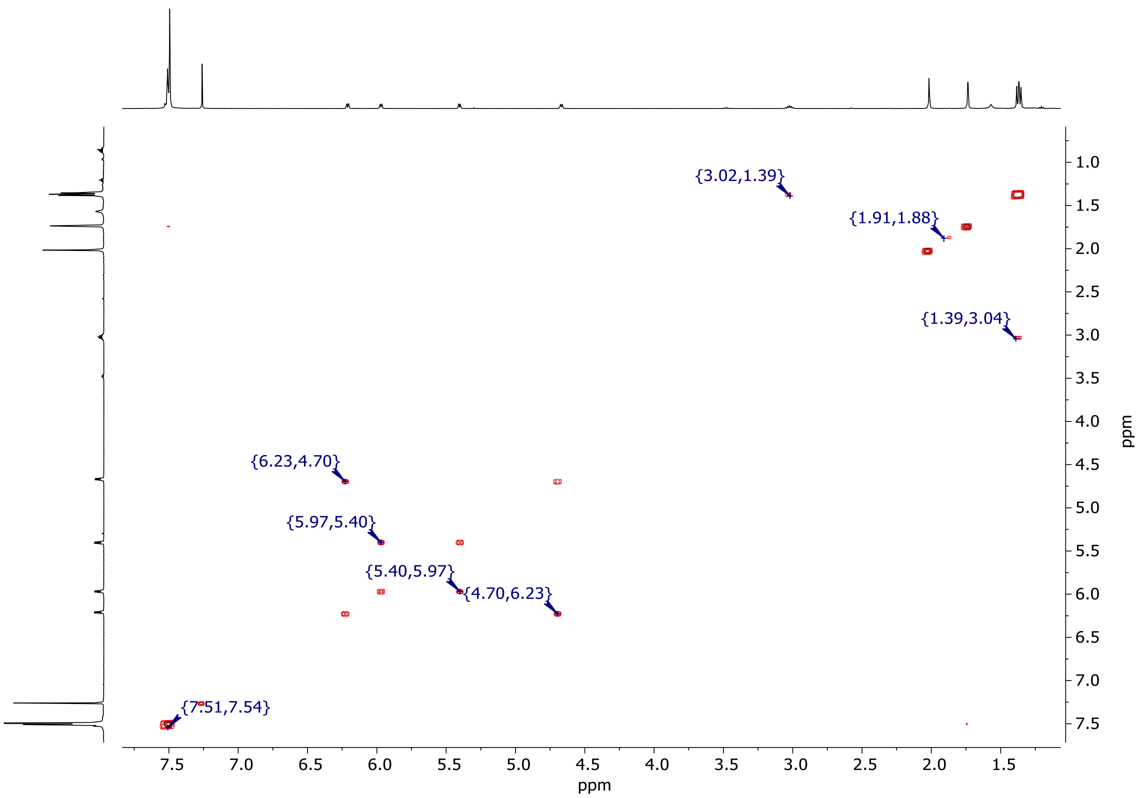

Figure S13. COSY spectrum (500 MHz, $CDCl_3$) of **Ru^{Cl}-NCCH$_3$**.

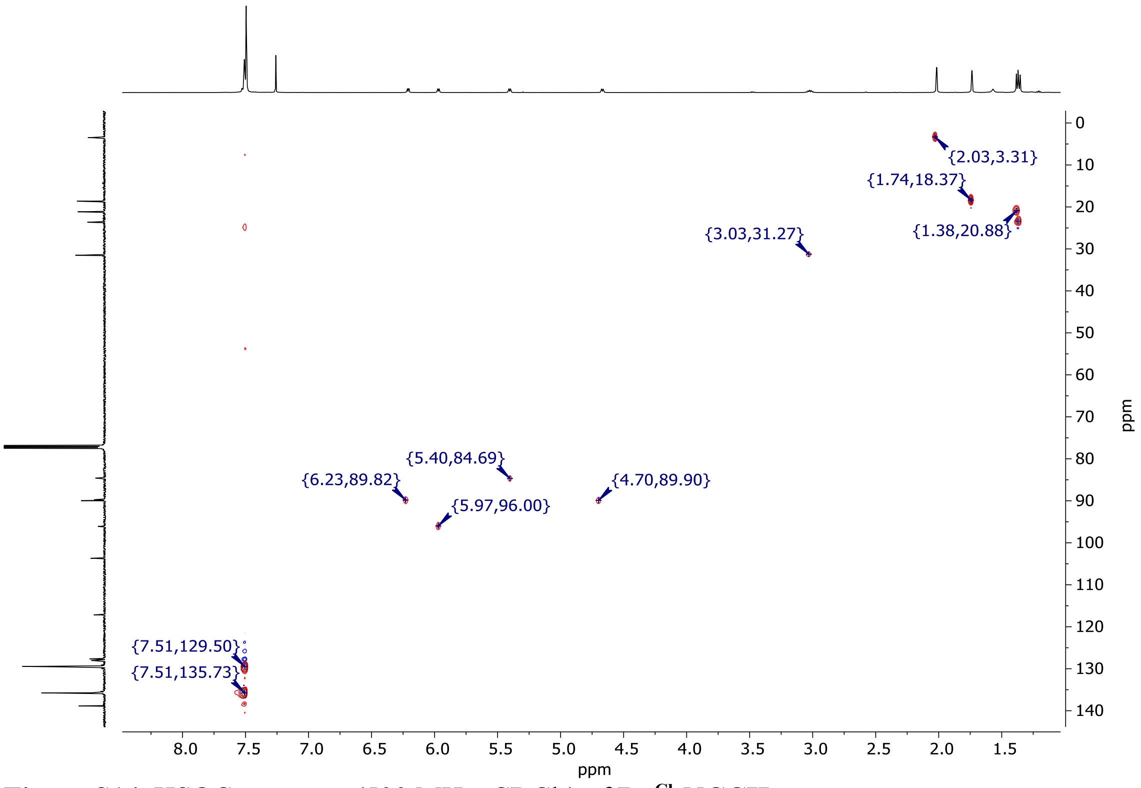

Figure S14. HSQC spectrum (500 MHz, $CDCl_3$) of **Ru^{Cl}-NCCH$_3$**.

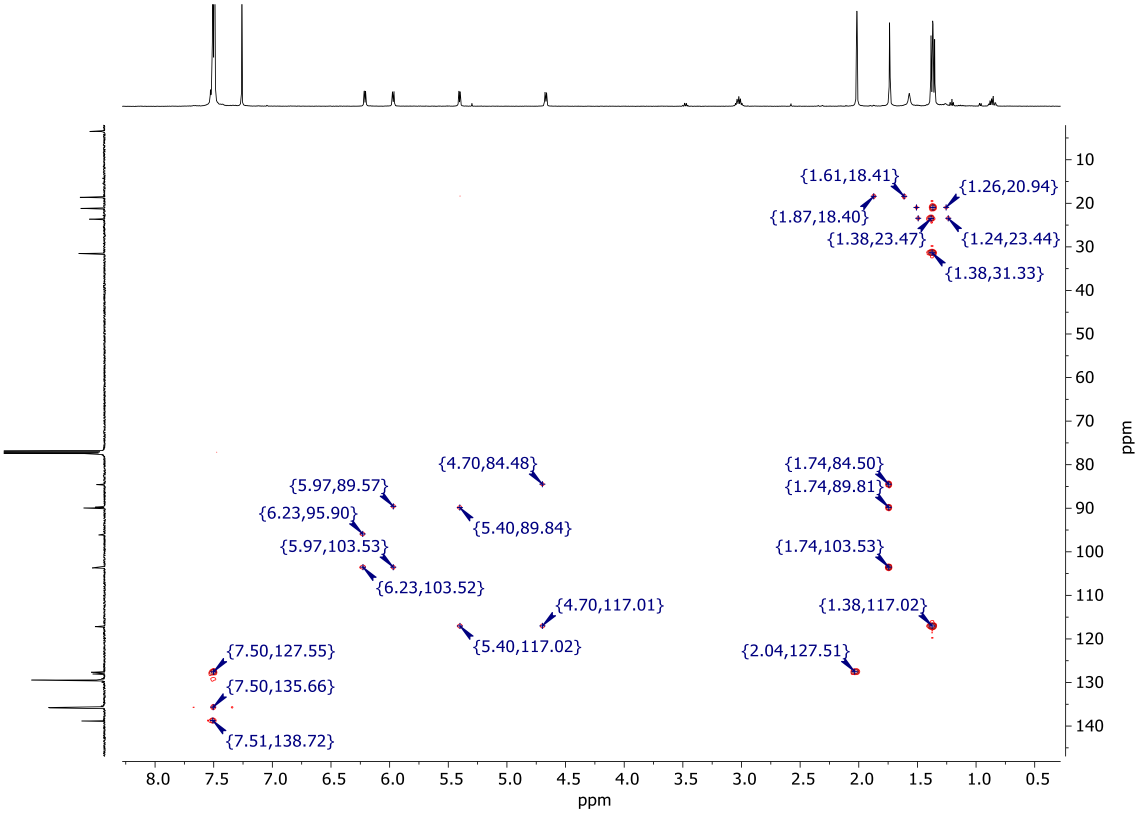

Figure S15. HMBC spectrum (500MHz, $CDCl_3$) of **Ru^{Cl}-NCCH$_3$**.

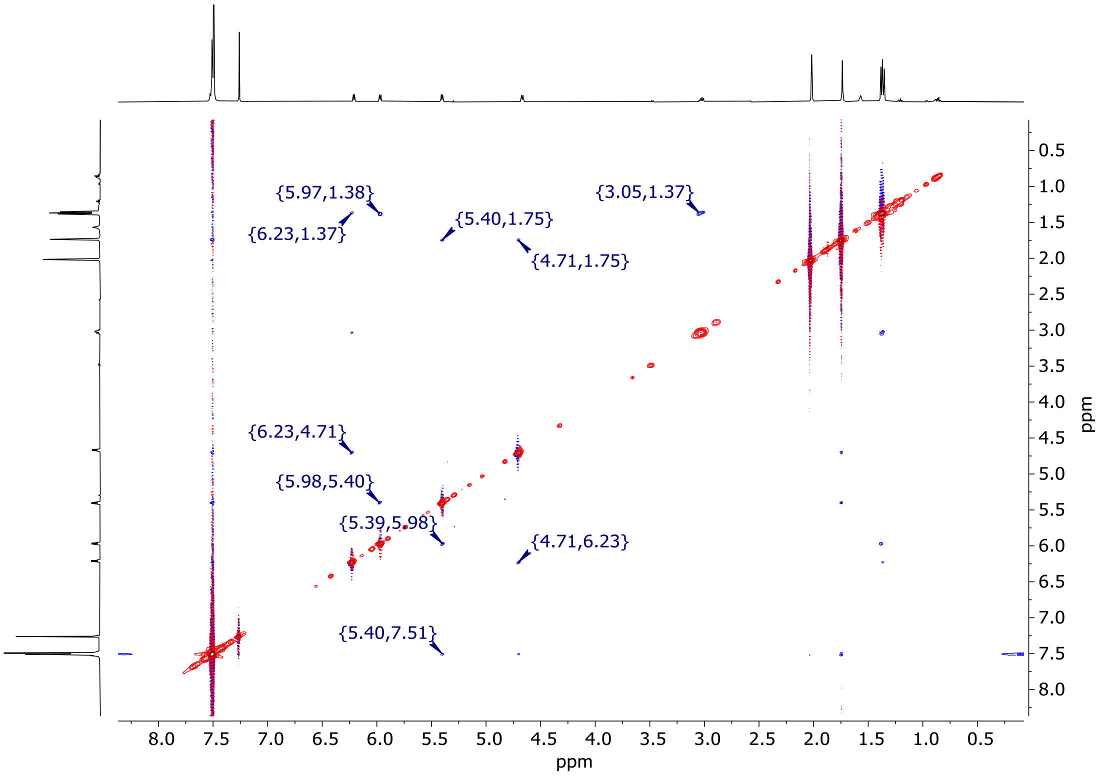

Figure S16. NOESY spectrum (500MHz, $CDCl_3$) of **Ru^{Cl}-NCCH$_3$**.

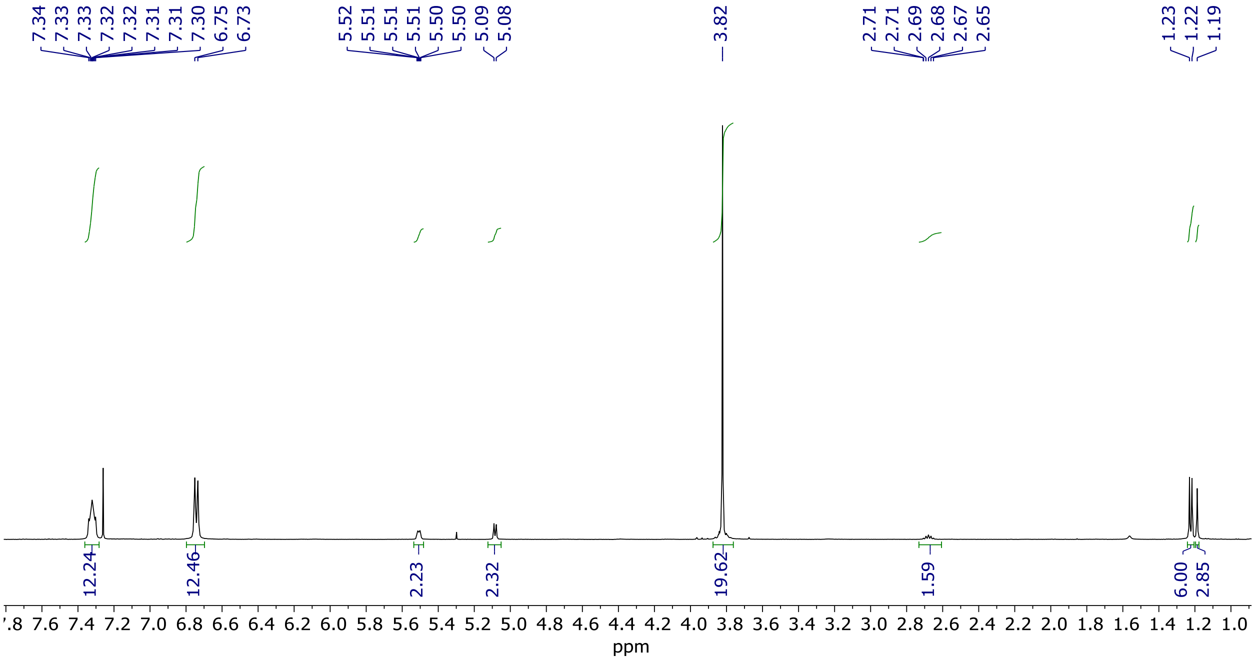

Figure S17. ^{1}H NMR (500 MHz, $CDCl_3$) of **Ru^{OMe}-PAr$_3$.**

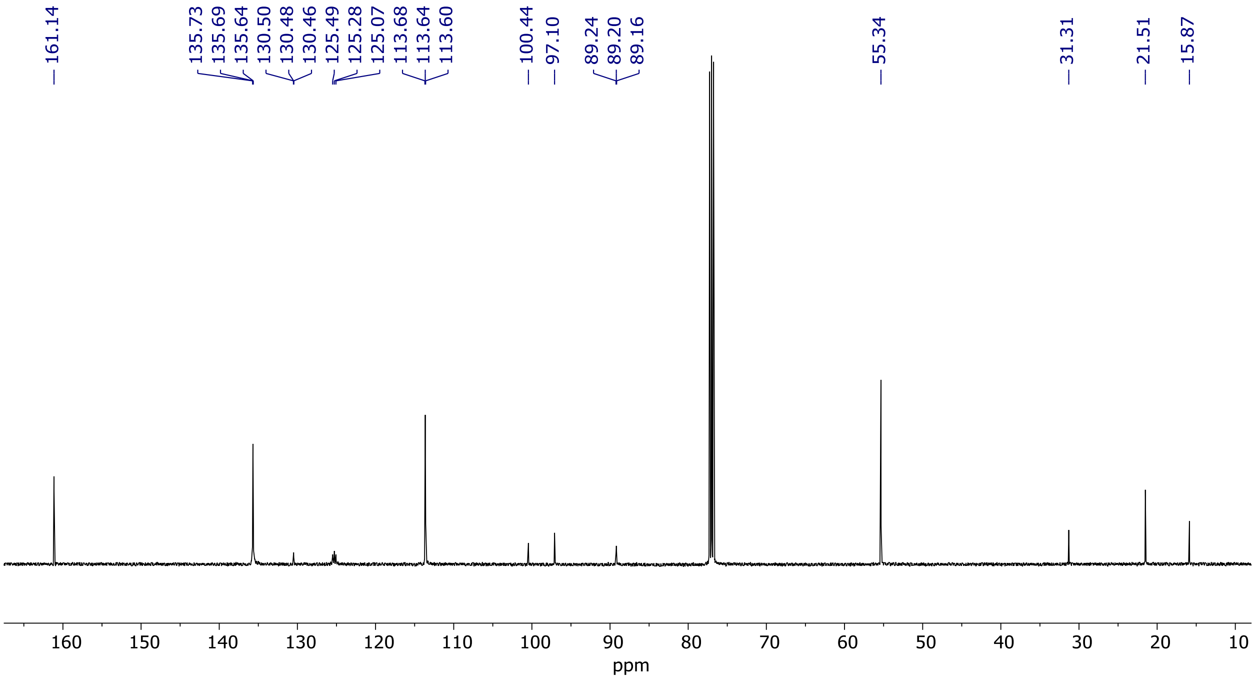

Figure S18. $^{13}C\{^{1}H\}$ NMR spectrum (125 MHz, $CDCl_3$) of **Ru^{OMe}-PAr$_3$**.

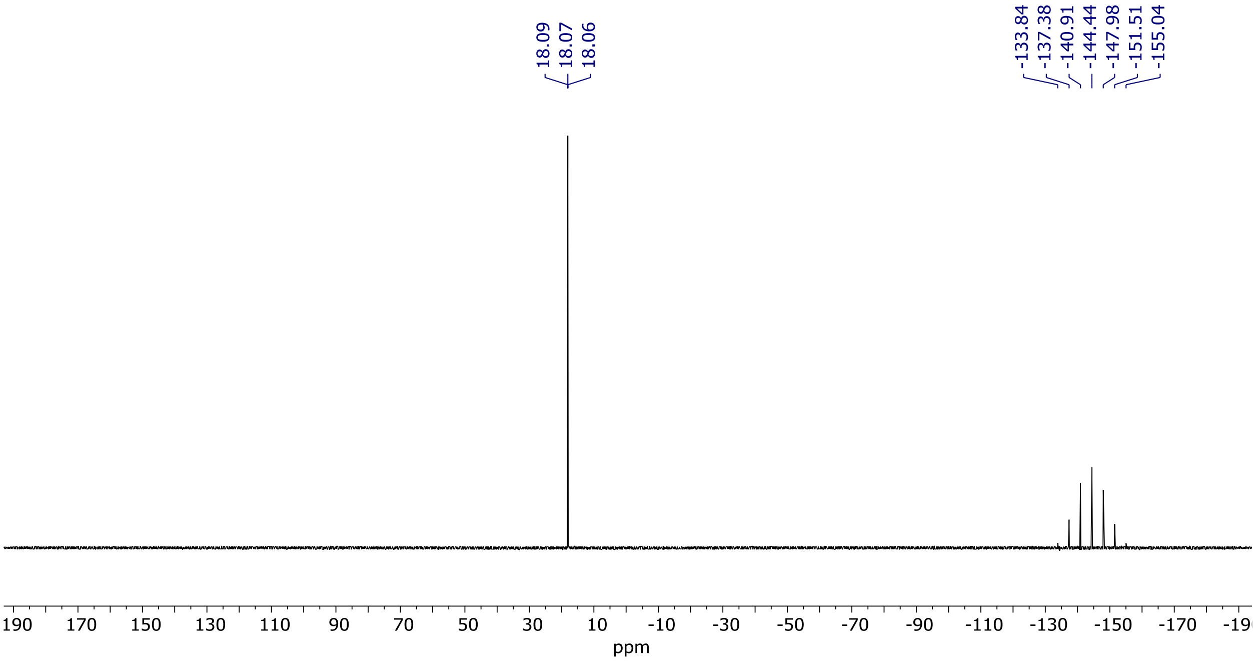

Figure S19. $^{31}P\{^{1}H\}$ NMR (202 MHz, $CDCl_3$) of **Ru^{OMe}-PAr$_3$**.

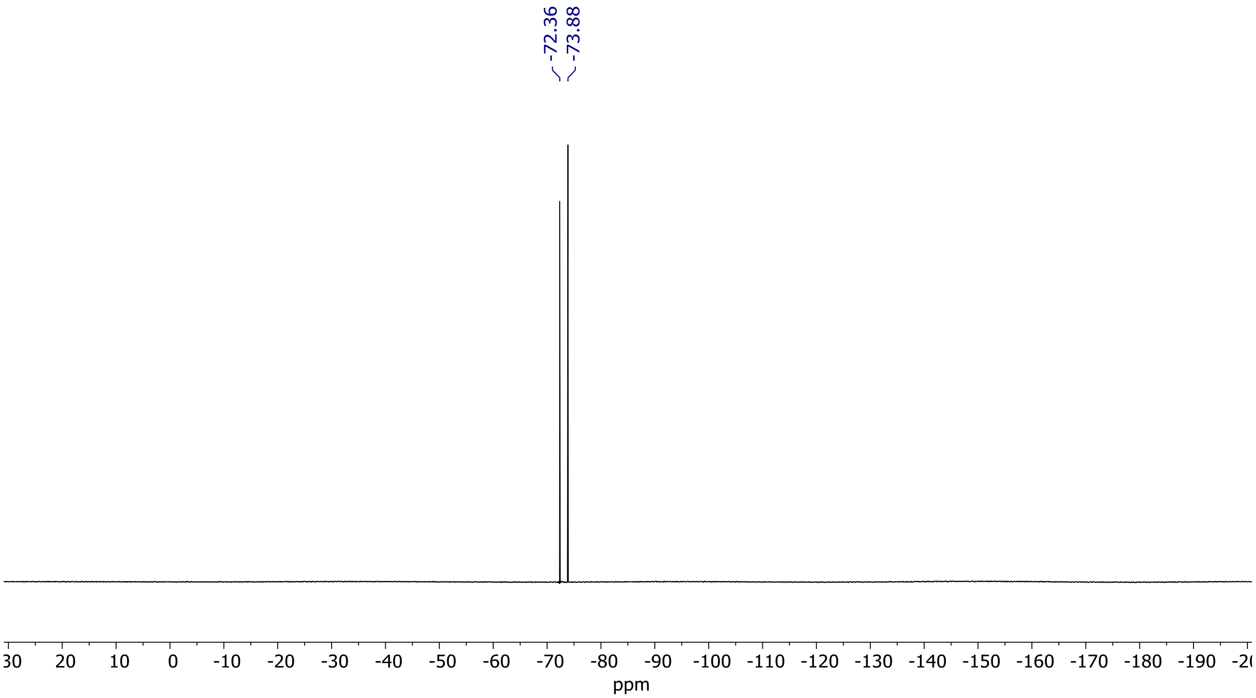

Figure S20. ^{19}F NMR (469 MHz, $CDCl_3$) of **Ru^{OMe}-PAr$_3$**.

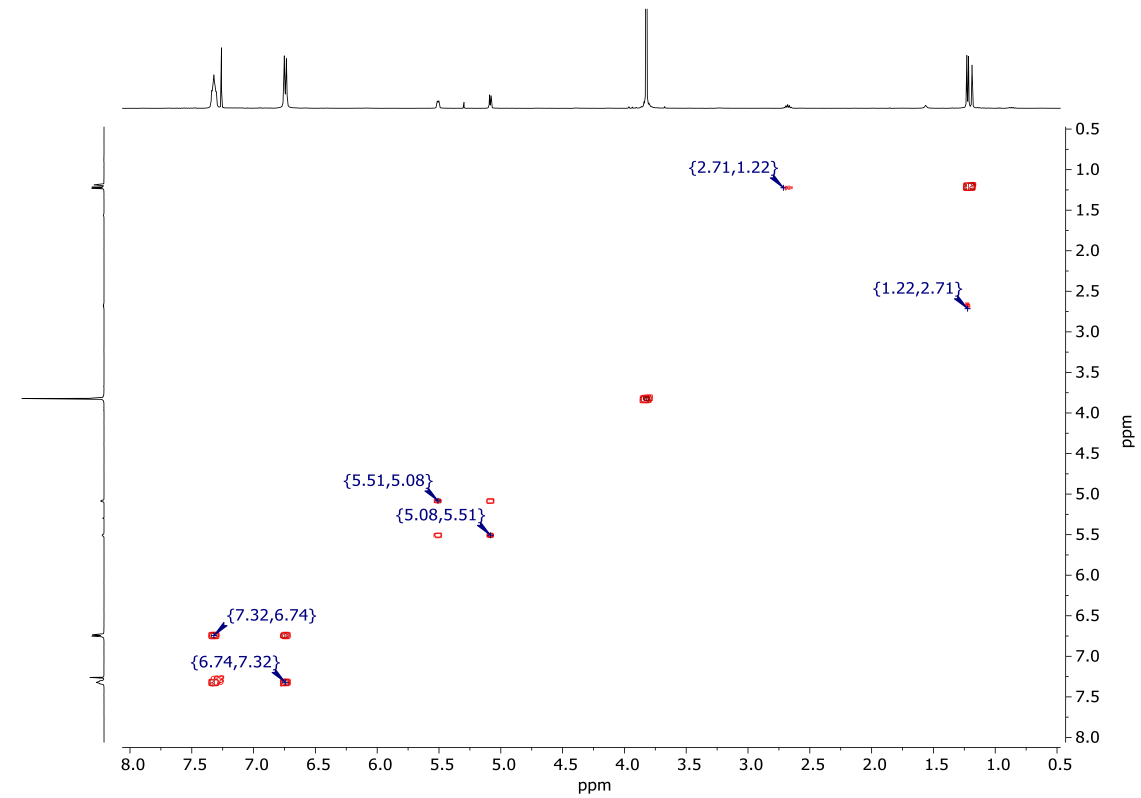

Figure S21. COSY spectrum (500 MHz, $CDCl_3$) of **Ru^{OMe}-PAr$_3$**.

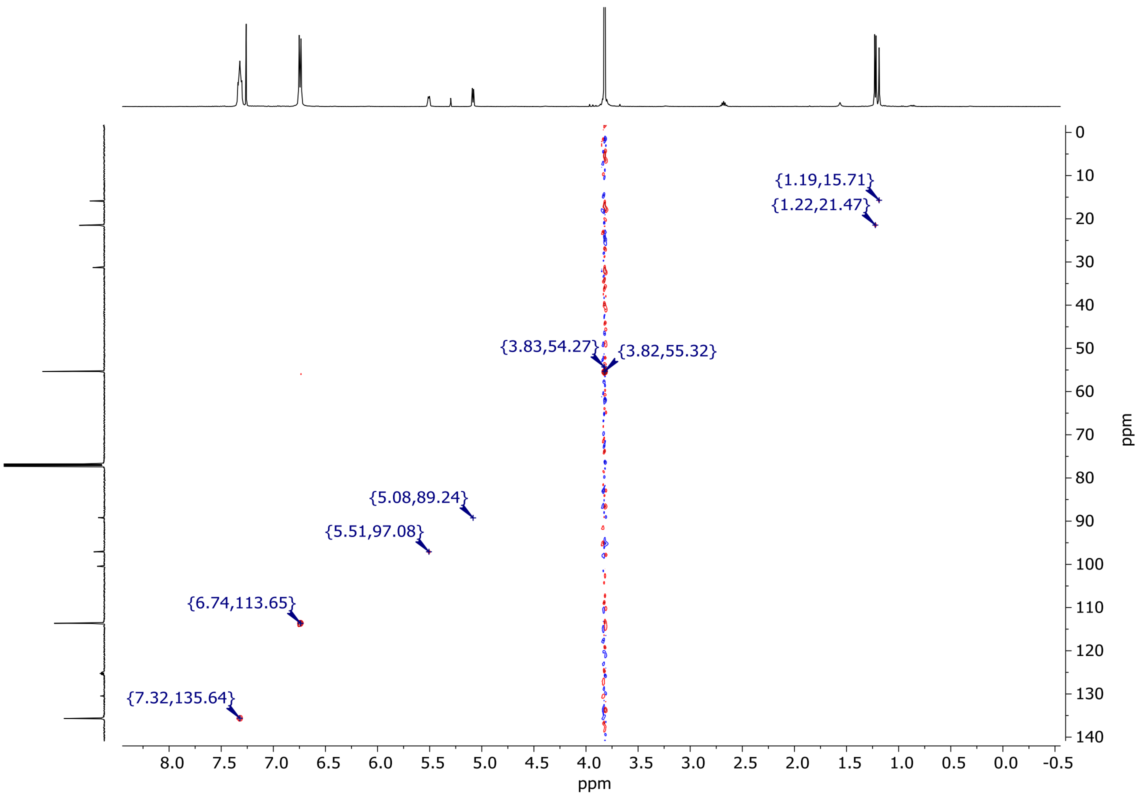

Figure S22. HSQC spectrum (500 MHz, $CDCl_3$) of **Ru^{OMe}-PAr$_3$**.

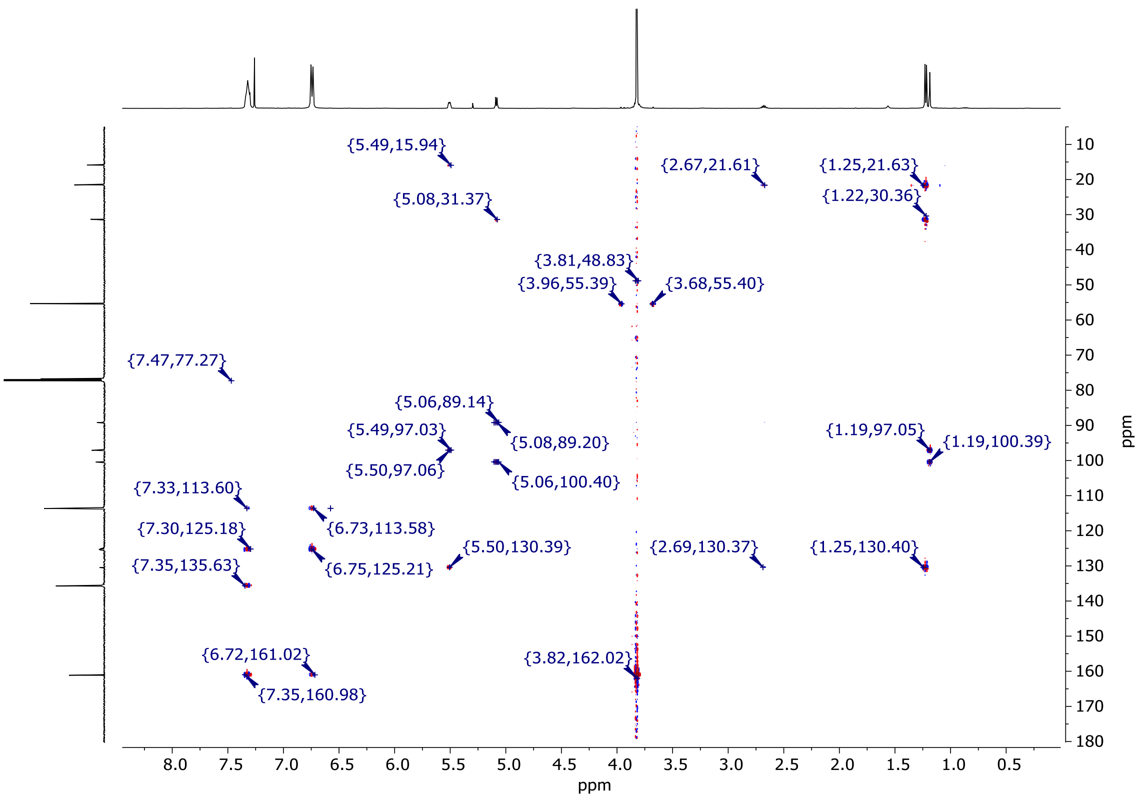

Figure S23. HMBC spectrum (500MHz, $CDCl_3$) of **Ru^{OMe}-PAr$_3$**.

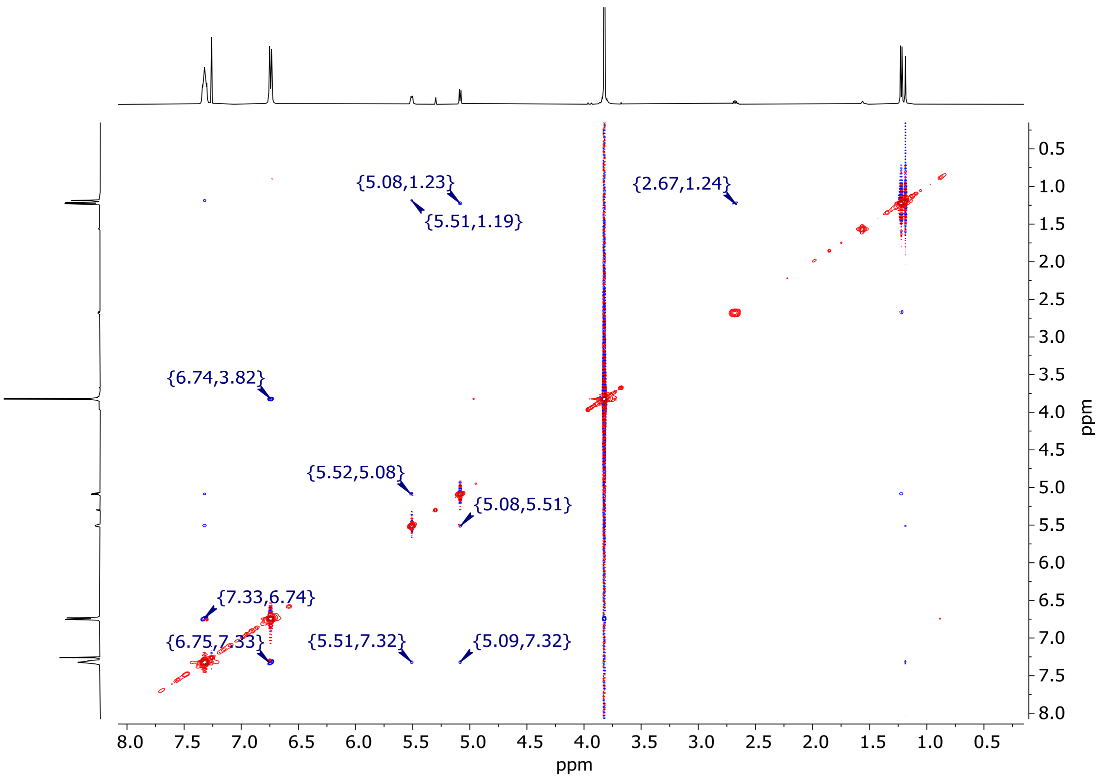

Figure S24. NOESY spectrum (500MHz, $CDCl_3$) of **Ru^{OMe}-PAr$_3$**.

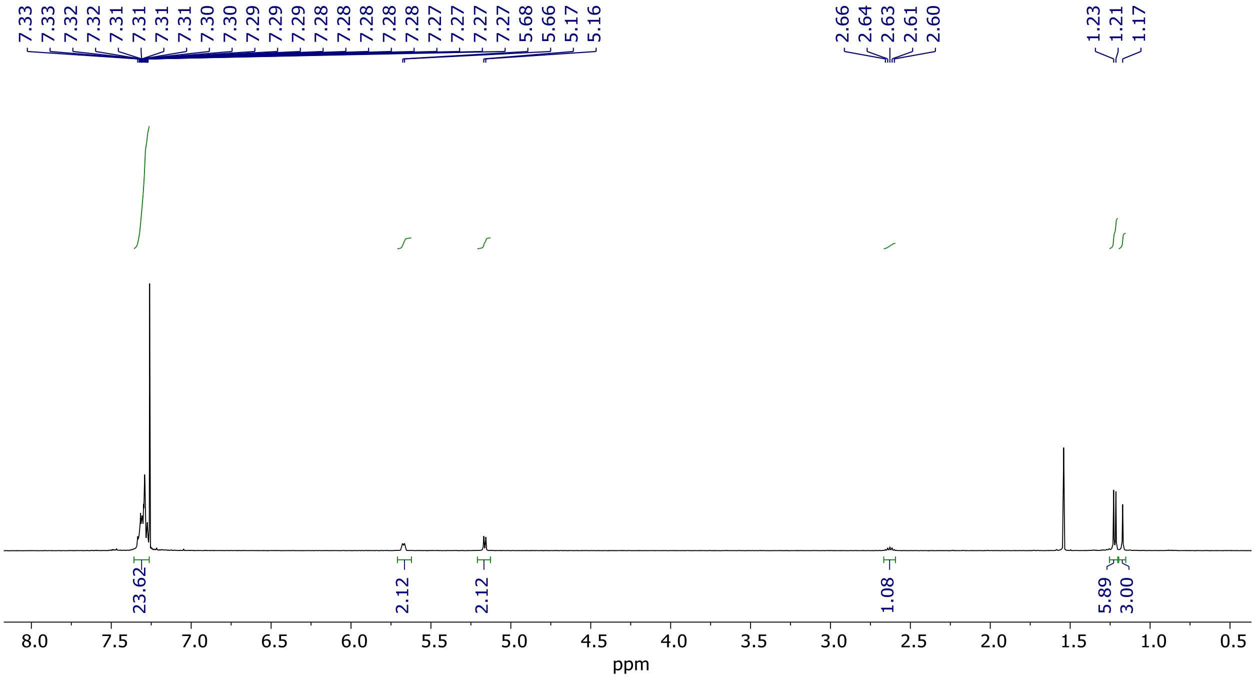

Figure S25. ^{1}H NMR (500 MHz, $CDCl_3$) of **Ru^{Cl}-PAr$_3$.**

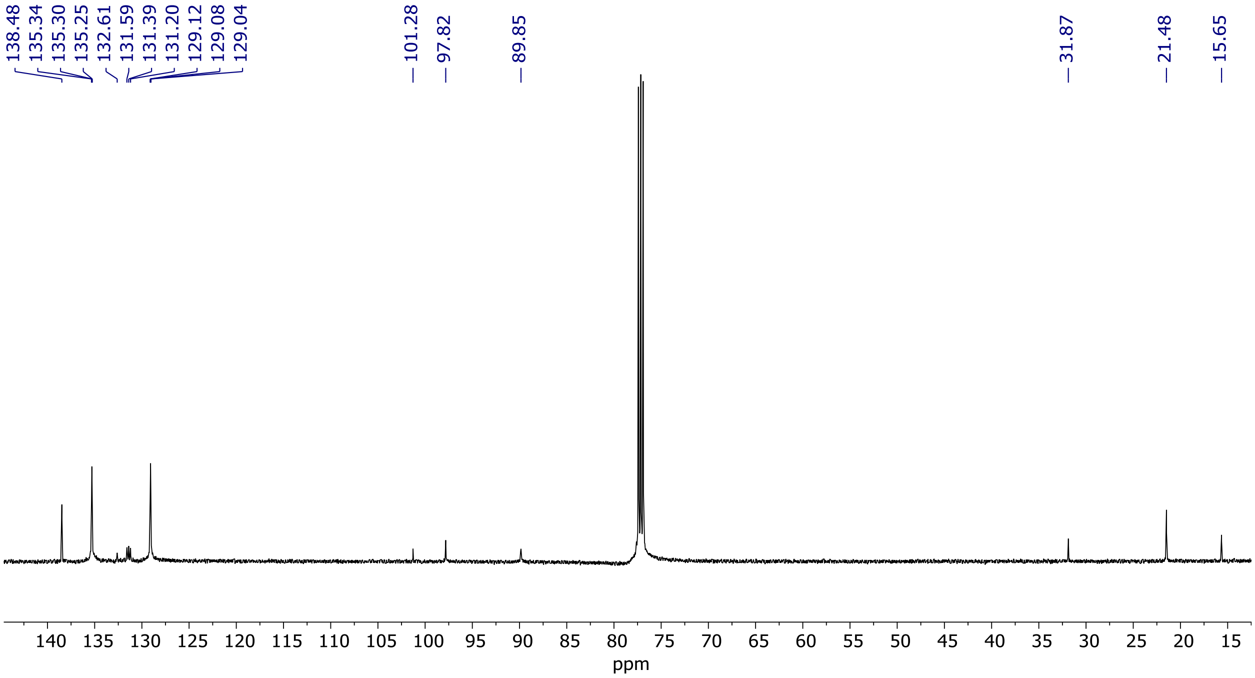

Figure S26. $^{13}C\{^{1}H\}$ NMR spectrum (125 MHz, $CDCl_3$) of **Ru^{Cl}-PAr$_3$**.

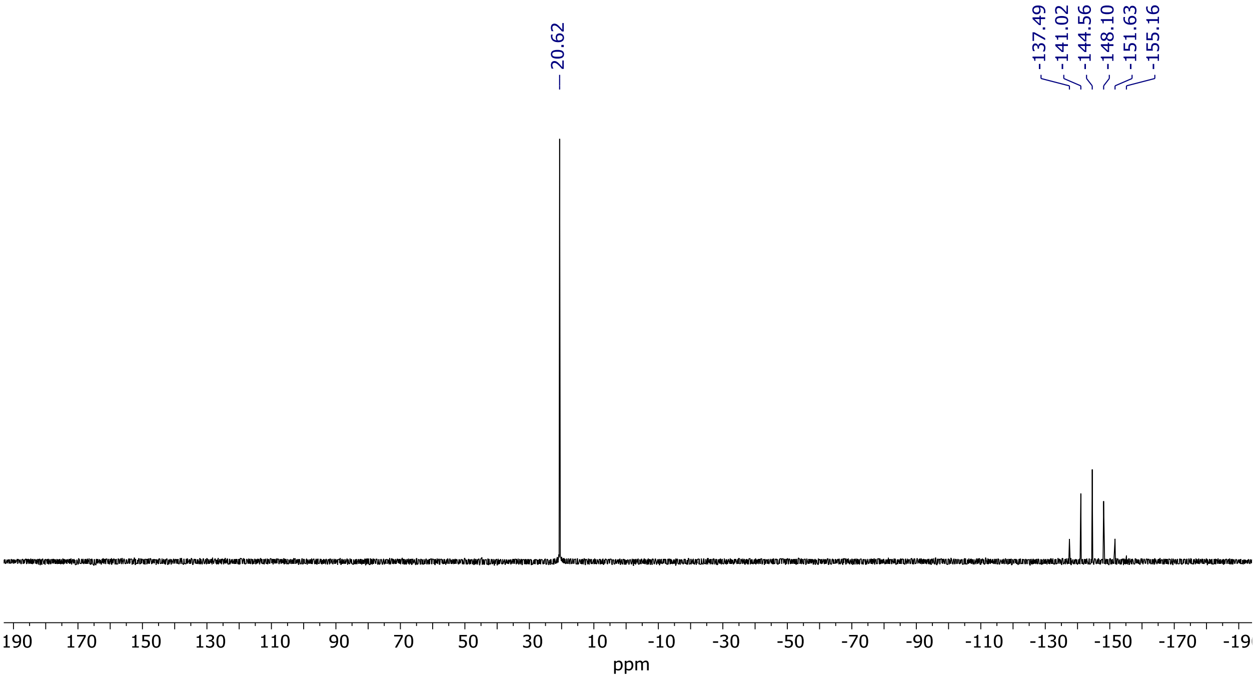

Figure S27. $^{31}P\{^1H\}$ NMR (202 MHz, $CDCl_3$) of **Ru^{Cl}-PAr$_3$**.

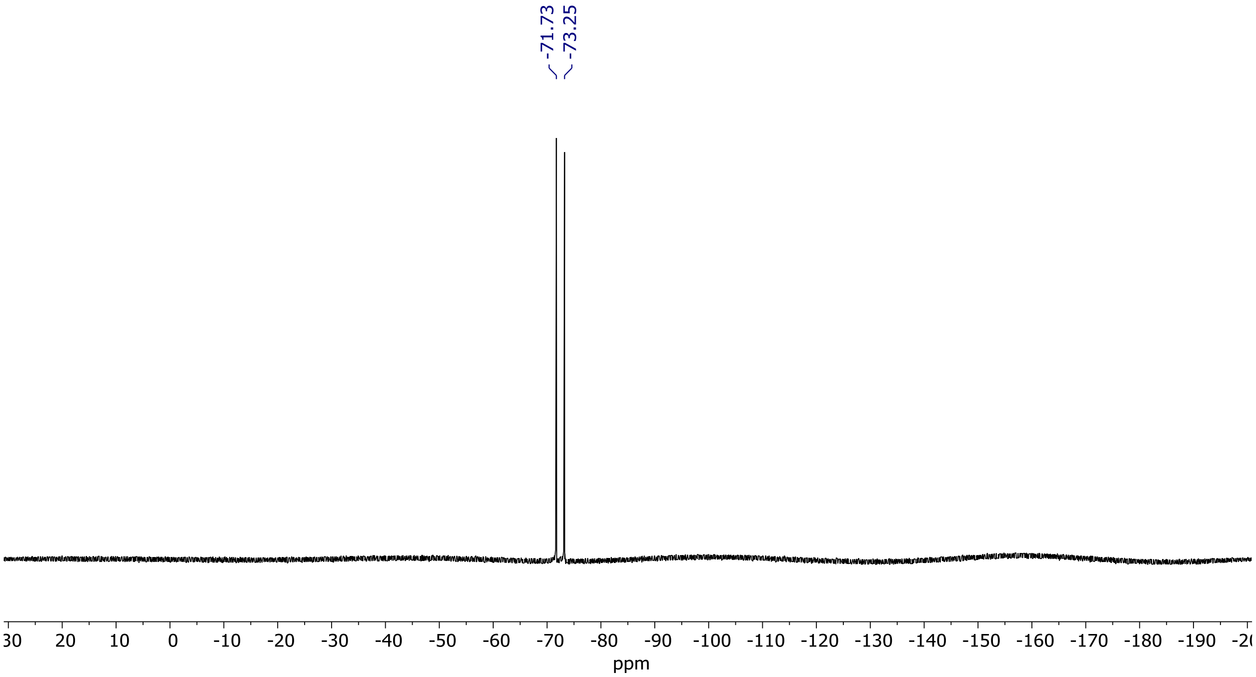

Figure S28. ^{19}F NMR (469 MHz, $CDCl_3$) of **Ru^{Cl}-PAr$_3$**.

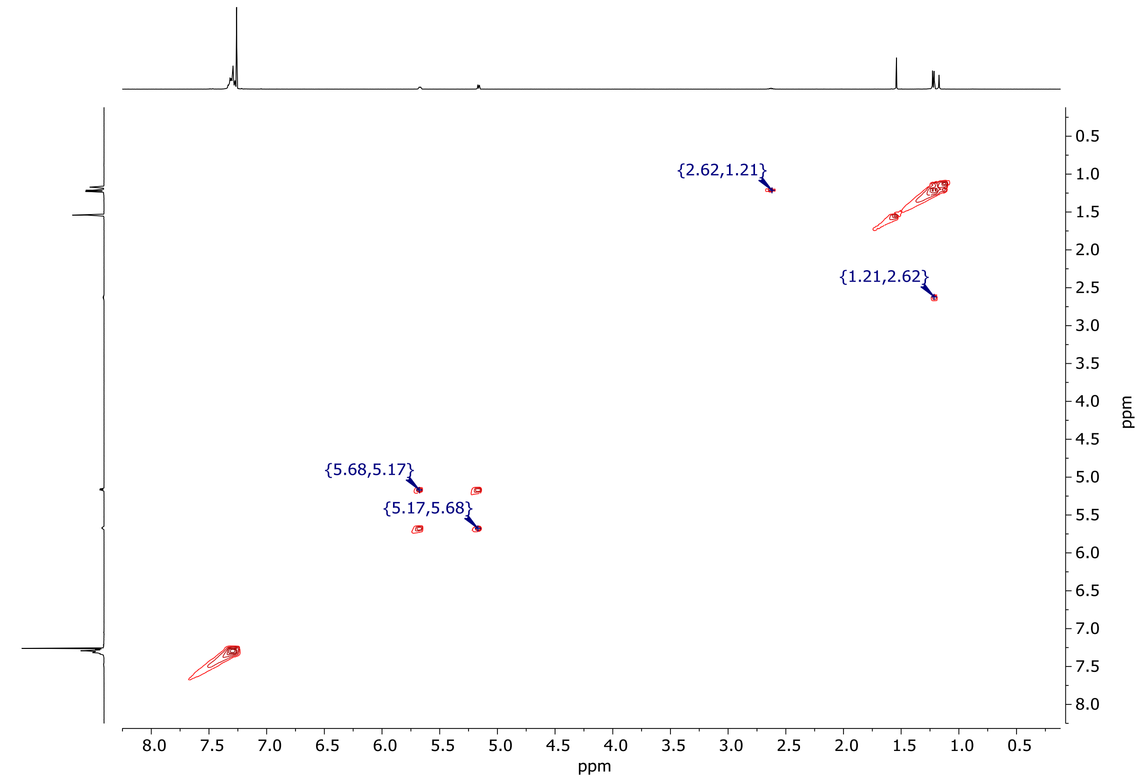

Figure S29. COSY spectrum (500 MHz, $CDCl_3$) of **Ru^{Cl}-PAr$_3$**.

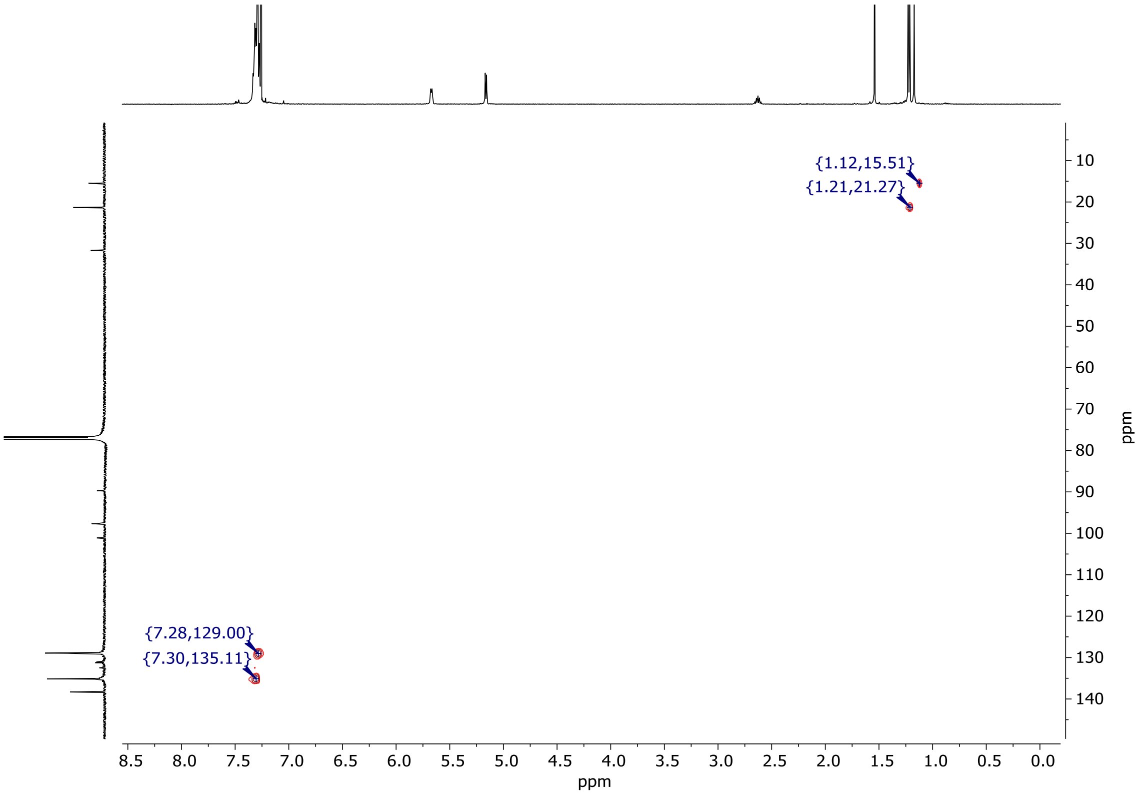

Figure S30. HSQC spectrum (500 MHz, $CDCl_3$) of **Ru^{Cl}-PAr$_3$**.

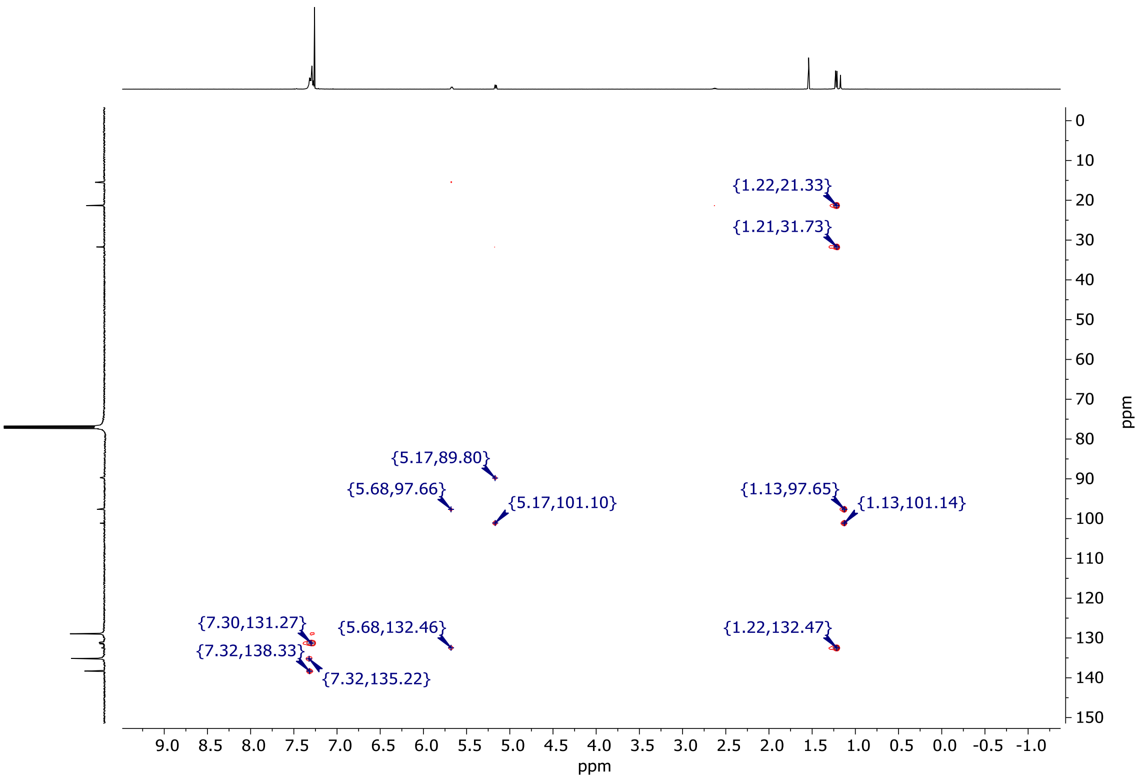

Figure S31. HMBC spectrum (500MHz, $CDCl_3$) of **Ru^{Cl}-PAr$_3$.**

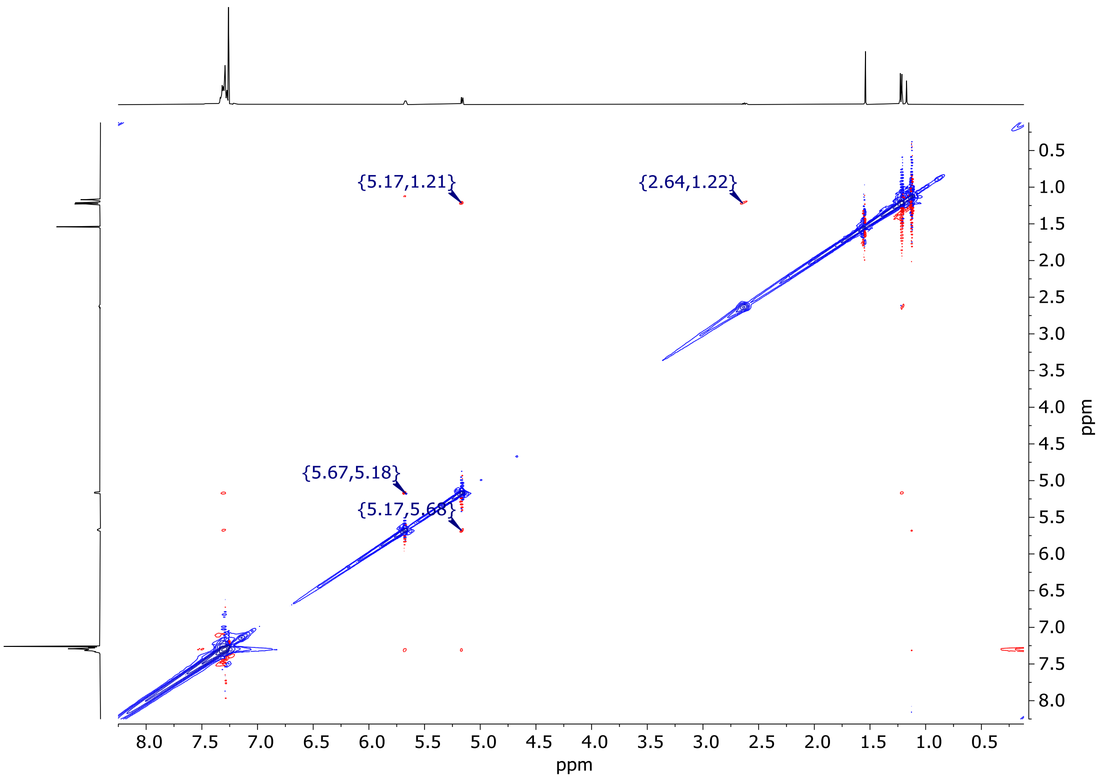

Figure S32. NOESY spectrum (500MHz, $CDCl_3$) of **Ru^{Cl}-PAr$_3$**.

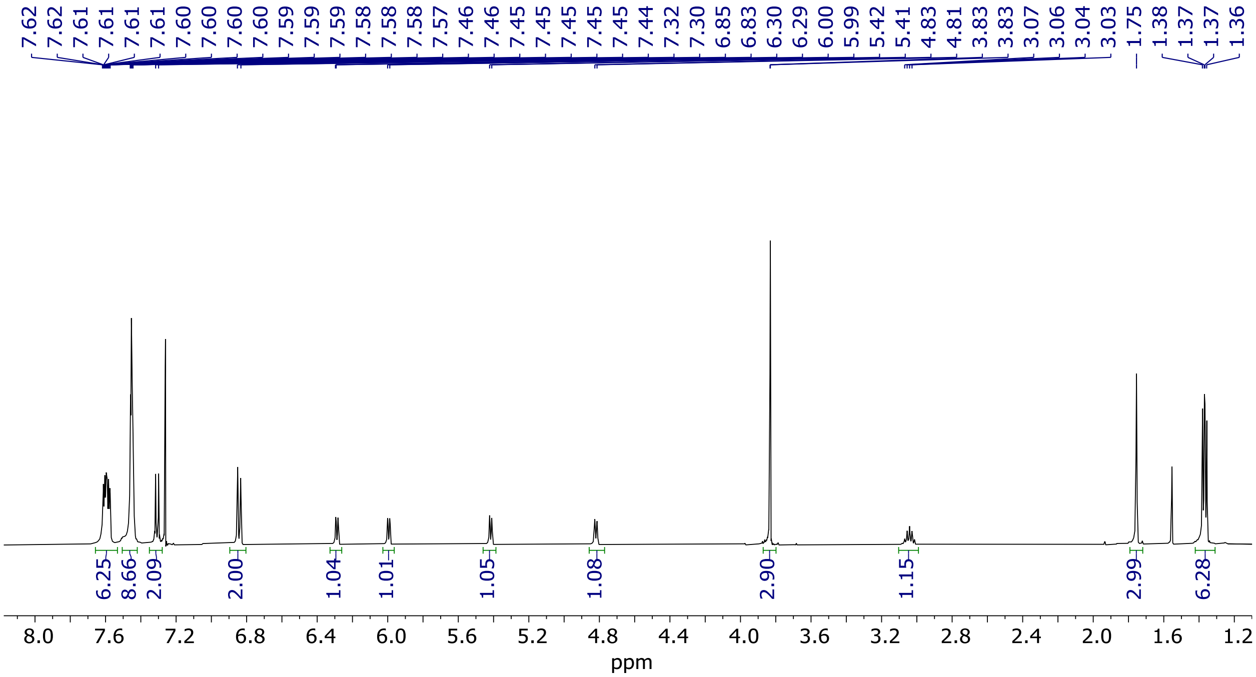

Figure 33. ^{1}H NMR (500 MHz, $CDCl_3$) of **Ru^{H}-NCAr^{OMe}.**

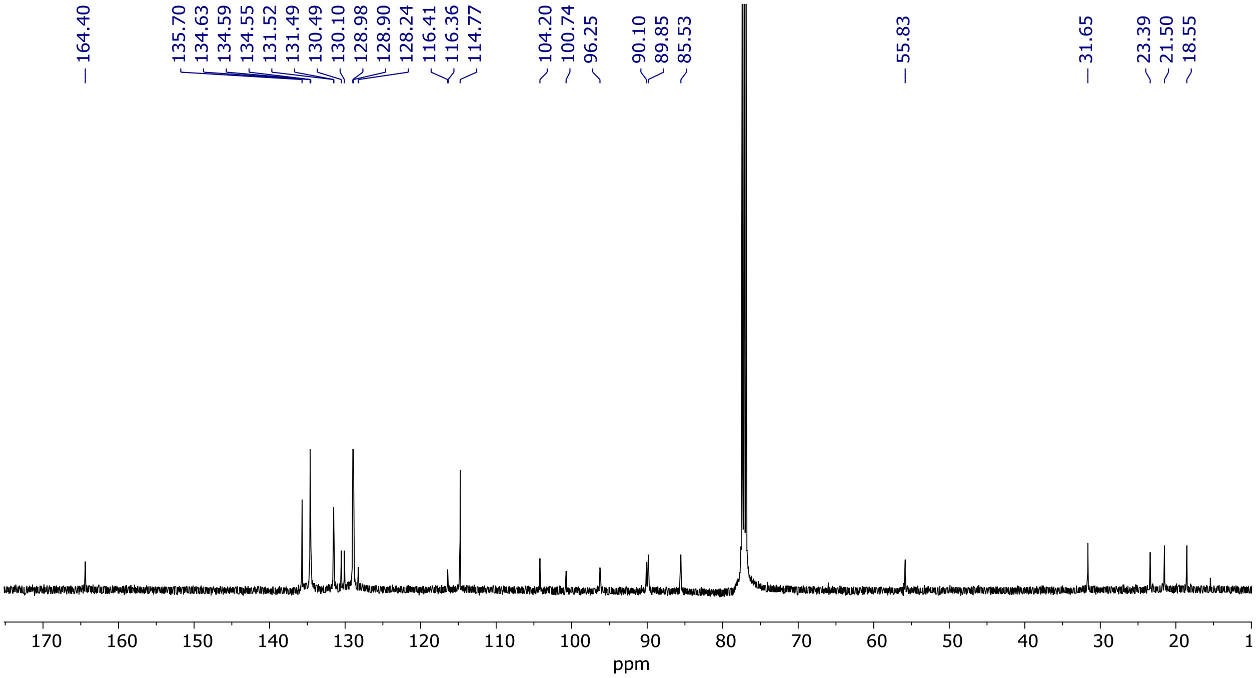

Figure S34. $^{13}C\{^{1}H\}$ NMR spectrum (125 MHz, $CDCl_3$) of **Ru^{H}-NCAr^{OMe}**.

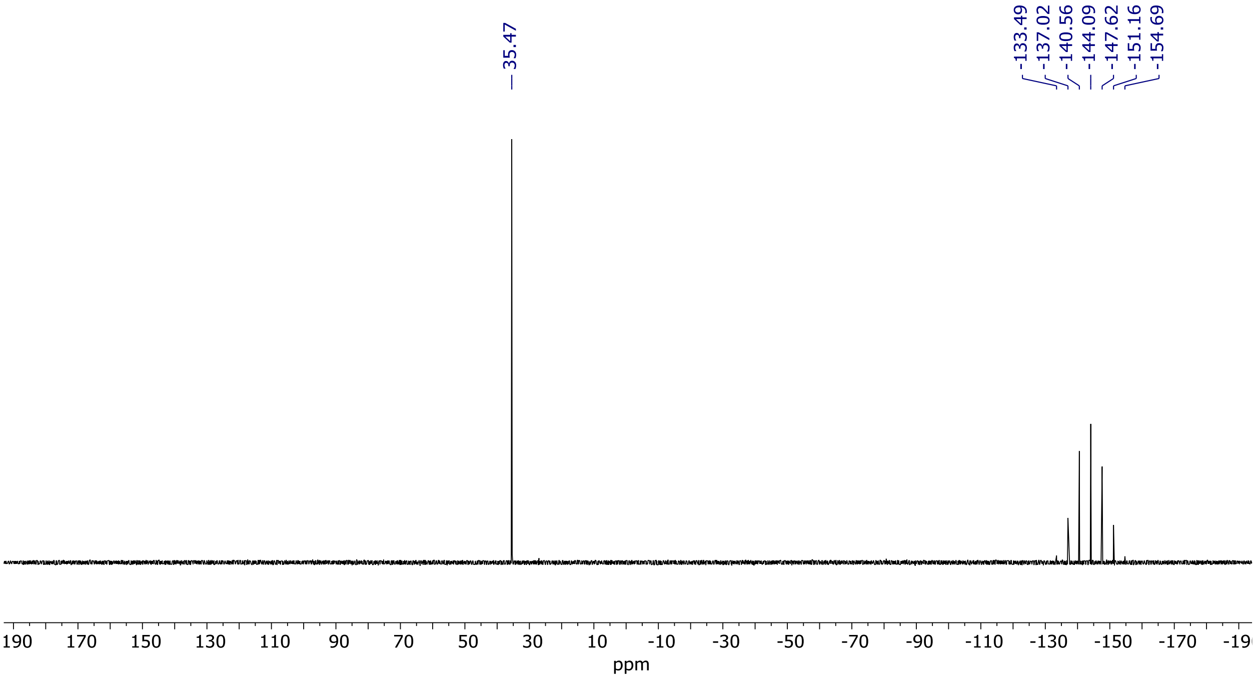

Figure S35. $^{31}P\{^{1}H\}$ NMR (202 MHz, $CDCl_3$) of $\mathbf{Ru^{H}\text{-}NCAr^{OMe}}$.

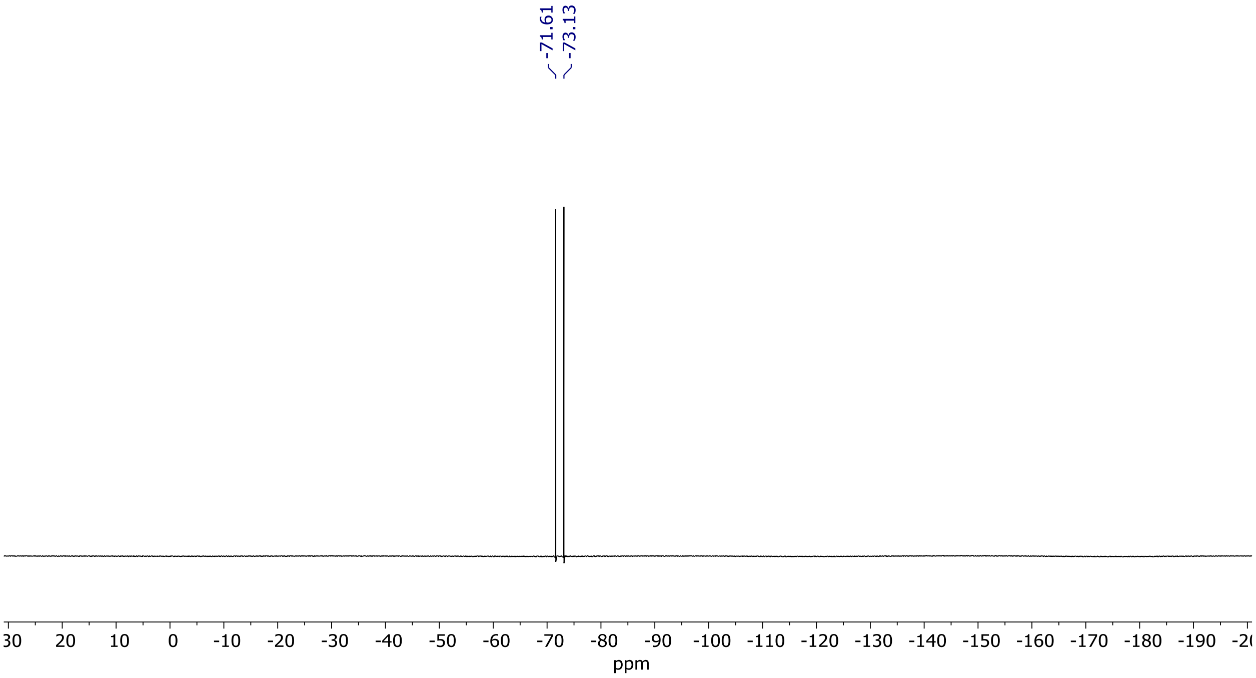

Figure S36. ^{19}F NMR (469 MHz, $CDCl_3$) of **4-OMe**.

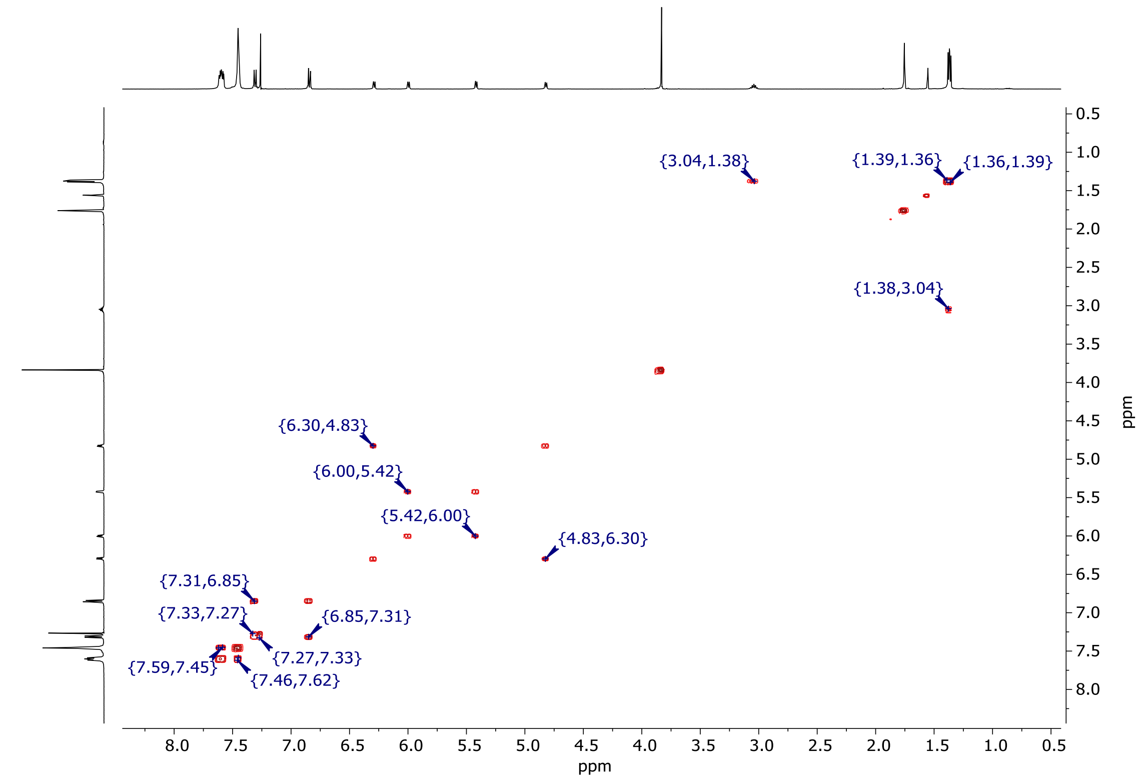

Figure S37. COSY spectrum (500 MHz, $CDCl_3$) of **Ru^{H}-NCAr^{OMe}**.

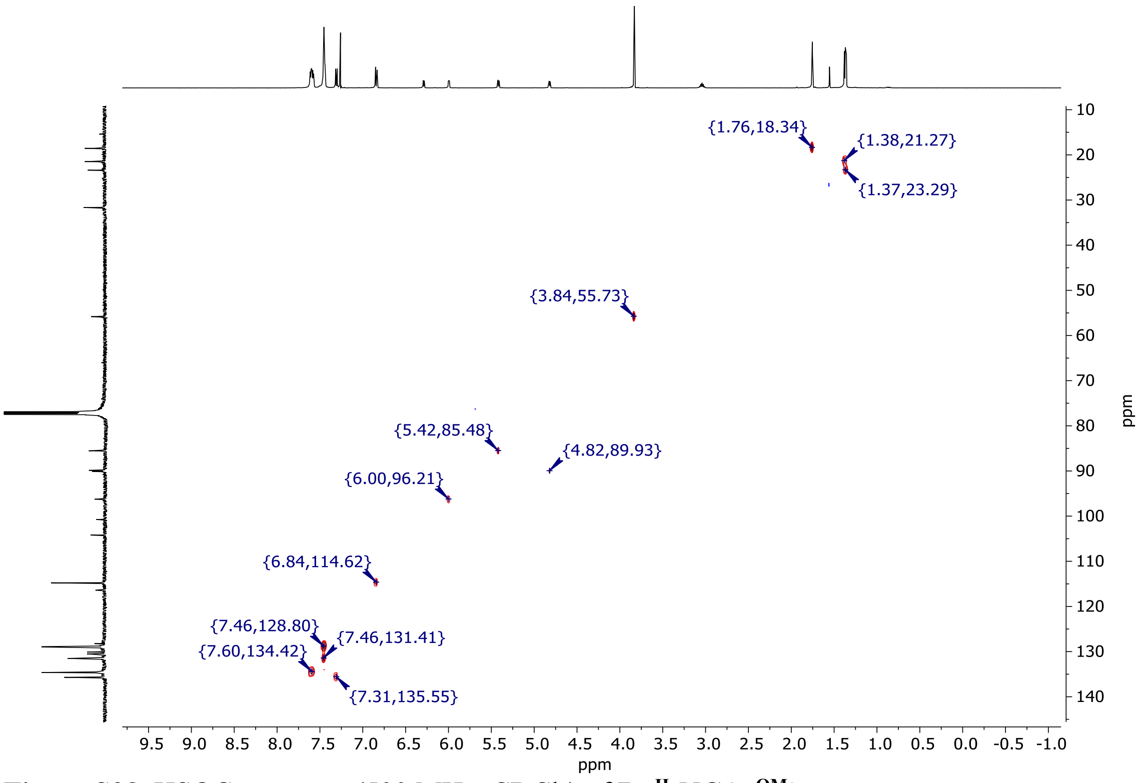

Figure S38. HSQC spectrum (500 MHz, $CDCl_3$) of **Ru^{H}-NCAr^{OMe}**.

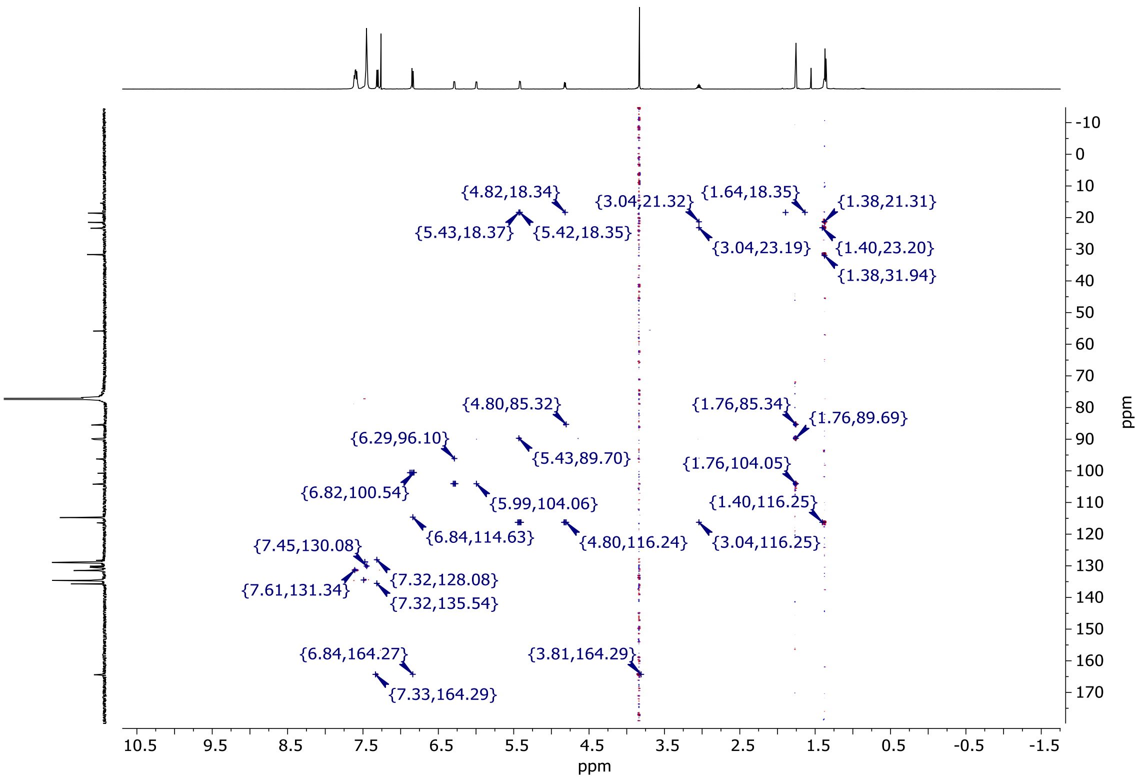

Figure S39. HMBC spectrum (500MHz, $CDCl_3$) of **Ru^{H}-NCAr^{OMe}**.

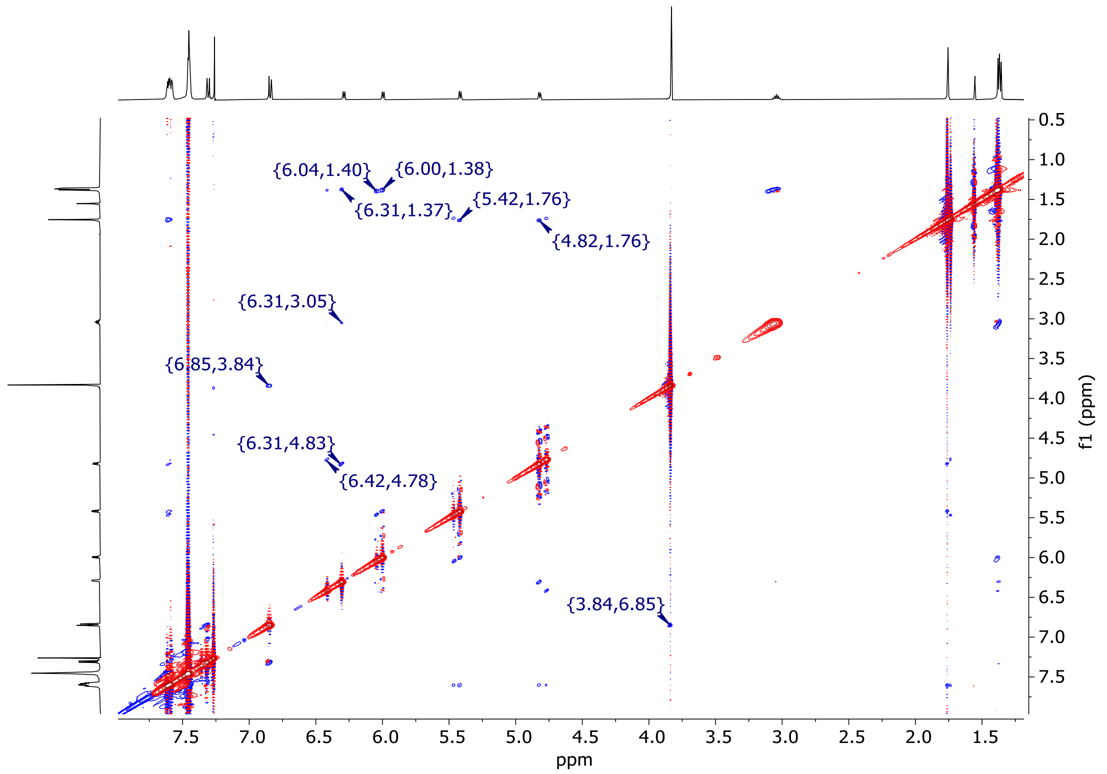

Figure S40. NOESY spectrum (400 MHz, CD_2Cl_2) of **Ru^{H}-NCAr^{OMe}**.

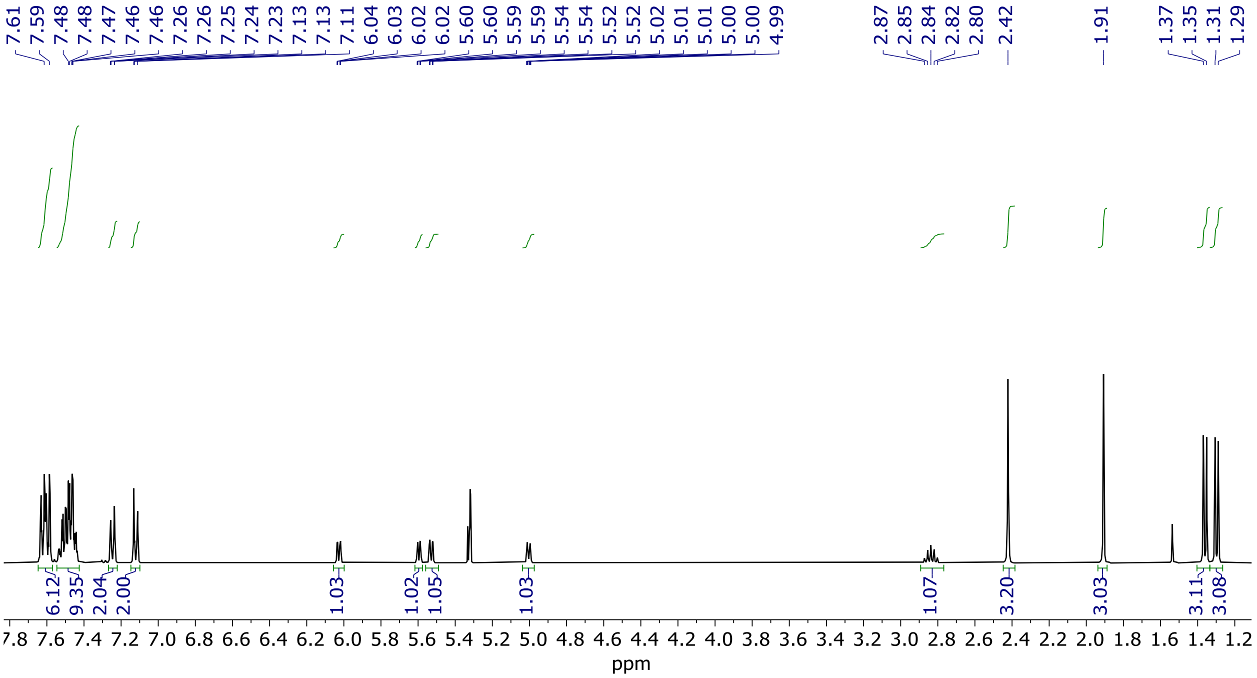

Figure S41. ^{1}H NMR (400 MHz, CD_2Cl_2) of **Ru^{H}-NCAr^{Me}**.

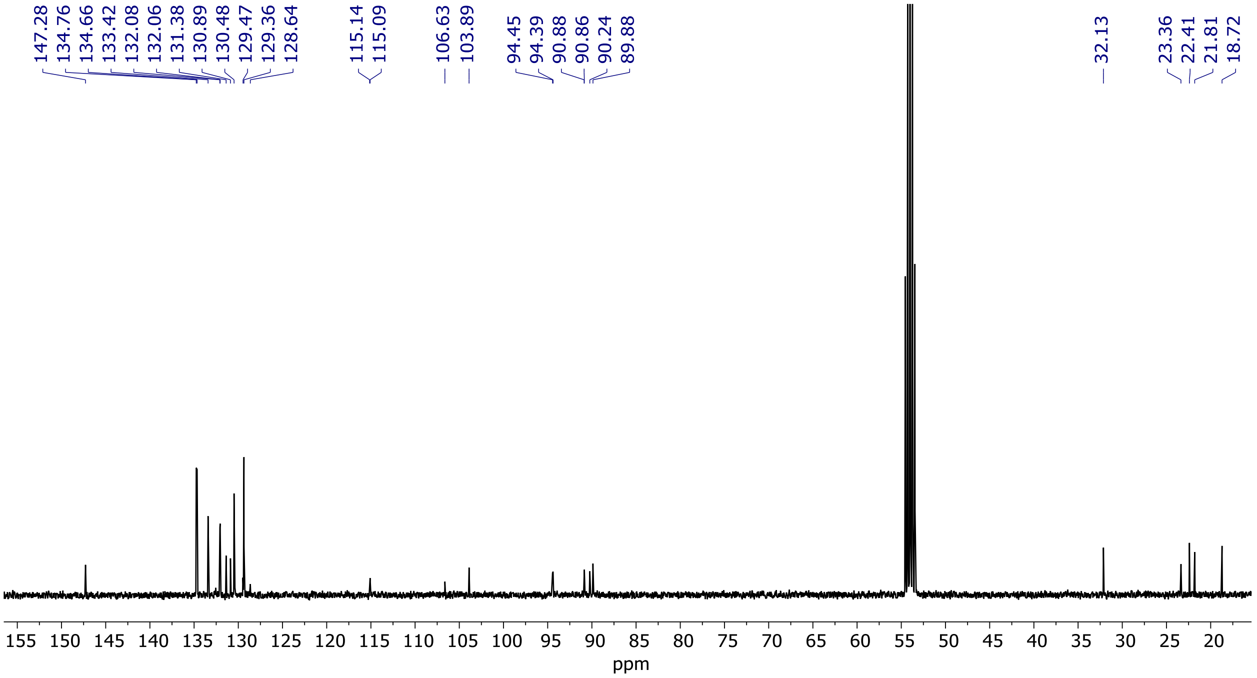

Figure S42. $^{13}C\{^{1}H\}$ NMR spectrum (101 MHz, CD_2Cl_2) of **Ru^{H}-NCAr^{Me}**.

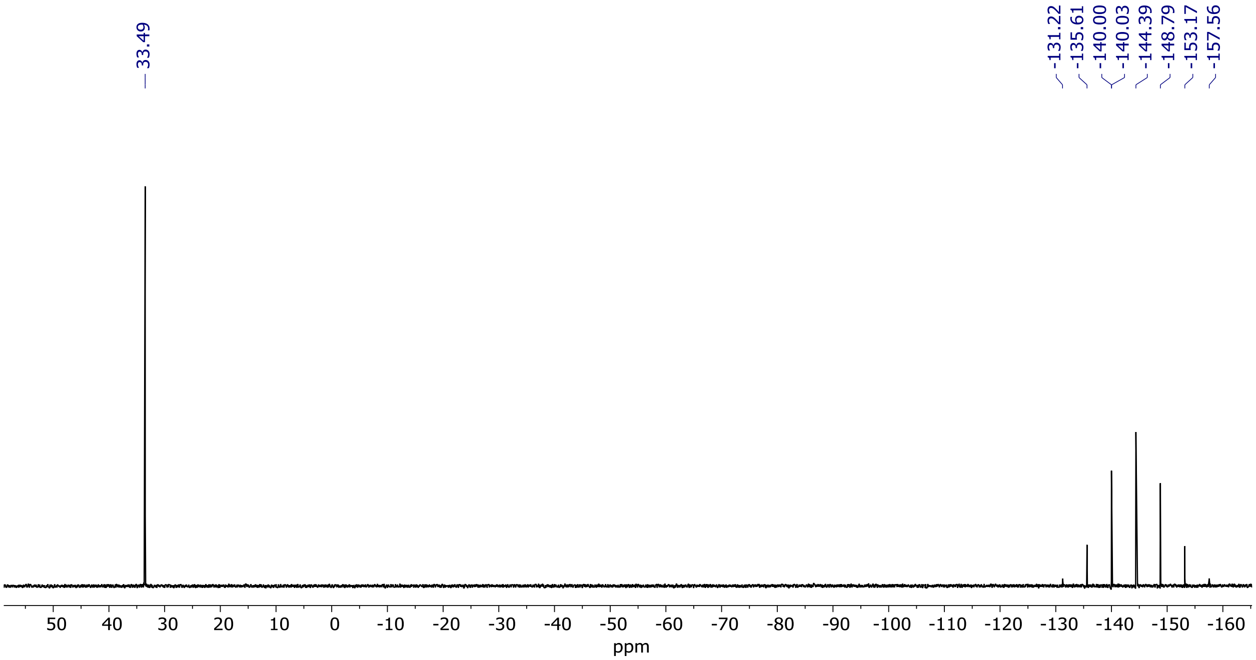

Figure S43. $^{31}P\{^1H\}$ NMR (162 MHz, CD_2Cl_2) of **Ru^{H}-NCAr^{Me}**.

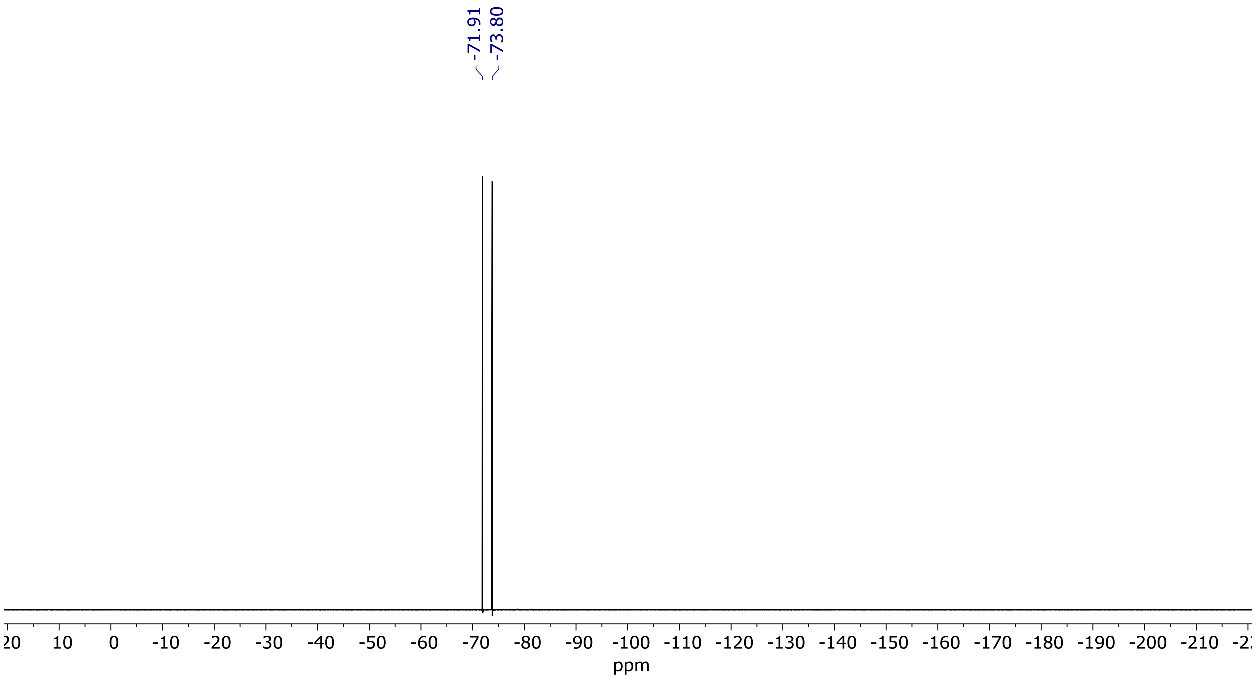

Figure S44. ^{19}F NMR (376 MHz, CD_2Cl_2) of **Ru^{H}-NCAr^{Me}**.

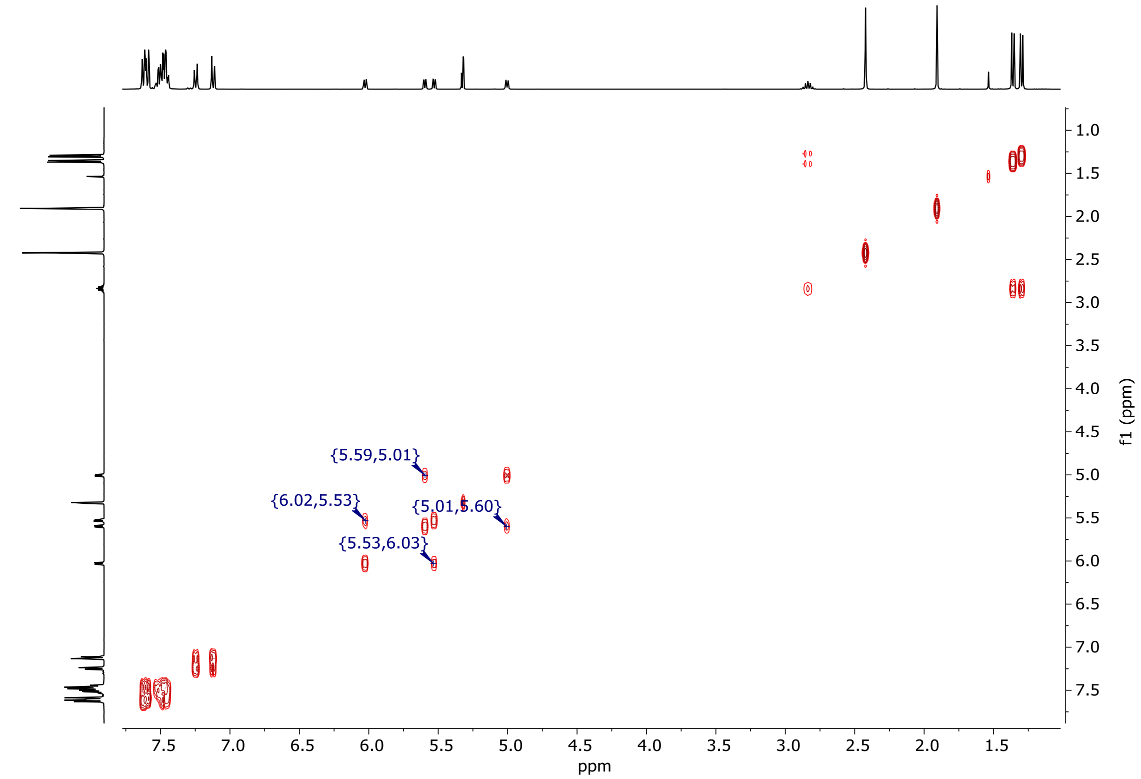

Figure S45. COSY spectrum (400 MHz, CD_2Cl_2) of **Ru^{H}-NCAr^{Me}**.

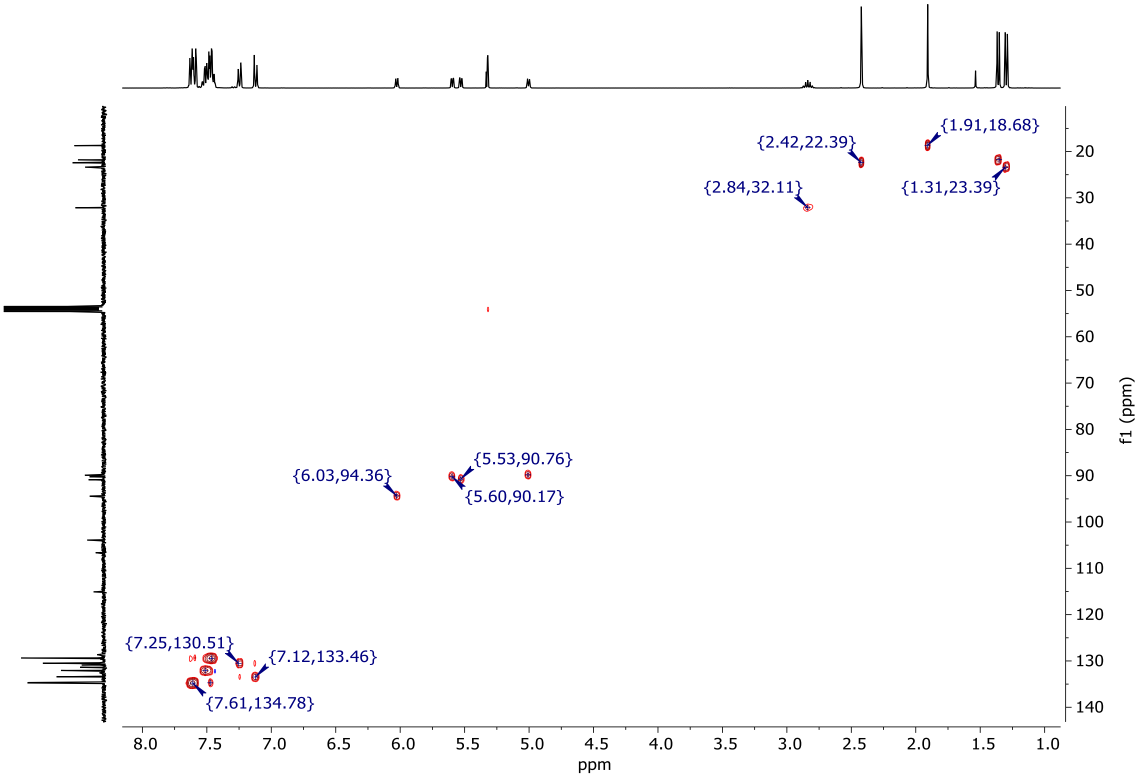

Figure S46. HSQC spectrum (400 MHz, CD_2Cl_2) of **Ru^{H}-NCAr^{Me}**.

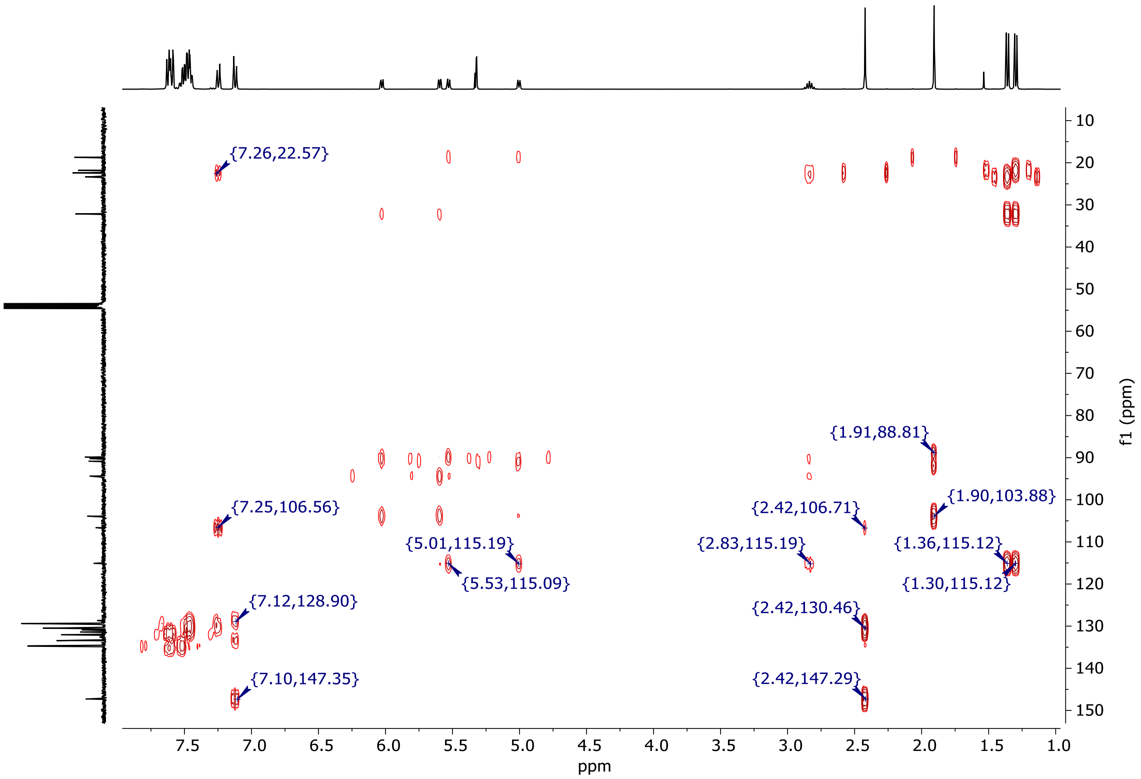

Figure S47. HMBC spectrum (400 MHz, CD_2Cl_2) of **Ru^{H}-NCAr^{Me}**.

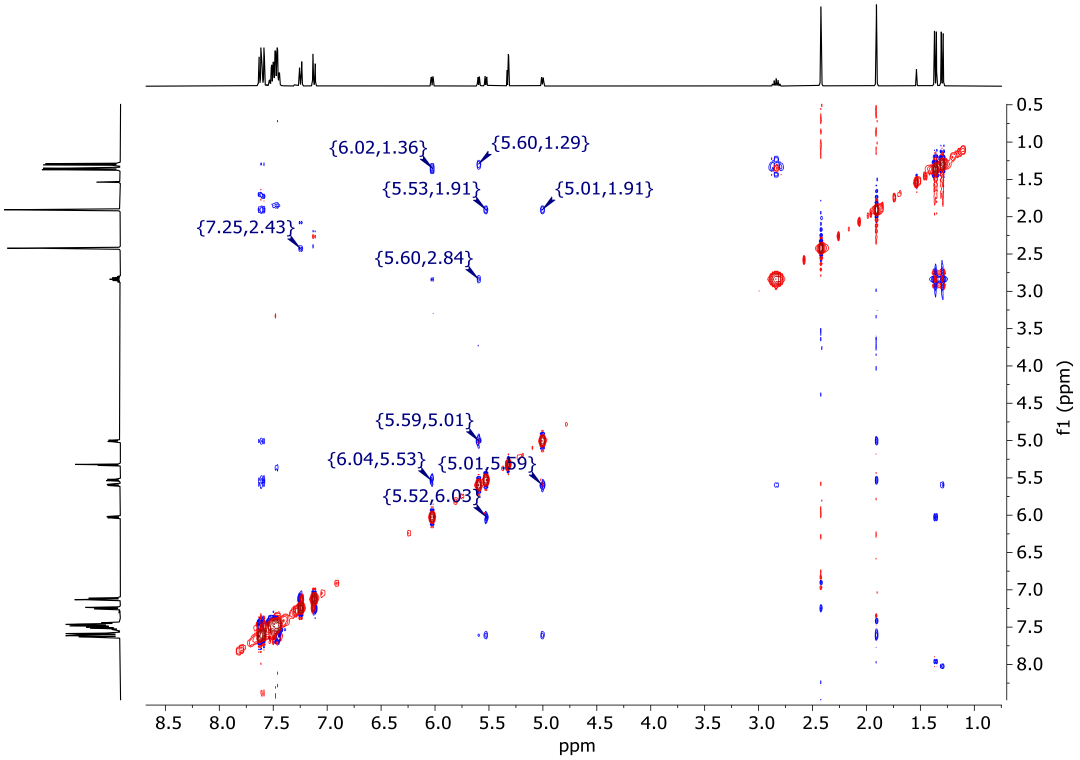

Figure S48. NOESY spectrum (400 MHz, CD_2Cl_2) of **Ru^{H}-NCAr^{Me}**.

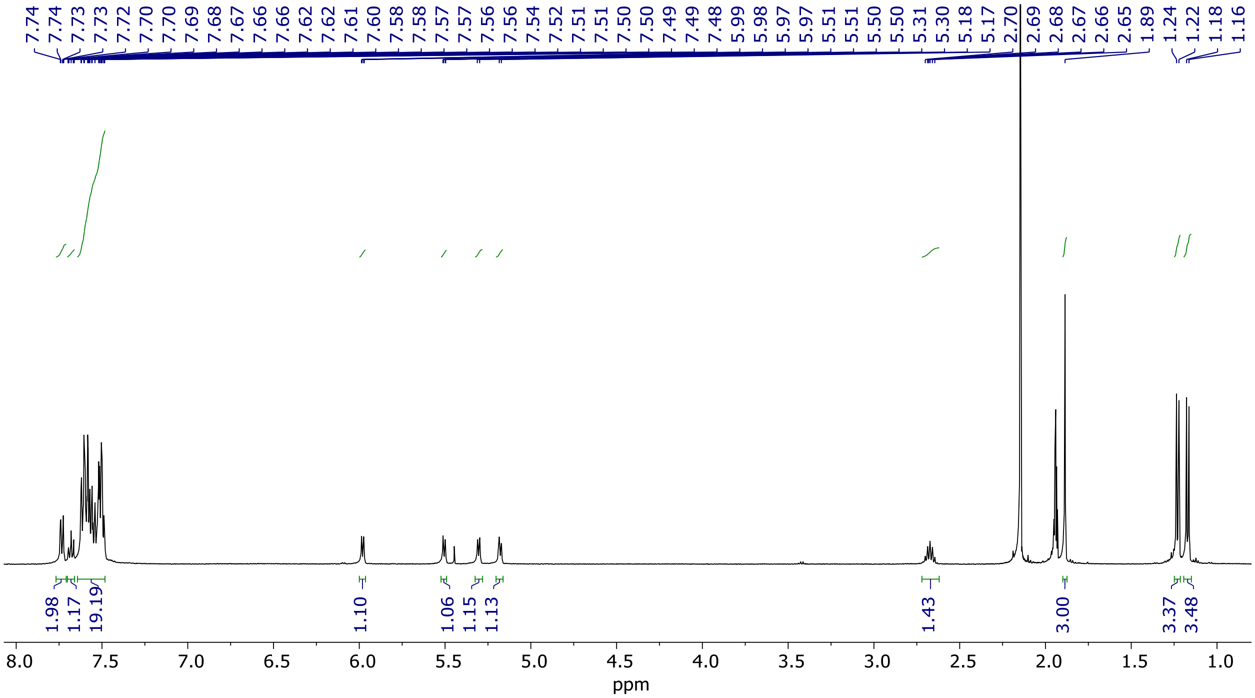

Figure S49. ^{1}H NMR (400 MHz, CD_2Cl_2) of **Ru^{H}-NCAr^{H}**.

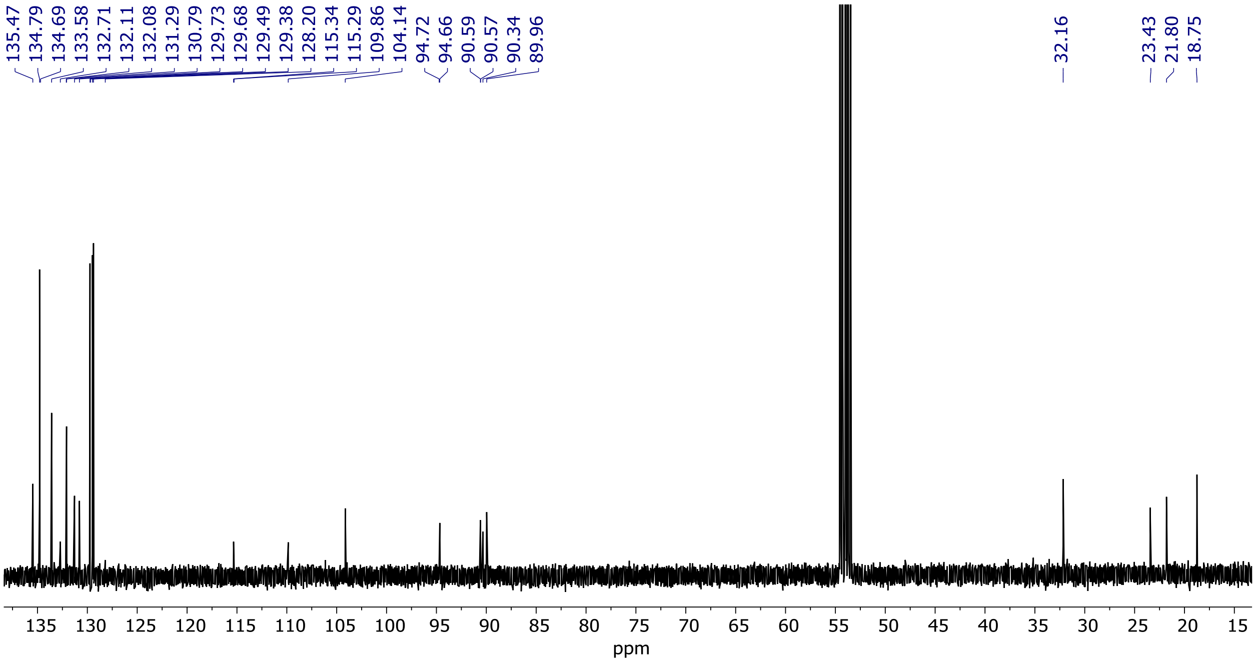

Figure S50. $^{13}C\{^{1}H\}$ NMR spectrum (101 MHz, CD_2Cl_2) of **Ru^{H}-NCAr^{H}** .

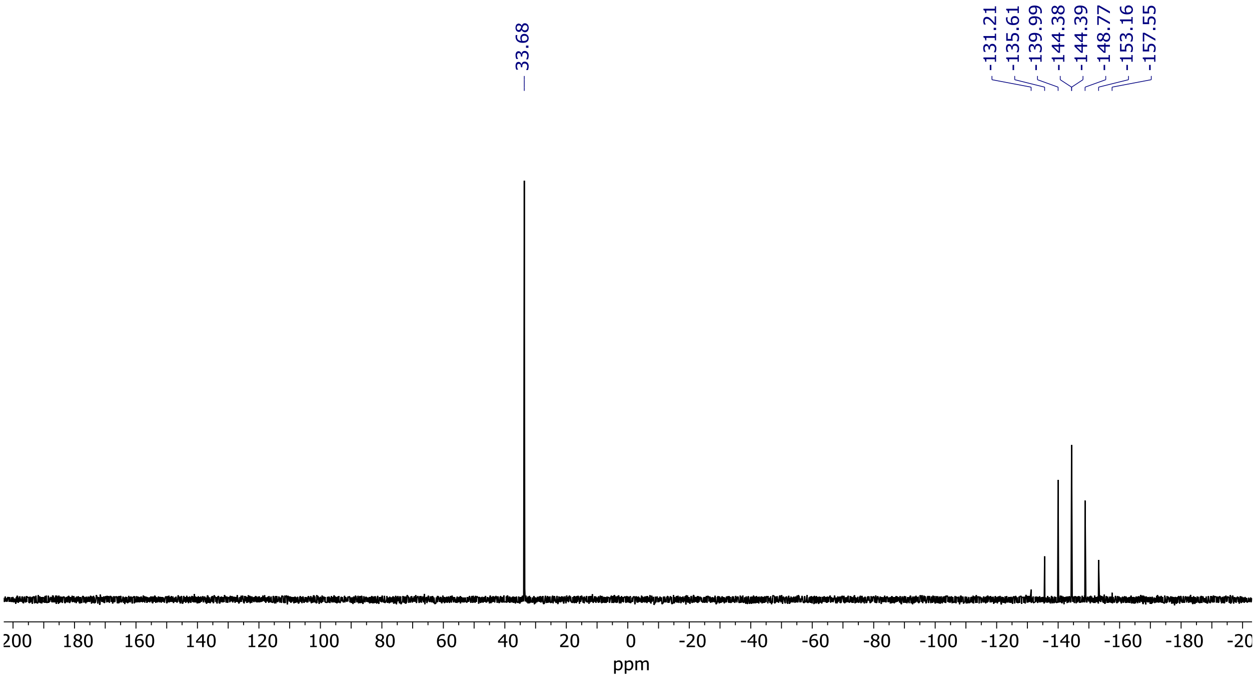

Figure S51. $^{31}P\{^{1}H\}$ NMR (162 MHz, CD_2Cl_2) of **Ru^{H}-NCAr^{H}**.

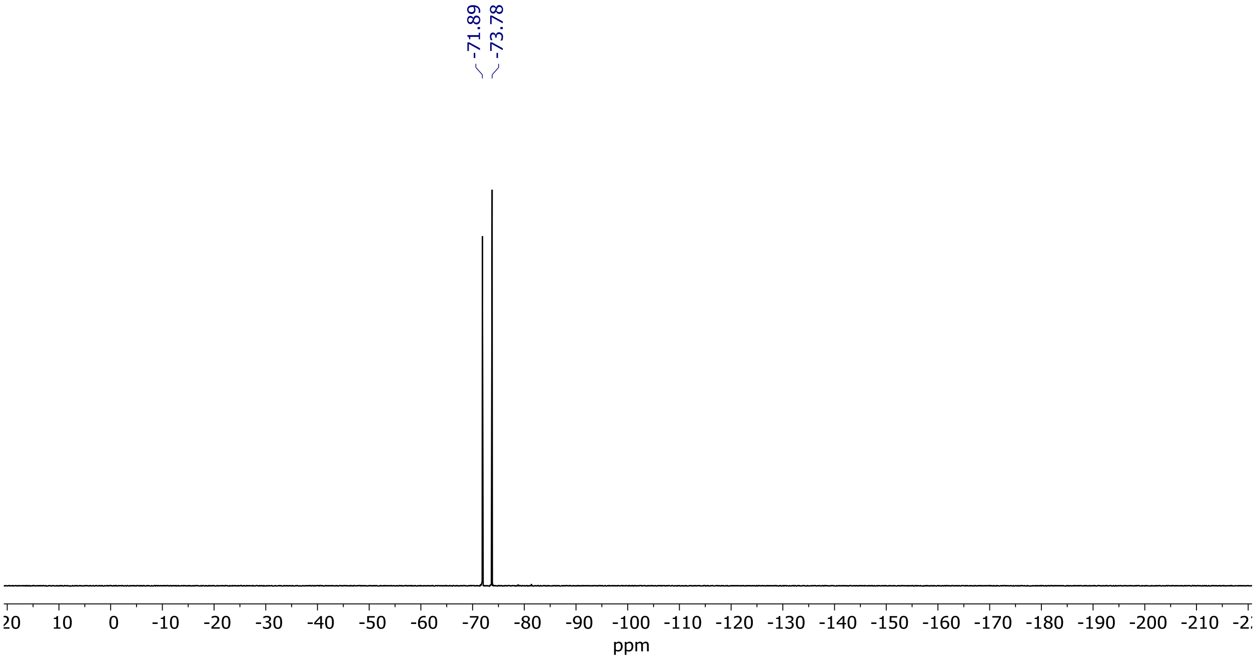

Figure S52. ^{19}F NMR (376 MHz, CD_2Cl_2) of **Ru^{H}-NCAr^{H}**.

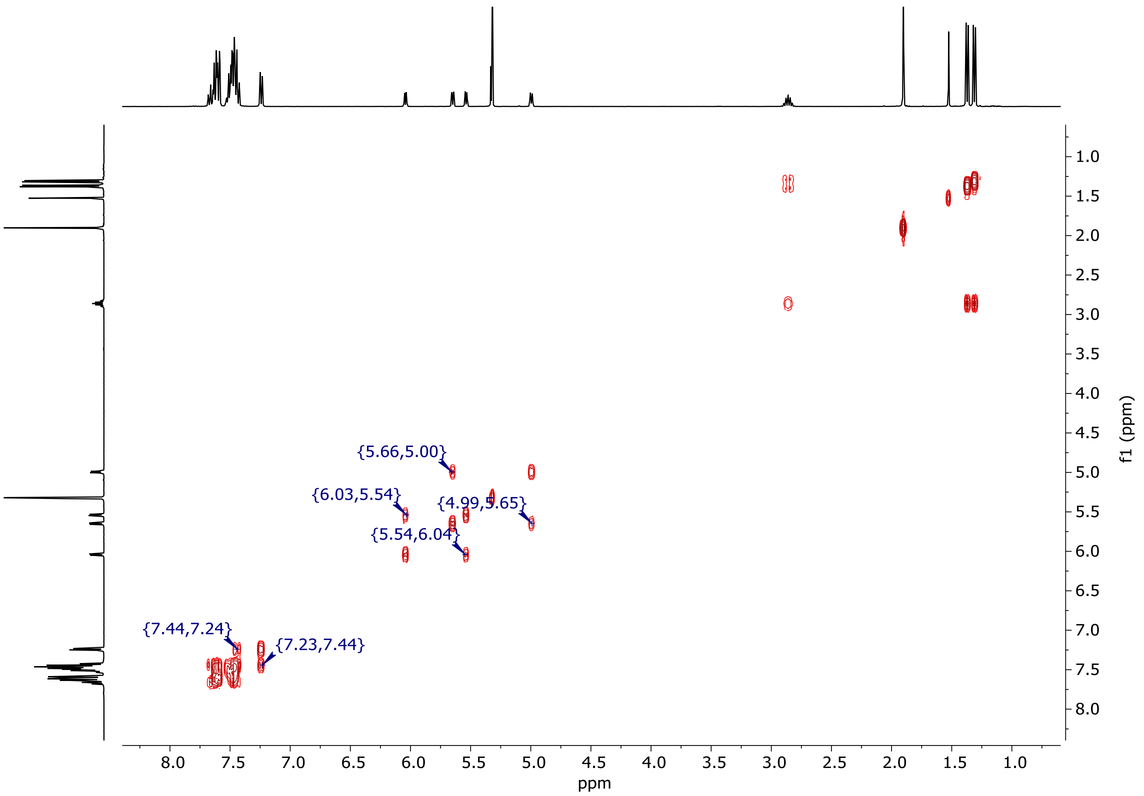

Figure S53. COSY spectrum (400 MHz, CD_2Cl_2) of **Ru^{H}-NCAr^{H}**.

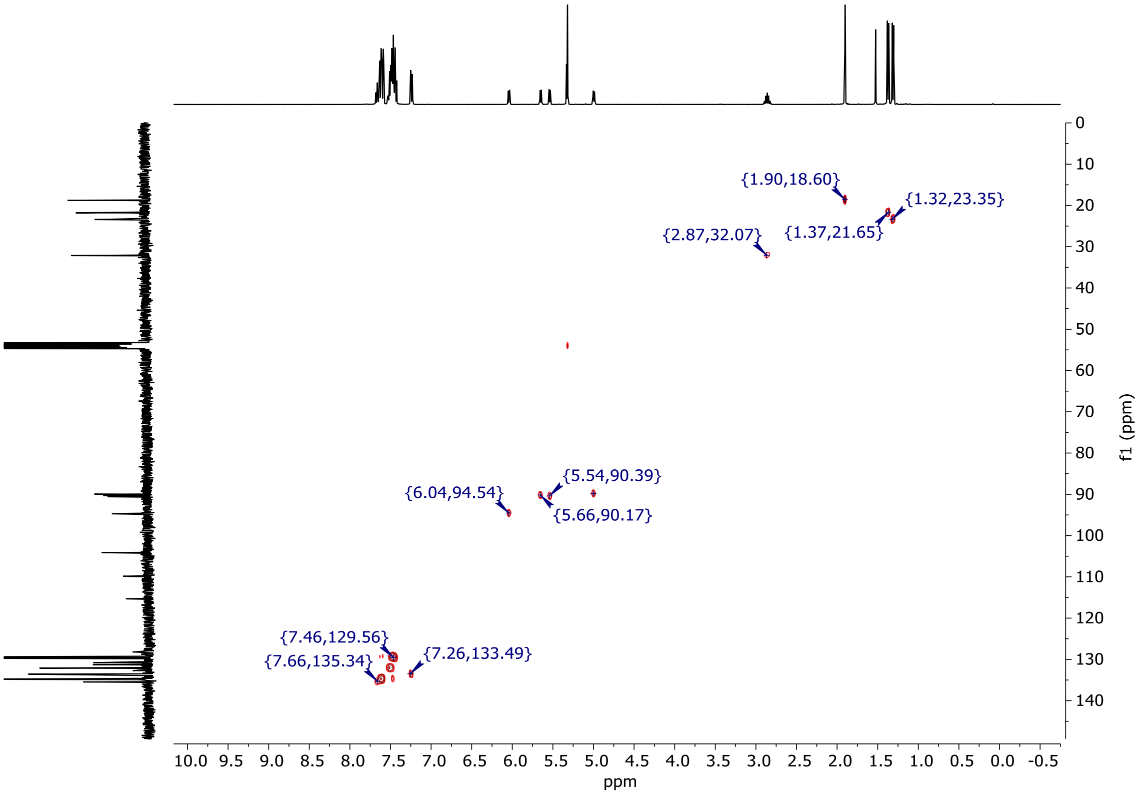

Figure S54. HSQC spectrum (400 MHz, CD_2Cl_2) of **Ru^{H}-NCAr^{H}**.

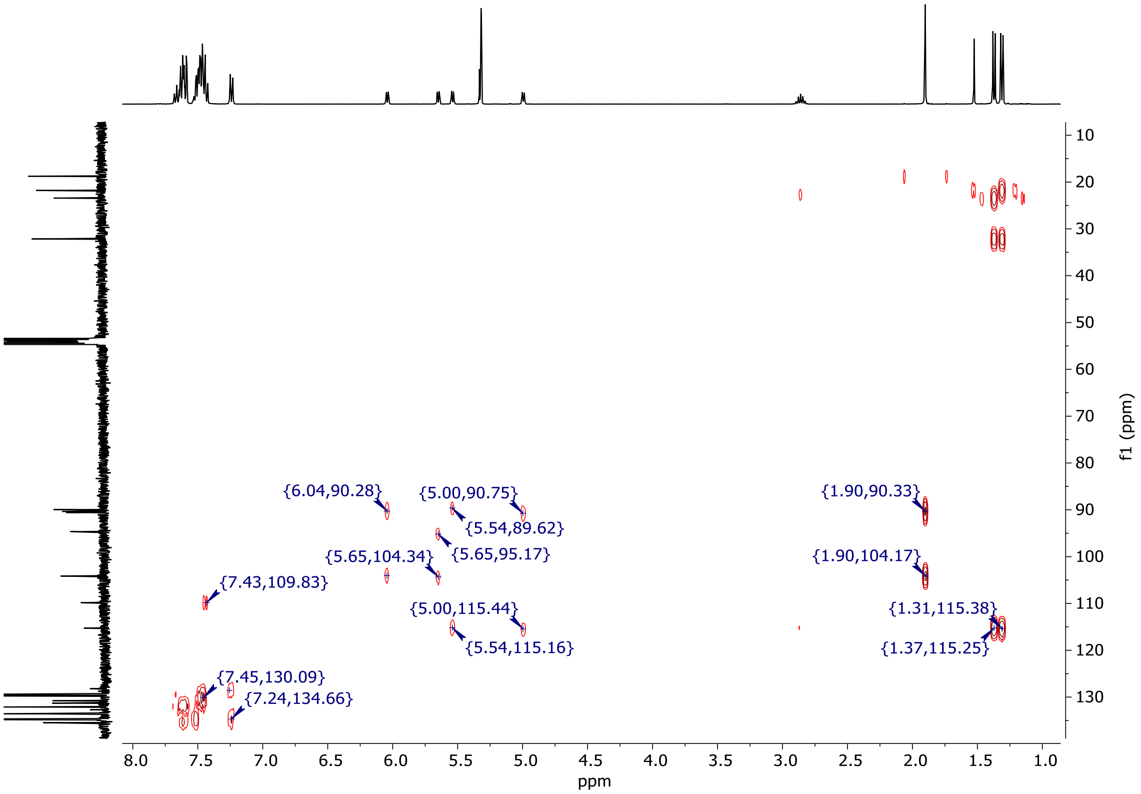

Figure S55. HMBC spectrum (400 MHz, CD_2Cl_2) of **Ru^{H}-NCAr^{H}**.

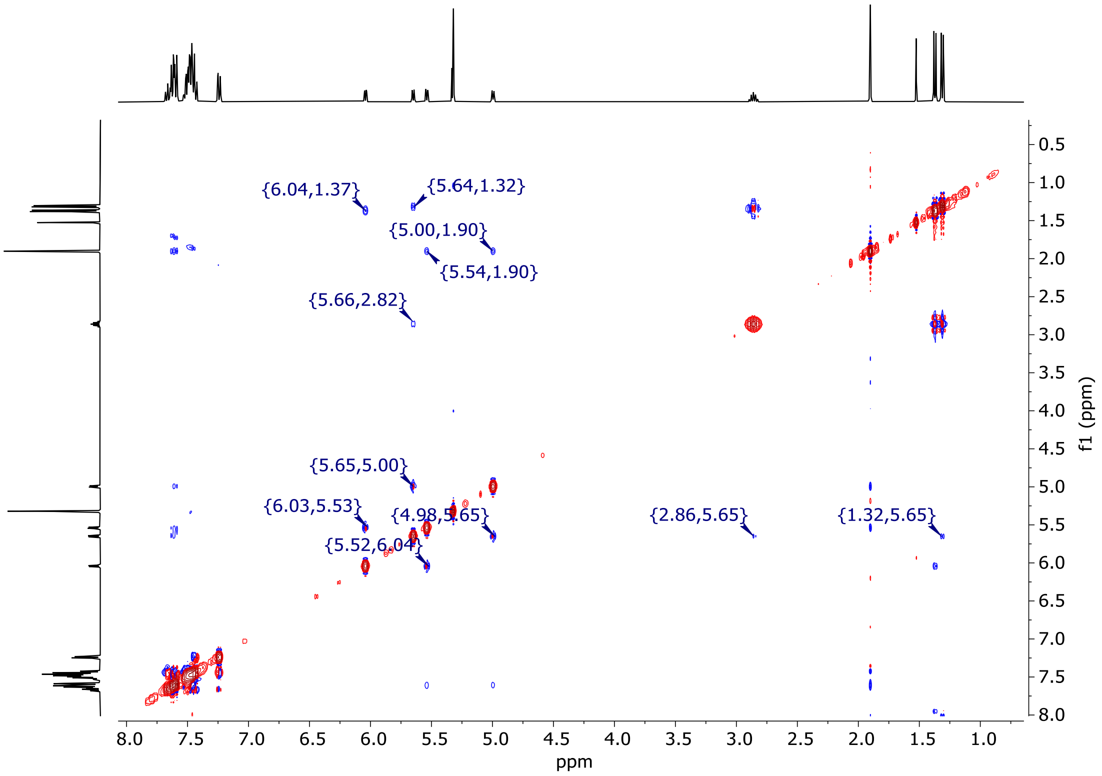

Figure S56. NOESY spectrum (400 MHz, CD_2Cl_2) of **Ru^{H}-NCAr^{H}**.

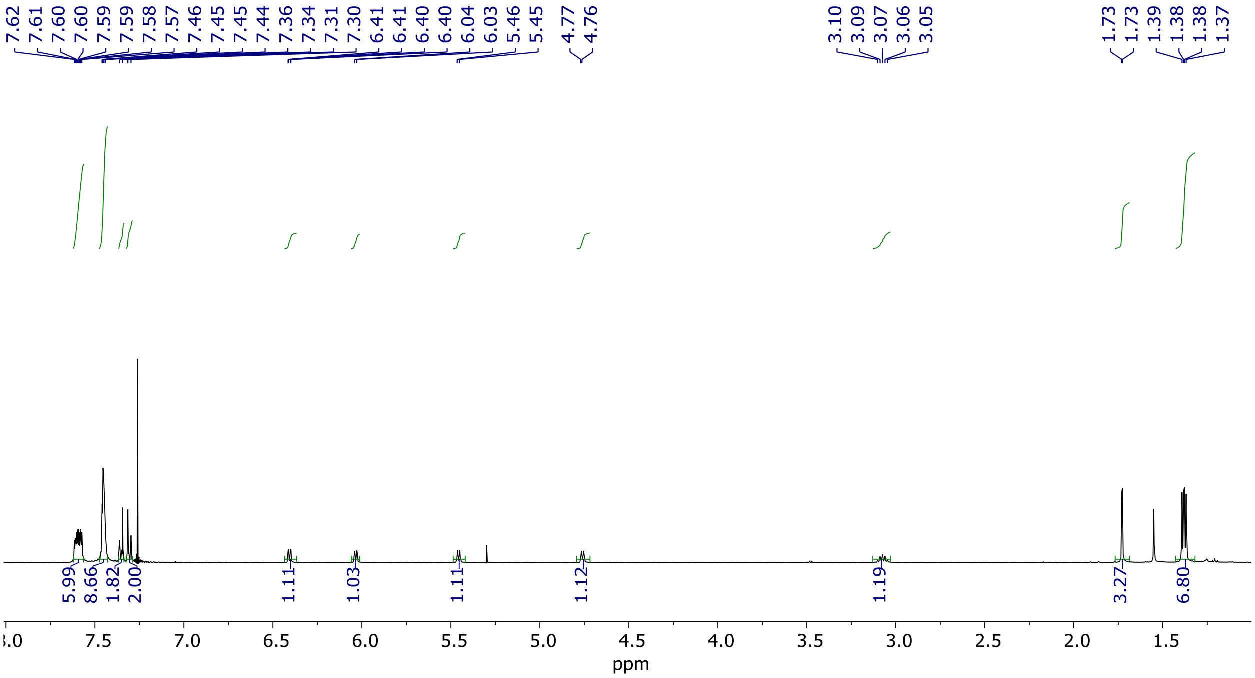

Figure S57. ^{1}H NMR (500 MHz, $CDCl_3$) of **Ru^{H}-NCAr^{Cl}.**

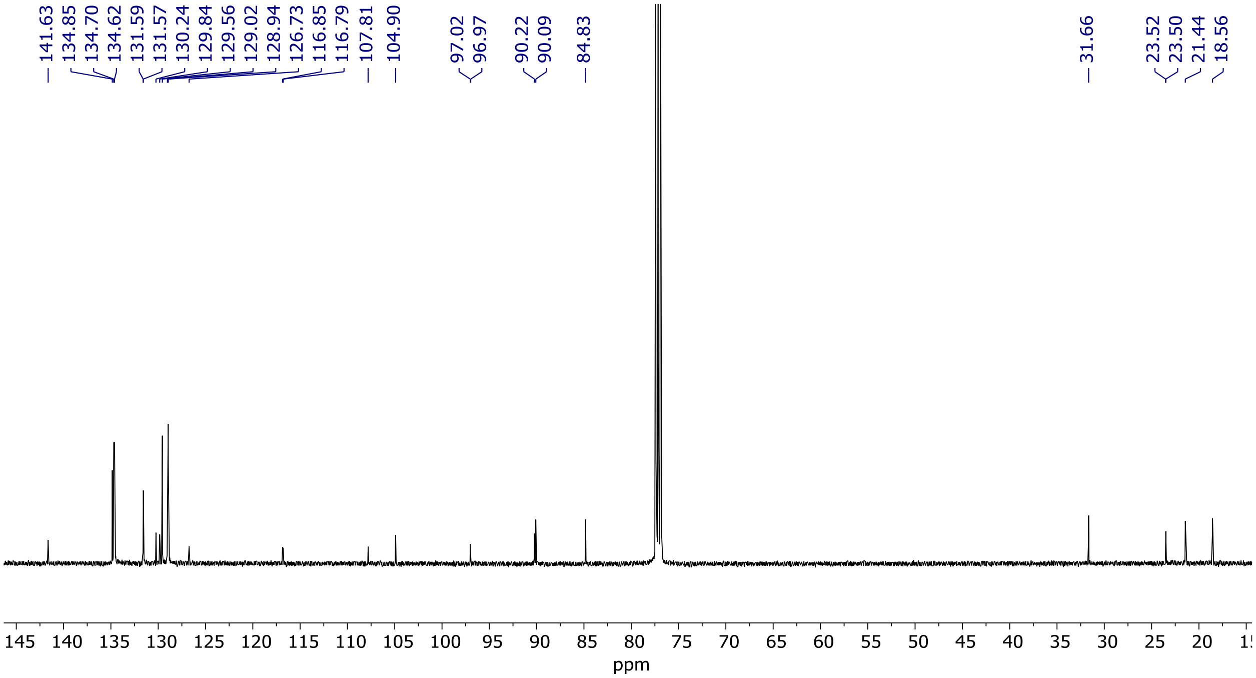

Figure S58. $^{13}C\{^{1}H\}$ NMR spectrum (125 MHz, $CDCl_3$) of **Ru^{H}-NCAr^{Cl}**.

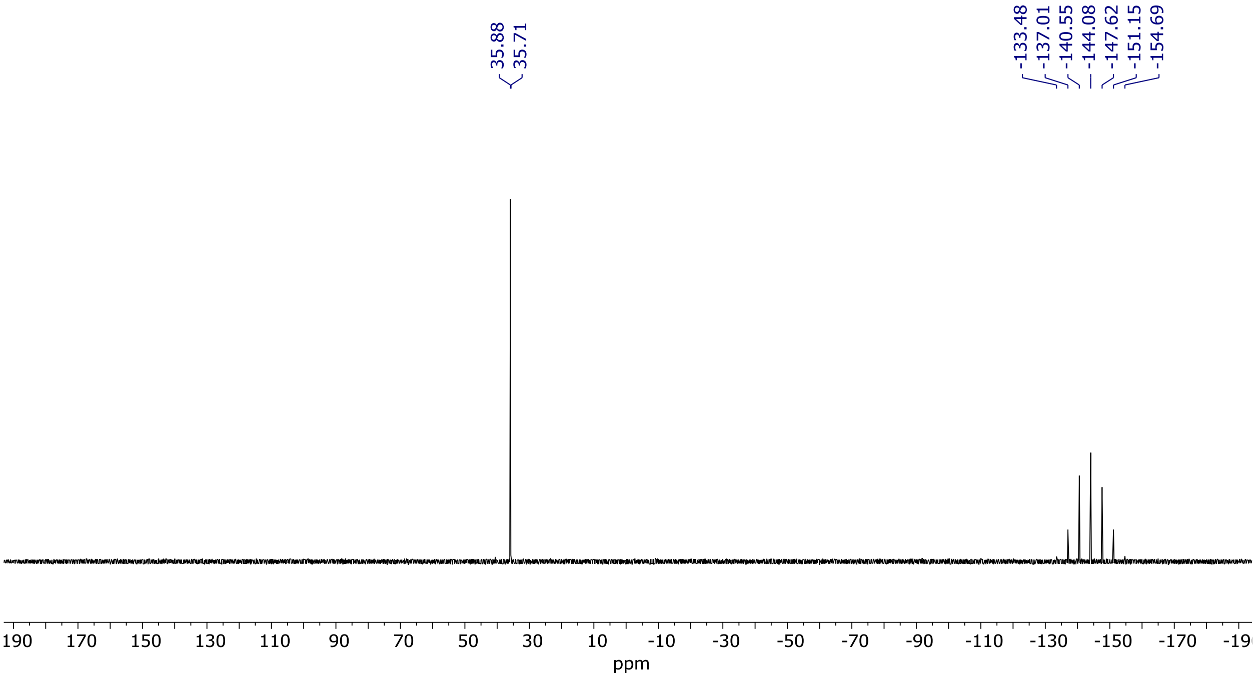

Figure S59. $^{31}P\{^{1}H\}$ NMR (202 MHz, $CDCl_3$) of **Ru^{H}-NCAr^{Cl}**.

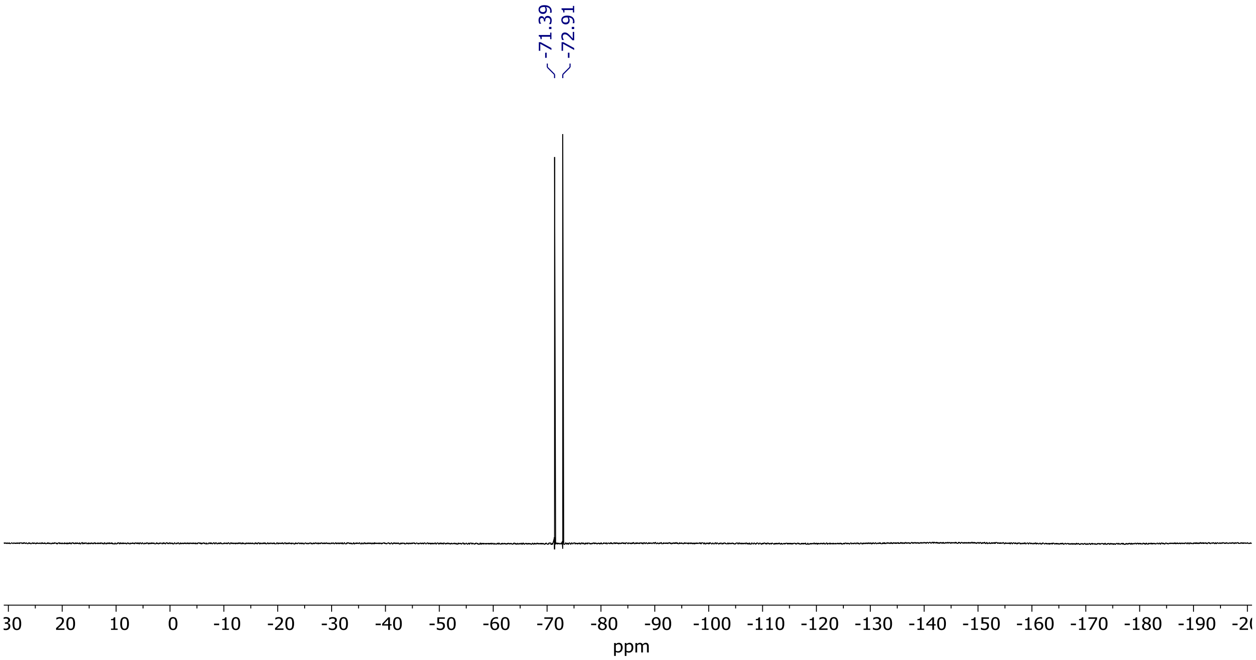

Figure S60. ^{19}F NMR (469 MHz, $CDCl_3$) of **Ru^{H}-NCAr^{Cl}**.

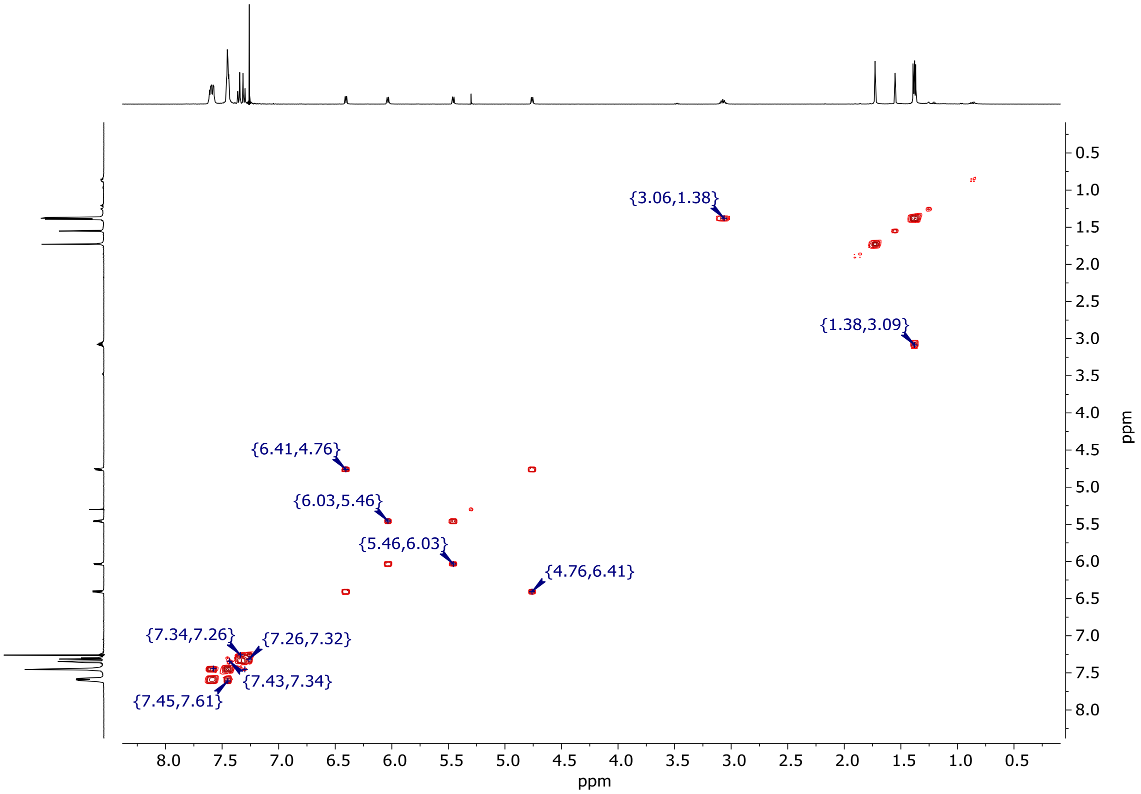

Figure S61. COSY spectrum (500 MHz, $CDCl_3$) of **Ru^{H}-NCAr^{Cl}** .

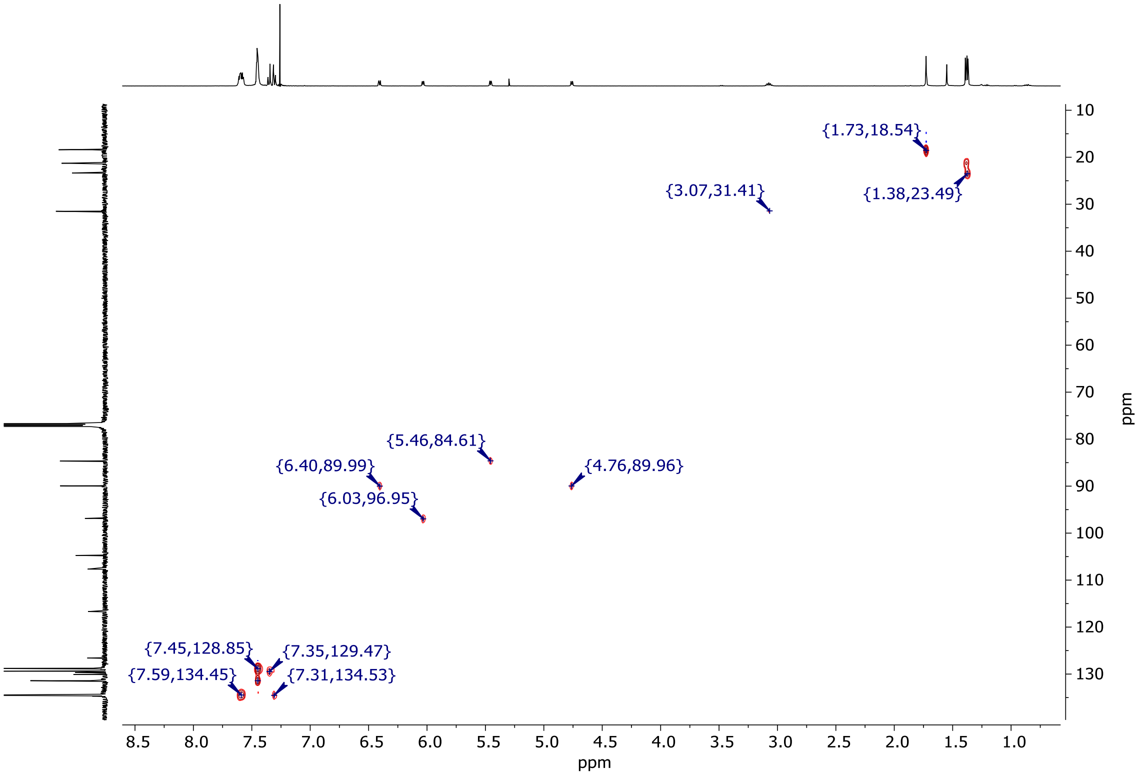

Figure S62. HSQC spectrum (500 MHz, $CDCl_3$) of **Ru^{H}-NCAr^{Cl}**.

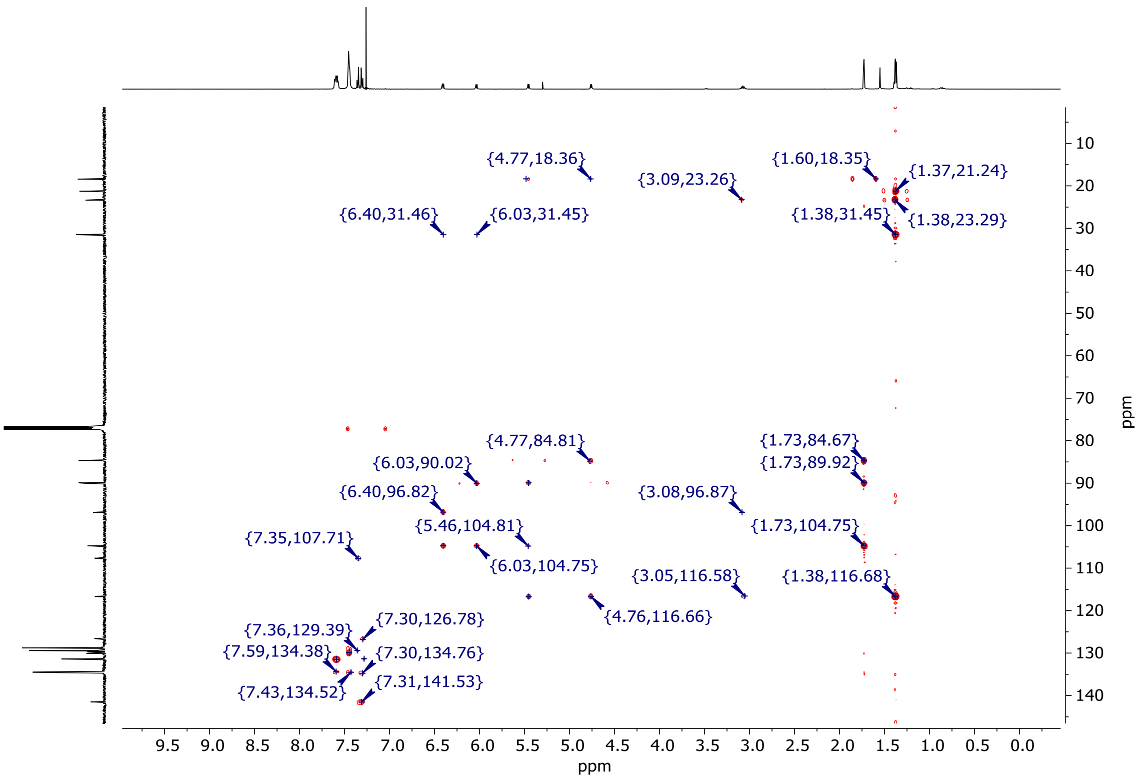

Figure S63. HMBC spectrum (500MHz, $CDCl_3$) of **Ru^{H}-NCAr^{Cl}**.

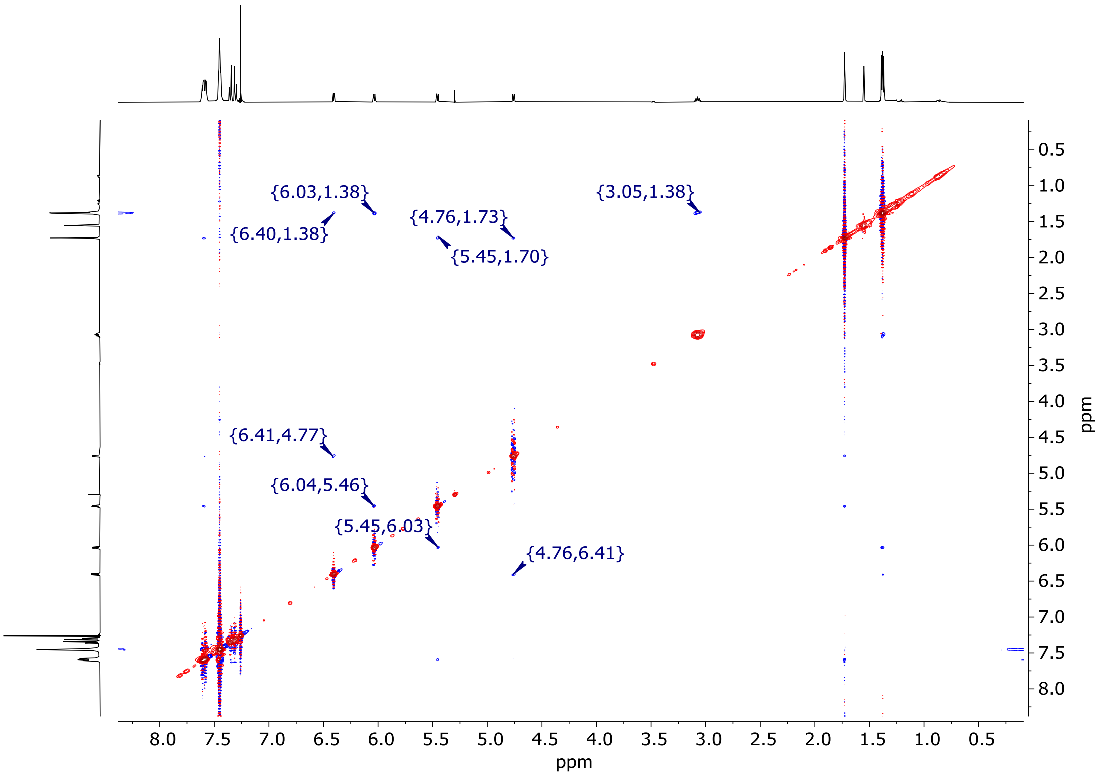

Figure S64. NOESY spectrum (500MHz, $CDCl_3$) of **Ru^{H}-NCAr^{Cl}**.

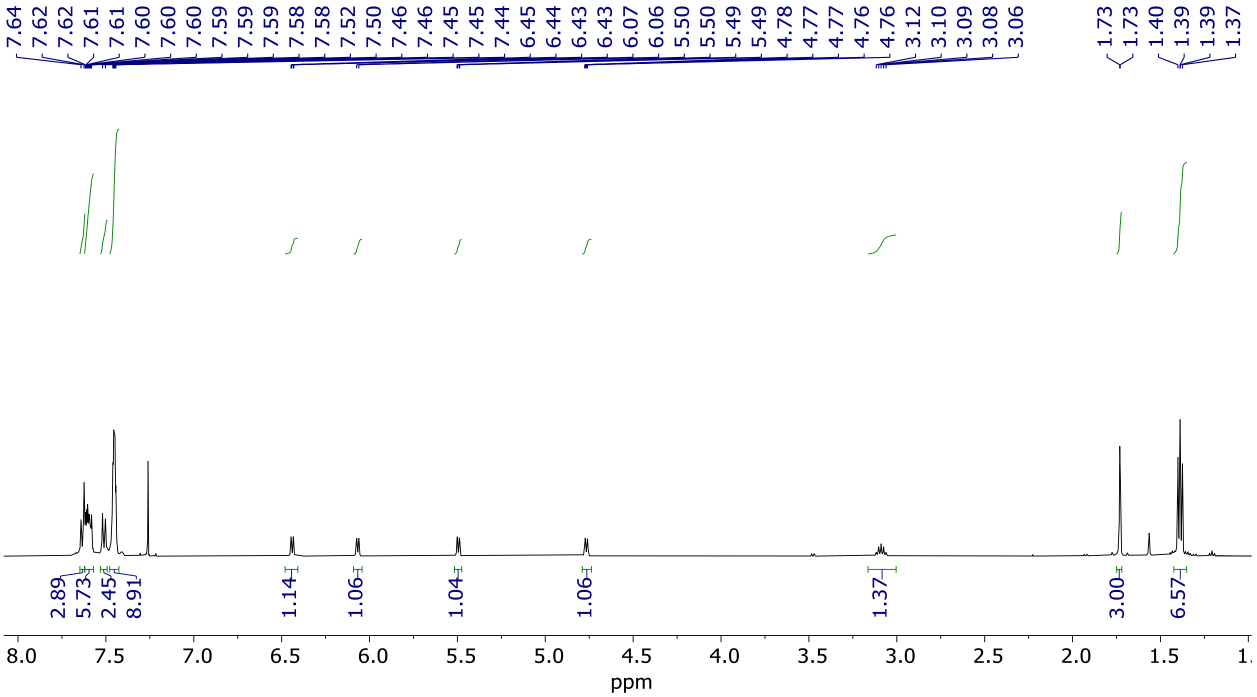

Figure S65. ^{1}H NMR (500 MHz, $CDCl_3$) of **Ru^{H}-NCAr^{CF3}**.

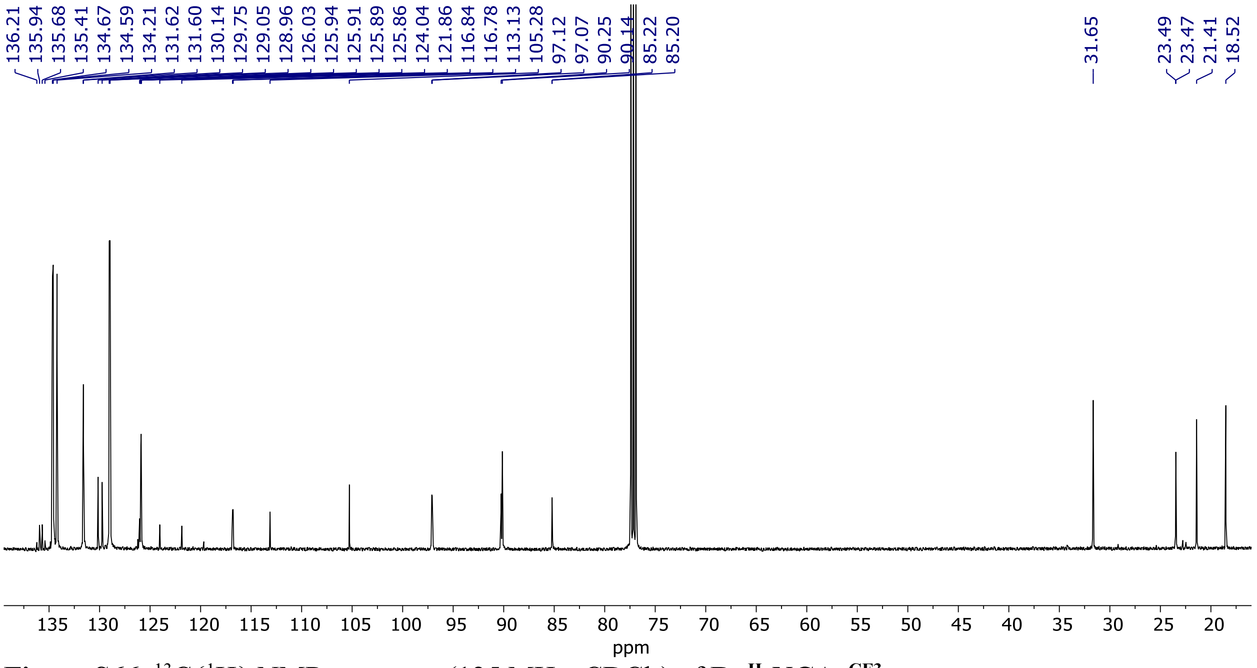

Figure S66. $^{13}C\{^{1}H\}$ NMR spectrum (125 MHz, $CDCl_3$) of **Ru^{H}-NCAr^{CF3}**.

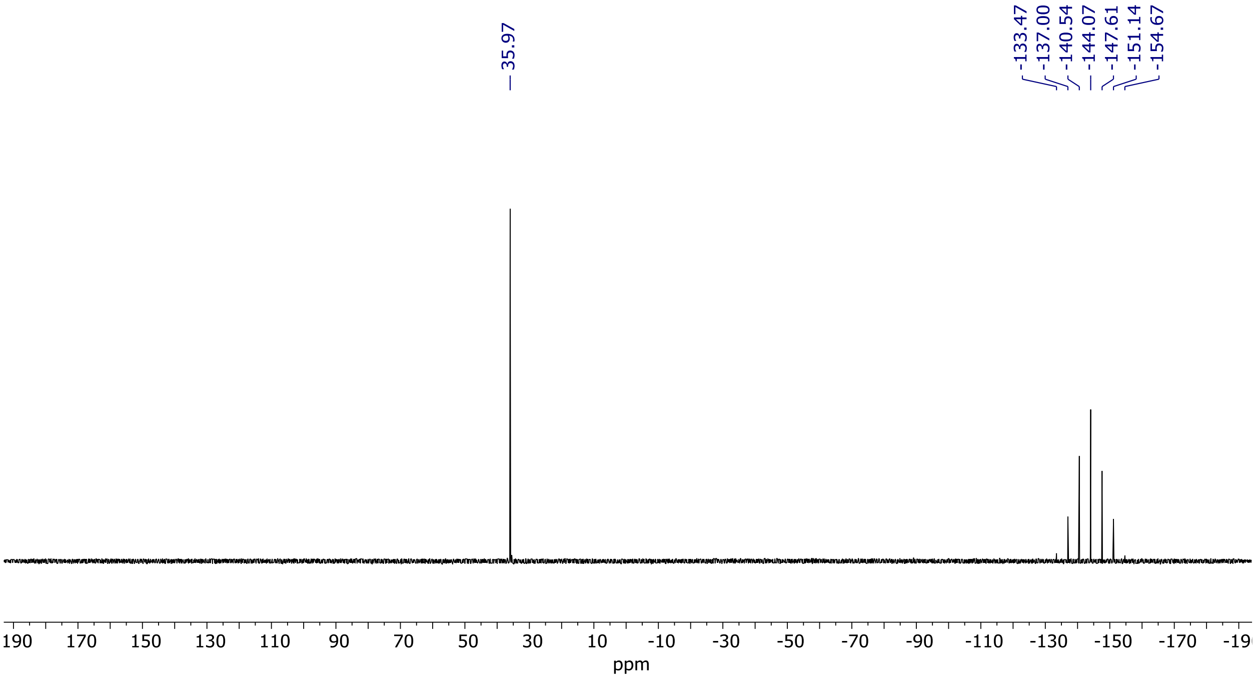

Figure S67. $^{31}P\{^{1}H\}$ NMR (202 MHz, $CDCl_3$) of **Ru^{H}-NCAr^{CF3}**.

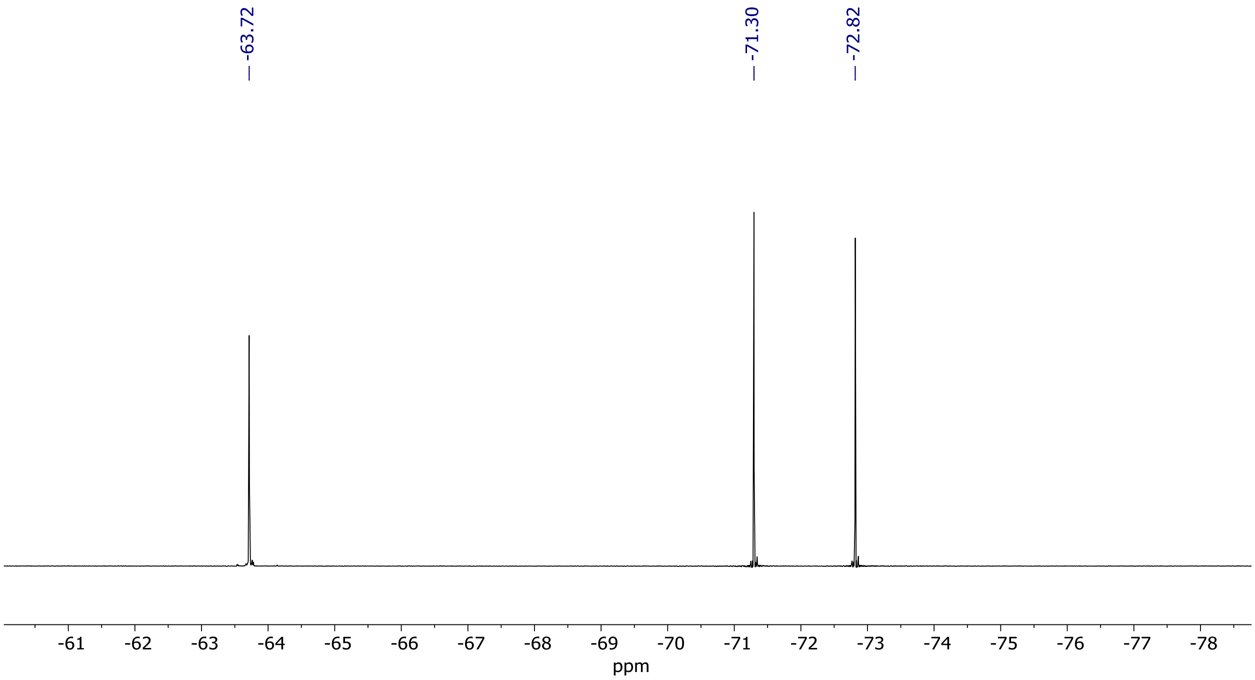

Figure S68. ^{19}F NMR (469 MHz, $CDCl_3$) of **Ru^{H}-NCAr^{CF3}**.

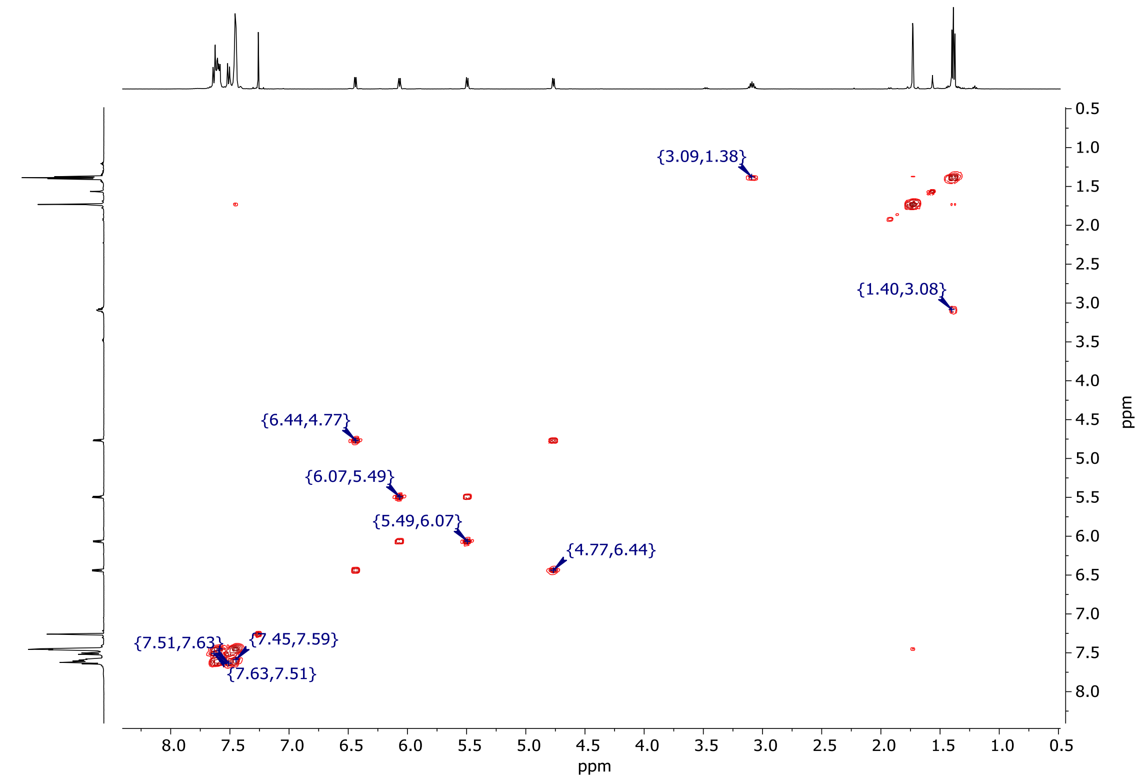

Figure S69. COSY spectrum (500 MHz, $CDCl_3$) of **Ru^{H}-NCAr^{CF3}**.

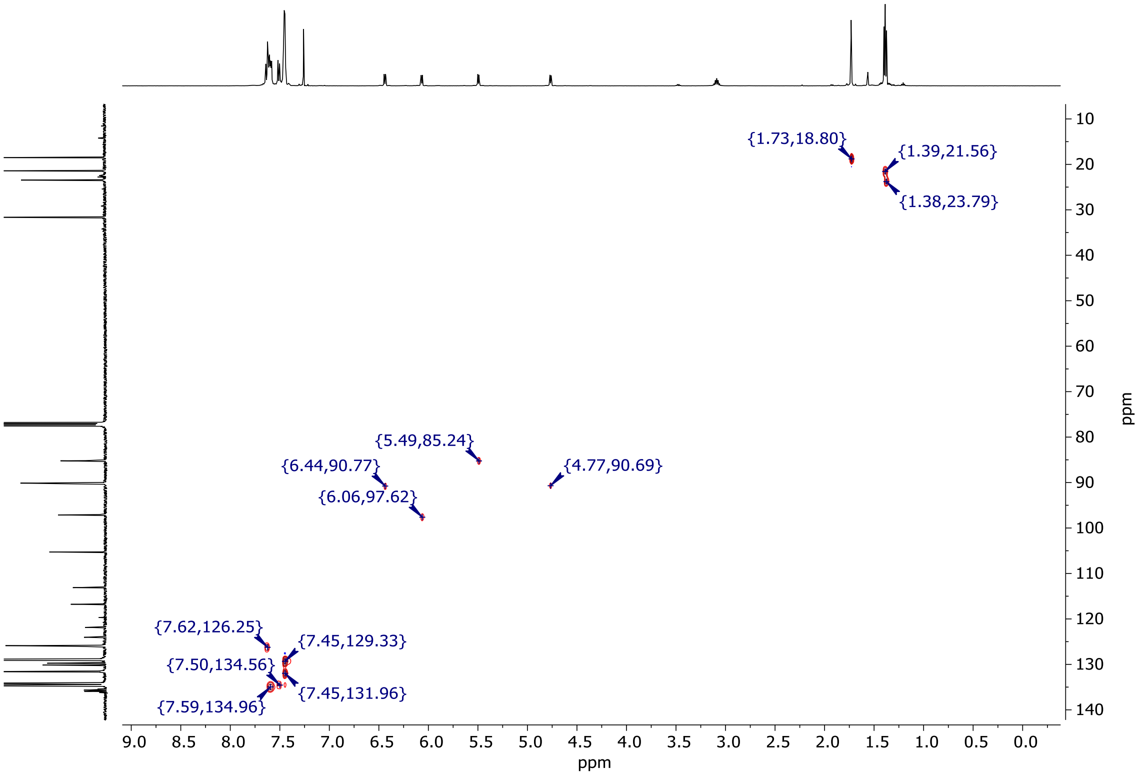

Figure S70. HSQC spectrum (500 MHz, $CDCl_3$) of **Ru^{H}-NCAr^{CF3}**.

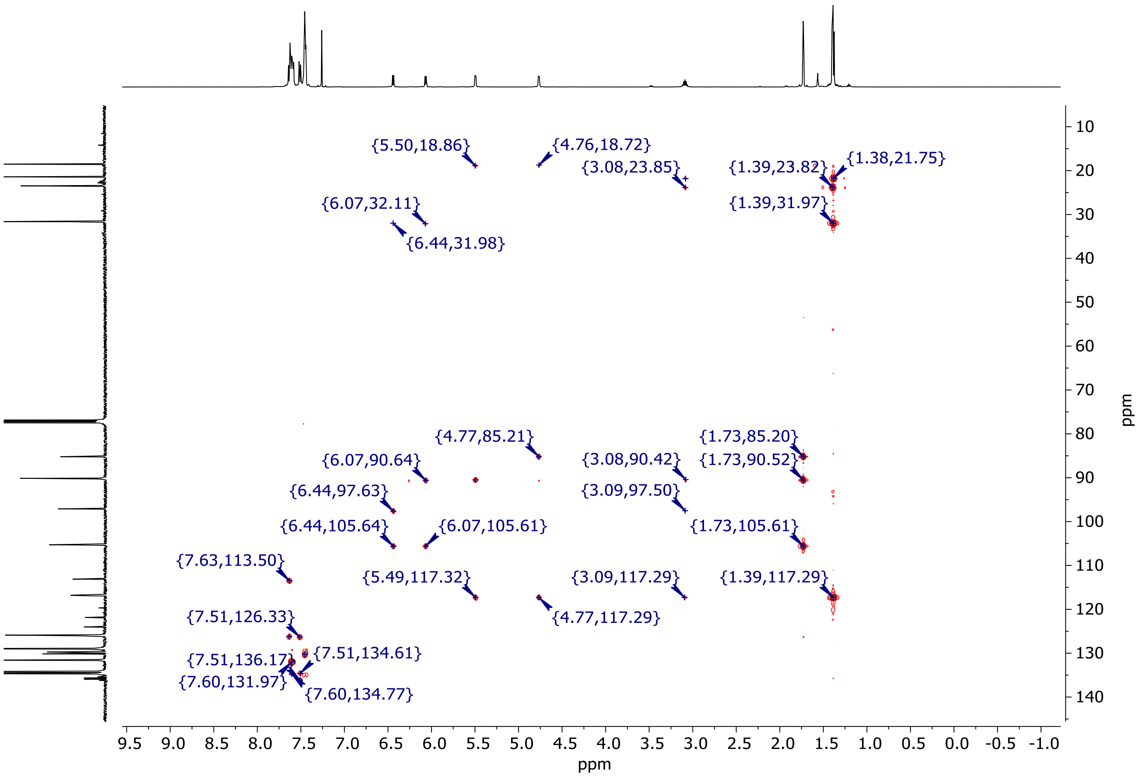

Figure S71. HMBC spectrum (500MHz, $CDCl_3$) of **Ru^{H}-NCAr^{CF3}**.

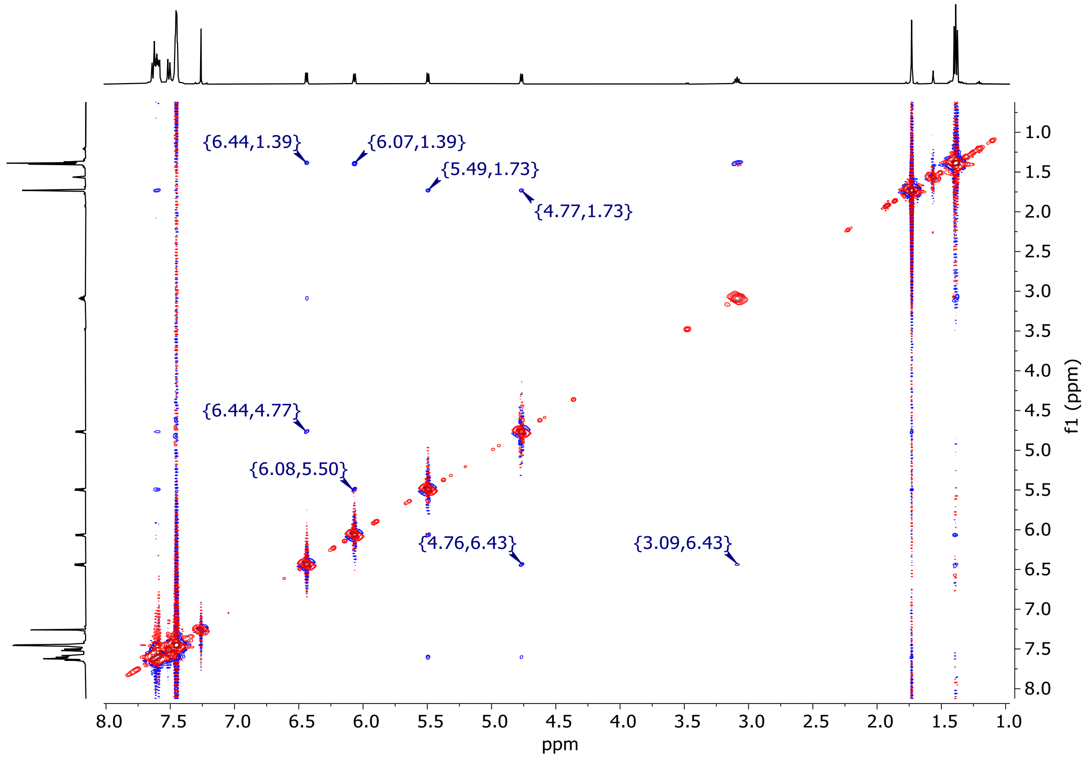

Figure S72. NOESY spectrum (500MHz, $CDCl_3$) of **Ru^{H}-NCAr^{CF3}**.

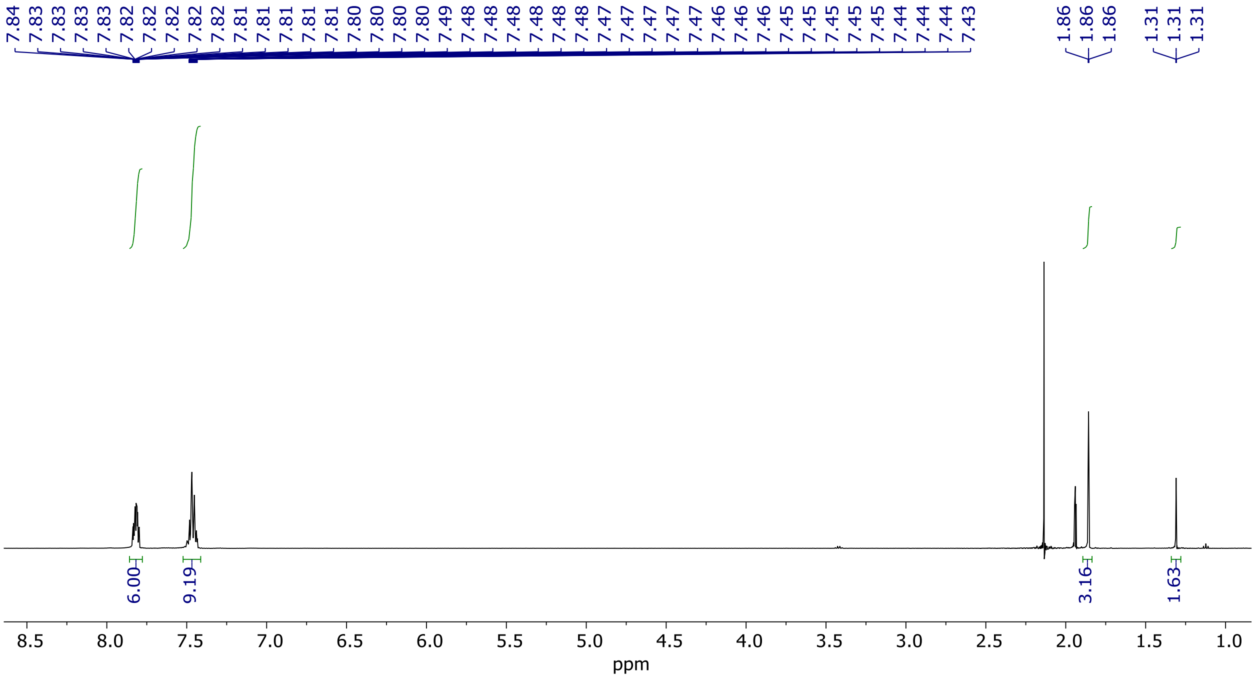

Figure S73. ^{1}H NMR (500 MHz, $CDCl_3$) of *trans*-*mer*-**C^{H}-NCCH$_3$**.

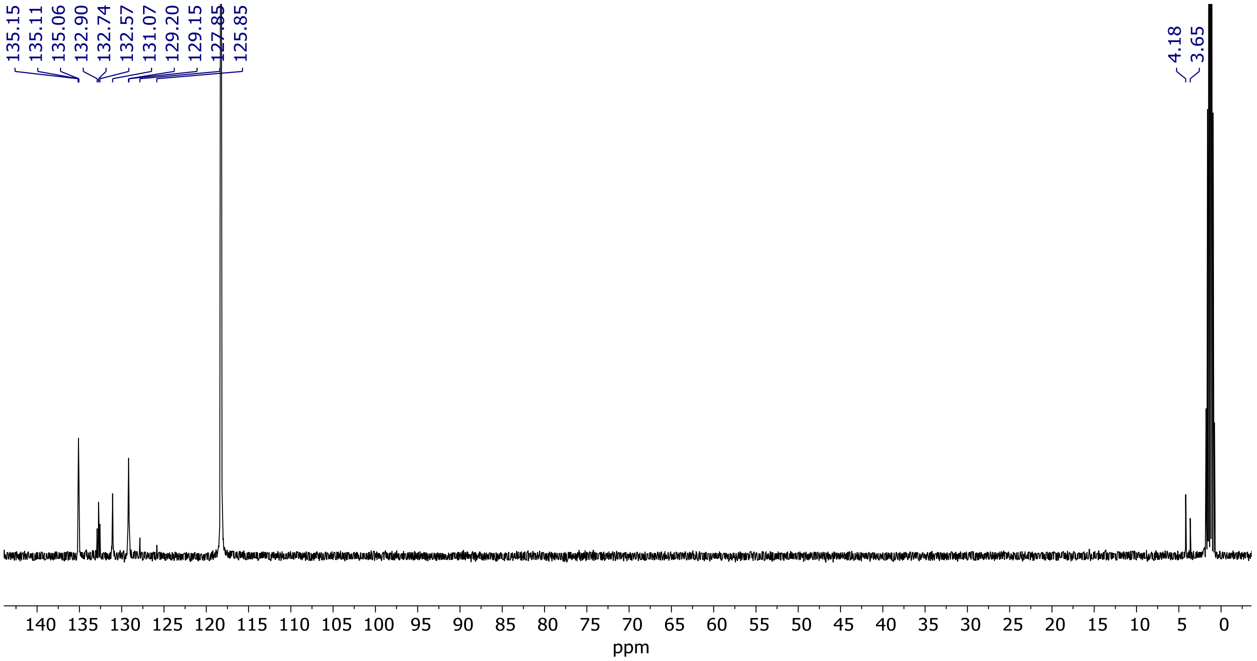

Figure S74. $^{13}C\{^{1}H\}$ NMR spectrum (125 MHz, $CDCl_3$) of *trans*-*mer*-**C^{H}-NCCH$_3$**.

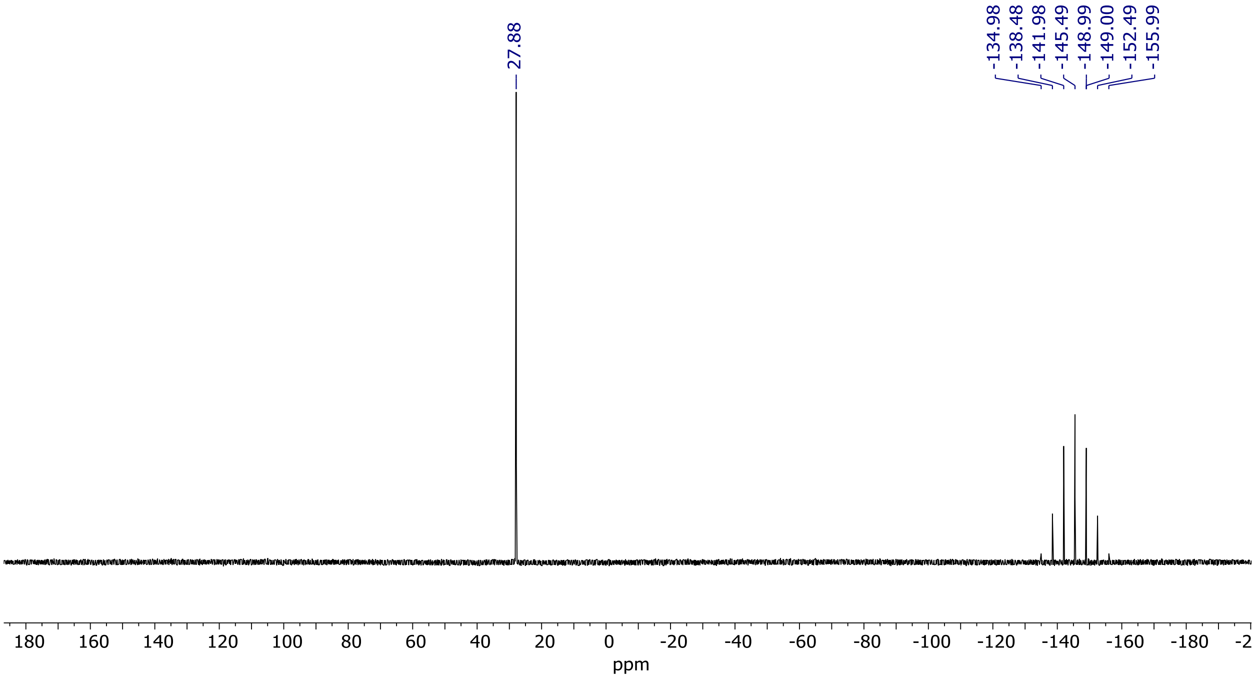

Figure S75. $^{31}P\{^{1}H\}$ NMR (202 MHz, $CDCl_3$) of *trans*-*mer*-**C^{H}-NCCH$_3$**.

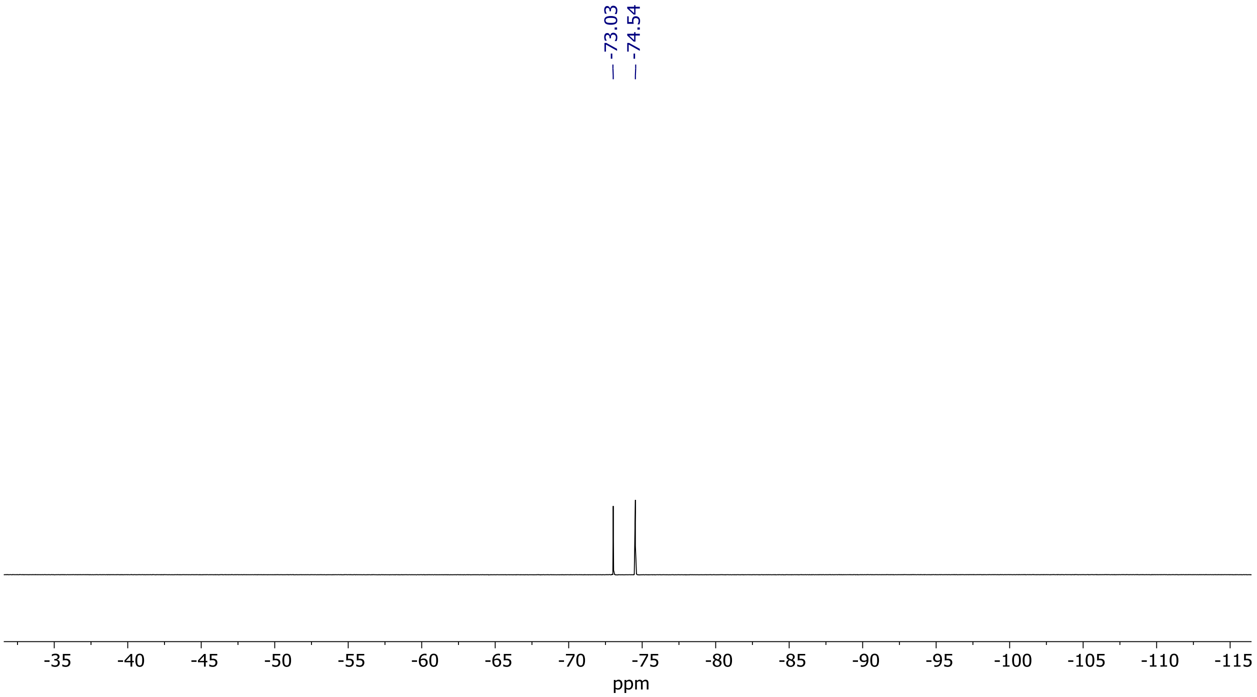

Figure S76. ^{19}F NMR (469 MHz, $CDCl_3$) of *trans*-*mer*-**C^{H}-NCCH$_3$**.

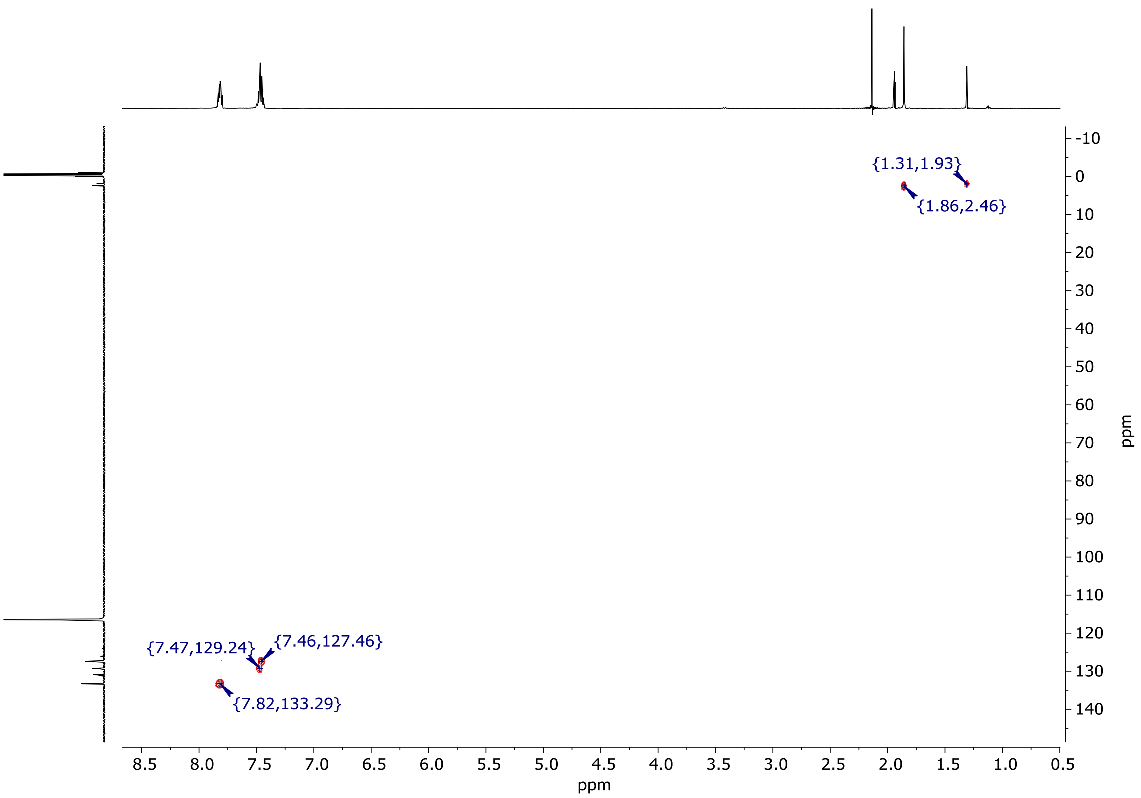

Figure S77. HSQC spectrum (500 MHz, $CDCl_3$) of *trans*-*mer*-**C^H^-NCCH~3~**.

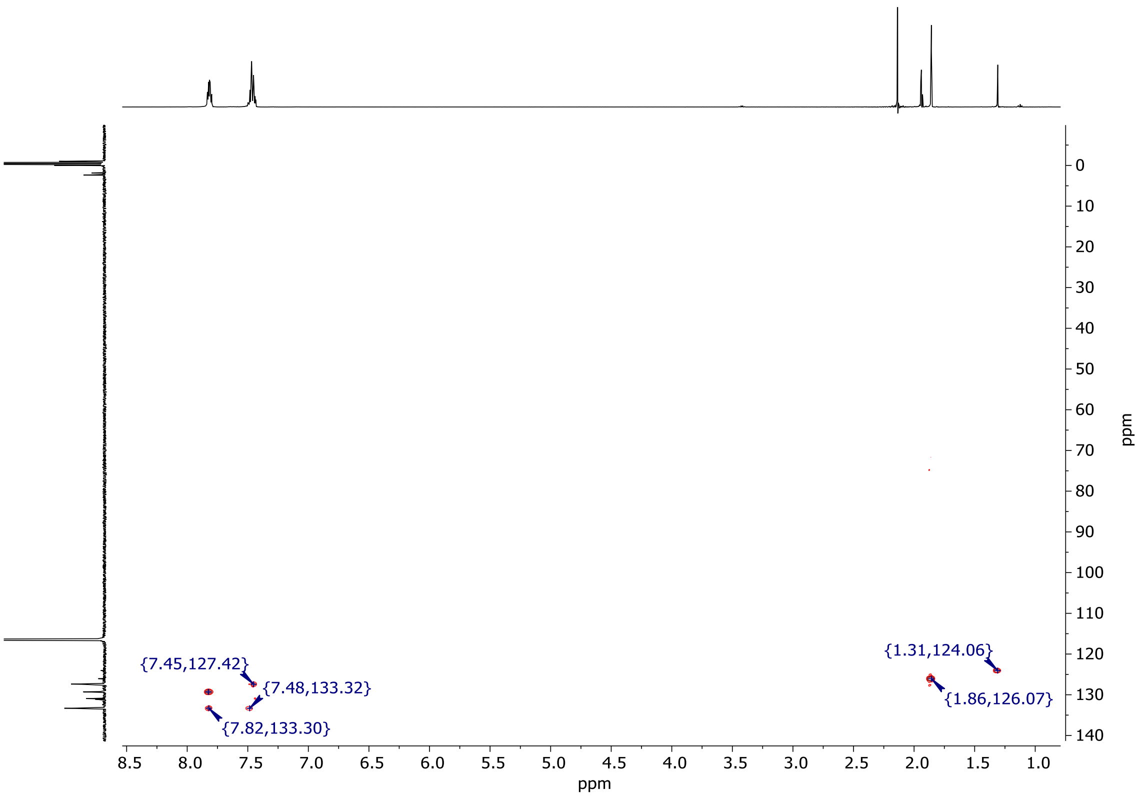

Figure S78. HMBC spectrum (500MHz, $CDCl_3$) of *trans*-*mer*-**C^H-$NCCH_3$**.

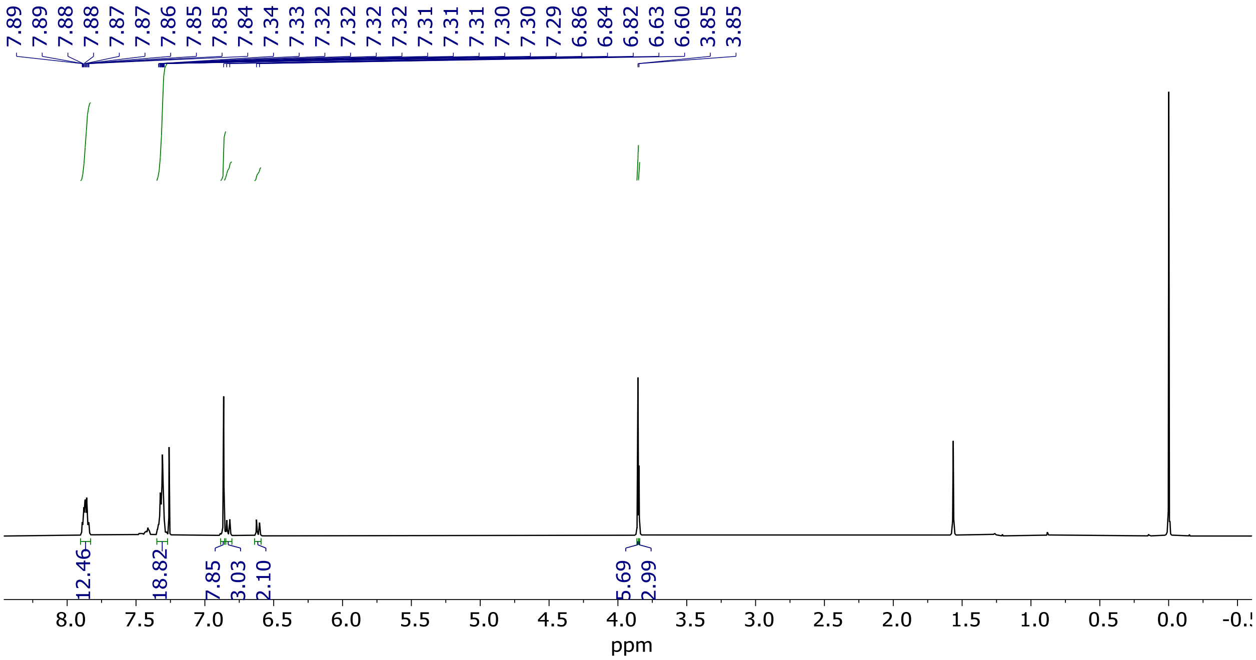

Figure S79. ^{1}H NMR (400 MHz, $CDCl_3$) of *trans*-*mer*-**C^{H}-NCAr^{OMe}**.

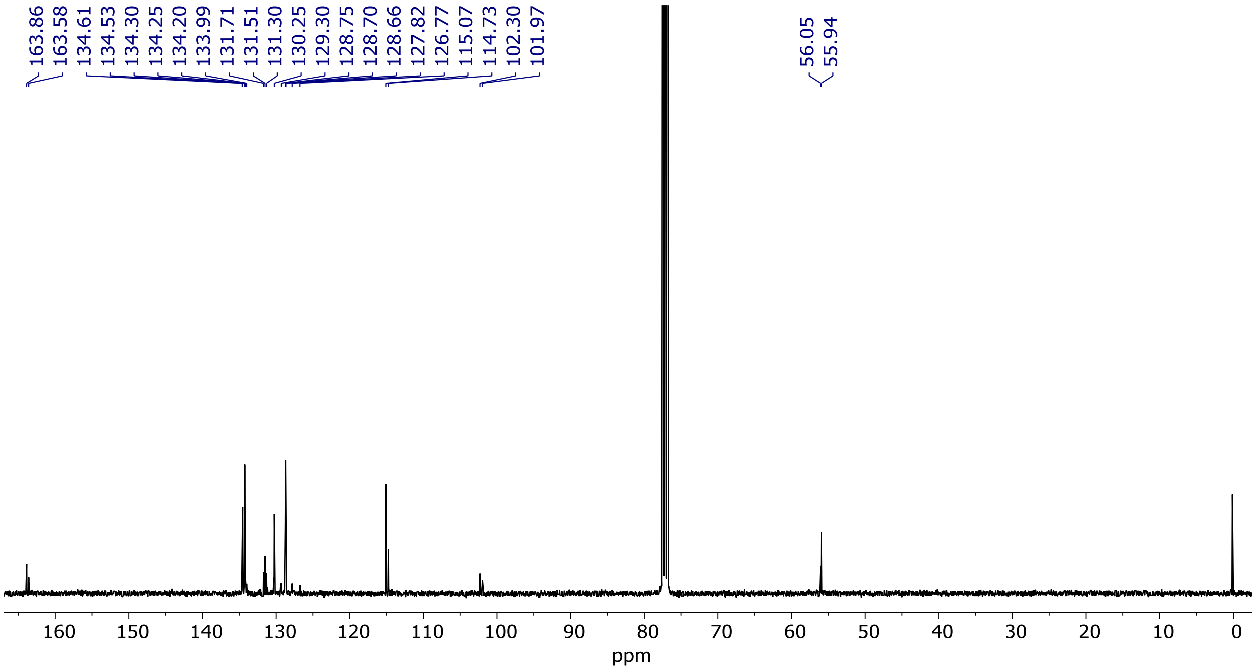

Figure S80. $^{13}C\{^{1}H\}$ NMR spectrum (101 MHz, $CDCl_3$) of *trans*-*mer*-**C^{H}-NCAr^{OMe}**.

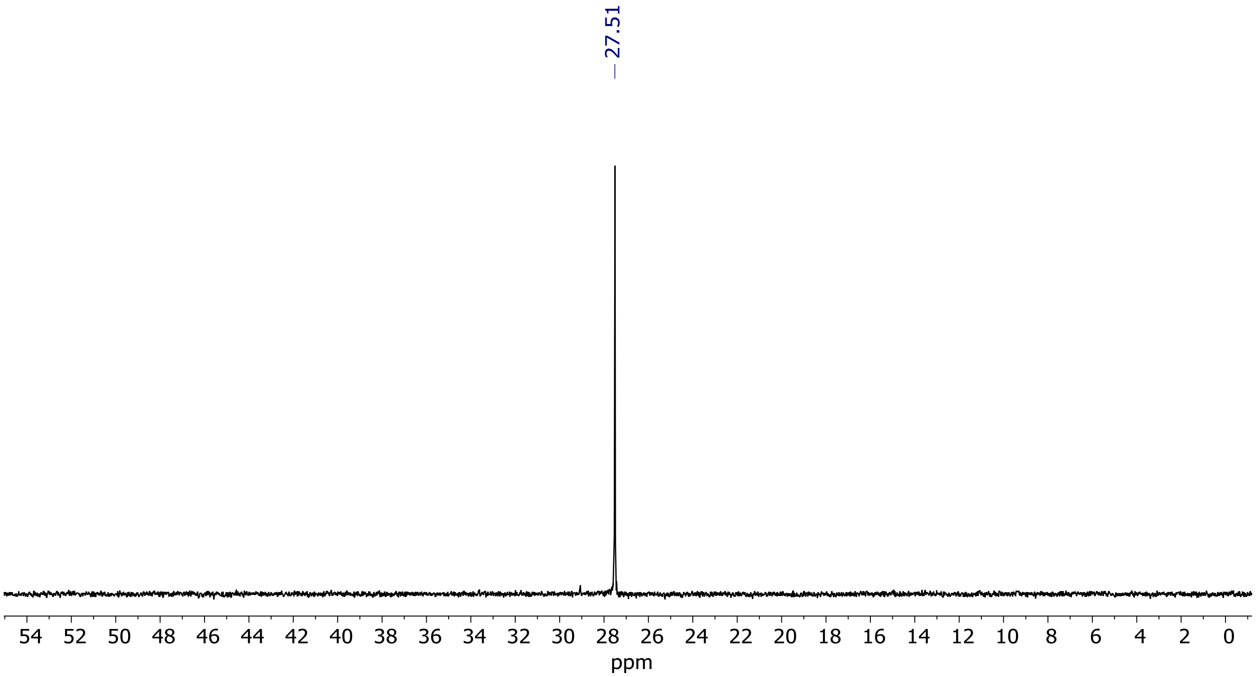

Figure S81. $^{31}P\{^{1}H\}$ NMR (162 MHz, $CDCl_3$) of *trans*-*mer*-**C^H-NCAr^OMe**.

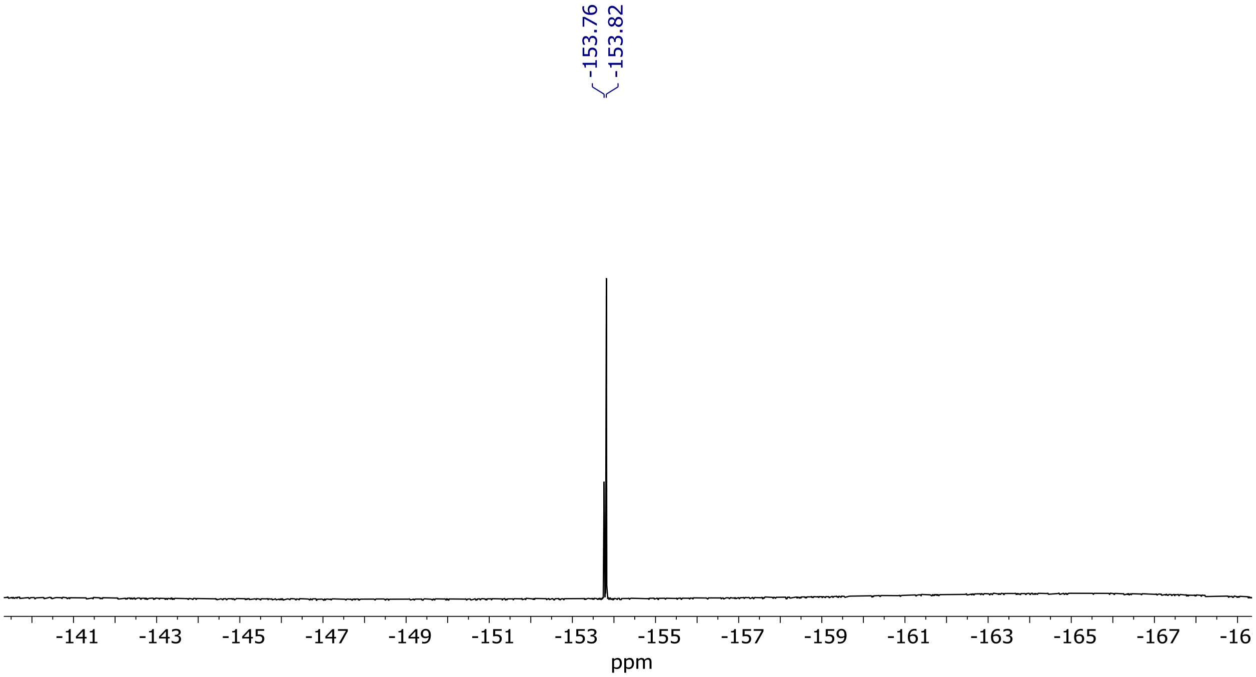

Figure S82. ^{19}F NMR (376 MHz, $CDCl_3$) of *trans*-*mer*-**C^H-NCAr^OMe**.

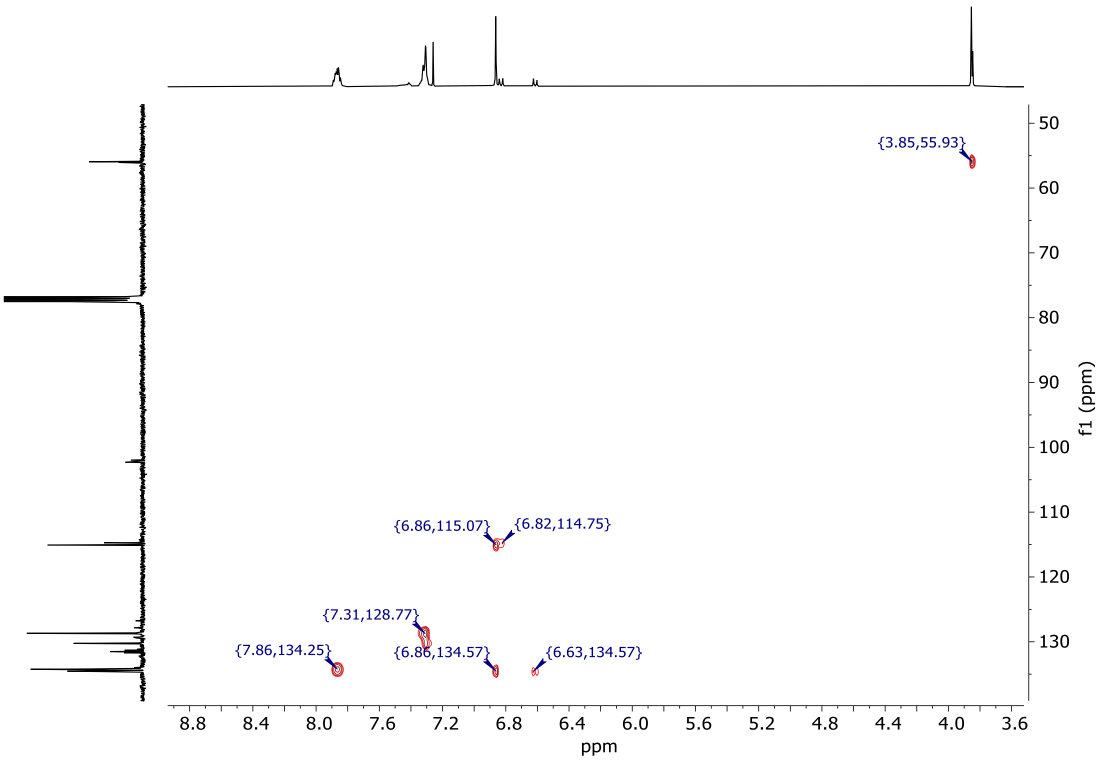

Figure S83. HSQC spectrum (400 MHz, $CDCl_3$) of *trans-mer*-**C^{H}-NCAr^{OMe}**.

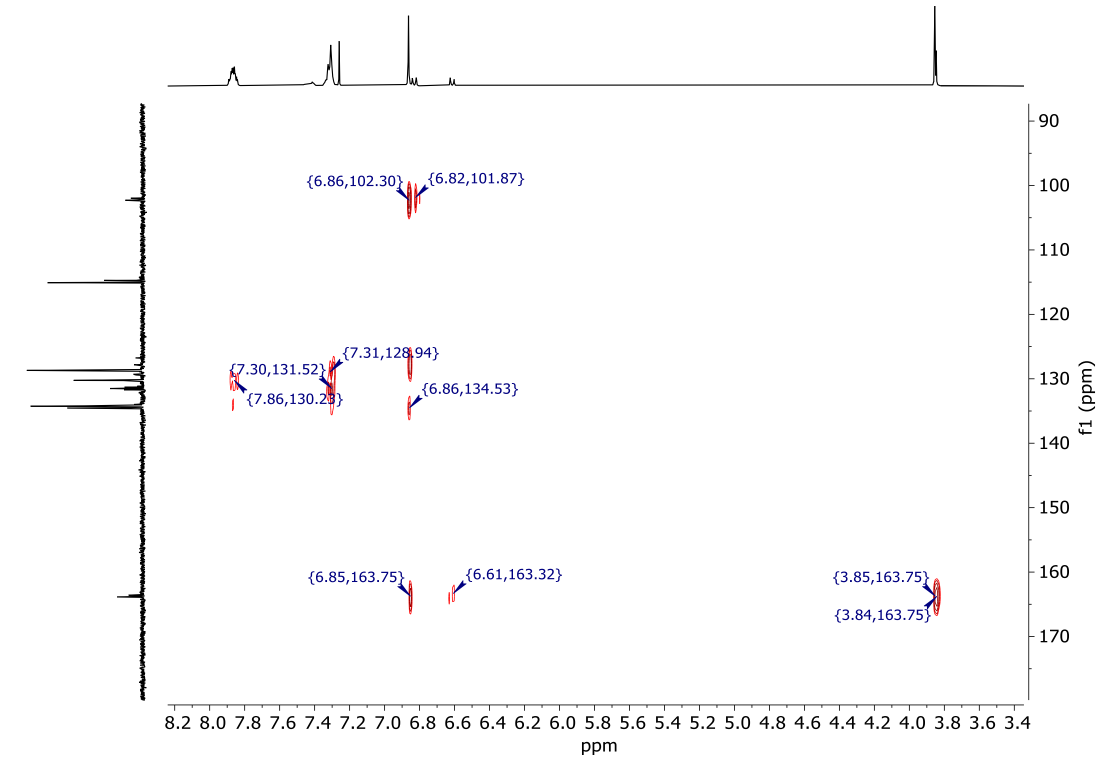

Figure S84. HMBC spectrum (400MHz, $CDCl_3$) of *trans-mer*-**C^{H}-NCAr^{OMe}**.

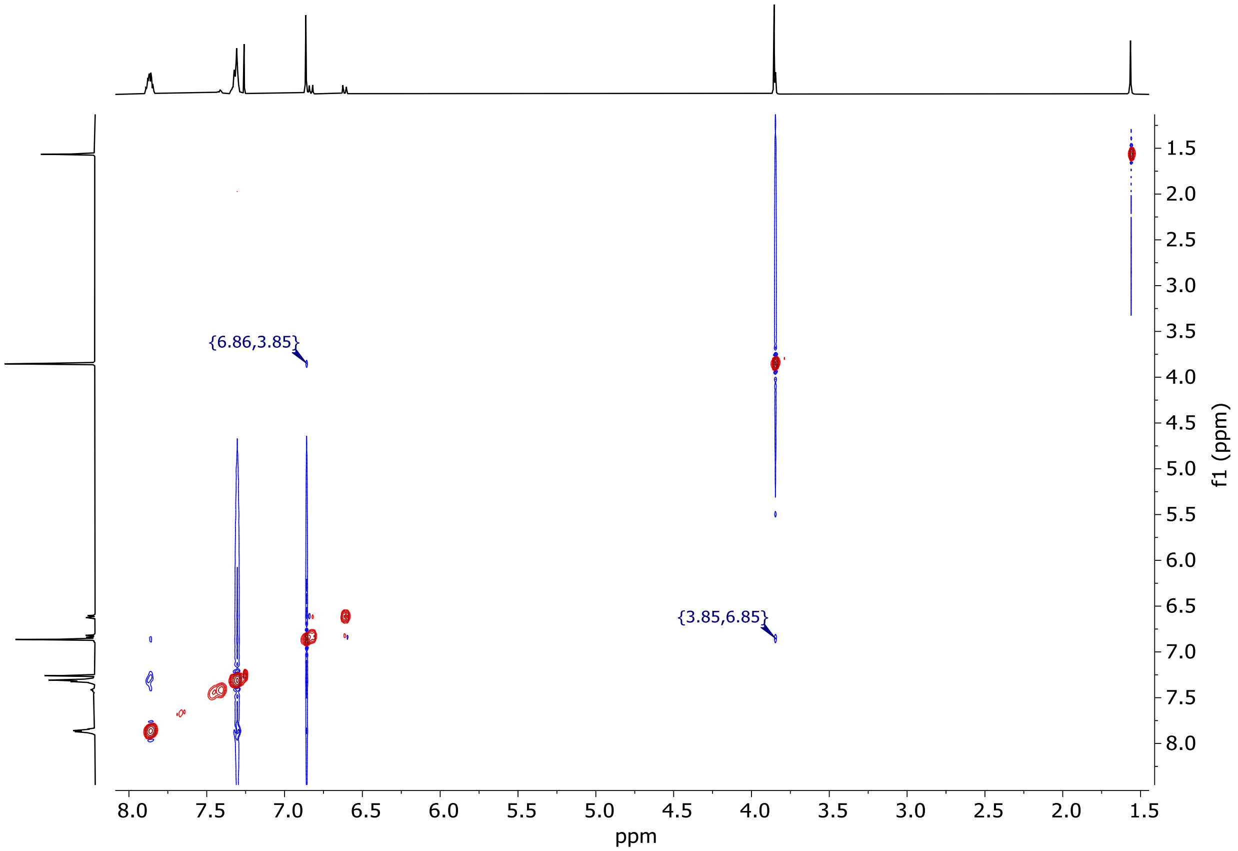

Figure S85. NOESY spectrum (400MHz, $CDCl_3$) of *trans*-*mer*-**C^{H}-NCAr^{OMe}**.

Stability studies of complexes ***Ru^{H}-NCCH$_3$*** *and* ***Ru^{H}-PAr$_3$****.*

Dichloride **Ru^{X}-Cl** complexes convert to several products over a timeframe of hours to days in either chlorinated ($CDCl_3$) or coordinating (CD_3CN) solvents, with the rate of decomposition being accelerated when samples were stored in ambient light.[1] Stability studies for complexes **Ru^{H}-NCCH$_3$** and **Ru^{H}-PAr$_3$** revealed a less uniform picture; solvento complex **Ru^{H}-NCCH$_3$** appeared fairly stable in solution (Figure S86), with only minimal byproducts (including **Ru^{H}-Cl**) being formed in $CDCl_3$ over several days (though reactivity of the PF_6^{-} counteranion was observed under these conditions). This reactivity appeared relatively independent of exposure to light. Addition of trace HCl did not dramatically increase the rate of decomposition, leading to formation of only marginally more **Ru^{H}-Cl** over several days. In CD_3CN, only a minor amount of free *p*-cymene was generated from **Ru^{H}-NCCH$_3$** over several days when samples were exposed to ambient light, while no evidence for decomposition was observed under exclusion of light. This is in contrast with the recently reported behavior of $[(p\text{-cymene})RuCl(L)P(OMe)_3]^{+}$ complexes (L = EtCN, CH_3CN),[2] which undergo dissociation of cymene to generate tetrakis(nitrile) complexes in CD_3CN over comparable timeframes. This observation indicates increased stability of the Ru–cymene interaction in the presence of triarylphosphine ligands vs. their phosphite analogues.

For bisphosphine complex **Ru^{H}-PAr$_3$** (Figure S89), evidence of decomposition to multiple unidentified species was observed over several days in $CDCl_3$, with complete disappearance of the NMR signals associated with the complex and formation of free *p*-cymene over 10 days. Treatment of $CDCl_3$ with K_2CO_3 to remove any acidic species from the solvent appeared to slow down this reactivity, with most of the signals corresponding to **Ru^{H}-PAr$_3$** still observed at 10 days (though again with decrease in intensity of the PF_6^{-} signals occasionally observed; Figures S90-S91). Addition of trace HCl did result in greater decomposition over the same time frame, consistent with participation of acidic species in decomposition of this complex (Figure S92). In CD_3CN, **Ru^{H}-PAr$_3$** is instead converted to its solvento analog **Ru^{H}-NCCH$_3$** over shorter time frames (24-48 hours), with the rate of conversion being slightly faster rate in ambient light vs. in the dark (Figures S93-94). In these experiments, signals corresponding to free *p*-cymene as well as *trans-mer-* **C^{H}-NCCH$_3$** were observed. This reactivity appears to require both the presence of free PPh_3 as well as ambient light, as further reaction between solvento **Ru^{H}-NCCH$_3$** and PPh_3 (generated from decomposition of **Ru^{H}-PAr$_3$**) was not observed under exclusion of light.

While prone to decomposition in solution, complexes **Ru^{X}-Cl** are highly stable in the solid state, with no evidence of decomposition over several years, even in the presence of air, water, and/or light. Instead, samples of complexes **Ru^{X}-NCCH$_3$** and **Ru^{X}-PAr$_3$** discolor over periods of weeks to months, especially when exposed to ambient atmosphere. While solution stability is not significantly different for **Ru^{X}-NCCH$_3$/Ru^{X}-PAr$_3$** complexes vs. their **Ru^{X}-Cl** analogues (except for rapid conversion of **Ru^{H}-PAr$_3$** to **Ru^{H}-NCCH$_3$** in CD_3CN), the substantially reduced stability in the solid state is consistent with increased reactivity of cationic **Ru^{X}-NCCH$_3$/ Ru^{X}-PAr$_3$**. This observation correlates with the relative rates of the electrochemically induced chemical reactions of complexes **Ru^{X}-L**, with cationic complexes displaying faster reaction with CH_3CN following oxidation to Ru(III).

Characterization of bis(phosphine) and solvento complexes also provided new insight into our earlier stability studies on dichloride complexes **Ru^{X}-Cl**.[1] In particular, comparison of spectroscopic data obtained for **Ru^{X}-NCCH$_3$** complexes to that obtained from stability studies for complexes **Ru^{X}-Cl** revealed that dichloride complexes in this family frequently generate solvento species over time when exposed to acetonitrile; these results highlight the propensity of these complexes to undergo ligand substitution processes even at the Ru(II) oxidation state, which has been proposed as one of the key steps in the biological activation mechanism of these compounds.[3]

To probe the relevance of this reactivity to electrochemical investigations, a solution of **Ru^{H}-Cl** in CH_3CN (0.1 *M* $[^{n}Bu_4][PF_6]$ in CH_3CN, [Ru] ≈ 2 m*M*) was monitored over time via both spectroscopic and voltammetric methods. Generation of solvento complex **Ru^{X}-NCCH$_3$** was observed in ^{31}P NMR data (Figures S92), while CV analysis also indicated formation of a small amount of **B^{H}-NCCH$_3$**, the product of *p*-cymene loss from **Ru^{X}-NCCH$_3$**. These observations are consistent with accelerated conversion in the presence of the supporting $[^{n}Bu_4][PF_6]$ electrolyte, which may facilitate Cl^{-} dissociation due to increased solution polarity. These results emphasize the need for the use of freshly prepared solutions in analyses of

these compounds and highlight the importance of assessing speciation of these complexes in solutions that contain possible exogenous ligands (such as coordinating solvents). However, since no evidence for changes in the number of phosphine or chloride ligands has thus far been observed as a result of ET, generation of solvento or bis(phosphine) species does not appear to be relevant to the chemical reactivity observed following electrochemical oxidation.

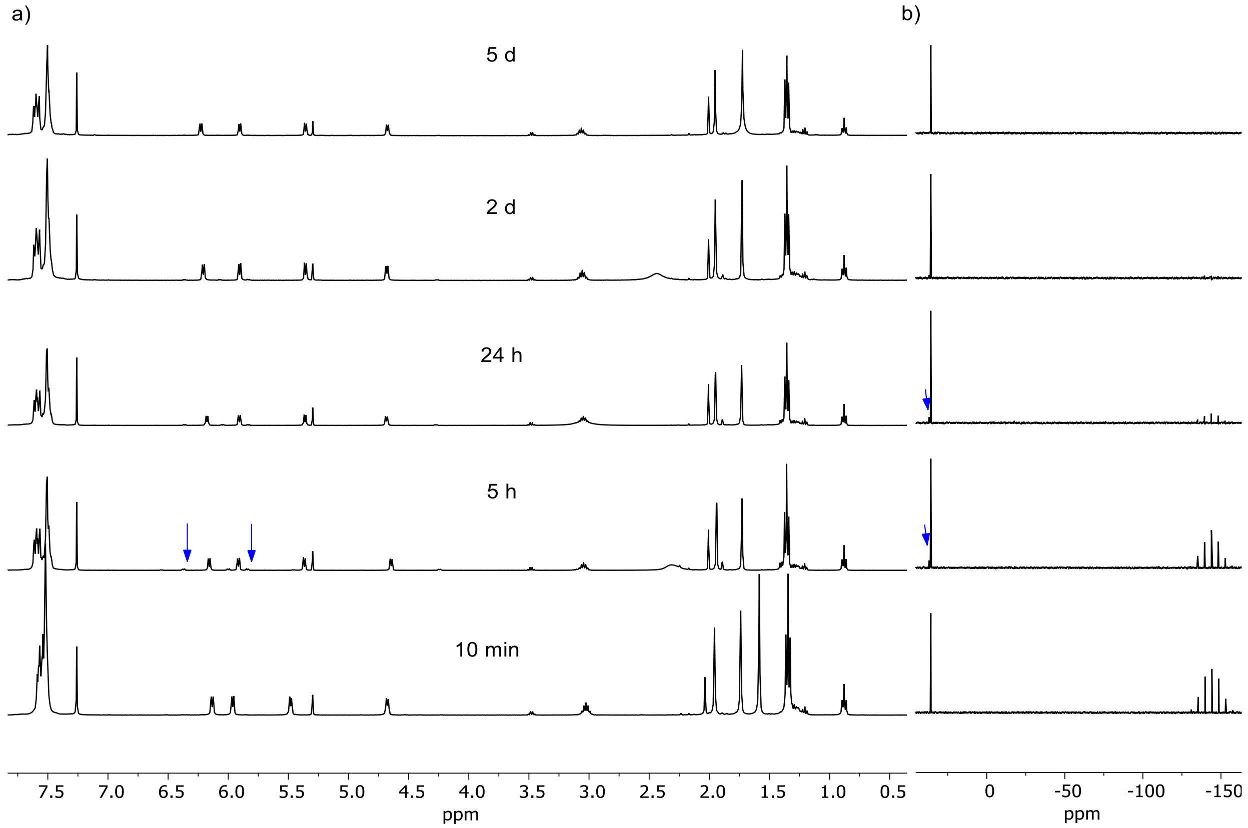

Figure S86. ^{1}H (a) and $^{31}P\{^{1}H\}$ (b) NMR spectra for complex **Ru^{H}-NCCH$_3$** in $CDCl_3$ over time. Decomposition to various species (blue arrows) is observed at early timepoints, though these impurities disappear at longer time points. Little to no free *p*-cymene is observed. Disappearance of the septet at –144 ppm in the ^{31}P NMR spectrum suggests decomposition of the PF_6^- counteranion.

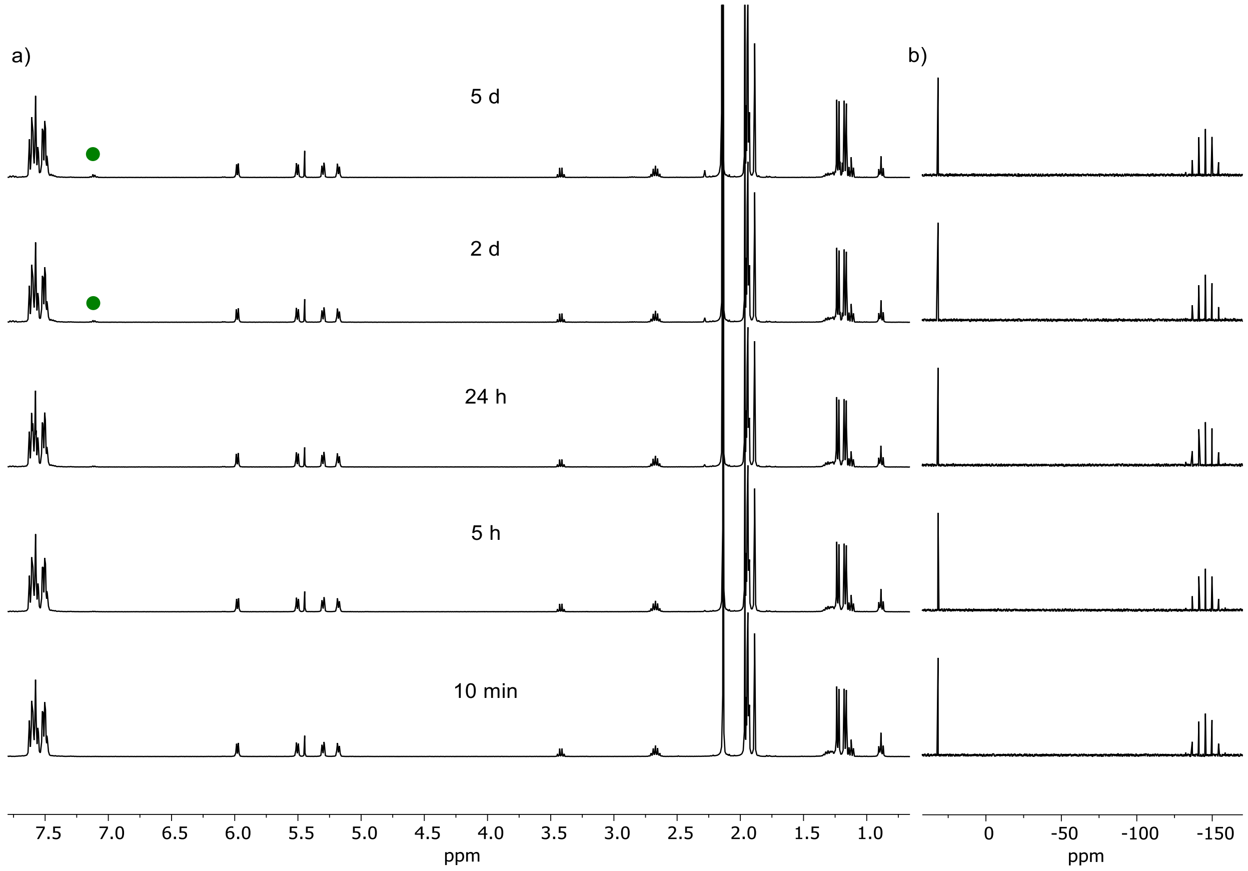

Figure S87. ^{1}H (a) and $^{31}P\{^{1}H\}$ (b) NMR spectra for complex **Ru^{H}-NCCH$_3$** in CD_3CN over time. At longer time points, formation of free *p*-cymene is observed (green circles).

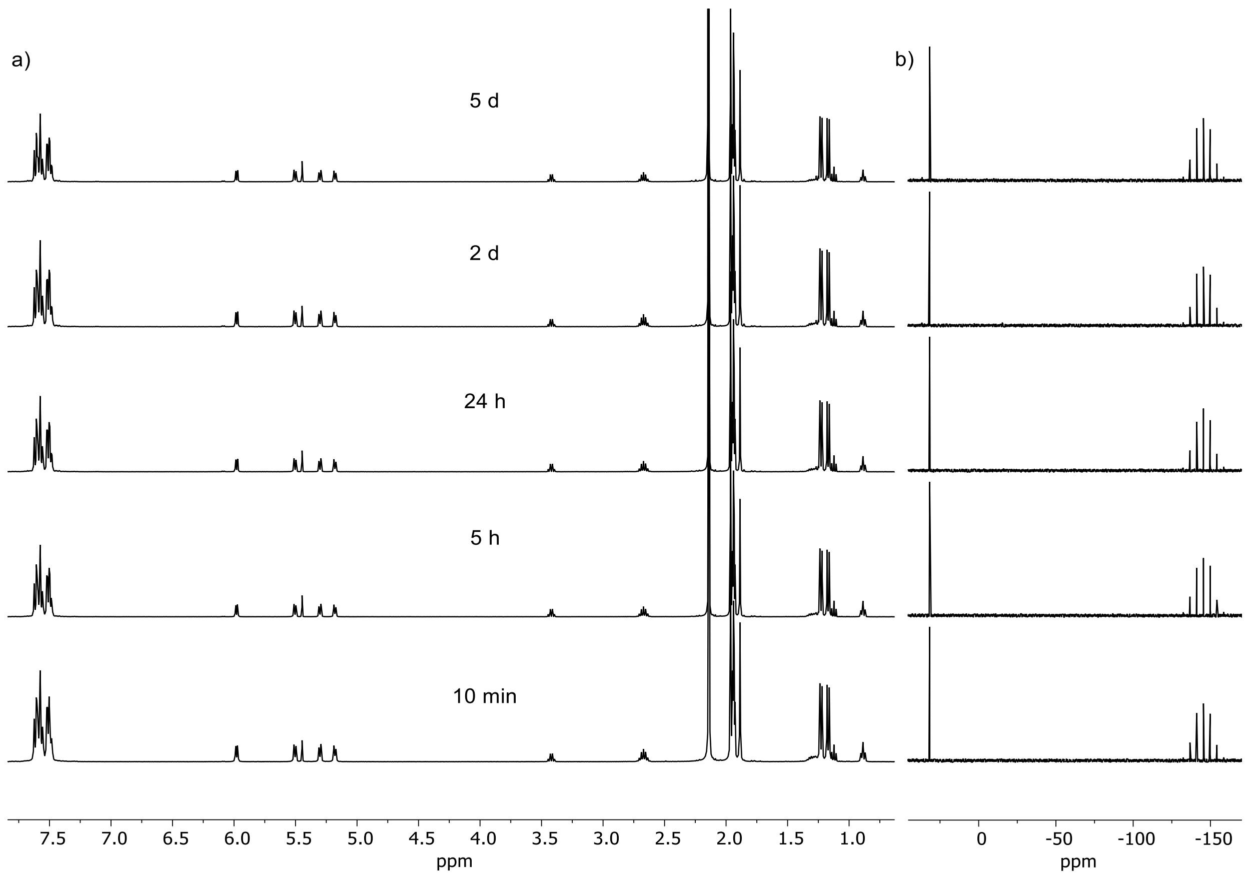

Figure S88. ^{1}H (a) and $^{31}P\{^{1}H\}$ (b) NMR spectra for complex **Ru^H-NCCH$_3$** in CD_3CN over time under exclusion of light. No evidence for the formation of free *p*-cymene is observed even at longer time points.

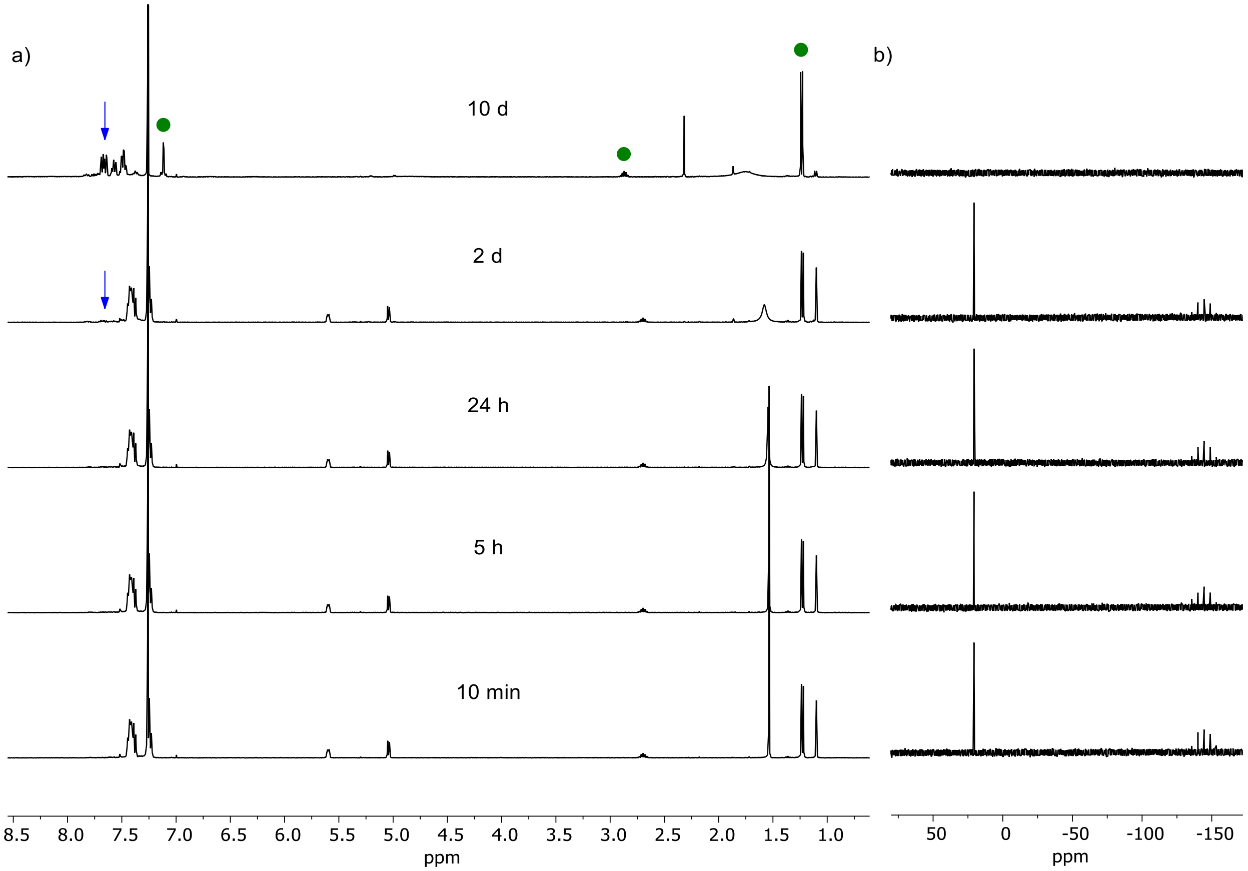

Figure S89. ^{1}H (a) and $^{31}P\{^{1}H\}$ (b) NMR spectra for complex **Ru^{II}-PAr$_3$** in $CDCl_3$ over time. Decomposition to various species (blue arrows, green circles) is observed. At long time points, free *p*-cymene is generated (green circles). Disappearance of signals in the ^{31}P NMR spectrum is postulated to be the consequence of generation of paramagnetic species.

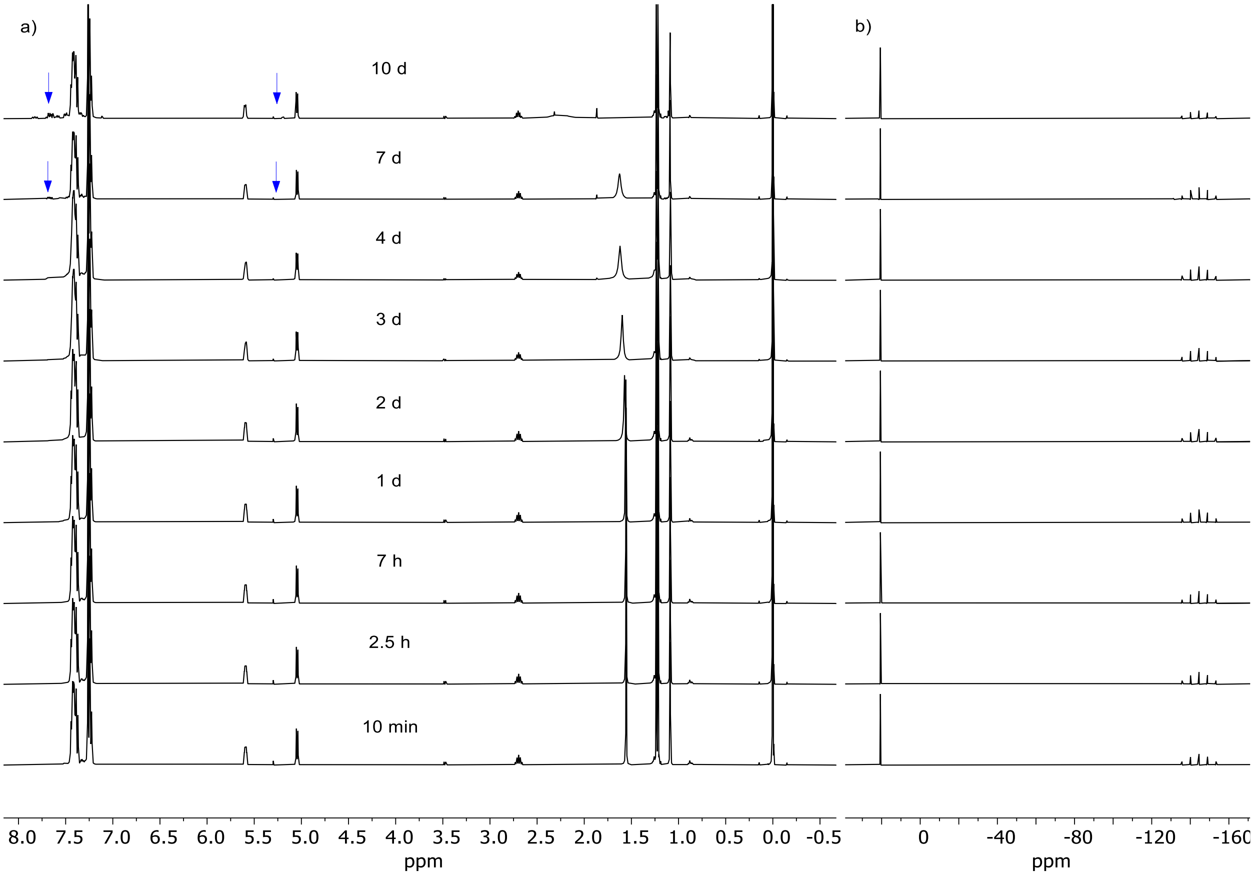

Figure S90. ^{1}H (a) and $^{31}P\{^{1}H\}$ (b) NMR spectra for complex **Ru^{H}-PAr$_3$** over time in $CDCl_3$ stored over K_2CO_3. Decomposition to various species (blue arrows) is slower than in untreated $CDCl_3$.

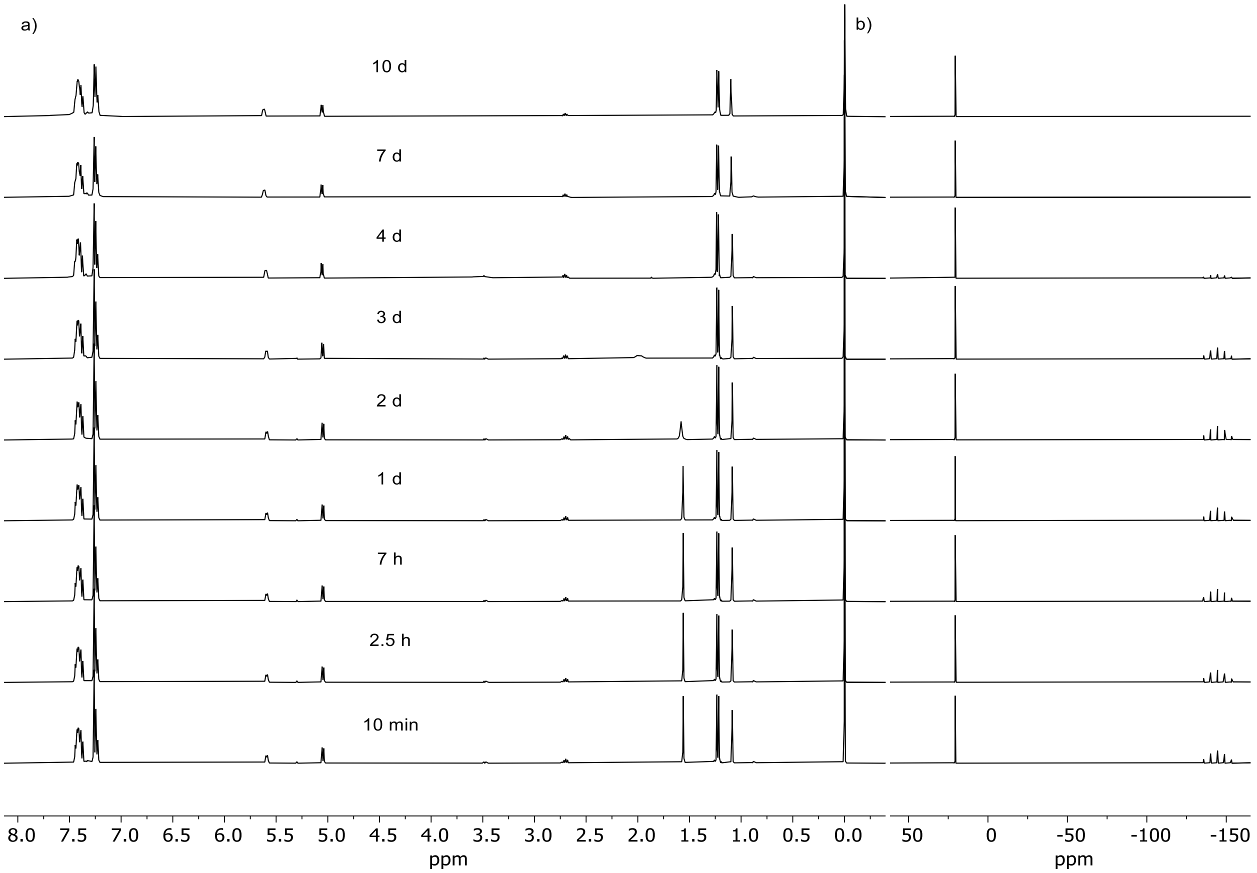

Figure S91. ^{1}H (a) and $^{31}P\{^{1}H\}$ (b) NMR spectra for complex **Ru^{H}-PAr$_3$** over time in $CDCl_3$ (stored over K_2CO_3) in the dark. Fewer Ru-containing byproducts are observed vs. samples stored under the same conditions in ambient light.

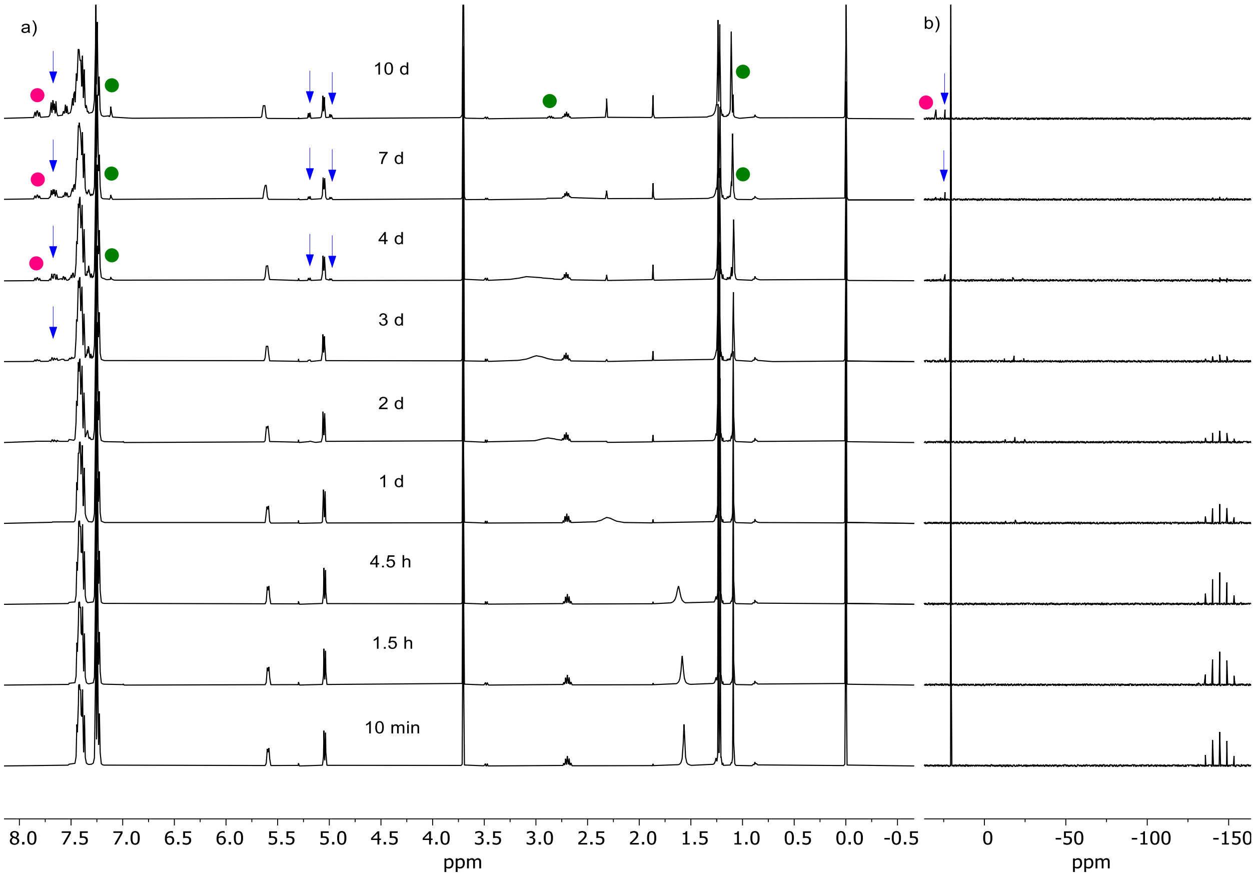

Figure S92. ^{1}H (a) and $^{31}P\{^{1}H\}$ (b) NMR spectra for complex **Ru^{H}-PAr$_3$** over time in $CDCl_3$ with 1 m*M* HCl (added as HCl/dioxane solution). Decomposition to various species is faster than in the absence of acid. Pink circles correspond to the signals for *trans*-*mer*-**C^{H}-NCCH$_3$** (see Figures S73 and S75). Green circles correspond to free *p*-cymene.

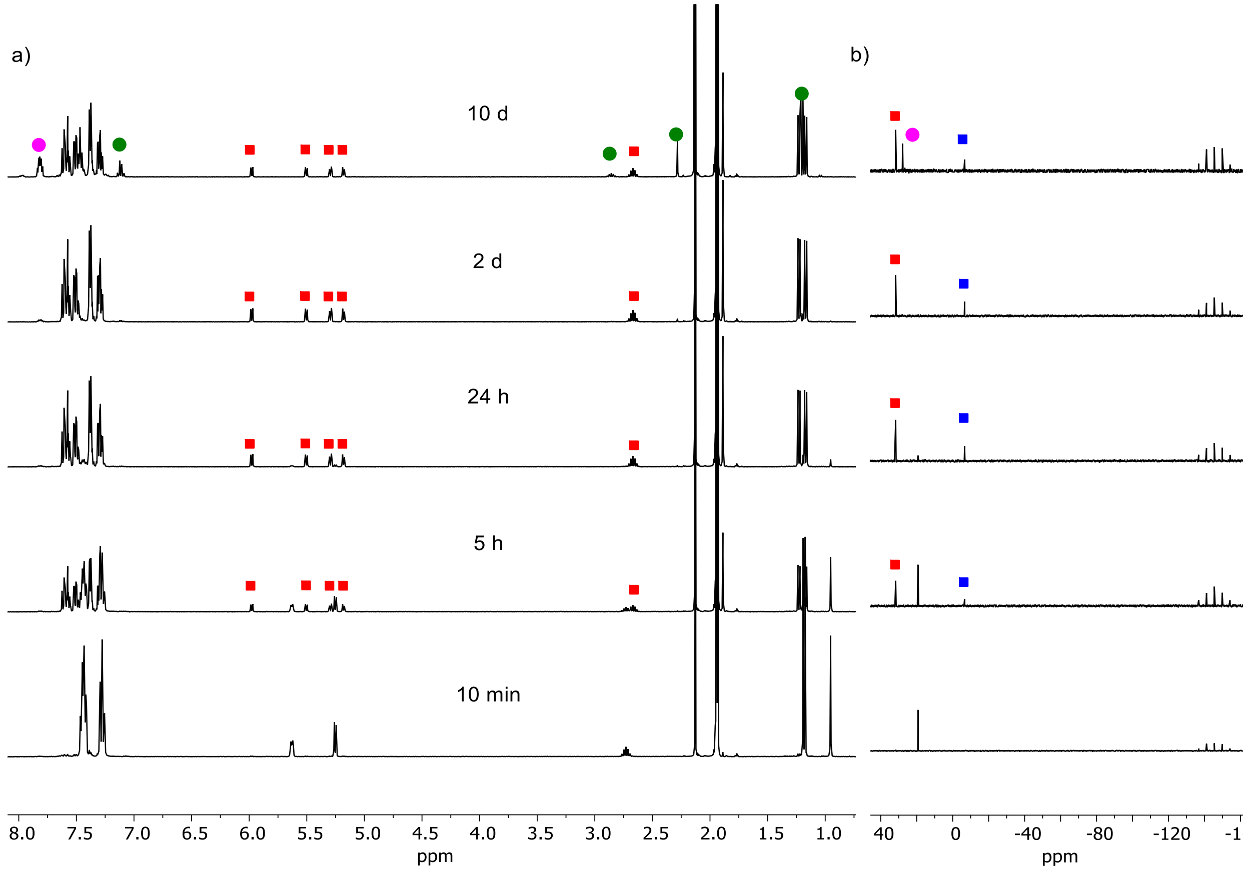

Figure S93. ^{1}H (a) and ^{31}P{^{1}H} (b) NMR spectra for complex **Ru^{H}-PAr$_3$** in CD_3CN over time. Decomposition to various species (green circles, blue and red squares) is observed. Red squares correspond to solvento complex **Ru^{H}-NCCH$_3$**; the ratio between **Ru^{H}-NCCH$_3$** and **Ru^{H}-PAr$_3$** is 1.3:1 at 5 h, 5:1 at 1 d, and >95:1 at 2 d. Blue squares correspond to the signals for free PPh_3. Pink circles correspond to the signals for *trans*-*mer*-**C^{H}-NCCH$_3$** (see Figures S73 and S75). Green circles correspond to free *p*-cymene.

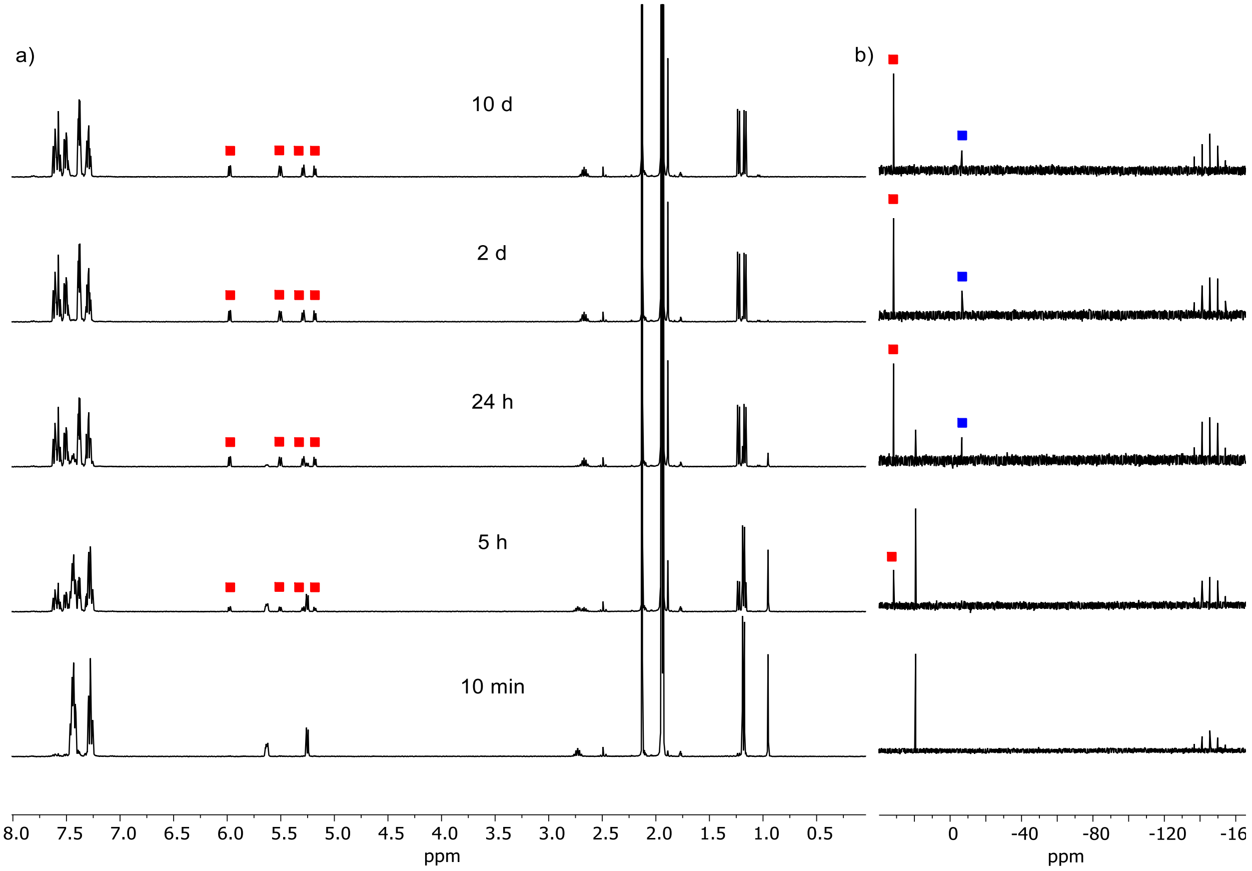

Figure S94. ^{1}H (a) and ^{31}P{^{1}H} (b) NMR spectra for complex **Ru^{H}-PAr$_3$** in CD_3CN over time under exclusion of light. Decomposition to various species (green circles, blue and red squares) is observed. Red squares correspond to solvento complex **Ru^{H}-NCCH$_3$**; the ratio between **Ru^{H}-NCCH$_3$** and **Ru^{H}-PAr$_3$** is 0.4:1 at 5 h, 4:1 at 1 d, and >95:1 at 2 d. Blue squares correspond to the signals for free PPh_3. No evidence for formation of *trans*-*mer*-**C^{H}-NCCH$_3$** or free *p*-cymene is observed.

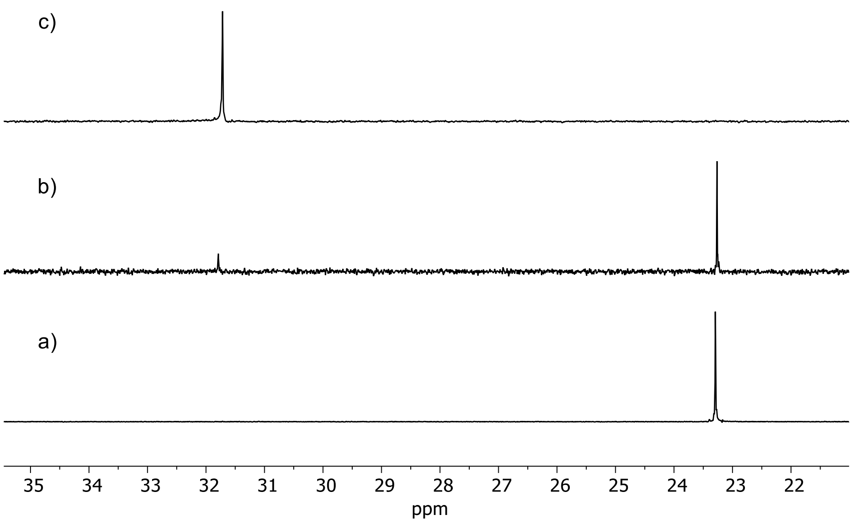

Figure S95. $^{31}P\{^{1}H\}$ NMR spectra (202 MHz) for (a) dichloride complex **Ru^{H}-Cl** (CD_3CN) (b) **Ru^{H}-Cl** after 24 h in a 0.1 *M* CH_3CN solution of [$^{n}Bu_4N$][PF_6] (~30 equiv.) (CH_3CN), and (c) solvento complex **Ru^{H}-NCCH$_3$** (CD_3CN).

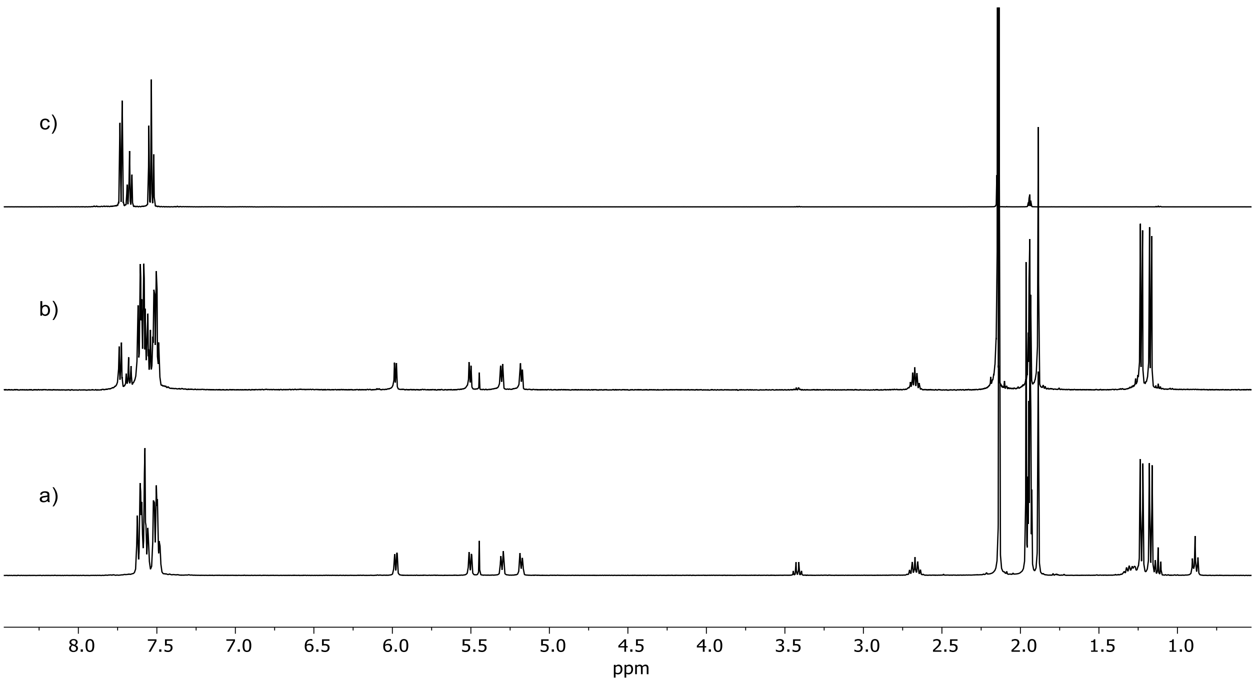

Figure S96. ^{1}H NMR (400 MHz, CD_3CN) of (a) **Ru^{H}-NCCH$_3$**, (b) **Ru^{H}-NCAr^{H},** and (c) PhCN.

Table S1. ^{31}P NMR chemical shifts for complexes Ru^X-L.

X	σ_p	^{31}P NMR (δ, ppm) in $CDCl_3$			
		**Ru^X-Cl**	**Ru^X-$NCCH_3$**	**Ru^X-PAr_3**	**Ru^H-$NCAr^X$**
OMe	–0.27	21.40	35.16	18.07	35.47
Me	–0.17	22.84	-	-	35.59
H	0	24.16	35.78	19.32	35.73
F	0.06	22.81	-	-	-
Cl	0.23	23.66	35.16	20.62	35.88
Br	0.23	24.07	-	-	-
CF_3	0.54	24.64	-	-	35.97

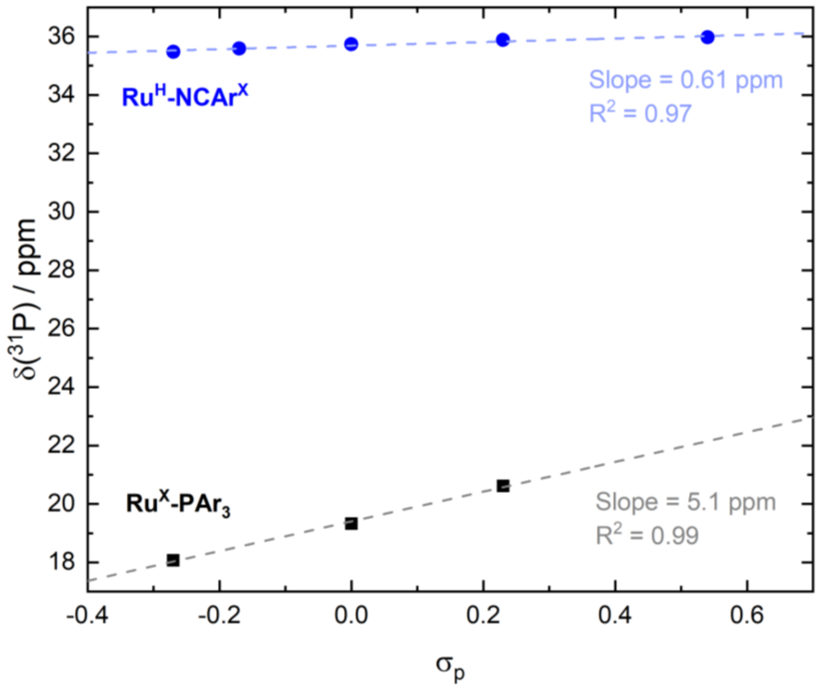

Figure S97. Plot of ^{31}P NMR chemical shifts for complexes **Ru^H-$NCAr^X$** (blue circles) and **Ru^X-PAr_3** (black squares) vs. σ_p.

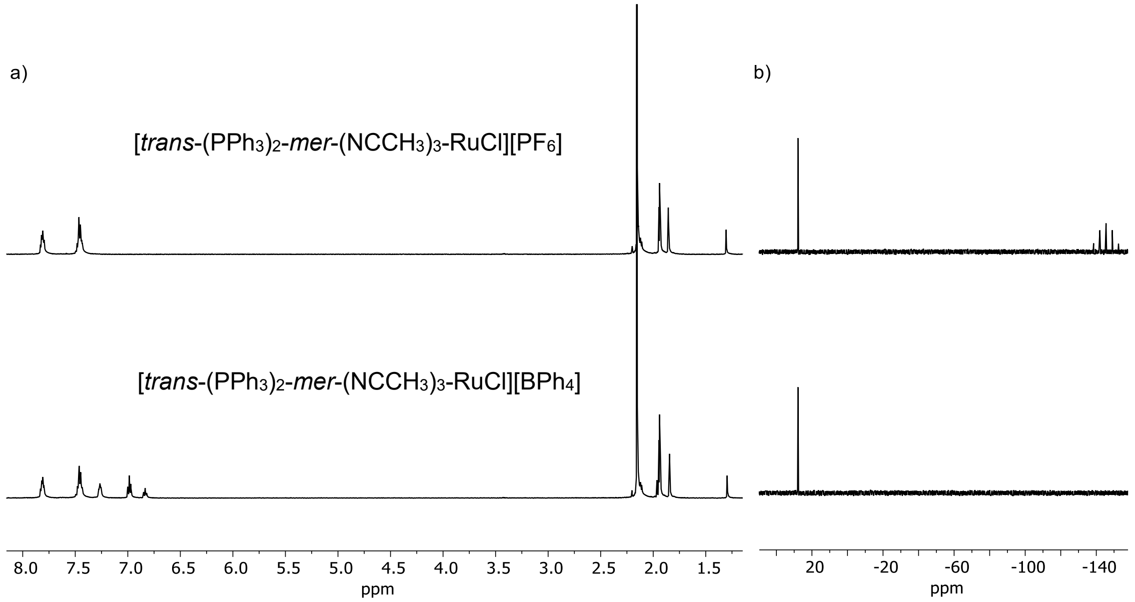

Figure S98. ^1H (a) and $^{31}P\{^1H\}$ (b) NMR spectra for [*trans*-$(PPh_3)_2$-*mer*-$(NCCH_3)_3$-RuCl]X; upper panel, X = PF_6^- (**C^H-$NCCH_3$**); lower panel, X = BPh_4^-.

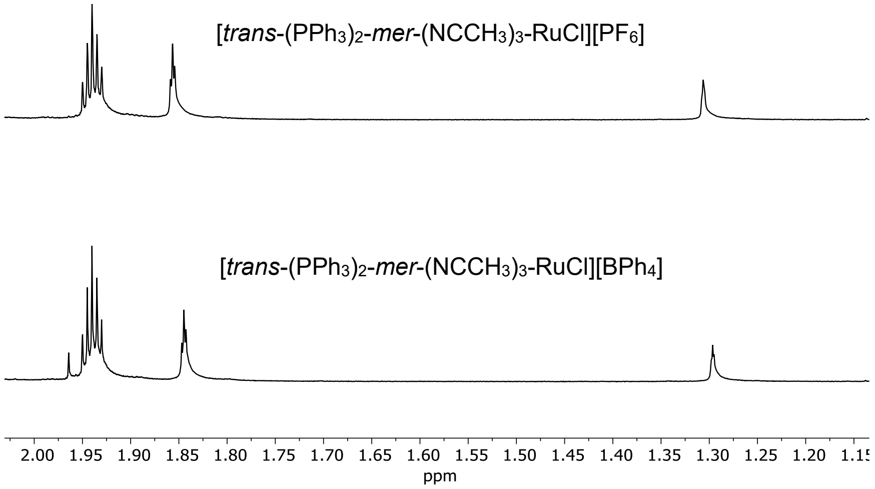

Figure S99. Expanded view of the aliphatic region of ^1H NMR spectra for [*trans*-$(PPh_3)_2$-*mer*-$(NCCH_3)_3$-RuCl]X complexes; the triplets at 1.86 and 1.31 ($^5J_{H,P}$ = 1.1, 0.8 Hz, respectively) correspond to two inequivalent Ru-bound CH_3CN ligands, each coupled to two equivalent ^{31}P nuclei. Upper panel, X = PF_6^-; lower panel, X = BPh_4^-

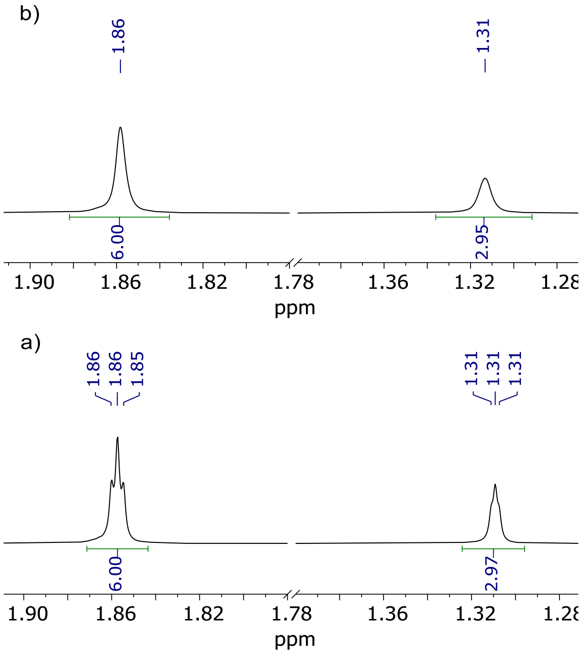

Figure 100. Partial ^{1}H NMR spectra (400 MHz, CD_3CN) for the *trans-mer*-**C^H^-NCCH₃** complex (only parts of the aliphatic region shown); (a) no decoupling; (b) ^{31}P-decoupled data.

*Characterization of cis-mer-**C**$^{\mathbf{H}}$**-NCCH**$_3$ in coordinating vs. non-coordinating solvent.*

In the original report for *cis-mer*-**C**$^{\mathbf{H}}$**-NCCH**$_3$,[4] two signals in the aliphatic region of the ^{1}H NMR spectrum of this compound (δ 2.15 and 2.12 ppm) were assigned to the two sets of inequivalent CH_3CN ligands, with an additional signal at δ 2.02 ppm assigned to a co-crystallized H_2O molecule. Since the signals for the CH_3CN ligands are very close to the location of the signal for H_2O in CD_3CN (2.13 ppm),[5] we aimed to confirm the nature of these resonances. In our hands, ^{1}H NMR data for this material collected in dry CD_3CN revealed no signal near 2.15 ppm (Figure S97), indicating that the originally reported signals were not due to Ru-bound CH_3CN ligands. Instead, the only signal from the Ru compound in this region is a broad singlet at 2.03 ppm (which was also observed in the original report); the integration of this signal, however, displays an integration consistent with two CH_3CN ligands, and must therefore correspond to the two *trans* $NCCH_3$ moieties. Spectra of this complex also routinely display a signal for free CH_3CN with integrations close to one CH_3CN molecule even after prolonged washing with Et_2O and drying *in vacuo*. Taken together, these observations suggest that one of the three CH_3CN ligands in the original complex undergoes fast exchange with CD_3CN solvent.

Intriguingly, NMR data collected in acetone-d_6 indicated a different symmetry for this compound, as only a singlet (δ 47.6 ppm) was observed in the ^{31}P data rather than the broad multiplets observed in CD_3CN (Figure S101). These observations are consistent with formation of a *dimeric* product in non-coordinating solvent (e.g. acetone or CH_2Cl_2), rather than the expected monomeric Ru complex (Scheme S1). In the original report of the *cis-mer*-**C**$^{\mathbf{H}}$**-NCCH**$_3$ complex, dimerization processes had been described for complexes supported by bidentate bisphosphine ligands, though analogous analyses for the bis(PPh_3) complex were not discussed.[4] In these cases, the authors postulated that complexes were isolated as monomers in the solid state, and that dimerization occurred upon dissolution in non-coordinating solvent. These observations are consistent with chloride-bridged dimeric Ru complexes containing both phosphine and nitrile ligands that have appeared in the literature,[4,6-11] including some that display differential behavior in coordinating vs. non-coordinating solvent.[4,11]

Isolation of *cis-mer*-**C**$^{\mathbf{H}}$**-NCCH**$_3$ as a monomeric solid from the synthetic procedure (which could then undergoes dimerization only when dissolved in non-coordinating solvent) is corroborated by the elemental analysis for the material.[4] In both the original report and in our hands, combustion analysis of *cis-mer*-**C**$^{\mathbf{H}}$**-NCCH**$_3$ supports formulation as monomeric $[RuCl(PPh_3)_2(NCCH_3)_3][PF_6]$ (with or without adventitious H_2O), rather than dimeric $[RuCl(PPh_3)_2(NCCH_3)_2]_2[PF_6]_2$ (Table S5). While the driving force for loss of CH_3CN and generation of the dimeric structure in non-coordinating solvent is unclear, the observation of possible fast exchange of a single CH_3CN ligand with solvent in CD_3CN suggests increased lability of one of the nitrile groups, which may drive the equilibrium towards the dimeric compound at low concentrations of CH_3CN in non-coordinating solvent. Notably, ^{1}H NMR data for *cis-mer*-**C**$^{\mathbf{H}}$**-NCCH**$_3$ in CD_3CN indicates that the two CH_3CN ligands present in the complex are chemically equivalent; this suggests that the CH_3CN located in a *trans* position with respect to a PPh_3 ligands is the ligand prone to exchange (consistent with the greater *trans* effect of PPh_3 vs. CH_3CN).[12] Furthermore, loss of this CH_3CN ligand opens a coordination site *cis* to the lone Cl^- ligand; binding of the Cl^- ligand from a different Ru unit at this position in a bridging fashion gives a single dimeric product with D_{2h} symmetry in which all four P nuclei are chemically equivalent–consistent with the lone singlet observed in the ^{31}P data in acetone-d_6 (Scheme S1). Conversely, dimerization involving loss of CH_3CN from at least one of the two coordination sites *cis* to the PPh_3 groups would give products with C_2, C_i, or C_1 symmetry (or mixtures of these), in which not all PPh_3 ligands would be chemically equivalent, which would result in multiple ^{31}P signatures (likely with substantial P-P couplings).

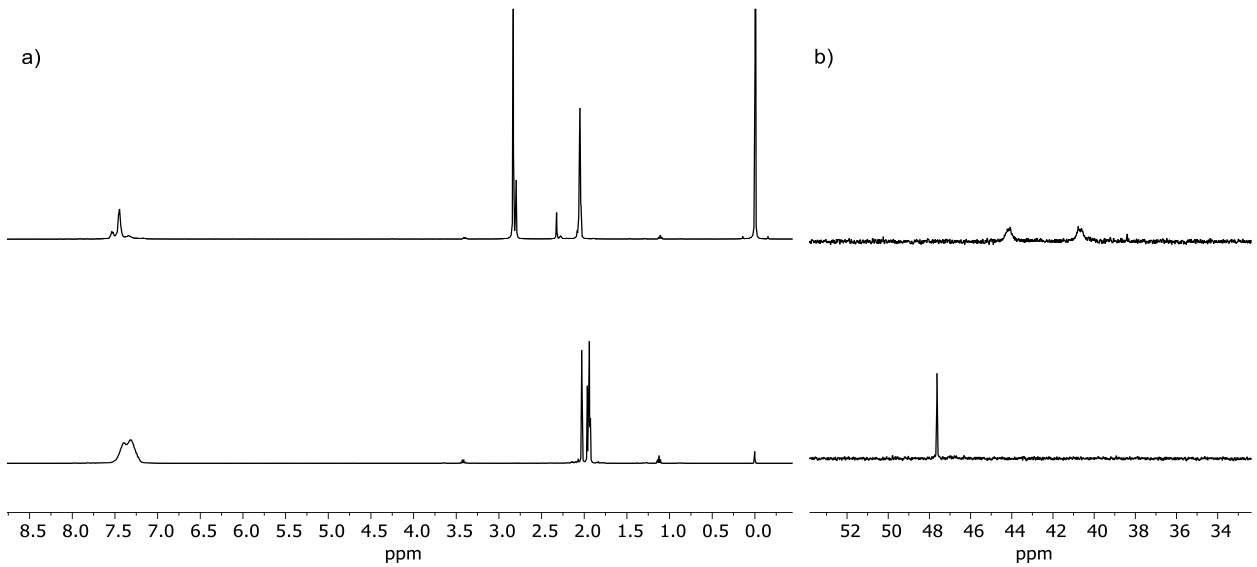

Figure S101. ^{1}H (a) and ^{31}P{^{1}H} (b) NMR spectra for *cis-mer*-**C^H-NCCH$_3$** in CD$_3$CN (upper panel) and acetone-*d*$_6$ (lower panel), suggesting different complex geometry in coordinating vs. non-coordinating solvent.

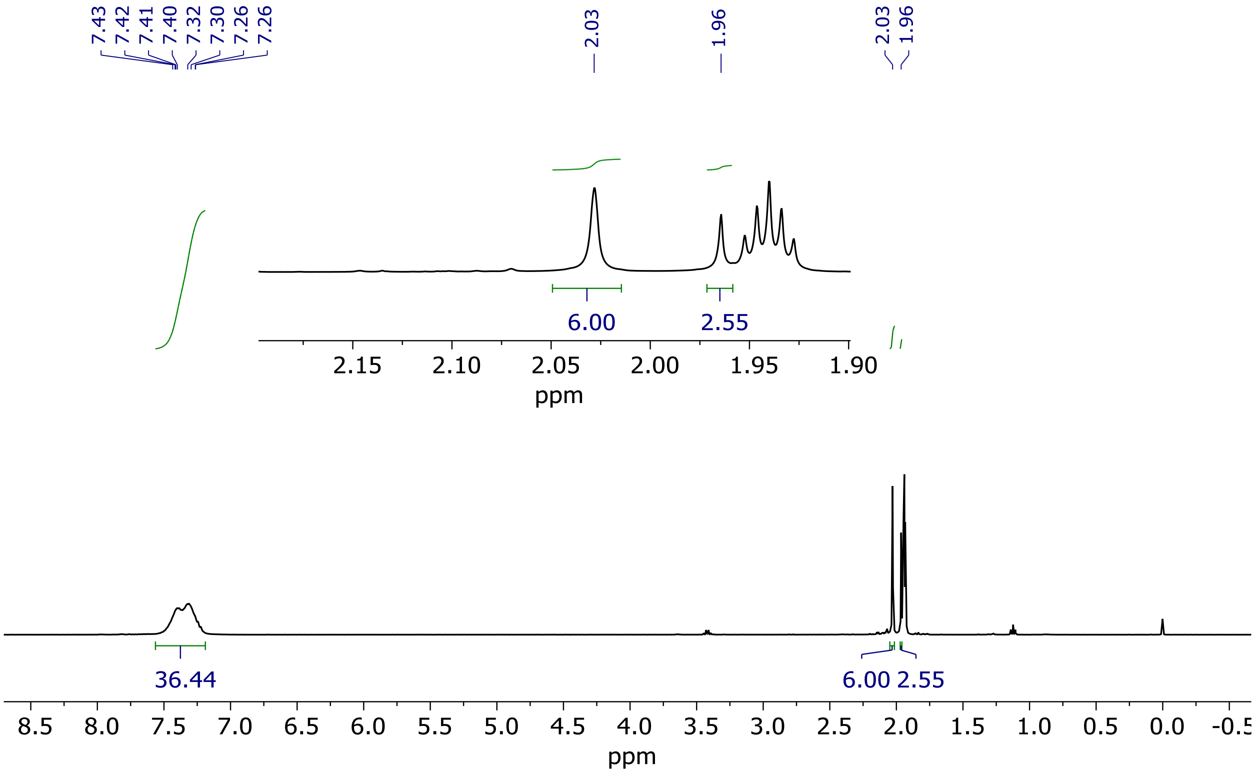

Figure S102. ^{1}H NMR data (400 MHz) for a sample of *cis-mer*-**C^H-NCCH$_3$** in dry CD$_3$CN. Inset: expanded view of the acetonitrile region of the spectrum. The signal at 2.03 ppm corresponds to two Ru-bound acetonitrile ligands based on the integration vs. the aromatic region. The signal at 1.96 ppm corresponds to free acetonitrile, indicating the presence of ~1 equivalent of free acetonitrile.

Scheme S1. Dimerization of *cis-mer*-C^{H}-$NCCH_3$.

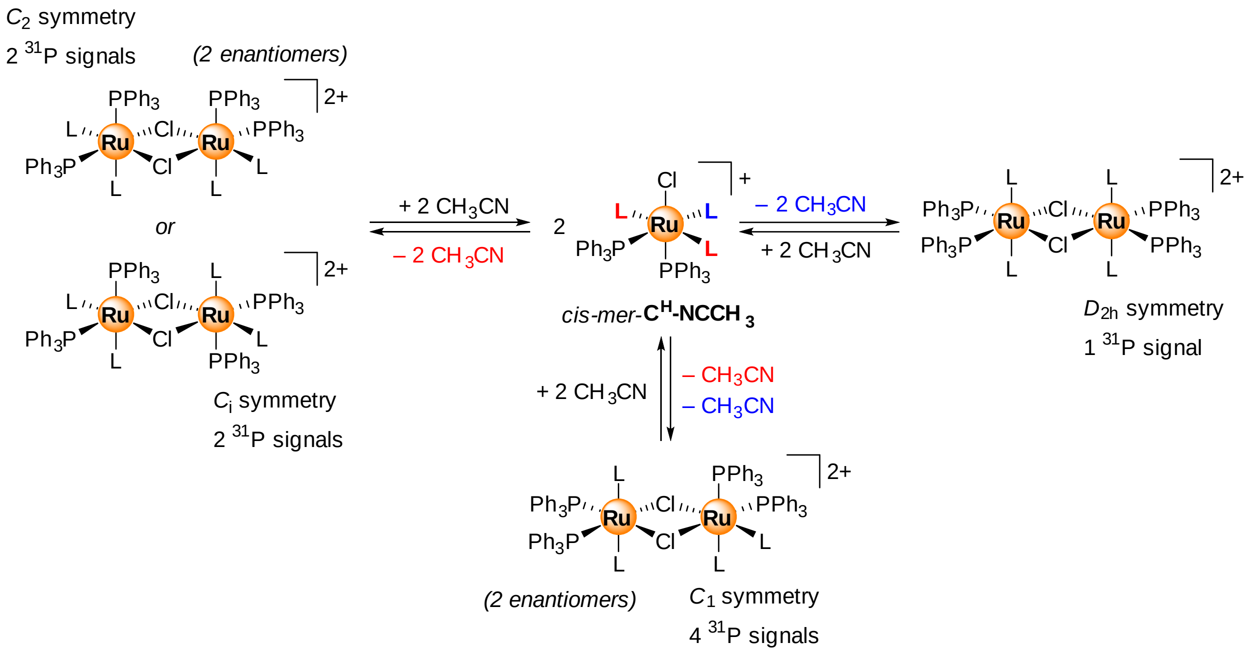

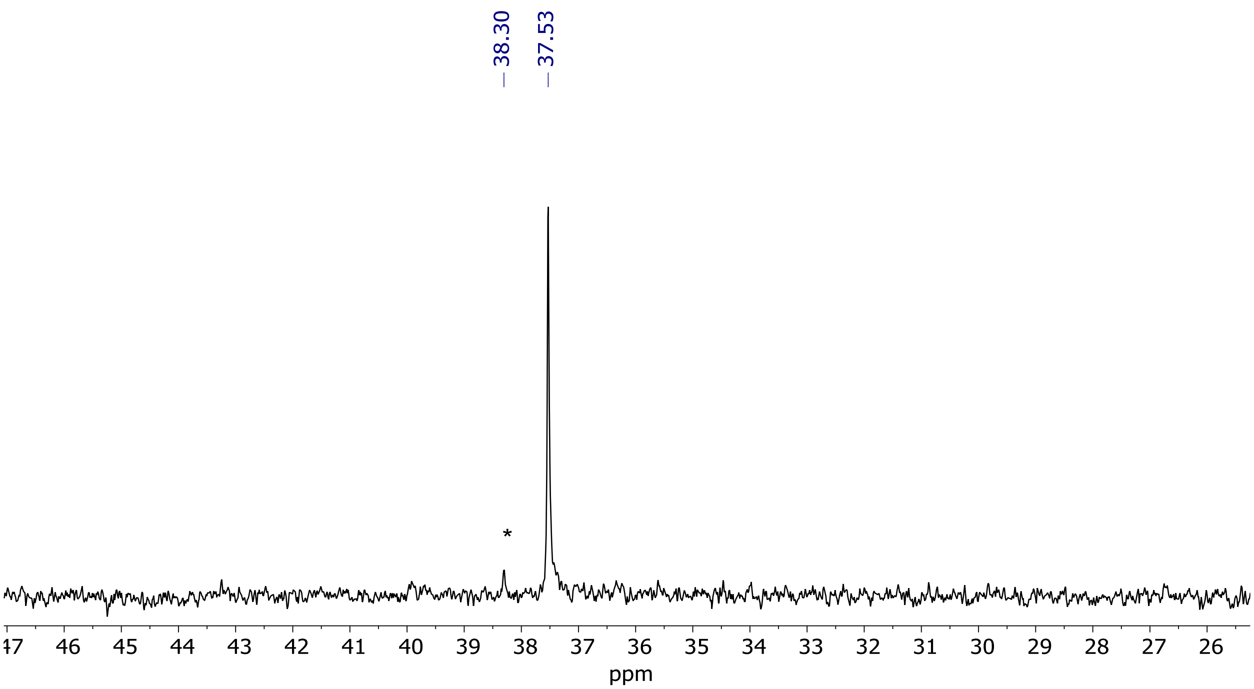

Figure S103. $^{31}P\{^1H\}$ NMR (162 MHz, CD_2Cl_2) for putative *cis-fac*-**C^{H}-$NCCH_3$**; the peak marked with (*) corresponds to an unidentified impurity.

Table S2. Spectroscopic and electrochemical data for isomers of complex $\mathbf{C^{H}}$**-NCCH$_3$**.

Complex	^{31}P NMR^{a} (δ, ppm)	$E°'(Ru^{3+/2+})^{b}$ (V vs. $Fc^{+/0}$)
cis-fac-$\mathbf{C^{H}}$**-NCCH$_3$**	37.5 (s)	+0.87
cis-mer-$\mathbf{C^{H}}$**-NCCH$_3$**	44, 41 (m)	+1.06
trans-mer-$\mathbf{C^{H}}$**-NCCH$_3$**	27.8 (s)	+0.87

^{a} in CD_3CN; ^{b} in 0.1 *M* [$^{n}Bu_4N$][PF_6] in CH_3CN

Proposed mechanism for formation of cis-fac- ***C^{H}-NCCH$_3$*** *from $RuCl_2(PPh_3)_3$.*

A proposed mechanism for the formation of *cis-fac-* ***C^{H}-NCCH$_3$*** as the initial tris(nitrile) isomer generated from $RuCl_2(PPh_3)_3$ is shown in Scheme S2. Reactions of this common precursor with nitriles have been investigated, and dissociation of one of the PPh_3 ligands to give complexes of the form $RuCl_2(PPh_3)(NCCH_3)_2$ is well-established, with the isomeric form(s) of the isolated products being dependent on reaction conditions (solvent, temperature) and the nature of the nitrile.[4,10,13-14] We hypothesize that initial binding of CH_3CN at the open coordination site in $RuCl_2(PPh_3)_3$ (*trans* to the unique PPh_3 ligand)[15] is followed by preferential substitution of one of the two PPh_3 ligands in a *trans* arrangement (due to the greater *trans* effect of PPh_3 vs. CH_3CN)[12] with a second nitrile ligand, giving a *trans*-Cl_2-*cis*-$(NCCH_3)_2$ intermediate. Abstraction of Cl^{-} from this species and binding of a third nitrile equivalent therefore necessarily results in formation of *cis-fac*-$\mathbf{C^{H}}$**-NCCH$_3$**. When chloride abstraction is fast, accumulation of this complex is possible, as in our treatment of a freshly-prepared solution of $RuCl_2(PPh_3)_3$ with Ag^{+}; with Cl^{-}-abstracting agents that react more slowly (NH_4^{+}, K^{+}), however, further conversion of this complex to its *cis-mer* and *trans-mer* isomers (or of any intervening intermediates from the reaction of $RuCl_2(PPh_3)_3$ with nitrile) competes with Cl^{-} abstraction, and the *cis-fac* isomer is therefore not observed.

Scheme S2. Proposed mechanism for generation of *cis-fac*-C^{H}-NCCH$_3$ from $RuCl_2(PPh_3)_3$

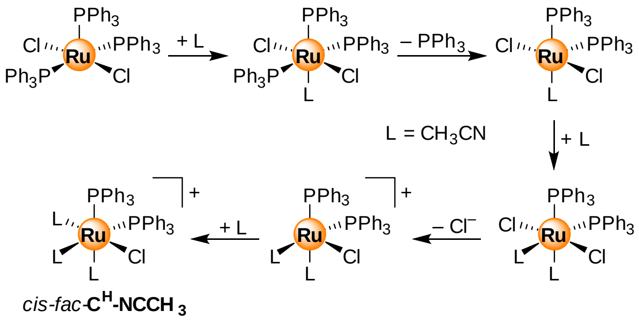

Electrochemistry

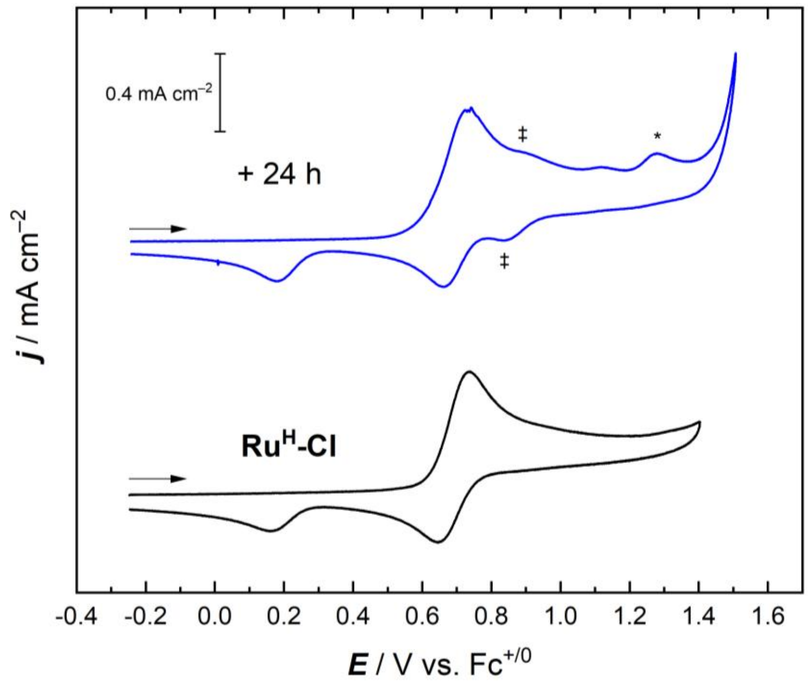

Figure S104. CV data (0.1 *M* [^{n}Bu$_4$N][PF$_6$], 100 mV/s; [Ru] = 3 m*M*) for complex **Ru^{H}-Cl** in CH_3CN over time. Lower trace, black: CV data for **Ru^{H}-Cl** immediately after solution preparation; upper trace, blue: CV data for the same solution after 24 h at room temperature; peaks labeled with (*) correspond to complex **Ru^{H}-NCCH$_3$**; peaks labeled with (‡) correspond to complex **B^{H}-NCCH$_3$**.

Table S3. Reduction potentials[a] for complexes Ru^{X}-L in CH_2Cl_2.

X	σ_p(X)[b]	$E°'$ ([**Ru^{X}-Cl**]$^{+/0}$)	$E°'$ ([**Ru^{X}-NCCH$_3$**]$^{2+/+}$)	E_{pa} ([**Ru^{X}-PAr$_3$**]$^{2+/+}$)
OMe	–0.27	+0.63	+1.28	+1.32
H	0	+0.71	+1.37	+1.40
Cl	0.23	+0.76	+1.42	+1.54

[a] in V vs. Fc$^{+/0}$; [b] from reference [16];

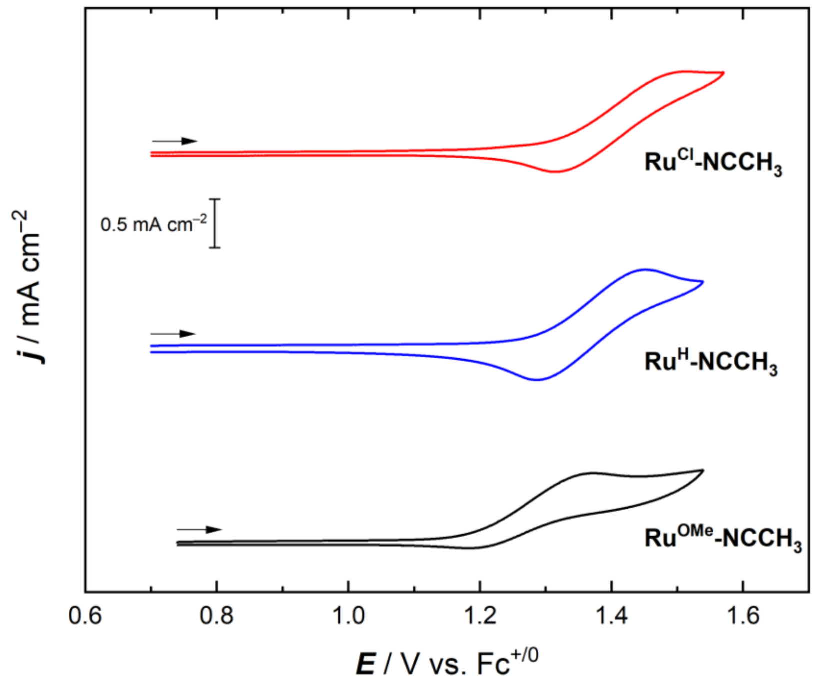

Figure S105. CV data (anodic scanning) for **Ru^{X}-NCCH$_3$** complexes in CH_2Cl_2 (0.1 *M* [^{n}Bu$_4$N][PF$_6$], 100 mV/s; [Ru] = 3 m*M*).

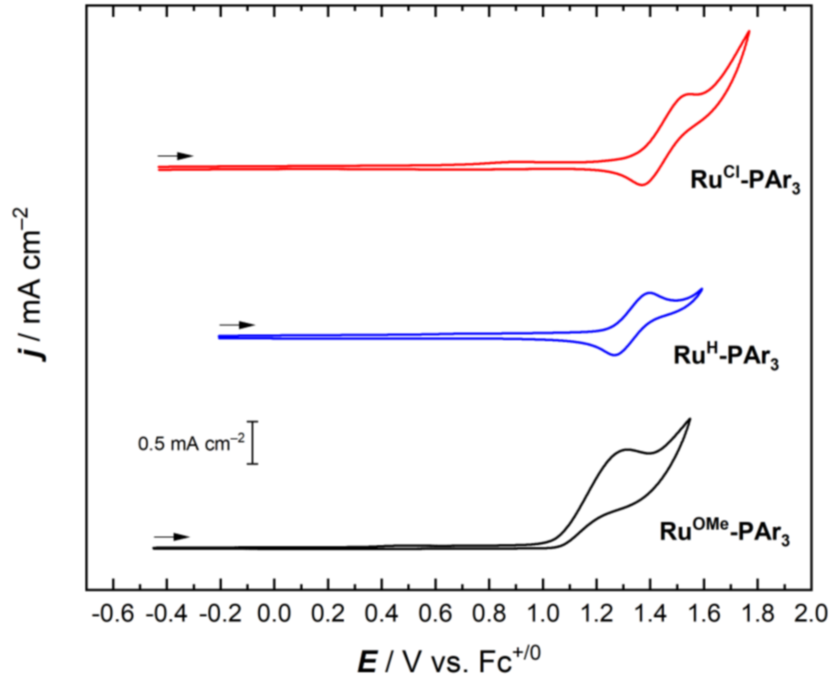

Figure S106. CV data (anodic scanning) for **Ru^{X}-PAr$_3$** complexes in CH_2Cl_2 (0.1 *M* [^{n}Bu$_4$N][PF$_6$], 100 mV/s; [Ru] = 3 m*M*).

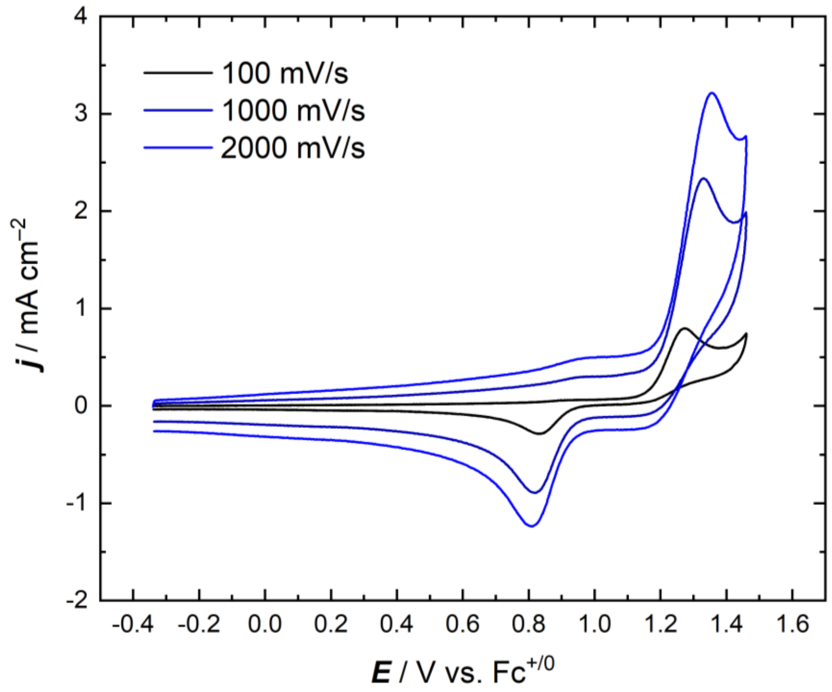

Figure S107. CV data for **Ru^{H}-NCCH$_3$** (0.1 *M* [^{n}Bu$_4$N][PF$_6$] in CH$_3$CN; [Ru] = 3 m*M*) at various scan rates, indicating the persistence of the irreversible nature of the oxidation of **Ru^{H}-NCCH$_3$** and of features corresponding to complex **B^{H}-NCCH$_3$** even at fast scan rates.

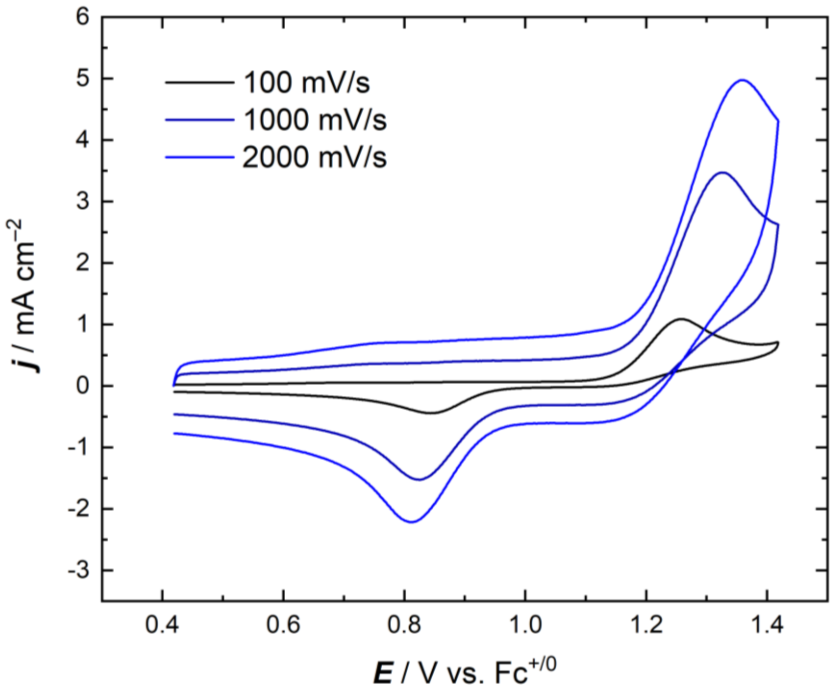

Figure S108. CV data for **Ru^{H}-PAr$_3$** (0.1 *M* [^{n}Bu$_4$N][PF$_6$] in CH$_3$CN; [Ru] = 3 m*M*) at various scan rates, indicating the persistence of the irreversible nature of the oxidation of **Ru^{H}-PAr$_3$** and of features corresponding to complex **C^{H}-NCCH$_3$** even at fast scan rates.

*Reductive electrochemistry of **Ru^X-NCCH$_3$** and **Ru^X-PAr$_3$** complexes.*

Unlike their **Ru^X-Cl** analogues, whose behavior at more reducing potentials (more negative than –1 V vs. Fc$^{+/0}$) is difficult to deconvolute, **Ru^X-NCCH$_3$** and **Ru^X-PAr$_3$** complexes display more regular, though highly irreversible, reduction events in both CH_3CN and CH_2Cl_2 (Figures S103-S106). These reduction events generally display larger cathodic currents than the 1e$^–$ events associated with the Ru(III)/Ru(II) couple, suggesting the possible transfer of multiple electrons. Analogous reductions have been reported in literature systems involving (*p*-cymene)Ru fragments bearing a single Cl$^–$ ligand and supported by either chelating bis(phosphine) platforms or by mixed P-N donor sets.[11,17] In the case of [(*p*-cymene)RuCl(PAr$_3$)(L^N)]$^+$ complexes (L^N = N-donor ligand, such as pyridine or CH_3CN), these reductions were assigned as ECE` processes: initial reduction by one electron (E) is followed by a chemical reaction likely involving dissociation of the Cl$^–$ ligand (C),which results in generation of a complex with a more positive reduction potential than the initial Ru(II) species, causing the immediate transfer of a second electron (E').[11] Similarly, the reduction of [(*p*-cymene)RuCl(Ph$_2$P–N^R–PPh$_2$)]$^+$ complexes (R = alkyl or aryl) was reported to involve the transfer of multiple electrons via ECE` mechanisms, generating formally Ru(0) species that were unstable under electrochemical conditions, leading to observation of attenuated anodic return currents. Notably, similar mechanisms are well-established in reduction of isoelectronic Rh(III) complexes supported by cyclopentadienyl-type ligands and by bidentate N- or P-donor frameworks, though the resulting Rh(I) complexes are substantially more stable and can often be prepared chemically.[18-20] The larger cathodic currents observed for the reduction of complexes **Ru^X-NCCH$_3$** and **Ru^X-PAr$_3$** support analogous mechanisms in these systems. The irreversibility of these reductions can be ascribed due to further reactivity of the electron-rich Ru(0) complex (*p*-cymene)Ru(PAr$^X{}_3$)$_2$, the putative product of the aforementioned 2e$^–$ reduction, which is well known for analogous Ru(0) species.[21]

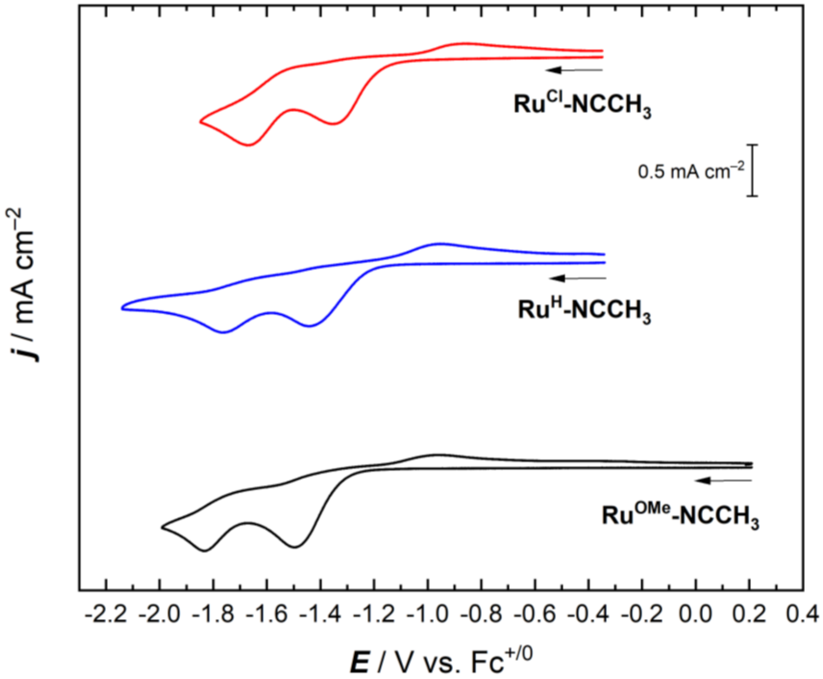

Figure S109. CV data (cathodic scanning) for **Ru^X-NCCH$_3$** complexes in CH_3CN (0.1 *M* [^{n}Bu$_4$N][PF$_6$], 100 mV/s; [Ru] = 3 m*M*).

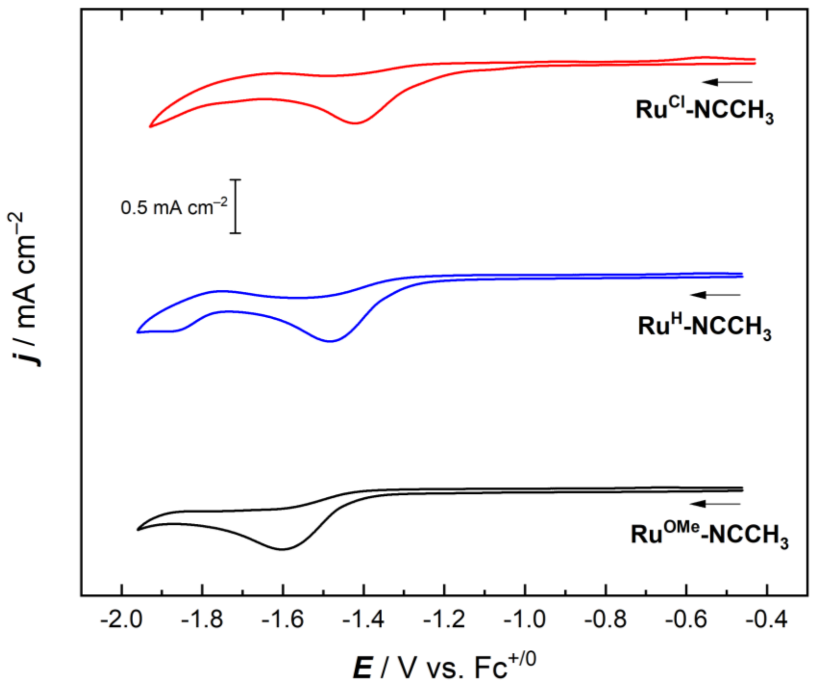

Figure S110. CV data (cathodic scanning) for **Ru^X-NCCH$_3$** complexes in CH_2Cl_2 (0.1 *M* [^{n}Bu$_4$N][PF$_6$], 100 mV/s).

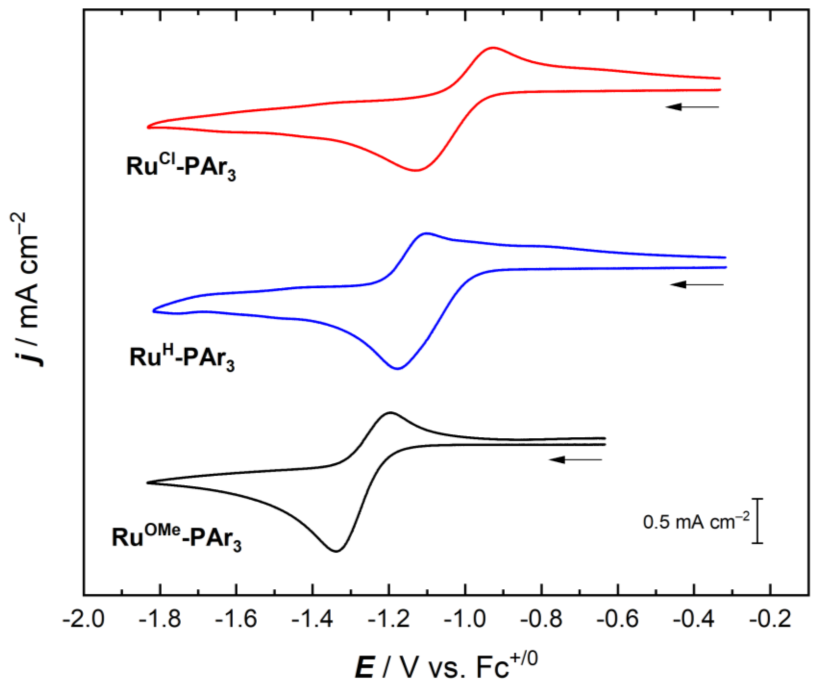

Figure S111. CV data (cathodic scanning) for **Ru^X-PAr$_3$** complexes in CH_3CN (0.1 *M* [^{n}Bu$_4$N][PF$_6$], 100 mV/s).

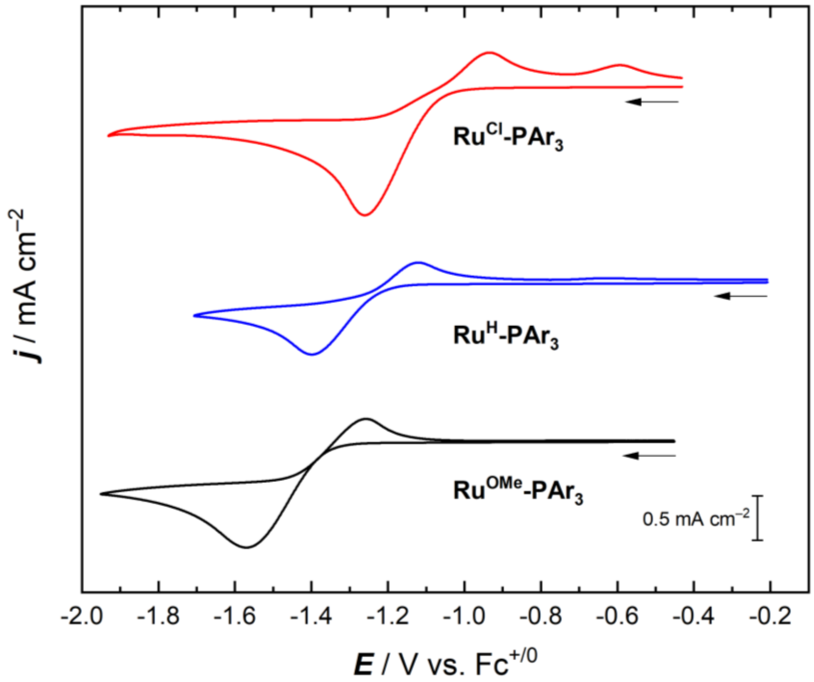

Figure S112. CV data (cathodic scanning) for **Ru^X-PAr$_3$** complexes in CH_2Cl_2 (0.1 *M* [$^nBu_4N$][PF_6], 100 mV/s).

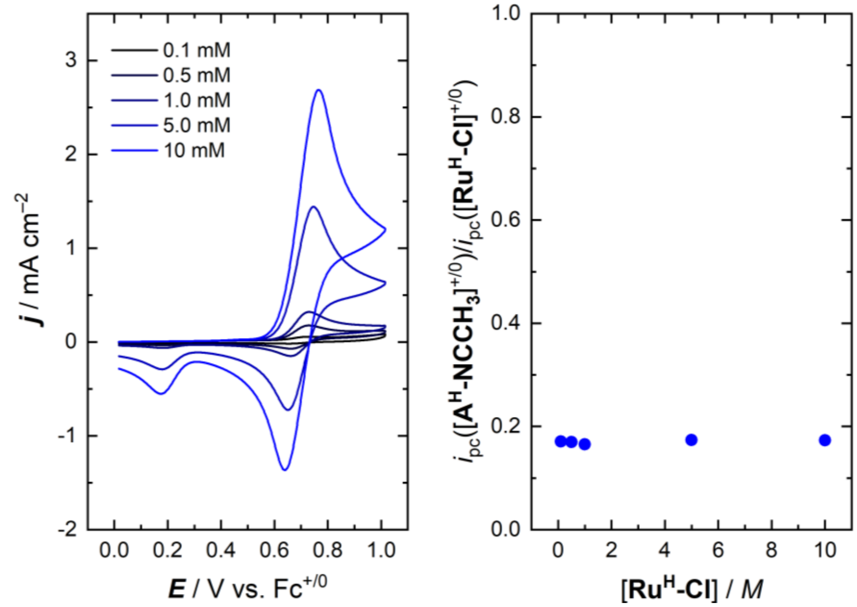

Figure S113. Left panel: CV data (0.1 *M* [$^nBu_4N$][PF_6] in CH_3CN, 100 mV/s) for **Ru^H-Cl** at different [Ru] concentrations (0.1-10 mM). Right panel: plot of the ratio between the cathodic peak current for the **[Ru^H-Cl]$^{+/0}$** couple (i_{pc}(**[Ru^H-Cl]$^{+/0}$**)) and the cathodic peak current for the **[A^H-NCCH$_3$]$^{+/0}$** couple (i_{pc}(**[A^H-NCCH$_3$]$^{+/0}$**)) vs. the concentration of **Ru^H-Cl**.

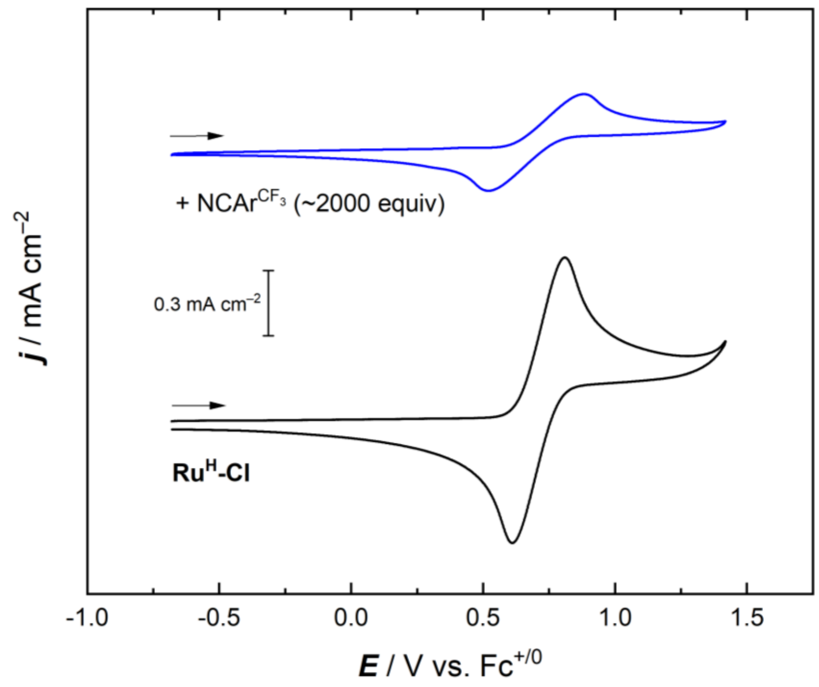

Figure S114. CV data (0.1 *M* [^{n}Bu$_4$N][PF$_6$] in CH$_2$Cl$_2$, 100 mV/s) for complex **Ru^H-Cl** in the presence of ~ 4 *M* (~2,000 equivalents) of NCAr^{CF3}, showing no evidence of formation of the **A^H-NCAr^{CF3}** byproduct.

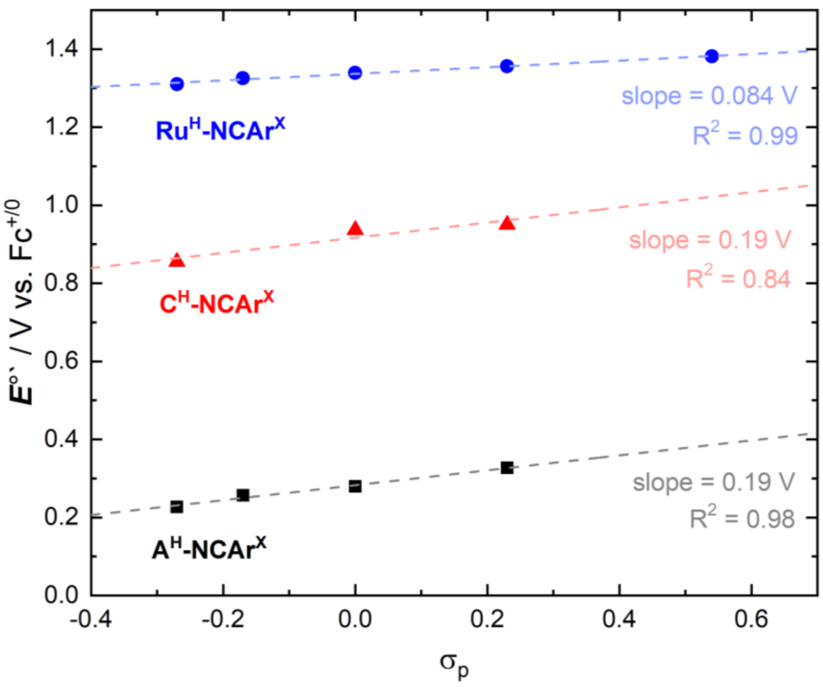

Figure S115. Plot of $E°'$ vs. σ_p for benzonitrile complexes **Ru^H-NCAr^X** (blue circles), **C^X-NCAr^X** (red triangles), and **A^H-NCAr^X** (black squares). The slopes and R^2 values for the lines of best fit are included.

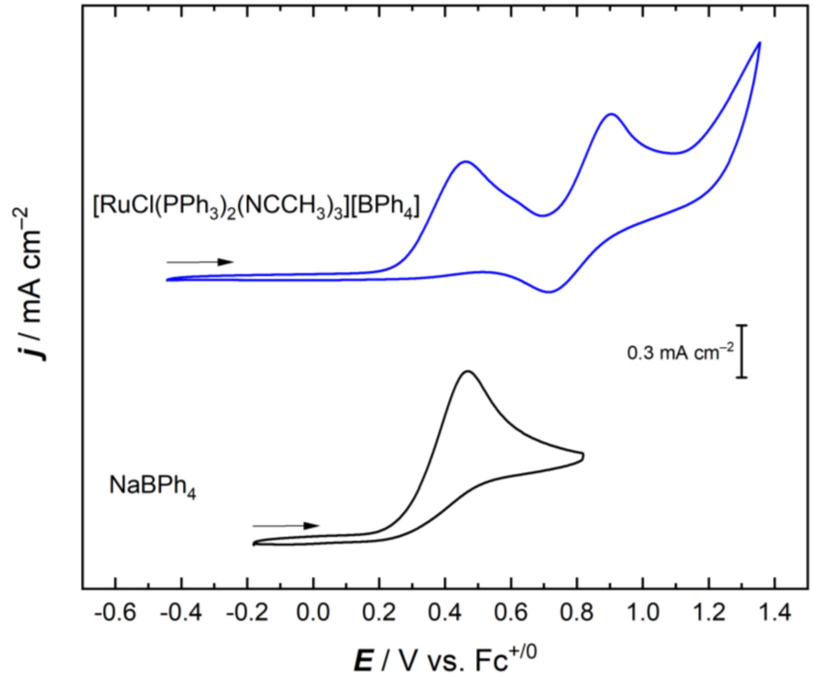

Figure S116. CV data (0.1 *M* [^{n}Bu$_4$N][PF$_6$] in CH$_3$CN, 100 mV/s) for [*trans*-(PPh$_3$)$_2$-*mer*-(NCCH$_3$)$_3$-RuCl][BPh$_4$] (upper trace, blue; [Ru] = 3 m*M*) and NaBPh$_4$ (lower trace, black; [NaBPh$_4$] =3 m*M*).

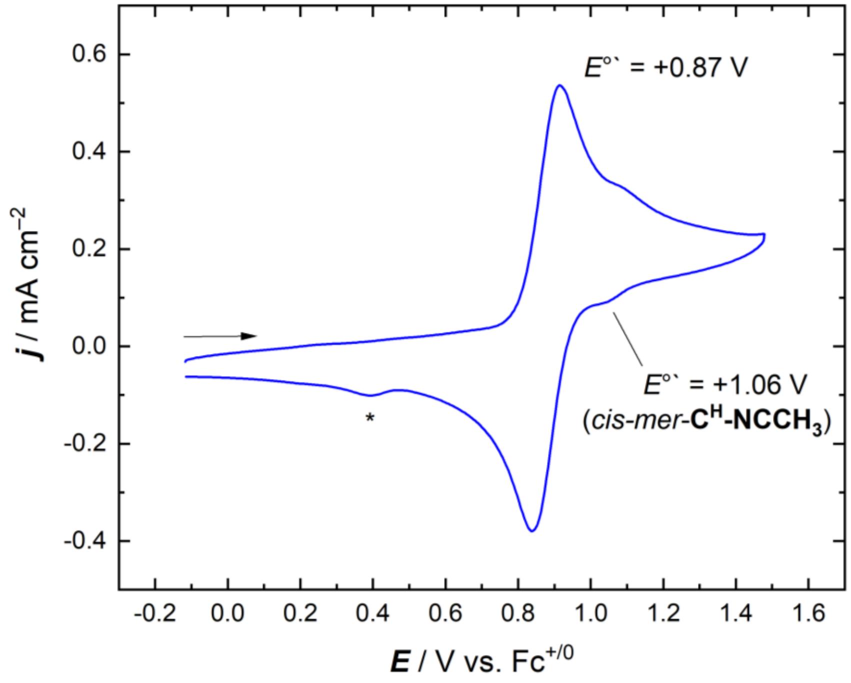

Figure S117. CV data (0.1 *M* [^{n}Bu$_4$N][PF$_6$] in CH$_3$CN, 100 mV/s, [Ru] = 3 m*M*) for *cis*-*fac*-**C^H-NCCH$_3$**; peaks marked with (*) correspond to unidentified Ru impurities.

Table S4. Reduction potentials[a] for complexes **C^H-NCAr^X** generated electrochemically from **Ru^H-PAr₃**.

X	σ_p[b]	$E°'$([**C^H-NCAr^X**]$^{2+/+}$)
OMe	–0.27	0.86
H	0	0.93
Cl	0.23	0.95

[a] in V vs. $Fc^{+/0}$; [b] from reference [16]

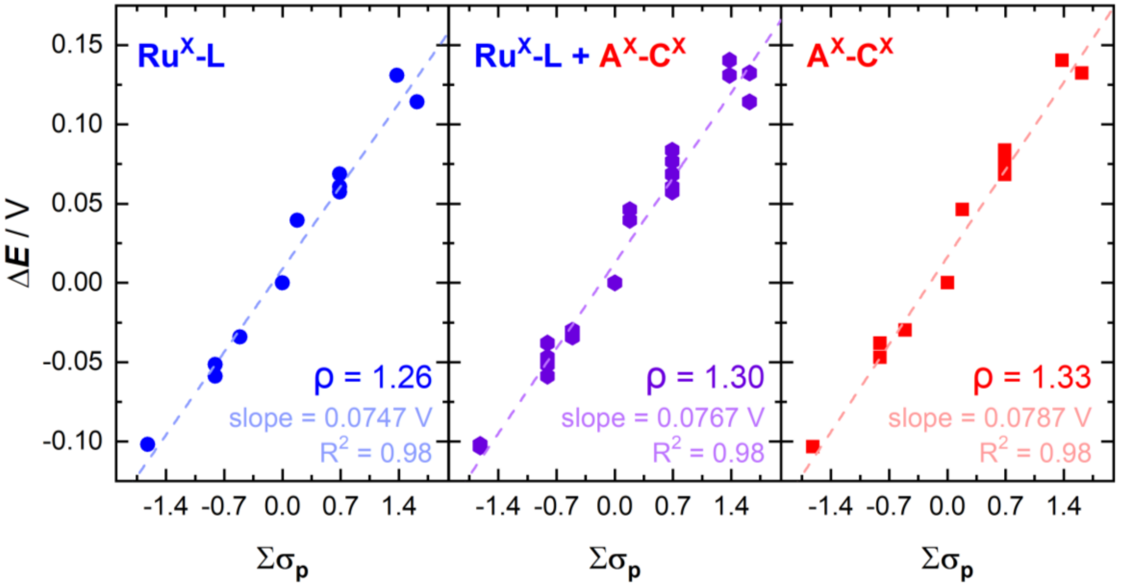

Figure S118. Hammett plots for **Ru^X-L** complexes (left panel, blue circles), **A^X-NCCH$_3$/B^X-NCCH$_3$/C^X-NCCH$_3$** (right panel, red squares), and all mono- and bis(phosphine) complexes in the present study (center panel, purple hexagons). The slopes and R^2 values for the lines of best fit and the calculated values of ρ from each series are included.

*Bulk electrolysis of **Ru^H-Cl***

Controlled-potential electrolysis of **Ru^H-Cl** was attempted according to the procedure by Wright.[22] A solution of **Ru^H-Cl** (0.224 g, 0. 394 mmol) in CH_3CN containing 0.1 *M* $[NH_4][PF_6]$ was electrolyzed at +1.0 V vs. $Fc^{+/0}$ in a two compartment bulk-electrolysis cell separated by porous glass frit using a large surface-areas reticulated carbon working electrode and a platinum gauze counter electrode (BASi). During electrolysis, the color of the solution changed from yellow to red-brown. Subsequent electrolysis at –0.5 V vs. $Fc^{+/0}$ resulted in a change in color of the solution back to yellow. The solution was removed from the cell and the solvent was removed *in vacuo*. The resulting solid was washed with water (3 x 10 mL) to remove the electrolyte and the solid was air-dried.

Comparison of the NMR data in Figure S113 to that reported by Wright and Smith for the putative $RuCl_2(PPh_2(CH_2)_3Ph)(NCCH_3)_3$ complex obtained via bulk electrolysis requires re-referencing of the ^{31}P data in the original report; Wright and Smith reported the chemical shift of bulk electrolysis product as –93.68 ppm;[22] however, no referencing procedure was described in this report. Comparison of the ^{31}P chemical shift for the $PPh_2(CH_2)_3Ph$ free ligand (reported by Wright and Smith as –157.1 ppm) to that described in its original synthesis (–15.9 ppm vs. 85% H_3PO_4),[23] indicates that the ^{31}P chemical shift for the product obtained by Wright and Smith via electrolysis of (cymene)$RuCl_2(PPh_2(CH_2)_3Ph)$ is +47.5 ppm vs. the traditional H_3PO_4 reference, which we have used in our NMR analysis.

As shown in Figure S112, following bulk electrolysis of **Ru^H-Cl**, both **B^H-NCCH$_3$** and **Ru^H-NCCH$_3$** are observed in the first voltammetric cycle, indicating that both complexes are present in the product mixture. ^{31}P analysis of this material (Figure S113) revealed the expected peak at 31.7 ppm for **Ru^H-NCCH$_3$**; a second peak at 49.9 ppm must therefore be ascribed to tetrakis(nitrile) complex **B^H-NCCH$_3$**. The chemical shift for this complex, whose identity is strongly supported by the CV data, is quite similar to that reported by Wright and Smith, suggesting a possible reformulation of this product as the analogous tetrakis(nitrile) complex $[RuCl\,(PPh_2(CH_2)_3Ph)(NCCH_3)_4]^+$. This is also consistent with the single ^1H NMR resonance corresponding to Ru-bound CH_3CN observed in this material, as any isomer of $RuCl_2(PPh_2(CH_2)_3Ph)(NCCH_3)_3$ is expected to display two distinct CH_3CN environments, while all CH_3CN ligands would be equivalent in the *trans* isomer of $[RuCl\,(PPh_2(CH_2)_3Ph)(NCCH_3)_4]^+$.

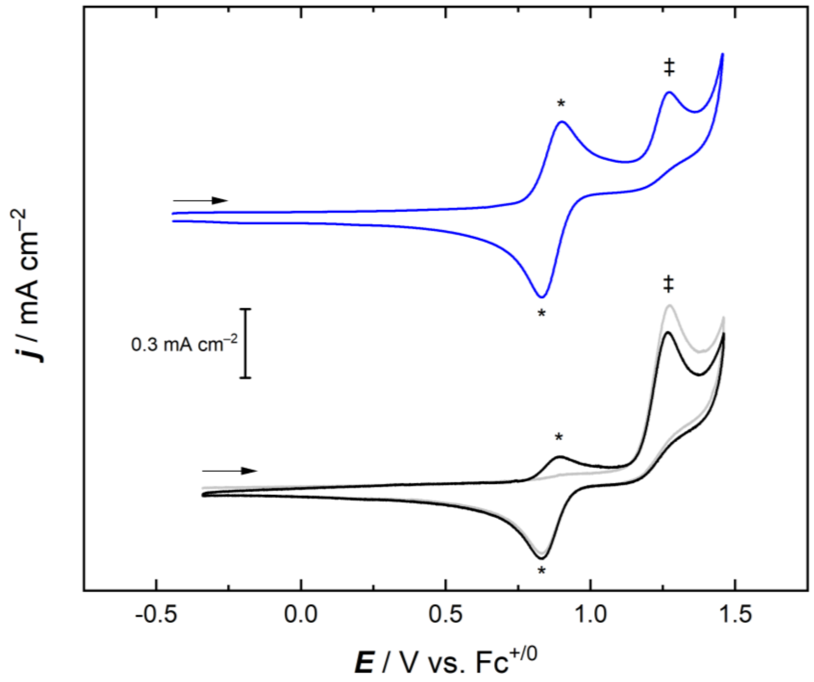

Figure S119. Upper trace (blue): CV data (0.1 *M* [^{n}Bu$_4$N][PF$_6$] in CH_3CN) for the material obtained from controlled-potential electrolysis of **Ru^{H}-Cl**. Lower trace (gray, black): CV data (0.1 *M* [^{n}Bu$_4$N][PF$_6$] in CH_3CN) for **Ru^{H}-NCCH$_3$**; the first voltammetric cycle is shown in gray. Peaks marked with (‡) correspond to **Ru^{H}-NCCH$_3$**. Peaks marked with (*) correspond to **B^{H}-NCCH$_3$**.

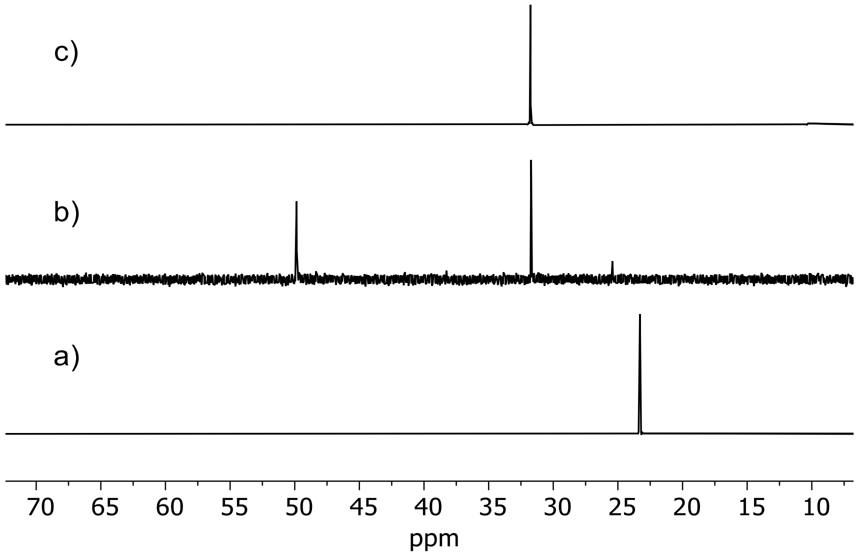

Figure S120. (a) ^{31}P NMR data for **Ru^{H}-Cl**; (b) ^{31}P NMR data for the material obtained from controlled-potential electrolysis of **Ru^{H}-Cl**; the most downfield signal is at 49.9 ppm (cf. 47.5 ppm reported for putative $RuCl_2(PPh_2(CH_2)_3Ph)(NCCH_3)_3$);[22] (c) ^{31}P NMR data for **Ru^{H}-NCCH$_3$**.

Elemental analysis of *cis-mer*-C^H-NCCH$_3$.

Table S5. Elemental analysis results for *cis-mer*- **C^H-NCCH$_3$** samples.

	C (%)	H (%)	N (%)	***Reference***
Calculated composition				
$[RuCl(PPh_3)_2(NCCH_3)_3][PF_6]$	54.29	4.23	4.52	
$[RuCl(PPh_3)_2(NCCH_3)_3][PF_6]\cdot H_2O$	53.26	4.36	4.44	
$[RuCl(PPh_3)_2(NCCH_3)_2]_2[PF_6]_2$	54.09	4.09	3.15	
Found composition				
cis-mer-**C^H-NCCH$_3$**	53.43	4.13	4.28	[4]
cis-mer-**C^H-NCCH$_3$** (sample A)	54.46	4.25	4.40	*this work*
cis-mer-**C^H-NCCH$_3$** (sample B)	53.37	4.33	4.19	*this work*

X-Ray Crystallography

Table S6. Crystal and refinement data

	**Ru^{OMe}-PAr_3**	**Ru^{H}-$NCAr^{OMe}$**	**Ru^{H}-$NCAr^{Me}$**	**Ru^{H}-$NCAr^{H}$**
CCDC number	2532847	2532845	2532848	2532844
empirical formula	$C_{52}H_{55.75}ClF_6O_6P_3Ru$	$C_{36}H_{36}ClF_6NOP_2Ru$	$C_{36}H_{36}ClF_6NP_2Ru$	$C_{35}H_{34}ClF_6NP_2Ru$
formula wt	1120.14	811.12	795.12	781.09
T (K)	100(2)	100(2)	100(2)	100(2)
a, Å	20.421(2)	17.733(6)	10.0976(7)	9.998(2)
b, Å	11.1731(14)	10.602(4)	11.0768(7)	10.805(3)
c, Å	22.370(3)	18.998(6)	16.5250(10)	17.132(4)
α, deg	90	90	89.367(2)	88.289(9)
β, deg	108.336(3)	90	87.168(2)	88.470(7)
γ, deg	90	90	63.710(2)	63.358(7)
V, Å^3	4844.9(10)	3572(2)	1654.98(19)	1653.3(7)
Z	4	4	2	2
cryst syst	monoclinic	orthorhombic	triclinic	triclinic
space group	$P2_1/n$	$Pca2_1$	P-1	P-1
ρ_{calcd}, g/cm^3	1.536	1.508	1.596	1.569
2θ range, deg	4.12 to 52.124	3.842 to 52.202	4.102 to 52.046	4.218 to 52.126
μ, mm^{-1}	0.552	0.665	0.713	0.713
abs corr	numerical	numerical	numerical	numerical
GOOF^c	1.102	1.029	1.054	1.088
R1,^{a} wR2^b (I > 2σ(I))	0.0494, 0.1021	0.0424, 0.0925	0.0315, 0.0627	0.0281, 0.0671

^{a} $R1 = \Sigma||F_o|-|F_c||/\Sigma|F_o|$ ^{b} $wR2 = [\Sigma[w(F_o^2-F_c^2)^2]/\Sigma [w(F_o^2)^2]]^{1/2}$ ^{c} $GOOF = S = [\Sigma[w(F_o^2-F_c^2)^2]/(n-p)]^{1/2}$

Table S7. Crystal and refinement data (cont'd).

	**Ru^H-NCAr^Cl**	**Ru^H-NCAr^CF3**	*trans-mer-***C^H-NCCH₃**
CCDC number	2532846	2532850	2532849
empirical formula	$C_{35}H_{33}Cl_2F_6NP_2Ru$	$C_{216}H_{198}Cl_6F_{54}N_6P_{12}Ru_6$	$C_{60.5}H_{52.5}BCl_{2.5}F_4N_3O_{3.5}P_2Ru$
formula wt	815.53	5094.55	1216.00
T (K)	100(2)	100(2)	100(2)
a, Å	10.202(3)	11.1010(6)	12.1350(8)
b, Å	11.114(3)	20.4390(12)	16.3962(10)
c, Å	16.605(5)	24.9043(14)	16.4673(10)
α, deg	90.012(10)	73.678(2)	95.843(2)
β, deg	92.521(12)	87.214(2)	106.509(2)
γ, deg	116.799(9)	77.860(2)	107.462(2)
V, $Å^3$	1678.6(8)	5301.3(5)	2933.3(3)
Z	2	1	2
cryst syst	triclinic	triclinic	triclinic
space group	P-1	P-1	P-1
ρ_{calcd}, g/cm^3	1.613	1.596	1.377
2θ range, deg	4.106 to 52.516	3.914 to 52.012	4.176 to 52.016
μ, mm^{-1}	0.783	0.685	0.496
abs corr	numerical	numerical	numerical
GOOF[c]	1.057	1.094	1.049
R1,[a] wR2[b]	0.0494,	0.0472,	0.0469,
(I > 2σ(I))	0.1187	0.1032	0.1222

[a] $R1 = \Sigma||F_o|-|F_c||/\Sigma|F_o|$ [b] $wR2 = [\Sigma[w(F_o^2-F_c^2)^2]/\Sigma [w(F_o^2)^2]]^{1/2}$ [c] $GOOF = S = [\Sigma[w(F_o^2-F_c^2)^2]/(n-p)]^{1/2}$

Evaluation of possible π-π interactions in solid-state structures of bis(phosphine) complexes.

In the solid-state (XRD) structure of complex **Ru^{OMe}-PAr$_3$**, possible π-π interactions are observed between aryl rings on the two phosphine ligands. Similar relative orientations of aryl groups are observed in the solid-state structures of other bis(phosphine) complexes,[24-25] though examples exist of structures of similar complexes that lack this parallel arrangement of aromatic groups.[26-27] To gain further insight into these possible π-π interactions, the structures of **Ru^{OMe}-PAr$_3$** and relevant literature compounds were evaluated using the Aromatics Analyzer tool in the Mercury crystallographic software.[28] In complexes with aryl groups in near-parallel orientations (interplane angle < 15°), interactions with scores between 5.3 and 7 were detected, consistent with interactions generally moderate in strength. In several structures, however, moderate to strong interactions (scores = 5.3-8.4) between aryl groups with more obtuse interplane angles (40-60°) were observed, including in some that lacked any parallel aryl groups. In the majority of structures, furthermore, strong π-π interactions were identified that were intermolecular in nature, indicating that packing effects may also play a role in the orientation of these groups. Overall, therefore, while π-π interactions are observed in many bis(phosphine) complexes in the solid state, these effects are significantly convoluted (including between inter- and intramolecular interactions) and are likely not critical to stabilization of these compounds.

Table S8. Selected bond and angle metrics for tris(nitrile) complexes **C^{H}-L**.

	trans-mer-**C^{H}-NCAr^{OMe}**	*trans-mer*-**C^{H}-NCCH$_3$**[a,b]
CCDC #	2532849	266619
	bond lengths (Å)	
Ru–Cl	2.4007(8)	2.4141(9)
Ru–P	2.3888(9), 2.3800(8)	2.4076(9), 2.4145(9)
Ru–N	2.010(3), 1.994[c], 2.012[c]	2.034(2), 2.021(2), 2.014(2)
C–N	1.144(4), 1.150[c], 1.143[c]	1.128(4), 1.128(4), 1.134(3)
	bond angles (°)	
Cl–Ru–N	174.8[c]	178.66(6)
P–Ru–P	176.55(3)	177.78(2)
N–Ru–N[d]	174.1[c]	175.29(9)

[a] counteranion: BPh_4^-; [b] from reference [29]; [c] average of measurement from two disordered benzonitrile positions; [d] N–Rh–N angle for *trans* benzonitrile ligands

Refinement details for Ru^{OMe}-PAr$_3$

Three of the six aryl groups on the phosphine ligands in **Ru^{OMe}-PAr$_3$** displayed substantial disorder, which was successfully modeled in each case as two distinct orientations of the aryl group. The atoms in each of the two orientations for the disordered fragments were labeled with A/B. The occupancies of the two orientations of each aryl group were 64:36 (C25-C30 aryl group), 74:26 (C39-C44), and 56:44 (C46-C51). The outer-sphere PF_6^- counteranion was likewise disordered and was therefore modeled as two distinct orientations with 51:49 occupancy.

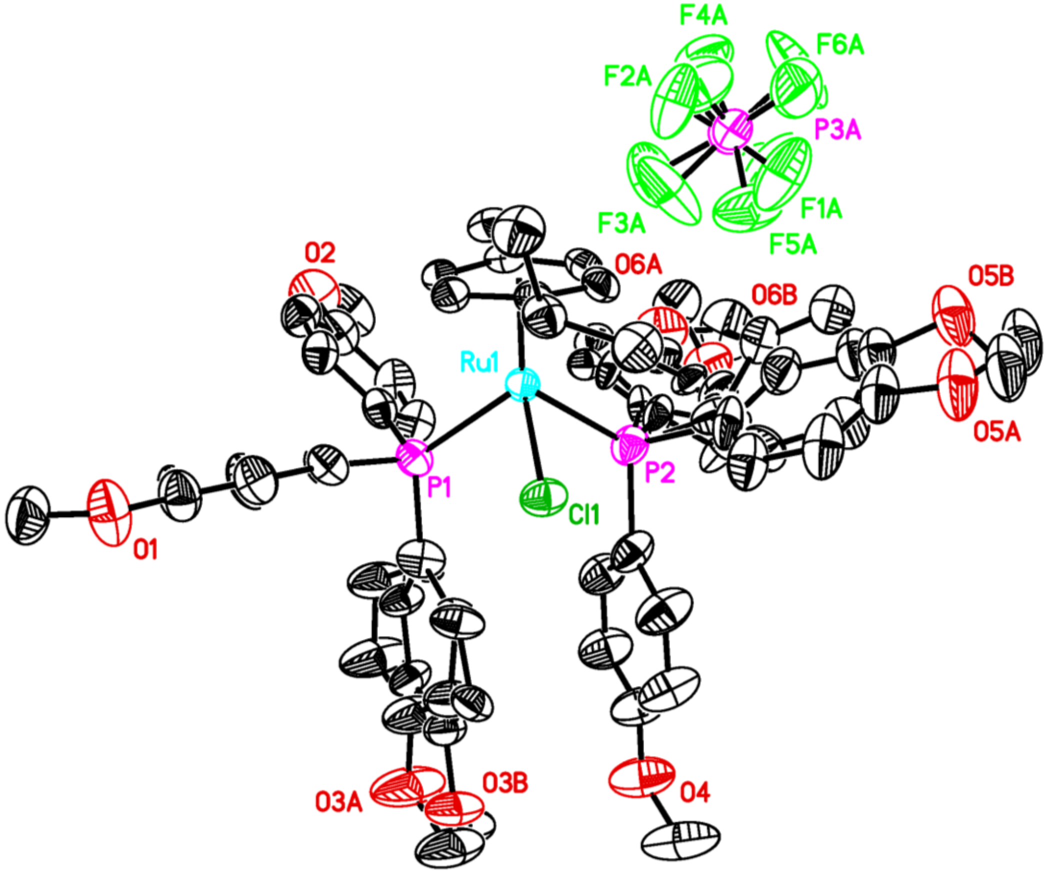

Figure S121. Full solid-state structure of **Ru^{OMe}-PAr$_3$**. Hydrogen atoms omitted for clarity. Both orientations are shown for the three disordered aryl groups and the PF_6^- counteranion (only one set of labels shown for clarity). Displacement ellipsoids shown at the 50% probability level. Data collected at 100 K.

Refinement details for Ru^{H}-NCAr^{OMe}

No special refinement needed.

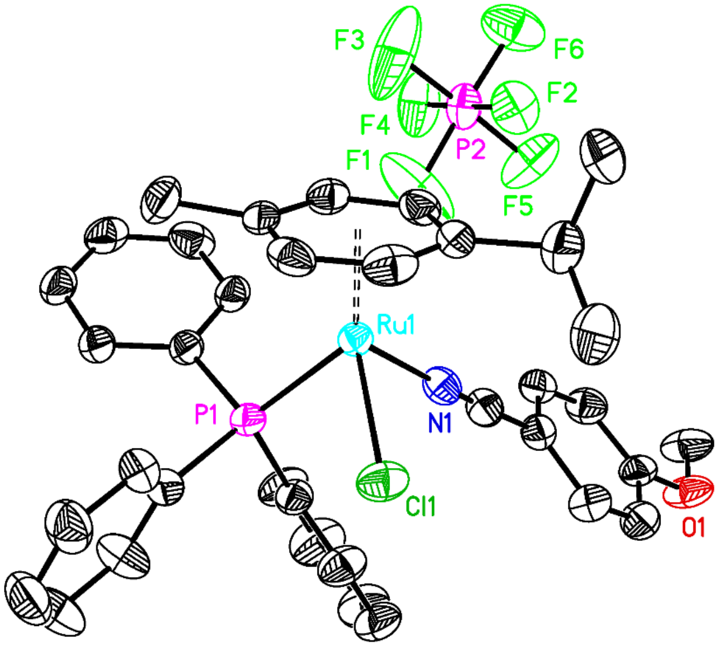

Figure S122. Full solid-state structure of **Ru^{H}-NCAr^{OMe}**. Hydrogen atoms omitted for clarity. Displacement ellipsoids shown at the 50% probability level. Data collected at 100 K.

Refinement details for Ru^{H}-NCAr^{Me}
No special refinement needed.

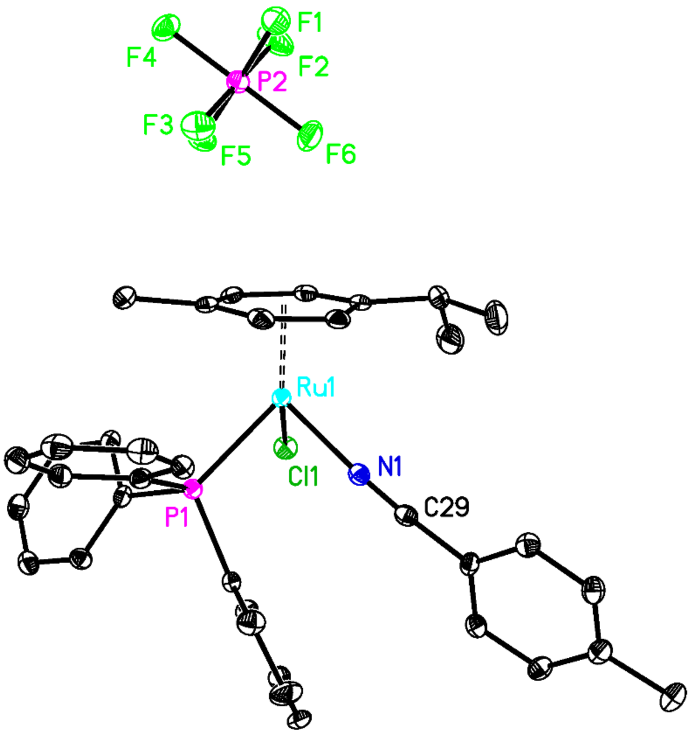

Figure S123. Full solid-state structure of **Ru^{H}-NCAr^{Me}** . Hydrogen atoms omitted for clarity. Displacement ellipsoids shown at the 50% probability level. Data collected at 100 K.

Refinement details for Ru^{H}-NCAr^{H}

No special refinement needed.

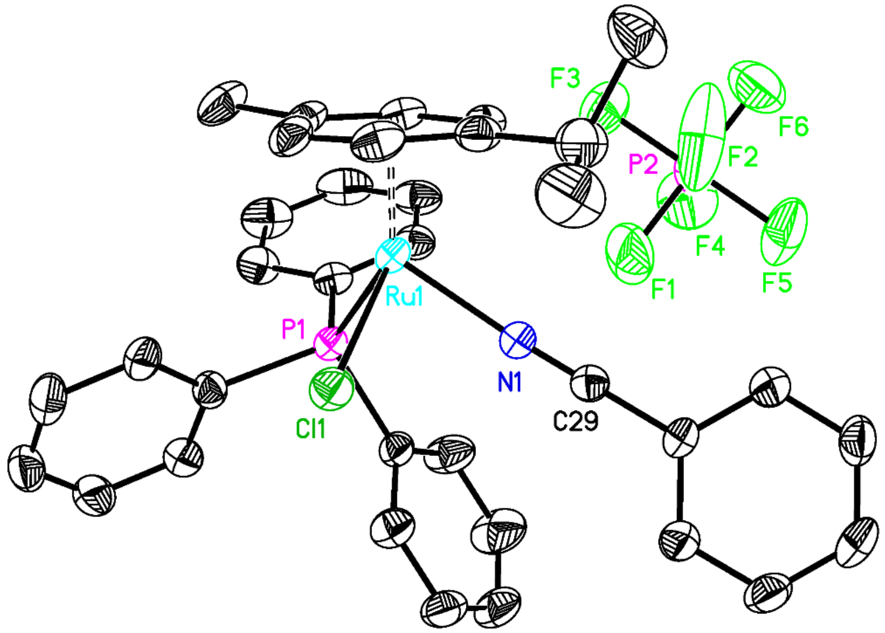

Figure S124. Full solid-state structure of **Ru^{H}-NCAr^{H}**. Hydrogen atoms omitted for clarity. Displacement ellipsoids shown at the 50% probability level. Data collected at 100 K.

Refinement details for Ru^H-NCAr^{Cl}

No special refinement needed.

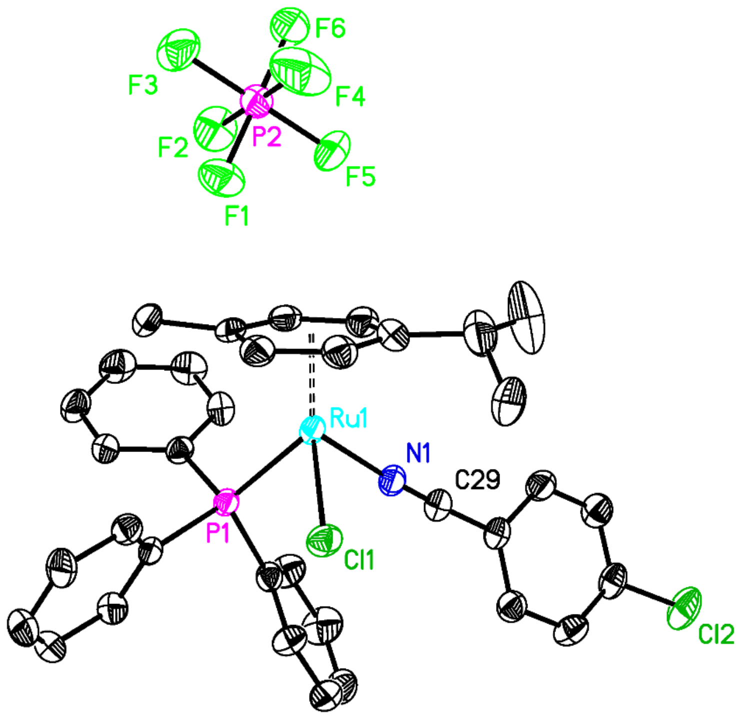

Figure S125. Full solid-state structure of **Ru^H-NCAr^{Cl}**. Hydrogen atoms omitted for clarity. Displacement ellipsoids shown at the 50% probability level. Data collected at 100 K.

Refinement details for Ru^{H}-NCAr^{CF3}

The *i*-Pr group on the cymene ligand bound to Ru2 was disordered over two positions. This disorder was modeled as two orientations of one of the methyl substituents, with 67:33 occupancy. The position and anisotropic displacement parameters for the methyne carbons (C43a and C43b) were constrained to be identical with EADP and EXYZ commands.

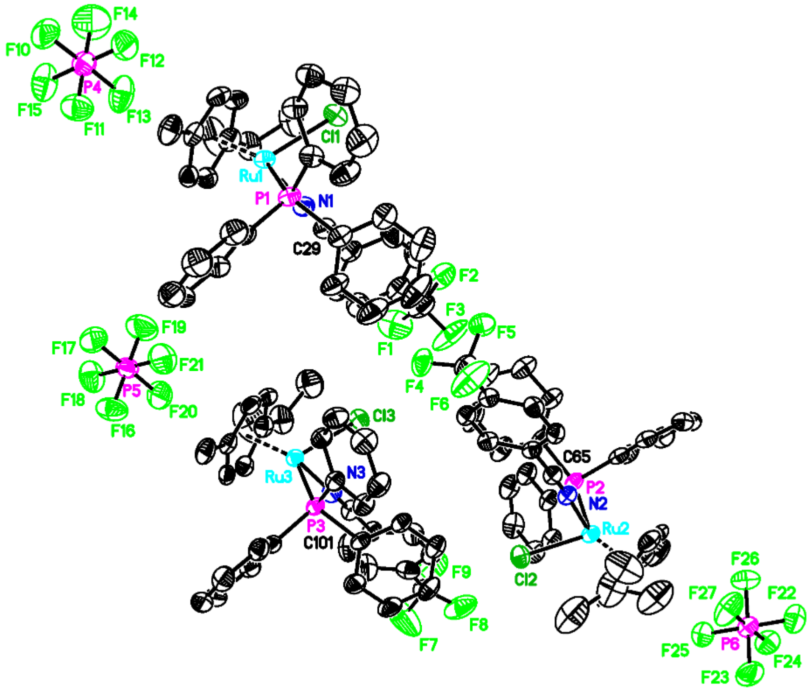

Figure S126. Full solid-state structure of **Ru^{H}-NCAr^{CF3}**. Hydrogen atoms omitted for clarity. Displacement ellipsoids shown at the 50% probability level. Data collected at 100 K.

Refinement details for *trans-mer*-C^{H}-NCAr^{OMe}

The structure for *trans*-*mer*-**C^{H}-NCAr^{OMe}** displayed substantial disorder in several portion of the ligand scaffolds. Disorder in one of the Ph rings on the P1 PPh_3 ligand was modeled as two orientations (rotated by ~50°) with 51:49 occupancy. Two of the benzonitrile ligands (bound to Ru via N2 and N3, respectively) were also disordered; each was modeled as two orientations of the entire benzonitrile ligand, with 60:40 and 52:48 occupancy, respectively. The anisotropic parameters of the nitrogen atoms in each disordered benzonitrile frameworks (N2A and N2B, N3A and N3C) was constrained using EADP commands. The outer-sphere BF_4^- counteranion was disordered over two positions with 51:49 occupancy. The structure also contained two co-crystallized solvent molecules, a $CHCl_3$ and an H_2O, each with 50% occupancy; these molecules were located in channels limited by benzonitrile arene groups on adjacent complexes, and were thus substantially disordered. The position and anisotropic displacement parameters of the carbon atom in the $CHCl_3$ molecule (C61) were restrained using DFIX and SIMU commands. Anisotropic refinement of the O atom in the water molecule (O4) led to poor convergence; therefore, this atom was refined isotropically.

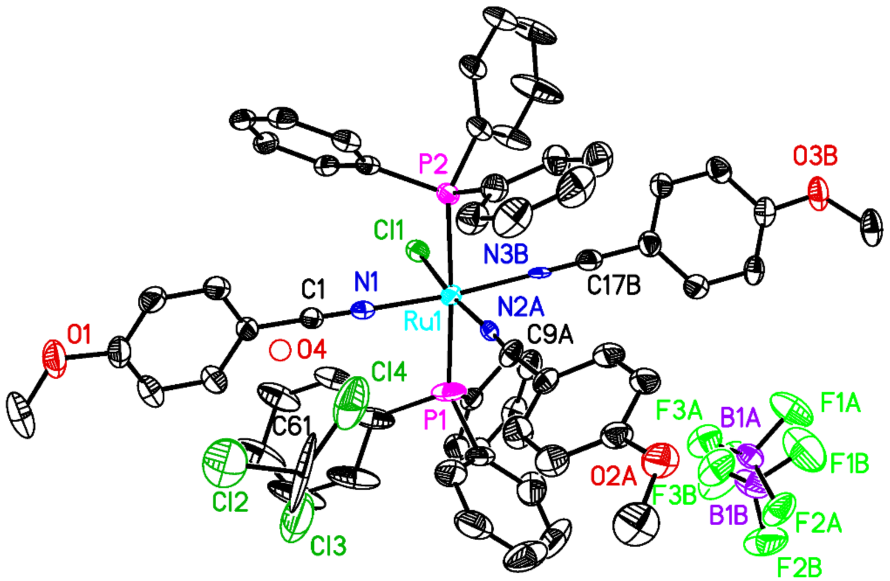

Figure S127. Solid-state structure of *trans-mer*-**C^{H}-NCAr^{OMe}**. Hydrogen atoms omitted for clarity. Only only the major orientations (50-60%) of disordered fragments (two benzonitrile ligands and one Ph ring on PPh_3 ligand) shown for clarity. Displacement ellipsoids shown at the 50% probability level. Data collected at 100 K.

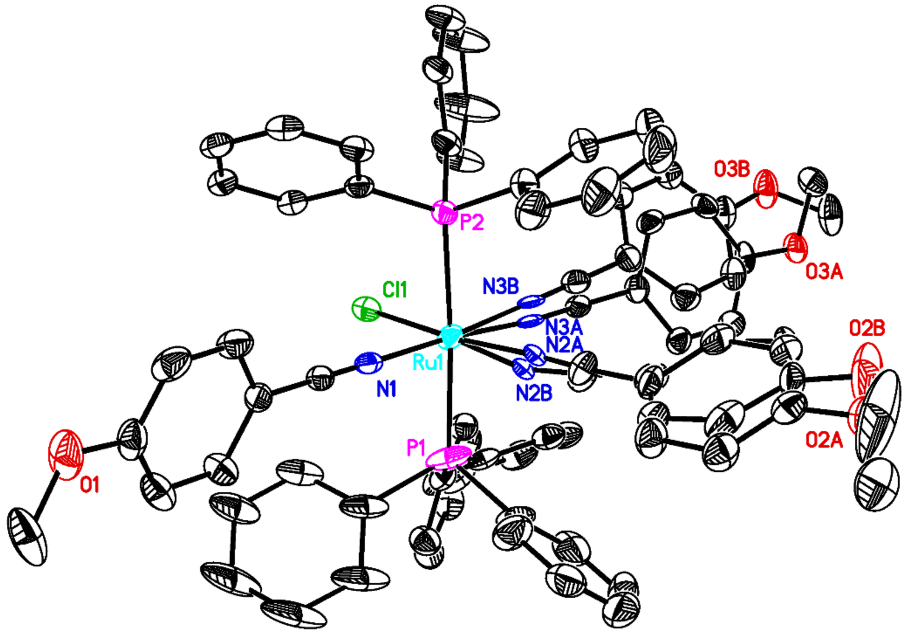

Figure S128. Solid-state structure of the cationic portion of *trans-mer*-**C^{H}-NCAr^{OMe}**. Hydrogen atoms, outer-sphere BF_4^- counteranion, and co-crystallized $CHCl_3$ and H_2O solvent molecules not shown for clarity. Displacement ellipsoids shown at the 50% probability level. Data collected at 100 K.

Preliminary structure for *trans-mer*-C^H-NCCH₃

Attempts to grow XRD-quality crystals of *trans-mer*- **C^H-NCCH$_3$** under various conditions were broadly unsuccessful, in part due to the high solubility of this compound in CH_3CN. Storage of a concentrated solution of *trans-mer*- **C^H-NCCH$_3$** in CH_3CN at –10 °C for several weeks did lead to formation small crystals as thin yellow blades that were prone to solvent loss when removed from solution. Still, XRD analysis of one of these crystals stored under Et_2O did provide a dataset of sufficient quality for partial refinement; based on this dataset, connectivity between the different atoms in the complex could be established, confirming the *trans* arrangement of the PPh_3 ligands and the *mer* arrangement of the three CH_3CN moieties.

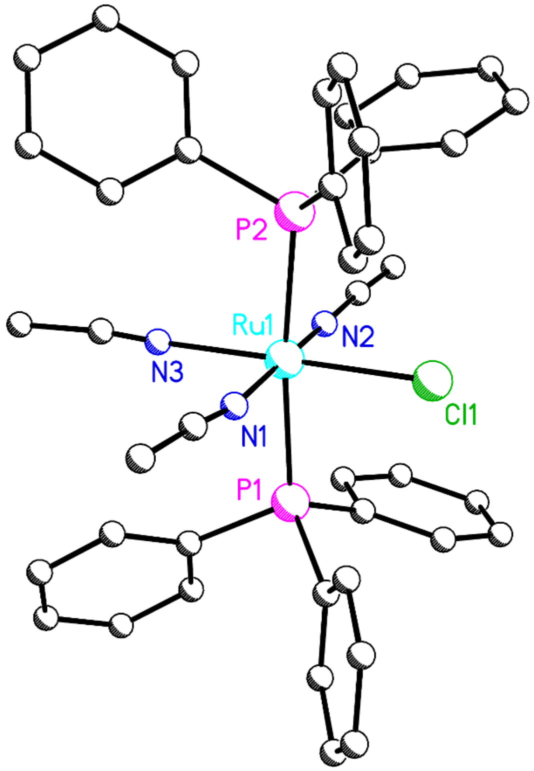

Figure S129. Preliminary XRD structure for the *trans-mer* isomer of complex **C^H-NCCH$_3$.**

References

(1) Micci, A. N.; Fumo, J. E.; Pike, R. D.; Lionetti, D., Effects of Substituted Triarylphosphine Ligands on Electron Transfer in [(*p*-Cymene)Ru] Complexes. *Organometallics* **2024**, *43*, 1912-1921.
(2) Ortiz-Pastrana, N.; Cervantes-Vásquez, M.; Picasso, M. T. C.; Paz-Sandoval, M. A., Comparative study of half-sandwich (η6-arene)$RuCl_2(P(OMe)_3)$ (p-cymene, C_6Me_6) towards AgOTf and the influence of coordinated solvents in the chemistry of p-cymene derivatives. *J. Organomet. Chem.* **2026**, 124069.
(3) Swaminathan, S.; Haribabu, J.; Balakrishnan, N.; Vasanthakumar, P.; Karvembu, R., Piano stool Ru(II)-arene complexes having three monodentate legs: A comprehensive review on their development as anticancer therapeutics over the past decade. *Coord. Chem. Rev.* **2022**, *459*, 214403.
(4) Fogg, D. E.; James, B. R., Chiral and achiral diphosphine complexes of ruthenium(II) incorporating labile nitrile ligands: Synthesis and solution chemistry of mono- and dinuclear derivatives of $Ru_2Cl_4(PP)_2$ (PP = chelating diphosphine). *Inorg. Chem.* **1997**, *36*, 1961-1966.
(5) Fulmer, G. R.; Miller, A. J. M.; Sherden, N. H.; Gottlieb, H. E.; Nudelman, A.; Stoltz, B. M.; Bercaw, J. E.; Goldberg, K. I., NMR Chemical Shifts of Trace Impurities: Common Laboratory Solvents, Organics, and Gases in Deuterated Solvents Relevant to the Organometallic Chemist. *Organometallics* **2010**, *29*, 2176-2179.
(6) Lackner, W.; Schmid, R.; Kirchner, K.; Mereiter, K., *CSD Communication* **2004**
(7) Patra, S. K.; Majumdar, M.; Bera, J. K., Ligand assisted homolytic cleavage of the Ru–Ru single bond in $[Ru_2(CO)_4]^{2+}$ core and the chemical consequence. *J. Organomet. Chem.* **2006**, *691*, 4779-4787.
(8) Kuo, Y. Y.; Haddow, M. F.; Jamieson, A. L.; Owen, G. R., Synthesis, structural characterisation and catalytic application of dichloro(η^6-*p*-cymene){diphenyl(3-methyl-2-indolyl)phosphine}ruthenium(II) in the transfer hydrogenation of ketones. *Transition Met. Chem.* **2013**, *38*, 641-648.
(9) Abramov, P. A., *CSD Communication* **2016**
(10) Cruz, S. S.; Amenta, D. S.; Gilje, J. W.; Yap, G. P. A., A re-examination of the reaction of dichloro-tris-(triphenylphosphino)ruthenium(II) with benzonitrile: Crystal structures of triphenylphosphine chloro ruthenium complexes. *Polyhedron* **2016**, *114*, 179-183.
(11) da Silva, J. P.; Fuganti, O.; Kramer, M. G.; Facchin, G.; Aquino, L. E. N.; Ellena, J.; Back, D. F.; Gondim, A. C. S.; Sousa, E. H. S.; Lopes, L. G. F.; Machado, S.; Guimarães, I. D. L.; Wohnrath, K.; de Araujo, M. P., Electrochemical, mechanistic, and DFT studies of amine derived diphosphines containing Ru(II)–cymene complexes with potent in vitro cytotoxic activity against HeLa and triple-negative breast cancer cells MDA-MB-231. *Dalton Trans.* **2020**, *49*, 16498-16514.
(12) Coe, B. J.; Glenwright, S. J., Trans-effects in octahedral transition metal complexes. *Coord. Chem. Rev.* **2000**, *203*, 5-80.
(13) Gilbert, J. D.; Wilkinson, G., New complexes of ruthenium(II) with triphenylphosphine and other ligands. *J. Chem. Soc. A* **1969**, 1749-1753.
(14) Al-Far, A. M.; Slaughter, L. M., *cis-cis-trans*-Bis(acetonitrile-κ*N*)-dichloridobis(triphenylphosphine-κ*P*)-ruthenium(II) acetonitrile disolvate. *Acta Crystallogr., Sect. E: Struct. Rep. Online* **2008**, *64*, M184-U1762.
(15) Laplaca, S. J.; Ibers, J. A., A Five-Coordinated d^6 Complex: Structure of Dichlorotris(Triphenylphosphine)Ruthenium(II). *Inorg. Chem.* **1965**, *4*, 778-783.
(16) Hansch, C.; Leo, A.; Taft, R. W., A survey of Hammett substituent constants and resonance and field parameters. *Chem. Rev.* **1991**, *91*, 165-195.
(17) Guimaraes, I. D. L.; Marszaukowski, F.; Rutka, P. B.; Borge, L. F.; Ribeiro, R. A. P.; de Lazaro, S. R.; Castellen, P.; Sagoe-Wagner, A.; Golsteyn, R. M.; Boere, R. T.; Wohnrath, K., Synthesis, characterization and anticancer activities of cationic η^6-cymene ruthenium(II) complexes containing phosphine and nitrogenous ligands. *Polyhedron* **2022**, *224*, 115980.
(18) Kaim, W.; Reinhardt, R.; Waldhor, E.; Fiedler, J., Electron transfer and chloride ligand dissociation in complexes $[(C_5Me_5)ClM(bpy)]^+/[(C_5Me_5)M(bpy)]^n$ (M=Co, Rh, Ir; n=2+,+,0,–): A combined electrochemical and spectroscopic investigation. *J. Organomet. Chem.* **1996**, *524*, 195-202.

(19) Lionetti, D.; Day, V. W.; Blakemore, J. D., Synthesis and Electrochemical Properties of Half-Sandwich Rhodium and Iridium Methyl Complexes. *Organometallics* **2017**, *36*, 1897-1905.
(20) Hopkins Leseberg, J. A.; Henke, W. C.; Blakemore, J. D., 1.09 - Electrochemistry in Organometallic Chemistry. In *Comprehensive Organometallic Chemistry IV*; Parkin, G., Meyer, K., O'hare, D., Eds.; Elsevier: Oxford, 2022, pp. 249-283.
(21) Grounds, H.; Anderson, J. C.; Hayter, B.; Blake, A. J., A New Oxidative Addition of Ruthenium(0) into an Aryl Halide Bond and Subsequent Intermolecular C−H Insertion. *Organometallics* **2009**, *28*, 5289-5292.
(22) Smith, P. D.; Wright, A. H., Synthesis and structure of a chelating arene-ruthenium complex $[RuCl_2(PPh_2(CH_2)_3\text{-}\eta^6\text{-}C_6H_5)]$. *J. Organomet. Chem.* **1998**, *559*, 141-147.
(23) Phillips, G.; Hermans, S.; Adams, J. R.; Johnson, B. F. G., Towards the synthesis of a metal hamburger complex: the interaction of the $PPh_2(CH_2)_3Ph$ ligand with ruthenium clusters. *Inorg. Chim. Acta* **2003**, *352*, 110-120.
(24) Polam, J. R.; Porter, L. C., Arene complexes of Ru(II) Part I. Synthesis, characterization and X-ray crystal structure of $[(\eta^6\text{-}C_6H_5CH_3)RuCl(PPh_3)_2][BF^4]$. *Inorg. Chim. Acta* **1993**, *205*, 119-121.
(25) Pereira, S. A. P.; Romano-deGea, J.; Barbosa, A. I.; Costa Lima, S. A.; Dyson, P. J.; Saraiva, M. L. M. F. S., Fine-tuning the cytotoxicity of ruthenium(ii) arene compounds to enhance selectivity against breast cancers. *Dalton Trans.* **2023**, *52*, 11679-11690.
(26) Lalrempuia, R.; Carroll, P. J.; Kollipara, M. R., Syntheses of $[(\eta^6\text{-}p\text{-cymene})Ru(EPh_3)_2Cl]^+$ complexes and molecular structure of chloro(η^6-*p*-cymene)-bis(triphenylphosphine)ruthenium(II) tetrafluoroborate (E = P, As and Sb). *J. Coord. Chem.* **2003**, *56*, 1499-1504.
(27) Chaplin, A. B.; Dyson, P. J., Catalytic activity of bis-phosphine ruthenium(II)-arene compounds: Structure-activity correlations. *Organometallics* **2007**, *26*, 2447-2455.
(28) Macrae, C. F.; Sovago, I.; Cottrell, S. J.; Galek, P. T. A.; McCabe, P.; Pidcock, E.; Platings, M.; Shields, G. P.; Stevens, J. S.; Towler, M.; Wood, P. A., Mercury 4.0: from visualization to analysis, design and prediction. *J. Appl. Crystallogr.* **2020**, *53*, 226-235.
(29) Naskar, S.; Bhattacharjee, M., Ruthenium cationic species for transfer hydrogenation of aldehydes: Synthesis and catalytic properties of $[(PPh_3)_2Ru(CH_3CN)_3Cl]^+[A]^-$ {A = BPh_4 or ClO_4} and $[(PPh_3)_2Ru(CH_3CN)_3Cl]^+[BPh_4]^-$ *J. Organomet. Chem.* **2005**, *690*, 5006-5010.
